# Quantifying label enrichment from two mass isotopomers increases proteome coverage for in vivo protein turnover using heavy water metabolic labeling

**DOI:** 10.1038/s42004-023-00873-x

**Published:** 2023-04-17

**Authors:** Henock M. Deberneh, Doaa R. Abdelrahman, Sunil K. Verma, Jennifer J. Linares, Andrew J. Murton, William K. Russell, Muge N. Kuyumcu-Martinez, Benjamin F. Miller, Rovshan G. Sadygov

**Affiliations:** 1 Department of Biochemistry and Molecular Biology, The University of Texas Medical Branch, Galveston, TX USA; 2 Department of Surgery, The University of Texas Medical Branch, Galveston, TX USA; 3 Sealy Center on Aging, The University of Texas Medical Branch, Galveston, TX USA; 4 Department of Neuroscience, Cell Biology and Anatomy, The University of Texas Medical Branch, Galveston, TX USA; 5 Oklahoma Medical Research Foundation, Oklahoma Nathan Shock Center, Oklahoma Center for Geosciences, Harold Hamm Diabetes Center, Oklahoma City, OK USA; 6 Oklahoma City Veterans Association, Oklahoma City, OK USA; 7 Present Address: Department of Molecular Physiology and Biological Physics, The University of Virginia, Charlottesville, VA USA

**Keywords:** Proteome, Mass spectrometry, Peptides, Networks and systems biology, Protein-protein interaction networks

## Abstract

Heavy water metabolic labeling followed by liquid chromatography coupled with mass spectrometry is a powerful high throughput technique for measuring the turnover rates of individual proteins in vivo. The turnover rate is obtained from the exponential decay modeling of the depletion of the monoisotopic relative isotope abundance. We provide theoretical formulas for the time course dynamics of six mass isotopomers and use the formulas to introduce a method that utilizes partial isotope profiles, only two mass isotopomers, to compute protein turnover rate. The use of partial isotope profiles alleviates the interferences from co-eluting contaminants in complex proteome mixtures and improves the accuracy of the estimation of label enrichment. In five different datasets, the technique consistently doubles the number of peptides with high goodness-of-fit characteristics of the turnover rate model. We also introduce a software tool, d2ome+, which automates the protein turnover estimation from partial isotope profiles.

## Abstract (web summary)

Heavy water metabolic labeling followed by liquid chromatography coupled mass spectrometry (LC-MS) is a powerful approach to characterize in vivo protein turnover rates, however, peptide co-elution causes overlap of their isotope profiles in LC-MS and affects the proteome coverage. Here, the authors develop an approach to increase the proteome coverage for in vivo protein turnover by using partial isotope profiles from two mass isotopomers.

## Introduction

Protein turnover plays a key role in maintaining protein homeostasis^1^. It is important to healthy biological functioning^2^ and is often dysregulated in diseases^3,4^. Metabolic labeling of live animals with stable isotopes followed by liquid chromatography coupled with mass spectrometry (LC-MS) and combined with sophisticated data processing algorithms has been a powerful tool for estimating the turnover of individual proteins in high-throughput and large-scale studies^5,6^. The labeling agents can be divided into two groups. The amino acid-based labeling^7–10^ uses a diet enriched in stable isotope labeled essential amino acids such as ^13^C_6_-Lys. The label incorporation results in separate mass profiles of unlabeled and labeled forms of a peptide. Only peptides containing the heavy amino acid are useful for protein turnover rate. Non-canonical amino acids have also been used to study the protein turnover in LC-MS^11,12^.

The atom-based labeling uses a diet enriched in heavy^13–17^ (such as ^13^C, ^15^N, ^2^H) or light^18^ (^12^C) atoms. In a strategy named SILAM^5^ (stable isotope labeling of mammals), rats and mice have been metabolically labeled with a diet containing ^15^N-labeled spirulina. Turnover rates of individual proteins in mouse brain and liver have been reported^14^. The atom-based labeling results in composite spectra of unlabeled and labeled forms of a peptide. Therefore, the estimation of label enrichment resulting from atom-based labeling agents is computationally more complex^13^.

An alternative approach to protein turnover study is to characterize a protein based on its expression as a fusion protein with an appropriate tag^19^. Fluorescent proteins (FPs) have been widely exploited tags. Bleach-chase and photoactivation/photoconversion of FPs permit monitoring fluorescence (and estimating protein half-live) after the bleaching or change in fluorescent light color from the newly synthesized proteins in cell culture^20^. Tandem fluorescent proteins have also been developed and applied^21^. However, in living animals, photoactivation may not be easily achieved. SNAP-tag^22^ overcame this problem and was used to measure protein turnover in transgenic mice. FP tagging allows the detection of expression levels of target proteins. A technique termed Global protein stability, integrated fluorescence-based protein stability analysis, and the DNA microarray technology^23^. The stabilities of thousands of proteins in cell culture have been estimated^23^.

A classical method for estimating protein degradation in eukaryotes is the chemical inhabitation of the ribosome translocation with cycloheximide^24^. Protein abundances are measured by Western blot, using antibodies for target proteins. The dependence on antibody-based quantification limits the throughput of the method. A comprehensive review of the techniques used for protein turnover studies can be found elsewhere^25–27^.

Among the stable isotope labeling agents, heavy water (^2^H_2_O) is easy to use, biologically safe at low enrichments, relatively inexpensive, and does not require diet adaptation^28^. The deuterium (^2^H) incorporation from heavy water labeling has been monitored in vivo by stimulated Raman scattering microscopy^29^. The vibrational frequency of C-D bond is located in a cell-silent spectral window in which no other Raman peaks exists^30^. The technique is non-invasive and non-destructive. It allows for microscopic imaging of lipid and protein synthesis in vivo. However, currently, it does not provide the ability to determine the turnover rate of individual proteins.

Deuterium in heavy water is incorporated into all non-essential amino acids^31^. The gradual incorporation of the deuterium into a peptide decreases the relative isotope abundance (RIA) of its monoisotope. The monoisotopic RIA is computed from the complete isotope profile, which comprises up to six mass isotopomers. The time course of the RIA is modeled with an exponential decay function^32–36^. The coefficient of determination, R^2^, is often used as a measure of the goodness-of-fit (GOF) between the experimental data and theoretical curve fitting^36,37^. This approach can produce turnover rates of thousands of proteins; however, it has been observed that only 35–45% of all quantified peptides have been useful for the estimation of protein turnover rates^37,38^. For the rest of the quantified peptides, the R^2^ is too low (R^2^ < 0.8). One of the factors affecting the estimation of label incorporation is the complexity of the mammalian proteome; the isotope profiles of many peptides overlap in LC-MS. Since abundances of up to six mass isotopomers of a peptide are required for most approaches^36,39^, the chances of co-elution affecting the estimation of monoisotopic RIA are high.

This work develops an approach to increase the proteome coverage for protein turnover by using partial isotope profiles. The basis of this strategy is the estimation of label enrichment of a peptide from any pair of its mass isotopomers. We provide theoretical formulas for the time courses of six mass isotopomers. Here, the formulas are utilized to determine label enrichment, followed by protein turnover rate estimations. Previously, Papageorgopoulos^32^ and colleagues have shown that the label enrichment during metabolic labeling can be modeled as linear regression on the ratios of pairs of mass isotopomers. The mass isotopomer abundances, in turn, were modeled as polynomials of the enrichment. The formulas presented in this work provide the polynomials and their coefficients analytically. They allow computationally efficient estimations of label enrichment in data analysis of large-scale datasets.

We applied the method to five datasets acquired on Orbitrap Eclipse and Orbitrap Q Exactive HF mass spectrometers. The Orbitrap Eclipse dataset (acquired for this study from murine liver tissue) was used as the developmental dataset to compute RIA from partial isotope profiles and compare it with the traditional approach. The other datasets (Orbitrap Q Exactive HF) are from a recent study^36^, which generated LC-MS data of heavy water labeled samples of four murine tissue types. The approach using two mass isotopomers (partial isotope profiles) has consistently increased the number of quantified peptides in both datasets and across all tissue types.

## Results

**Fig. 1:**
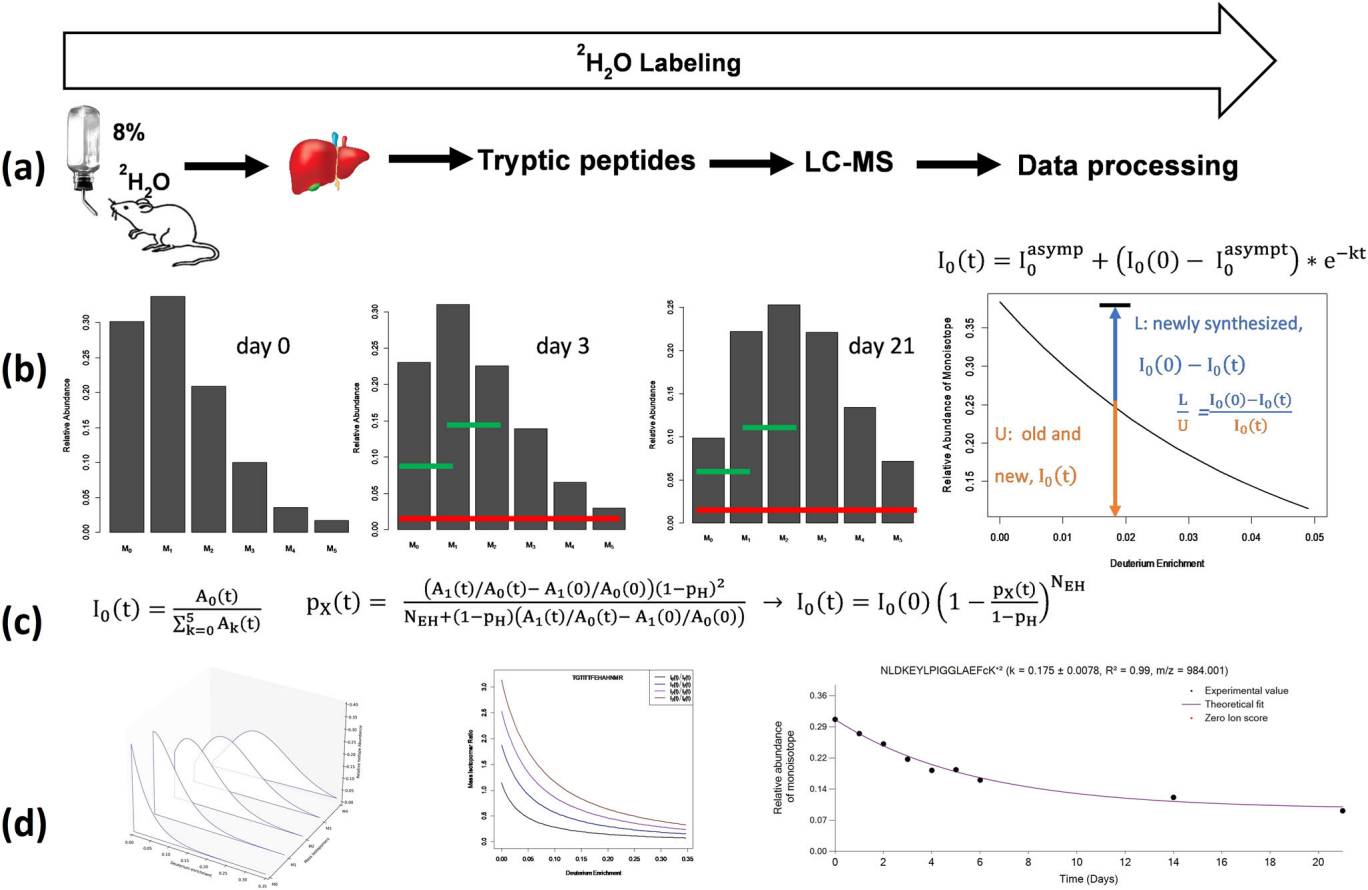
An approach to estimate protein turnover rates from the abundances of two mass isotopomers. **a** Experimental and workflow of metabolic labeling, LC-MS, and data processing. **b** Relative abundances of mass isotopomers shifted during the gradual incorporation of deuterium from heavy water. Shown are the isotope profiles of peptide, NLDKEYLPIGGLAEFCK, at three labeling durations: 0, 3, and 21 days. The turnover rate is determined from the depletion of the monoisotopic RIA, I_0_(t). **c** The monoisotopic RIA is traditionally obtained as normalized monoisotopic abundance from the complete isotope profile (red bar). This work determines the monoisotopic RIA from only two mass isotopomers (green bars). At first, the deuterium enrichment of a peptide is determined from two mass isotopomers. Then the deuterium enrichment is used to calculate the monoisotopic RIA. The time course of I_0_(t) is fit to an exponential decay function to obtain the turnover rate. **d** The RIAs of the first five mass isotopomers of the peptide as a function of deuterium enrichment. The I_k-1_(t)/I_k_(t) ratios. The experimental values (black circles) and theoretical fit (purple) for the I_0_(t) of the peptide. I_0_(0) is the monoisotopic RIA of the unlabeled peptide. N_EH_ is the number of hydrogens accessible to deuterium in drinking water. p_X_(t) is the deuterium enrichment of a peptide at the labeling time point t. A_i_(t) is the raw (non-normalized) abundance of the i^th^ mass isotopomer measured in LC-MS. L– labeled, Uunlabeled, LC liquid chromatography, MS mass spectrometry.

Protein turnover rates are computed using the monoisotopic RIA, I_0_(t), obtained from LC-MS data of heavy water metabolically labeled peptides. At every time point of labeling, the monoisotopic RIA is determined as the normalized abundance of the monoisotope from the complete isotope profile of a peptide^36,37^, Supplementary Eq. (1). The time series of the monoisotopic RIA of a peptide is modeled as an exponential decay function to obtain the turnover rate, Fig. 1.

1 $${{{{{{\rm{I}}}}}}}_{0}\left({{{{{\rm{t}}}}}}\right)={{{{{{\rm{I}}}}}}}_{0}^{{{{{{\rm{asymp}}}}}}}+\left({{{{{{{\rm{I}}}}}}}_{0}\left(0\right)-{{{{{\rm{I}}}}}}}_{0}^{{{{{{\rm{asymp}}}}}}}\right){{{{{{\rm{e}}}}}}}^{-{{{{{\rm{kt}}}}}}}$$

In Eq. (1), I_0_(0) is the monoisotopic RIA of the unlabeled peptide, I_0_^asymp^ is the monoisotopic RIA achieved at the plateau of labeling, t is the labeling duration, and k is the turnover rate (also referred to as degradation rate constant^26,40^). I_0_^asymp^ is calculated from the number of hydrogens accessible to deuterium from heavy water (N_EH_), the deuterium enrichment of body water (p_W_), and the natural abundance of deuterium (p_H_) as shown in Supplementary Eq. (10). GOF characteristics such as the coefficient of determination (R^2^), Pearson correlation, and residual standard error are used to evaluate the quality of the theoretical fit to the experimental data. Normally, R^2^ > 0.8 is used to filter peptides^36,37^. The residual standard error is used to compute the confidence interval of the turnover rate, k (Supplementary Eqs. (8), (9), and (11)).

The exponential decay model relies on the accurate estimations of the monoisotopic RIAs. Since mammalian proteome samples are complex, their peptides often coelute, and isotope profiles overlap. Examples of overlapping isotope profiles are provided in Supplementary Figs. 1–5. The overlaps resulted in inaccurate estimations of the monoisotopic RIA when complete isotope profiles were used. However, the label enrichment could accurately be estimated from the non-affected mass isotopomers which can then be used to reconstruct the monoisotopic RIA (as will be shown below). For example, the deuterium enrichment, p_X_(t), is obtained, in the equation for the ratio of abundances of the second (A_2_(t)) and first heavy mass isotopomers (A_1_(t)):

2 $$\frac{{{{{{{\rm{A}}}}}}}_{2}\left({{{{{\rm{t}}}}}}\right)}{{{{{{{\rm{A}}}}}}}_{1}\left({{{{{\rm{t}}}}}}\right)}=\left\{\frac{{{{{{{\rm{I}}}}}}}_{2}\left(0\right)}{{{{{{{\rm{I}}}}}}}_{0}\left(0\right)}-\frac{{{{{{{\rm{I}}}}}}}_{1}\left(0\right)}{{{{{{{\rm{I}}}}}}}_{0}\left(0\right)}{{{{{{\rm{b}}}}}}}_{1}\left(0\right)+\frac{{{{{{{\rm{N}}}}}}}_{{{{{{\rm{EH}}}}}}}+1}{{{{{{{\rm{N}}}}}}}_{{{{{{\rm{EH}}}}}}}-1}\left({{{{{{\rm{b}}}}}}}_{2}\left(0\right)-{{{{{{\rm{b}}}}}}}_{2}\left({{{{{\rm{t}}}}}}\right)\right)\right\}{{{{{{\rm{I}}}}}}}_{0}\left({{{{{\rm{t}}}}}}\right)+{{{{{{\rm{b}}}}}}}_{1}\left({{{{{\rm{t}}}}}}\right)$$

b_n_(t) is defined as:

$${{{{{{\rm{b}}}}}}}_{{{{{{\rm{n}}}}}}}\left({{{{{\rm{t}}}}}}\right)=\left(\begin{array}{c}{{{{{{\rm{N}}}}}}}_{{{{{{\rm{EH}}}}}}}\\ {{{{{\rm{n}}}}}}\end{array}\right){\left(\frac{{{{{{{\rm{p}}}}}}}_{{{{{{\rm{X}}}}}}}\left({{{{{\rm{t}}}}}}\right)+{{{{{{\rm{p}}}}}}}_{{{{{{\rm{H}}}}}}}}{1-{{{{{{\rm{p}}}}}}}_{{{{{{\rm{H}}}}}}}-{{{{{{\rm{p}}}}}}}_{{{{{{\rm{X}}}}}}}\left({{{{{\rm{t}}}}}}\right)}\right)}^{{{{{{\rm{n}}}}}}}$$

In the above equation, A_i_(t) is the raw (non-normalized) abundance of the i^th^ mass isotopomer. Numerically, p_X_(t) is determined by minimizing the absolute value of the difference between experimental, $\frac{{{{{{{\rm{A}}}}}}}_{2}^{{{{{{\rm{expr}}}}}}}({{{{{\rm{t}}}}}})}{{{{{{{\rm{A}}}}}}}_{1}^{{{{{{\rm{expr}}}}}}}({{{{{\rm{t}}}}}})}$, and theoretical $\frac{{{{{{{\rm{A}}}}}}}_{2}\left({{{{{\rm{t}}}}}}\right)}{{{{{{{\rm{A}}}}}}}_{1}\left({{{{{\rm{t}}}}}}\right)}$, ratios from Eq. (2):

$${p}_{X}(t)={\arg }\mathop{{{\min }}}\limits_{{{{{{{\rm{p}}}}}}}_{{{{{{\rm{X}}}}}}}\left({{{{{\rm{t}}}}}}\right)}\left|\frac{{{{{{{\rm{A}}}}}}}_{2}\left({{{{{\rm{t}}}}}}\right)}{{{{{{{\rm{A}}}}}}}_{1}\left({{{{{\rm{t}}}}}}\right)}-\frac{{{{{{{\rm{A}}}}}}}_{2}^{{{{{{\rm{expr}}}}}}}({{{{{\rm{t}}}}}})}{{{{{{{\rm{A}}}}}}}_{1}^{{{{{{\rm{expr}}}}}}}({{{{{\rm{t}}}}}})}\right|{subject\; to}:{{{{{{\rm{p}}}}}}}_{{{{{{\rm{X}}}}}}}(t)\in \left[0{{{{{\rm{;}}}}}}{{{{{{\rm{p}}}}}}}_{{{{{{\rm{W}}}}}}}\right]$$

The analytical formulas for the ratios of other mass isotopomers (A_2_(t)/A_1_(t), A_1_(t)/A_0_(t), A_3_(t)/A_0_(t), and A_4_(t)/A_0_(t)) are provided in the Supplementary Notes. We have implemented this technique in the software tool d2ome^35^. The updated version of the tool will be referred to as d2ome + . The main differences from the previous version are the ability to quantify the label enrichment from two mass isotopomers and the computation of the confidence intervals. d2ome+ is available at GitHub, https://github.com/rgsadygov/d2ome.

Once p_X_(t) is determined from a ratio of pair of raw abundances (from Eq. (2), and Supplementary Eqs. (2), (3) for A_1_(t)/A_0_(t), A_2_(t)/A_0_(t)), the RIA of the monoisotope, $\widetilde{{{{{{{\rm{I}}}}}}}_{0}\left({{{{{\rm{t}}}}}}\right)}$, is reconstructed as:

3 $$\widetilde{{{{{{{\rm{I}}}}}}}_{0}\left({{{{{\rm{t}}}}}}\right)}={{{{{{\rm{I}}}}}}}_{0}\left(0\right){\left(1-\frac{{{{{{{\rm{p}}}}}}}_{{{{{{\rm{X}}}}}}}\left({{{{{\rm{t}}}}}}\right)}{1-{{{{{{\rm{p}}}}}}}_{{{{{{\rm{H}}}}}}}}\right)}^{{{{{{{\rm{N}}}}}}}_{{{{{{\rm{EH}}}}}}}}$$

The value of $\widetilde{{{{{{{\rm{I}}}}}}}_{0}\left({{{{{\rm{t}}}}}}\right)}$ is used in the time course data to fit the exponential decay function, Eq. (1). Thus, the monoisotopic RIA is estimated from the abundances of two mass isotopomers, instead of the completed isotope profile used by the traditional approach.

**Fig. 2:**
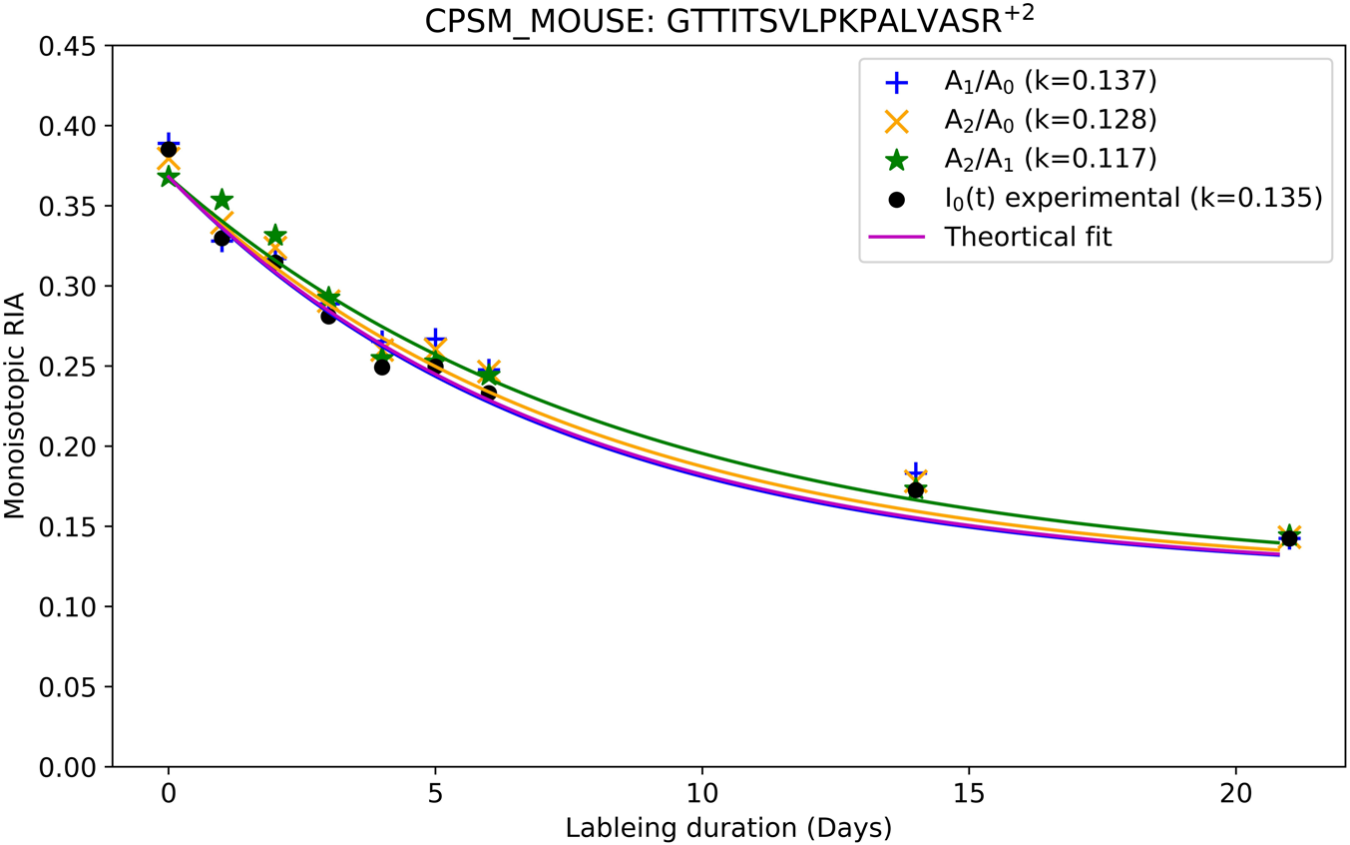
An approach using two mass isotopomers accurately reproduces the monoisotopic RIA from the complete isotope profiles. The time series of monoisotopic RIAs (y-axis) is shown along the labeling duration (x-axis). The turnover rates, which were computed by using the reconstruction of the RIAs by each one of the three ratios, A_1_(t)/A_0_(t) (blue pluses), A_2_(t)/A_0_(t) (yellow crosses), and A_2_(t)/A_1_(t) (green stars), agreed to up to 14% with the turnover rate (0.135 day^−1^) calculated using the complete isotope profile. Solid lines show the fits from the corresponding (based on the color of the data points) label enrichment determination technique. CPSM_MOUSE – mouse protein carbamoyl-phosphate synthase (ammonia), mitochondrial.

Figure 2 presents an example of the implementation of the approach for the CPMS_MOUSE peptide, GTTITSVLPKPALVASR. It shows the monoisotopic RIAs estimated from the complete isotope profile (black circles), A_1_(t)/A_0_(t) (blue pluses), A_2_(t)/A_0_(t) (green stars), and A_2_(t)/A_1_(t) (yellow crosses) ratios. The rates computed using the time series of reconstructed $\widetilde{{{{{{{\rm{I}}}}}}}_{0}\left({{{{{\rm{t}}}}}}\right)}$ by utilizing the label enrichment estimations from the ratio of raw abundance of two mass isotopomers ranged from 0.117 day^−1^ to 0.137 day^−1^. The turnover rate computed from the complete isotope profile was 0.135 day^−1^. In the Supplementary Data 1, there are RIA time series data for 1227 distinct peptide sequences (amino acid sequence, charge, post-translational modifications) which showed improvements in the GOF characteristics (R^2^) after the application of the method. The first page of the file includes improved RIAs estimations of peptides from Supplementary Figs. 1–5.

### The validations of monoisotopic RIAs and turnover rates estimated from partial isotope profiles

The Orbitrap Eclipse dataset was used to validate the use of partial isotope profiles for estimation of the monoisotopic RIAs and turnover rates. First, we compared the values of I_0_(t) from complete isotope profiles with those obtained by using a pair of mass isotopomers. The comparison was made for peptides with high GOF characteristics to the experimental data (R^2^ ≥ 0.95). Further filtering was done to restrict the peptides to those whose turnover rates fit the range of shortest (1 day) and longest durations of the labeling (21 days), e.g., 0.05 ≤ k ≤ 0.6. There were 1928 distinct peptides that passed the thresholds. It is assumed that the estimations of the monoisotopic RIAs from the complete isotope profiles were accurate, and the isotope profiles of these peptides served as a validation set for the method using only two mass isotopomers. At every time point of labeling, $\widetilde{{{{{{{\rm{I}}}}}}}_{0}\left({{{{{\rm{t}}}}}}\right)}$ was computed using Eq. (3) and compared with that obtained from full isotope profile, Supplementary Eq. (1). The density of the relative differences, $({{{{{{\rm{I}}}}}}}_{0}({{{{{\rm{t}}}}}})-\widetilde{{{{{{{\rm{I}}}}}}}_{0}({{{{{\rm{t}}}}}})})/{{{{{{\rm{I}}}}}}}_{0}({{{{{\rm{t}}}}}})$, is plotted in Supplementary Fig. 6. As the figure shows, the distribution is bell-shaped with a single mode at zero. The relative RIA differences did not exceed 10% of the RIA computed from the complete isotope profile, Supplementary Eq. (1). The final width of the distribution is attributed to the fluctuations in measurements of the mass isotopomer abundances^41^. The results showed that for high-quality spectral data, the values of the monoisotopic RIA, I_0_(t), computed from complete or partial isotope profiles, agreed well and validated our approach for using partial isotope profiles for estimating the monoisotopic RIA in experimental data.

Next, the turnover rates obtained from partial and complete isotope profiles were compared. For the high-quality dataset (R^2^ ≥ 0.95), we used Eq. (3) to compute turnover rates obtained from the reconstructed RIAs. The rates were compared with those obtained from RIAs computed from complete isotope profiles. Supplementary Fig. 7 shows the relative differences between turnover rates obtained by the original approach (using a full isotope profile to compute the monoisotopic RIA) and those obtained by using a pair of mass isotopomers, $\widetilde{{{{{{{\rm{I}}}}}}}_{0}\left({{{{{\rm{t}}}}}}\right)}$. The density of the distribution was zero-centered with a width about 20%. These data show that our approach using two mass isotopomers for computing the monoisotopic RIA can, in practice, be used to estimate the protein turnover rates. Supplementary Fig. 8 is the scatter plot of the relative errors of the turnover rates and I_0_(t) estimations from a pair of mass isotopomers. As seen from the figure, the joint distribution is centered around zero on both axes. The Pearson correlation between the relative differences was 0.11. It indicates that there was no systematic error in estimation of the turnover rate from the reconstructed monoisotopic RIA.

The monoisotopic RIAs obtained by using the A_1_(0)/A_0_(0), A_2_(0)/A_1_(0), and A_2_(0)/A_0_(0) ratios and complete isotope profiles were tested to determine how often each one of them improved the RIA estimations. The test determines if there is any redundancy in the estimations. Isotope profiles of non-labeled peptides were used because, for them, the accurate isotope profile is that of the natural isotope distribution, and a direct comparison can be made between the theoretical prediction and experimental observation. For this test, isotope profiles of peptides with R^2^ larger than 0.75 were used, (15700 peptides). Supplementary Fig. 9A shows the heat map and scatter plot of the experimentally (from complete isotope profiles) and theoretically (from the natural isotope distributions of atoms) computed monoisotopic RIAs. The ideal distribution would be the identity line (shown in red). Supplementary Fig. 9B–D show the heat map and scatter plots of monoisotopic RIAs obtained by using A_1_(0)/A_0_(0), A_2_(0)/A_1_(0), and A_2_(0)/A_0_(0) ratios versus the theoretical monoisotopic RIAs. As is seen from the figures and the correlation analyses, the best matches to the theoretical RIAs are obtained by using A_1_(0)/A_0_(0). Supplementary Fig. 10A shows the result obtained from choosing the best fit from any of the ratios, and Supplementary Fig. 10B shows the best fit from either a complete isotope profile or any of the ratios. The mean and standard deviation of the relative differences of RIAs from complete isotope profiles and theoretical calculations (using atomic isotope distributions) were 0.0427 and 0.093, respectively, Supplementary Fig. 9A. After combining the data from the complete mass isotopomer profiles and the ratios of abundances of mass isotopomers, the mean and standard deviation of the relative differences (from the theoretical values) were reduced to 0.0097 and 0.037, respectively, Supplementary Fig. 10B. Pearson correlation between the RIAs computed from only complete isotope profiles was 0.95. It increased to 0.99 when the ratios and complete isotope profiles were used. The greatest number (34%) of RIA estimations came from the A_1_(0)/A_0_(0) ratio, Supplementary Fig. 11. The other ratios and complete isotope profile estimations contributed approximately equally. In the d2ome+ workflow section of Supplementary Information, we provide additional data and analyses of the performances of complete and partial isotope approaches for the unlabeled sample. In particular, we provide the density plots of the relative errors of RIA estimations from all methods (Supplementary Fig. 12), the complementarity of the various isotope ratios (Supplementary Fig. 13), and improvements for the peptides whose monoisotopic RIAs are under or over-estimated by complete isotope profiles and were improved by partial isotope profiles (Supplementary Table 1). The description also contains the substantiation for using six mass isotopomers in the protein turnover study based on the deuterium labeling, Supplementary Fig. 14. In Supplementary Data 2, we provide the results of the estimations of the monoisotopic RIAs from each of the methods for all peptides in an unlabeled sample. The proportions of erroneously estimated RIAs were higher for the low abundance peptides, Supplementary Table 1. The relative error of the RIA estimation was higher at the start and end of the chromatographic peak elution, which is shown in the box plot, Supplementary Fig. 15. The analyses provide the characteristics of the monoisotopic RIA estimation from complete and partial isotope profiles with mass accuracy (Supplementary Table 2), chromatographic elution window (Supplementary Table 3), and sample fractionation (Supplementary Table 4).

### The approach using two mass isotopomers doubles the number of high-quality model fits

**Fig. 3:**
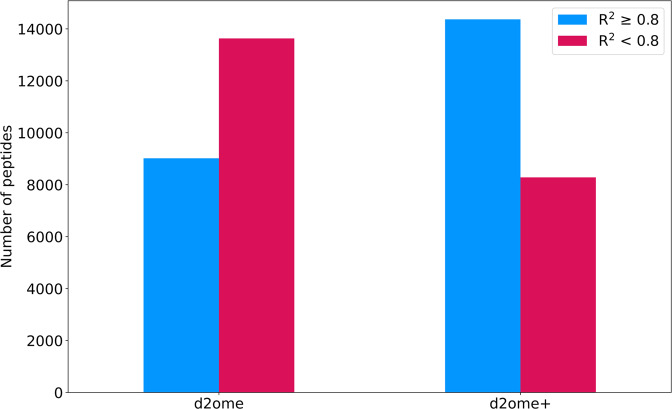
The number of peptides useful for protein turnover quantification increases by 60% after processing with d2ome + (two mass isotopomer approach). The blue bars show the number of peptides for which the coefficient of determination (R^2^) of the theoretical fit to the experimental data was equal to or larger than 0.8. The red bars show the number of peptides for which R^2^ is less than 0.8. From the estimations using complete isotope profiles (original d2ome), theoretical fits for 10644 distinct peptides had R^2^ ≥ 0.8. The use of partial isotope profiles (d2ome+) increased the corresponding number to 16708. The number of distinct peptides with R^2^ < 0.8 was reduced from 14610 to 8546.

**Fig. 4:**
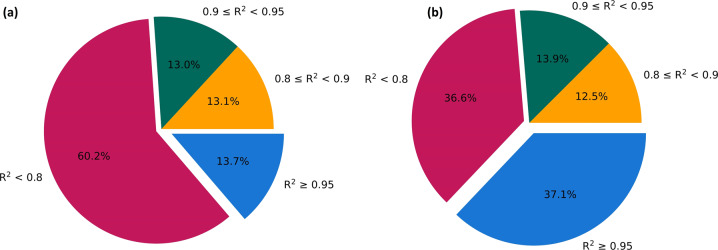
The number of peptides for which the coefficient of determination (R2) is 0.95 or higher has more than doubled when using d2ome + (two mass isotopomer approach). Shown are the pie charts of detailed percentages of the results turnover rates from d2ome (**a**) and d2ome + (**b**) approaches. The percentages of quantified peptides with respect to four different R^2^ intervals are shown. The percentage of peptides for which R^2^ ≥ 0.95 (blue color) has increased from 16 to 40%. This group has become the largest category of peptides after the theoretical fit using the monoisotopic RIAs obtained from the ratios of two mass isotopomers. The percentages of peptides with R^2^ < 0.8 (red color) decreased by 24%. The number of peptides in the other two categories remained unchanged.

**Fig. 5:**
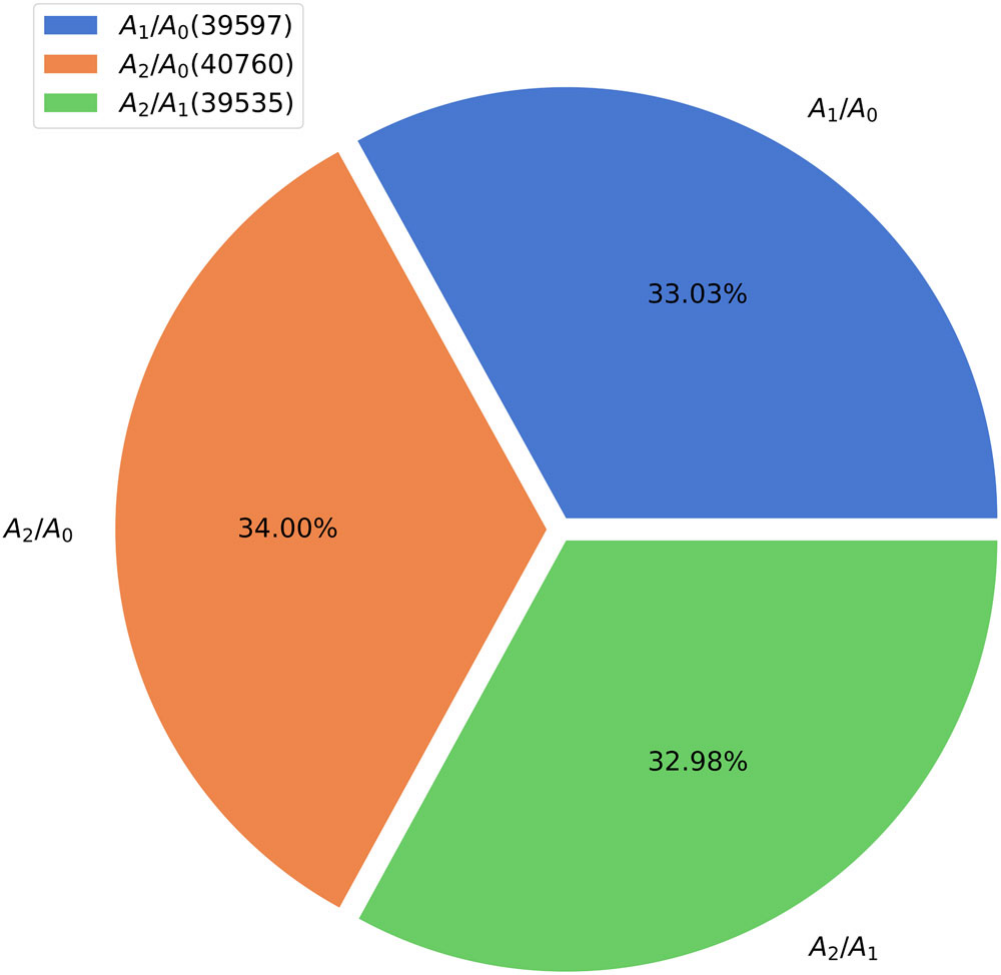
Each of the three ratios contributed about equally to the improved estimations of the monoisotopic RIA. The pie chart shows the percentages of data points for which the estimations of labeling enrichment from each ratio type were optimal. For 34% (~61000 data points) of improved estimations of the label enrichments, the improvements were achieved by using A_2_(t)/A_0_(t) ratio.

The Orbitrap Eclipse dataset was used to determine the performance of the approach using only two mass isotopomers, identify mass spectral characteristics of peptides for which the quantification was improved, and analyze the accuracy of the rate constant estimations for these peptides. The number of quantified peptides that passed the R^2^ threshold of 0.8 before and after the use of two mass isotopomer ratios are shown in Fig. 3. The time course of peptides that were quantified in at least four labeling durations were considered^36,39^. In the dataset, there were 22468 such peptides in total. d2ome+ increased the number of peptides with the improved theoretical fit (R^2^ ≥ 0.8) to the experimental data by 60%. The exact numbers of peptides are shown in Supplementary Table 5. To detail the improvements by the ranges of the R^2^ values, the latter were divided into four groups (R^2^ < 0.8; 0.8 ≤ R^2^ < 0.9; 0.9 ≤ R^2^ < 0.95; R^2^ ≥ 0.95). It is important to detail the improvements, as R^2^ ≥ 0.8 may mean 0.8 ≤ R^2^ < 0.85 or R^2^ ≥ 0.95. The latter interval indicates the most accurate fit to the experimental data. These results are shown in Fig. 4a (original d2ome) and B (d2ome + ). The improvement was in the category of peptides for which the R^2^ ≥ 0.95, where the number of peptides more than doubled, Supplementary Table 5. The improvements were achieved by each one of the ratios. It is shown in Fig. 5 which depicts proportions that each specific ratio (out of the three) had contributed to the improvements in the R^2^ coefficients. This finding indicates that all three mass isotopomers were important for improvement of the isotope enrichment estimations.

The results were analyzed to find out for which type of mass spectral data the two mass isotopomer approach improves the GOF of the exponential decay model. The focus was on the spectral accuracy (SA) of the complete isotope profiles and its effect on the R^2^. For this, at first, the distributions of SA of the data for which the R^2^ was high (R^2^ ≥ 0.95, 3106 peptides) and low (R^2^ ≤ 0.25, 7372 peptides) were computed. Since the isotope profiles of peptides change during the labeling, theoretical natural isotope distributions are not sufficient for estimating the SA. It was assumed that at the time point of labeling, t, the mass isotopomer distribution of a peptide is a composite spectrum, ${{{{{\rm{S}}}}}}({{{{{\rm{t}}}}}})$, of unlabeled (with the proportion θ), ${{{{{{\rm{S}}}}}}}_{{{{{{\rm{unlabeled}}}}}}}$, and labeled (with the (1– θ) proportion), ${{{{{{\rm{S}}}}}}}_{{{{{{\rm{labeled}}}}}}}$, forms of the peptide^42^:

4 $${{{{{\rm{S}}}}}}({{{{{\rm{t}}}}}})={{{{{{\rm{\theta }}}}}}\,{{{{{\rm{S}}}}}}}_{{{{{{\rm{unlabeled}}}}}}}+\left(1-{{{{{\rm{\theta }}}}}}\right){{{{{{\rm{S}}}}}}}_{{{{{{\rm{labeled}}}}}}}$$

where ${{{{{{\rm{S}}}}}}}_{{{{{{\rm{unlabeled}}}}}}}$ is the isotope profile of the unlabeled (natural) form of a peptide, and ${{{{{{\rm{S}}}}}}}_{{{{{{\rm{labeled}}}}}}}$ is the isotope profile of the fully labeled form of the peptide. ${{{{{{\rm{S}}}}}}}_{{{{{{\rm{labeled}}}}}}}$ is determined from the body water enrichment (p_W_) and the number of exchangeable hydrogens (N_EH_), using the formulas for mass isotopomers abundances (Eqs. (5)–(9) in the “Methods” section). θ can be between 0 to 1; zero corresponds to the unlabeled peptide, one is the fully labeled peptide. This is an often-used form of representation of a composite spectrum^43^, but here isotope profile of the labeled peptide is computed from a formula instead of the simulation, e.g., by Fourier transforms. θ is obtained from the minimization of the sum of squares of differences between computed, ${{{{{\rm{S}}}}}}({{{{{\rm{t}}}}}})$, and experimental, ${{{{{{\rm{S}}}}}}}^{{{{{{\rm{expr}}}}}}}({{{{{\rm{t}}}}}})$, isotope profiles:

$${{{{{\rm{\theta }}}}}}={\arg }\mathop{{{\min }}}\limits_{{{{{{\rm{\theta }}}}}}}{\left({{{{{{\rm{S}}}}}}}^{{{{{{\rm{expr}}}}}}}({{{{{\rm{t}}}}}})-{{{{{\rm{S}}}}}}({{{{{\rm{t}}}}}})\right)}^{2}={\arg }\mathop{{{\min }}}\limits_{{{{{{\rm{\theta }}}}}}}\mathop{\sum }\limits_{k=0}^{5}{\left({{{{{{\rm{S}}}}}}({{{{{\rm{t}}}}}})}_{{{{{{\rm{k}}}}}}}^{{{{{{\rm{expr}}}}}}}-{{{{{{\rm{S}}}}}}({{{{{\rm{t}}}}}})}_{k}\right)}^{2}{subject}\,{to}:{{{{{\rm{\theta }}}}}}\in \left[0{{{{{\rm{;}}}}}}1\right]$$

The minimum sum of the squares of the differences (SSD) between the theoretical profile from Eq. (4) and the experimental mass isotopomer profile of peptides characterizes the SA of the experimental mass isotopomer at the labeling time point t. The SSDs were calculated for two groups of peptides with R^2^ ≥ 0.95 and R^2^ ≤ 0.25. Supplementary Fig. 16 shows the density plots of the natural log-transformed values of SSDs for each group. The densities of the SSDs for these two groups were well separated. It shows that high GOF characteristics are obtained from isotope profiles that have better SAs. Our approach corrects the estimations of the label incorporation for peptides with moderate-to-low GOF characteristics.

### Accuracy of turnover rates obtained from the ratios

Since the real turnover rates of proteins are unknown, it is not possible to directly estimate the accuracy of the turnover rates. However, one can use the turnover rates of proteins quantified by multiple distinct peptides, as a basis for the comparison. In general, the estimations of turnover rates of proteins quantified by many peptides are robust. For this analysis, we used only proteins that had at least six distinct quantified peptides that passed the R^2^ ≥ 0.8 threshold (using complete isotope profiles). Turnover rates, whose GOF characteristics were improved with the computation using the ratios, were compared with the distribution of the rates obtained from the complete isotope profiles. For the comparison metric, a normalized difference between the protein turnover rate, k_prot_ (the median of turnover rates of all peptides of the protein), and the peptide turnover rate, k_pep_, was used. The metric was $({k}_{{pep}}-{k}_{{prot}})/\scriptstyle\sqrt{{{k}_{{pep}}}^{2}+{{k}_{{prot}}}^{2}}$. The histograms of the metric before and after using the ratios are shown in Supplementary Fig. 17. The addition of the peptides improved the mean and median, while the standard deviation of the distribution of the metric slightly increased. Thus, the median and mean of the test metric in the updated distribution were 0.02 and 0.04, respectively. In the original distribution, the corresponding values were 0.04 and 0.06. The standard deviation of the distribution before and after the update was 0.16 and 0.2. The numbers of distinct peptides that passed the R^2^ threshold before and after the use of the ratios for label enrichment were 7210 and 10543, respectively. The results show that using the ratios in peptide turnover rate calculations increased the number of distinct peptides and did not affect the accuracy of the turnover rate estimations for proteins.

### Murine liver protein turnover

In the Orbitrap Eclipse dataset, there were 2392 proteins, which had at least one peptide quantified in four or more experiments. As summarized in Supplementary Table 5, for 1769 of the proteins, there were at least one peptide with R^2^ ≥ 0.8 from time courses computed with complete isotope profiles. The number increased to 2108 for the approach using partial isotope profiles. The number of proteins with at least one peptide with R^2^ ≥ 0.95 increased by 80% when the monoisotopic RIA was computed from partial isotope profiles.

The global turnover rate of murine proteins has been reported to be 2–3 days^8^, which corresponds to turnover rates 0.23–0.35 day^−1^. The median and mean of the turnover rates in the dataset were 0.28 day^−1^ and 0.22 day^−1^, respectively. The density plot of the turnover rate distribution is shown in Supplementary Fig. 18. Proteins such as Major Urinary Protein and APOE turnover very fast^44^—by the time the data was collected for the first labeling time point, 1 day. For these proteins, it can accurately be stated that their turnover rates are faster than 0.69 day^−1^. The rates of all quantified proteins are reported in Supplementary Data 3. The tables also include the CIs for protein turnover rates which are computed from standard deviations of the rate constants of their constituent peptides (Supplementary Eq. (12)).

### Long-lived proteins

**Fig. 6:**
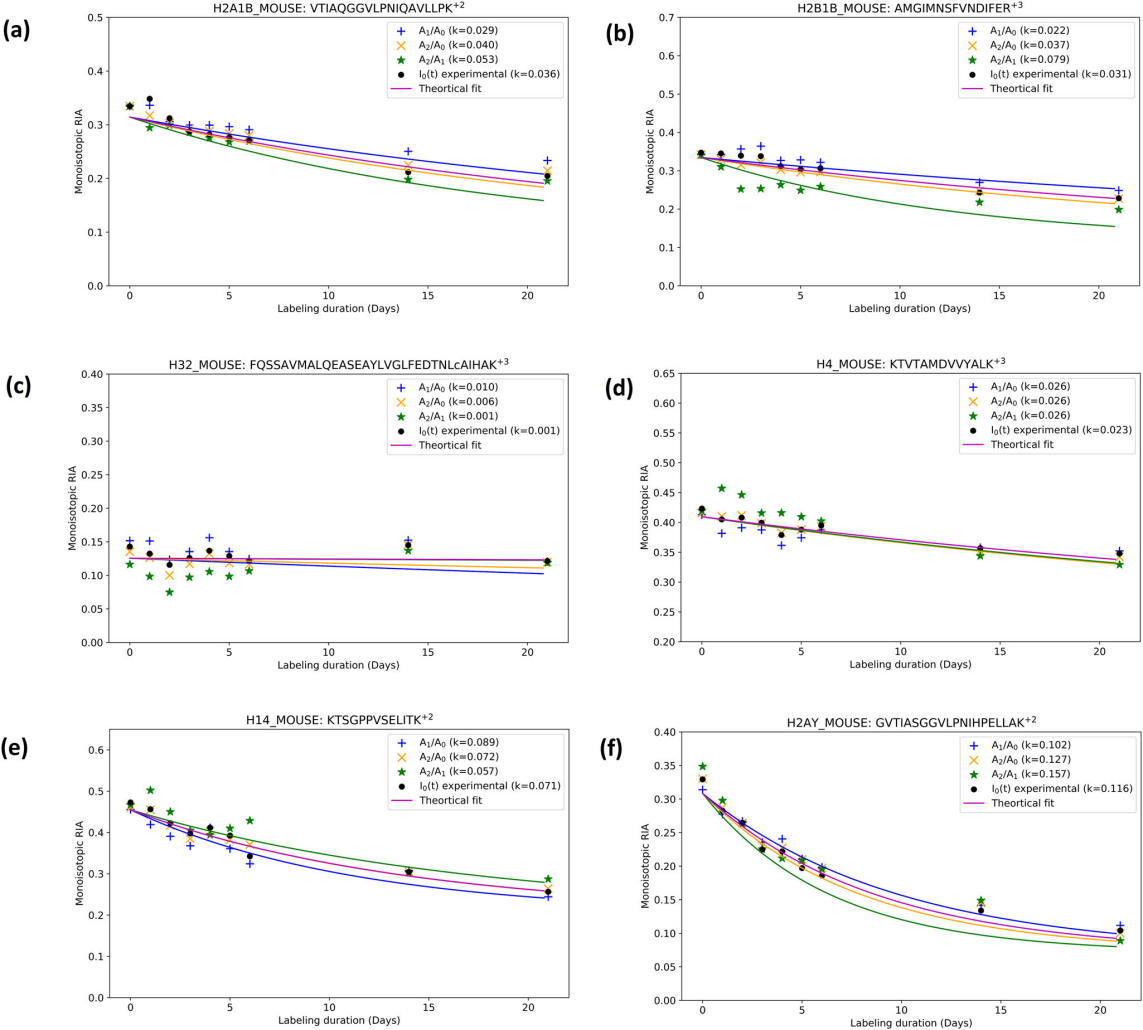
The turnover rates of histones are accurately computed by the approach using two mass isotopomers. Slow turnover rates of Histones are reflected in the time series of label incorporation obtained using complete (experimental) isotope profiles and reconstructed isotope profiles (using a pair of mass isotopomers). Shown are the labeling time series data of a peptide of histones: (**a**) H2A1B (histone 2A type 1-B), (**b**) H2B1B (histone 2B type 1-B), (**c**) H3.2 (histone H3.2), (**d**) H4 (histone H4), (**e**) H1.4 (histone H1.4), and (**f**) H2AY (core histone macro-H2A.1).

There existed a group of murine liver proteins with computed half-lives longer than 21 days (turnover rate smaller than 0.033 day^−1^). In general, long-lived proteins have received prominent attention in protein turnover studies^45,46^. Intracellular proteins with long lifespans have been linked to age-dependent defects, which include decreased fertility and functional decline in neurons^45^. We analyzed the list of proteins with half-lives longer than 21 days using the STRING^47^ database. The list included histones, lamins, collagens, nuclear pore proteins, hemoglobins, cytoskeletal keratins, Band 3 anion transport protein (major integral membrane glycoprotein of the erythrocyte membrane), and carbonic anhydrases. The STRING^47^ analysis generated the protein network shown in Supplementary Fig. 19. There are a few subnetworks that are made of interacting histones, cytoskeletal keratins, collagens, carbonic anhydrases, and hemoglobins. Figure 6 shows the representative time series data and the corresponding theoretical fits for different Histone proteins. Members (H2A1B_MOUSE, H2B1B_MOUSE, H31_MOUSE, H4_MOUSE) of the four histone families (H2A, H2B, H3, H4) that are the core components of nucleosome had slower turnover rates ranging from 0.024 day^−1^ to 0.043 day^−1^, Fig. 6A–D. Histones of the H1 family bind to the linker DNA between the nucleosomes. The H14_MOUSE variant had a faster turnover rate, 0.0715 day^−1^, Fig. 6E. Time series data from peptides of these and other slow turnover proteins obtained from time series of 96 distinct peptide sequences are shown in Supplementary Data 4. The approach using partial isotope profiles increased the number of distinct peptides that passed the GOF threshold by 60%.

One group of proteins provides a positive control for zero turnover rate computations. The contaminants, e.g., trypsin and various human keratins, are introduced into proteomic samples at each sample preparation^48^. They are not labeled with heavy water; their “turnover rate” should be zero. Supplementary Data 5 contains labeling time series of 123 distinct peptides from the contaminant proteins. These peptide sequences are not shared with any of the murine protein sequences in the SwissProt database. As seen from the figures, the computed turnover rates were practically zero. It is noted that for very slow turnover proteins (turnover rate less 0.01 day^−1^), the R^2^ is not an appropriate measure of the GOF^37^. For these proteins, the standard error of the theoretical fit (≤0.05) was used as a GOF measure.

### Turnover of proteins in complexes

**Fig. 7:**
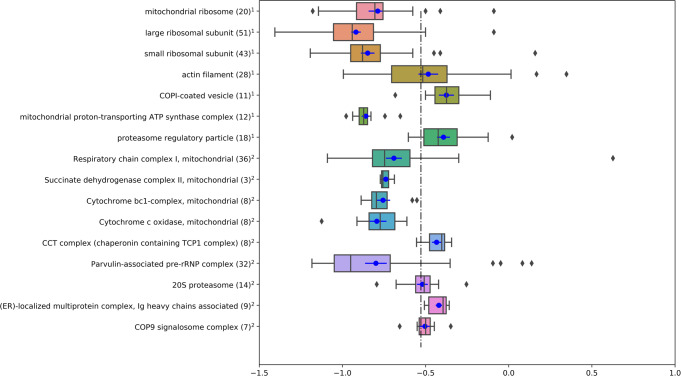
Proteins of a complex have similar turnover rates. Turnover rates of proteins in protein complexes obtained from Gene Ontology and CORUM databases. x-axis is the base-10 logarithm of the protein turnover rates. Shown are the boxplots of turnover rates of proteins for each complex. Each box comprises turnover rates between the 25th and 75th percentiles of the complex proteins. The vertical bar in each box is the median turnover rate of the proteins in the corresponding complex. The blue dot in each box is the mean turnover rate of the proteins in the complex. The horizontal blue line indicates the standard error of the mean. The dashed vertical line is the median protein turnover rate in the liver sample. COPI coatomer complex, ATP adenosine triphosphate, ER endoplasmatic reticulum, rRNP ribosomal ribonucleoprotein, COP9 Constitutive photomorphogenesis 9. 1—complexes obtained from the Gene Ontology database (cellular component). 2—complexes obtained from the CORUM database.

In general, proteins that form a physical complex are expected to have similar turnover rate. CORUM^49^ and Gene Ontology (GO)^50^ databases were used to determine the protein enrichments of complexes. A GO complex was included when the complex was not defined in CORUM. Figure 7 shows the boxplots of the ten-based logarithms of protein turnover rates in each complex. The number of quantified proteins in each complex is shown inside the parentheses. The vertical dashed line is the median of protein turnover rates (0.28 day^−1^). It corresponds to the median half-life of 2.5 days. The median turnover rate of each one of the mitochondrial respiratory chain complexes was smaller than the liver median. The observation agrees with mouse brain protein turnover, where the mitochondrial respiratory chain proteins were reported^7^ to turn over slower than the median protein turnover. The ribosomal proteins, too, had slower turnover rates than the median. This is different from the reported result in mouse brain^7^, where the median turnover rate of the ribosomal proteins was similar to that of all proteins. The Wilcoxon rank sum test was used to identify statistically significant differences in turnover rates of the complexes. The *p* values were adjusted for the multiple hypothesis testing. The proteins of the mitochondrial ribosome had statistically significant turnover rates from those of the large ribosomal subunit. The differences in turnover rates of the proteins of respiratory chain complex V (mitochondrial proton transporting ATP synthase) from those of the respiratory chain complexes I and III were statistically significant. While the median turnover rate of the complex V proteins was smaller than those of the complexes II and IV, the results were not statistically significant. The sample size in complex II was too small (three proteins out of four listed in the CORUM database). Therefore, the p value of the difference is not significant. In mouse brain, proteins in the complexes III and V had been reported^7^ to have similar behavior of the median turnover rates. Their turnover rates were slower than those of proteins in the other complexes of the mitochondrial respirator chain.

The FDR of the Wilcoxon rank sum test (*p*_value was equal to 0.04) for the comparison of turnover rates of the regulatory particle and core (20 S proteasome) complex of the proteasome was 0.07. The median turnover rate of the core complex proteins was smaller than that of the regulatory particle proteins. This result agrees with the corresponding result for turnover rates from several cell types^51^. The regulatory particle consists of the lid and base subcomplexes. It recognizes proteins destined for degradation by the proteasome. It is turned over faster than the core complex proteins (includes proteins for the catalytic subunit for the proteolysis). All Wilcoxon rank sum test results are provided in the Supplementary Data 6.

### Validations of the approach on datasets from four murine tissue types acquired on Orbitrap Q Exactive HF mass spectrometer

Since the Orbitrap Eclipse dataset was used for development and testing, we also used other datasets to confirm that d2ome+ increased the proteome coverage for protein turnover rate estimations. A recent study^36^ provided heavy water-labeled LC-MS dataset for analyses of protein turnover in four tissues of the C57/BL6J mouse strain. The data was acquired on Orbitrap Q Exactive HF. In addition to the liver, the study included kidney, heart, and muscle tissues. The latter two have slower protein turnover and differ in that regard from liver tissue analyzed in this study. We have processed the LC-MS data from all four tissue types for improvements in the GOF characteristic of the theoretical fit to the experimental time course data. Protein turnover rates are reported in Supplementary Data 3. The changes in the numbers of peptides that passed the R^2^ threshold for each tissue type are shown in Supplementary Fig. 20A–D. It is noteworthy that the percent of the peptides for which R^2^ ≥ 0.8 using the complete isotope profiles obtained in the Orbitrap Q Exactive HF mass analyzer was at least 50%, which was higher than that in the data acquired on Orbitrap Eclipse. For these data, the percent of high (R^2^ ≥ 0.8) GOF peptides ranged from 51% (muscle) to 62% (liver). Nonetheless, d2ome+ improved the quantification results in all four datasets. In each dataset, it has increased the number of peptides with R^2^ ≥ 0.8 by at least 30%. The peptide turnover rates for each tissue type obtained using heavy water and amino acid labeling matched closely. The correlation coefficients ranged from 0.96 (muscle proteome) to 0.87 (liver proteome). The scatter plots of the peptide turnover rates are shown in Supplementary Fig. 21A–D. All peptides contained at least one Lys amino acid, as the original study^36^ compared the turnover rates obtained from labeling with Lys with those using heavy water. In summary, the estimation of label enrichment from abundances of a pair of mass isotopomers improved the proteome coverage of turnover rate estimations from Orbitrap Q Exactive mass spectrometer for all four tissue types (with varying turnover rates). The result indicates that the approach taken in this work generalizes to slow (heart, muscle) and fast (liver, kidney) protein turnover tissues.

## Discussion

This work reports on the development of an approach to compute protein turnover from partial isotope profiles. It is based on the estimation of label enrichment from raw abundances of only two mass isotopomers. The enrichment was used to re-construct the corresponding monoisotopic RIA. The newly generated RIAs were used in the time series data to fit the exponential decay model to extract the turnover rate. The developments were necessitated by the observations^37,38^ that the traditional approach, which estimates the monoisotopic RIA from the complete isotope profiles, results in many quantified peptides that are not used for protein turnover rate estimations. Their GOF characteristics to experimental time series from these peptides were low, R^2^ < 0.8.

The ratios from three pairs of mass isotopomers were examined in the study, A_1_(t)/A_0_(t), A_2_(t)/A_0_(t), and A_2_(t)/A_1_(t). The best fit from all pairs of the ratios resulted in a 60% increase in the number of peptides that passed the R^2^ ≥ 0.8 thresholds. The number of peptides for which R^2^ ≥ 0.9 more than doubled. The examination of the contribution from each one of the ratios revealed the improvements resulted from all ratios with nearly equally contributions. It has been previously reported^52,53^ that contaminant peptides affect the quantification in protein turnover studies that included SILAC^54^ labeling. For example, a “prior ion ratio” was introduced as a metric for co-elution with the target peptide^52^. The prior ion was defined as an ion whose mass is one neutron less than that of the target peptide’s monoisotopic mass. The prior ion ratio was defined as the ratio of the abundance of the prior ion to the sum of the abundances in the isotopic cluster of the target. The ratio served as an indicator for contamination of the target peptides’ isotope cluster. In our approach, this (prior ion) co-elution will mainly describe the interferences with the monoisotope. In this case, the label enrichment estimation and follow-up quantification will be done using the A_2_/A_1_ ratio.

The spectral data, which improved the estimation of label enrichment from a pair of mass isotopomers, were analyzed to identify their common properties. The spectral accuracy of these peptides was poor. They are contrasted with the corresponding data for peptides with high R^2^, which showed good spectral accuracy. It was concluded that the approach for estimating label enrichments from a pair of mass isotopomers improves the labeling time series data for peptides whose mass profiles had inferences from contaminants. Since the complete isotope profile of a peptide requires measurements of up to six mass isotopomers, the co-elutions are likely in complex mammalian samples.

The approach was applied to other datasets from four tissue types with varying turnover rates. In all cases, the number of quantified peptides was increased, and the number of high-quality peptides was doubled. It shows that the method generalized well to slow and fast turnover proteins.

Previous studies have examined approaches for using partial isotope profiles to estimate label incorporation^43,55,56^. Thus, determination^43^ of the label enrichment from the first two mass isotopomers had been used for samples labeled with ^12^C. An approximation to Supplementary Eq. (2) was used to estimate the protein turnover in heavy water labeling from a single labeled sample and two mass isotopomers^55^. An approximate expression for the A_2_(t)/A_0_(t) ratio and a “fudge” factor for it were used in another study^56^. Another use of the ratios was to estimate the label enrichment from a regression model^32^. Regression coefficients of several ratios were determined from theoretical simulations of isotope profiles using known label enrichments. The regression coefficients then were used to determine the label enrichment from experimental isotope profile. From the accurate formulas, a single pair of mass isotopomers is enough to estimate the label enrichment. In addition, the applications in this work showed (Fig. 5 and Supplementary Figs. 9–13) that the estimations separately from each pair of mass isotopomers are necessary to improve the quality of the label quantification. The ratios are chosen from the first three mass isotopomers as they are often the most abundant for peptides. To the best of our knowledge, this work is the first to systematically implement the use of partial isotope profiles for computing the monoisotopic RIA in stable isotope labeling.

We compared the murine liver protein turnover rates from heavy water labeling (Orbitrap Eclipse)^14^ and heavy amino-acid, ^13^C_6_-Lys, labeling^36^. In both studies, the C57/BL6J mouse strain was used. In heavy amino acid-based labeling, protein turnover is calculated from peptides that contain at least one labelable amino acid. The scatter plot and heat map of protein turnover rates from all proteins are shown in Supplementary Fig. 22A. There were 984 proteins common to both datasets in the turnover rate range [0.01, 1.0] day^−1^. The turnover range was restricted based on the shortest and the longest durations of labeling. The Pearson correlation between the rates was 0.71. For proteins quantified by multiple peptides, the turnover rate estimations are expected to be accurate. When we filtered the proteins to require at least three unique peptides, the correlation increased to 0.85, and the number of common proteins reduced to 340. The corresponding scatter plot and heat map showed a structure in the data, Supplementary Fig. 22B. It was observed that for proteins whose turnover rate falls into the [0.01, 0.3] day^−1^ range, the differences between the turnover rates from the two labeling approaches were mainly smaller than 20%. In this range, there were 214 proteins, and for 164 of them, the turnover rates computed by the two labeling approaches differed by less than 20%. For the proteins in this turnover range, the largest relative deviation of rates between the two methods was 70%. We conclude that for abundant proteins in the [0.01, 0.3] day^−1^ range, both labeling methods produced similar and reproducible results. It is noted that the structure in the heat map was noticeable in the unfiltered (by the number of quantified peptides) data as well, Supplementary Fig. 22A.

In summary, we developed an approach to estimating protein turnover from two mass isotopomers. The approach improved the GOF characteristics of the protein turnover model in various tissue types with slow and fast turnover proteins. The implemented tool, d2ome + , is publicly available. It increases the proteome coverage in protein turnover studies using heavy water metabolic labeling and LC-MS. We expect that it will be useful to a broader community as a data processing tool and promote the applications of the heavy water labeling platform for protein turnover studies.

Here the approach was applied to a deterministic and one-compartment model of the depletion of monoisotopic RIA. There are more complex (such as stochastic and two-compartment) models for the time course of depletion and turnover rate^57,58^. The stochastic model accounts for the correlations in the time course data, and the two-compartment model incorporates label enrichment kinetics of amino acids. The suggested approach to estimating the label enrichment from a ratio of two mass isotopomers reconstructs the monoisotopic RIA. The reconstructed RIA can be used in complex models for protein turnover rate computation.

## Methods

Animal experiments, sample preparation, LC-MS experiments, protein identification, and d2ome+ workflow are described in the correspondingly named sections of Supplementary Information.

The time course of the monoisotopic RIA was given in Eq. (3) in the main text. The normalized abundances of the first three heavy mass isotopomers were previously derived^59^:

5 $${{{{{{\rm{I}}}}}}}_{1}\left({{{{{\rm{t}}}}}}\right)=\frac{\left(1-{{{{{{\rm{p}}}}}}}_{{{{{{\rm{H}}}}}}}-{{{{{{\rm{p}}}}}}}_{{{{{{\rm{X}}}}}}}({{{{{\rm{t}}}}}})\right){{{{{{\rm{p}}}}}}}_{{{{{{\rm{X}}}}}}}({{{{{\rm{t}}}}}})}{\left(1-{{{{{{\rm{p}}}}}}}_{{{{{{\rm{H}}}}}}}\right)}{{{{{{\rm{N}}}}}}}_{{{{{{\rm{EH}}}}}}}{{{{{{\rm{I}}}}}}}_{0}\left({{{{{\rm{t}}}}}}\right)+{\left(1-\frac{{{{{{{\rm{p}}}}}}}_{{{{{{\rm{X}}}}}}}({{{{{\rm{t}}}}}})}{1-{{{{{{\rm{p}}}}}}}_{{{{{{\rm{H}}}}}}}}\right)}^{{{{{{{\rm{N}}}}}}}_{{{{{{\rm{EH}}}}}}}}{{{{{{\rm{I}}}}}}}_{1}\left(0\right)$$

6 $${{{{{{\rm{I}}}}}}}_{2}\left({{{{{\rm{t}}}}}}\right)={{{{{{\rm{I}}}}}}}_{0}\left({{{{{\rm{t}}}}}}\right)\left\{\frac{{{{{{{\rm{I}}}}}}}_{2}\left(0\right)}{{{{{{{\rm{I}}}}}}}_{0}\left(0\right)}-\frac{{{{{{{\rm{I}}}}}}}_{1}\left(0\right)}{{{{{{{\rm{I}}}}}}}_{0}\left(0\right)}\frac{{{{{{{\rm{p}}}}}}}_{{{{{{\rm{H}}}}}}}{{{{{{\rm{N}}}}}}}_{{{{{{\rm{EH}}}}}}}}{\left(1-{{{{{{\rm{p}}}}}}}_{{{{{{\rm{H}}}}}}}\right)}+{{{{{{\rm{b}}}}}}}_{2}\left(0\right)-{{{{{{\rm{b}}}}}}}_{2}\left({{{{{\rm{t}}}}}}\right)\right\}+{{{{{{\rm{b}}}}}}}_{1}\left({{{{{\rm{t}}}}}}\right){{{{{{\rm{I}}}}}}}_{1}\left({{{{{\rm{t}}}}}}\right)$$

In Eq. (7), c_n_ denotes the following coefficient:

$${{{{{{\rm{c}}}}}}}_{{{{{{\rm{n}}}}}}}=\left(\begin{array}{c}{{{{{{\rm{N}}}}}}}_{{{{{{\rm{EH}}}}}}}+{{{{{\rm{n}}}}}}-1\\ {{{{{\rm{n}}}}}}\end{array}\right){\left(\frac{{{{{{{\rm{p}}}}}}}_{{{{{{\rm{H}}}}}}}}{1-{{{{{{\rm{p}}}}}}}_{{{{{{\rm{H}}}}}}}}\right)}^{{{{{{\rm{n}}}}}}}$$

b_n_(t) was defined in the main text.

For this work, we derived equations for the heavy mass isotopomers I_4_(t) and I_5_(t), and finalized the derivation of I_3_(t), which made it independent of the number of all hydrogens unlike the previous formula^59^. The heavy mass isotopomers are important because their relative abundance significantly increase even for small peptides (<1100 Da mass) after labeling with 5% enriched heavy water, Supplementary Fig. 14.

7 $${{{{{{\rm{I}}}}}}}_{3}({{{{{\rm{t}}}}}})= 	\,{{{{{{\rm{b}}}}}}}_{3}({{{{{\rm{t}}}}}}){{{{{{\rm{I}}}}}}}_{0}({{{{{\rm{t}}}}}})+{{{{{{\rm{b}}}}}}}_{2}({{{{{\rm{t}}}}}})({{{{{{\rm{I}}}}}}}_{1}({{{{{\rm{t}}}}}})-{{{{{{\rm{b}}}}}}}_{1}({{{{{\rm{t}}}}}}){{{{{{\rm{I}}}}}}}_{0}({{{{{\rm{t}}}}}})) \\ 	+{{{{{{\rm{b}}}}}}}_{1}({{{{{\rm{t}}}}}})\left\{{{{{{{\rm{I}}}}}}}_{2}({{{{{\rm{t}}}}}})-{{{{{{\rm{b}}}}}}}_{1}({{{{{\rm{t}}}}}})({{{{{{\rm{I}}}}}}}_{1}({{{{{\rm{t}}}}}})-{{{{{{\rm{b}}}}}}}_{1}({{{{{\rm{t}}}}}}){{{{{{\rm{I}}}}}}}_{0}({{{{{\rm{t}}}}}}))-{{{{{{\rm{b}}}}}}}_{2}({{{{{\rm{t}}}}}}){{{{{{\rm{I}}}}}}}_{0}({{{{{\rm{t}}}}}})\right\} \\ 	+\left\{\frac{{{{{{{\rm{I}}}}}}}_{3}(0)}{{{{{{{\rm{I}}}}}}}_{0}(0)}-{{{{{{\rm{c}}}}}}}_{1}\frac{{{{{{{\rm{I}}}}}}}_{2}(0)}{{{{{{{\rm{I}}}}}}}_{0}(0)}+{{{{{{\rm{c}}}}}}}_{2}\frac{{{{{{{\rm{I}}}}}}}_{1}(0)}{{{{{{{\rm{I}}}}}}}_{0}(0)}-{{{{{{\rm{c}}}}}}}_{3}\right\}{{{{{{\rm{I}}}}}}}_{0}({{{{{\rm{t}}}}}})$$

8 $${{{{{{\rm{I}}}}}}}_{4}({{{{{\rm{t}}}}}})= 	\,{{{{{{\rm{b}}}}}}}_{4}({{{{{\rm{t}}}}}}){{{{{{\rm{I}}}}}}}_{0}({{{{{\rm{t}}}}}})+{{{{{{\rm{b}}}}}}}_{3}({{{{{\rm{t}}}}}})\{{{{{{{\rm{I}}}}}}}_{1}({{{{{\rm{t}}}}}})-{{{{{{\rm{b}}}}}}}_{1}({{{{{\rm{t}}}}}}){{{{{{\rm{I}}}}}}}_{0}({{{{{\rm{t}}}}}})\}\\ 	+{{{{{{\rm{b}}}}}}}_{2}({{{{{\rm{t}}}}}})\{{{{{{{\rm{I}}}}}}}_{2}({{{{{\rm{t}}}}}})-{{{{{{\rm{b}}}}}}}_{1}({{{{{\rm{t}}}}}})({{{{{{\rm{I}}}}}}}_{1}({{{{{\rm{t}}}}}})-{{{{{{\rm{b}}}}}}}_{1}({{{{{\rm{t}}}}}}){{{{{{\rm{I}}}}}}}_{0}({{{{{\rm{t}}}}}}))-{{{{{{\rm{b}}}}}}}_{2}({{{{{\rm{t}}}}}}){{{{{{\rm{I}}}}}}}_{0}({{{{{\rm{t}}}}}})\} +{{{{{{\rm{b}}}}}}}_{1}({{{{{\rm{t}}}}}})\{{{{{{{\rm{I}}}}}}}_{3}({{{{{\rm{t}}}}}})\\ 	-{{{{{{\rm{b}}}}}}}_{1}({{{{{\rm{t}}}}}})[{{{{{{\rm{I}}}}}}}_{2}({{{{{\rm{t}}}}}})-{{{{{{\rm{b}}}}}}}_{1}({{{{{\rm{t}}}}}})({{{{{{\rm{I}}}}}}}_{1}({{{{{\rm{t}}}}}})-{{{{{{\rm{b}}}}}}}_{1}({{{{{\rm{t}}}}}}){{{{{{\rm{I}}}}}}}_{0}({{{{{\rm{t}}}}}}))-{{{{{{\rm{b}}}}}}}_{2}({{{{{\rm{t}}}}}}){{{{{{\rm{I}}}}}}}_{0}({{{{{\rm{t}}}}}})]-{{{{{{\rm{b}}}}}}}_{2}({{{{{\rm{t}}}}}})[{{{{{{\rm{I}}}}}}}_{1}({{{{{\rm{t}}}}}}) \\ 	-{{{{{{\rm{b}}}}}}}_{1}({{{{{\rm{t}}}}}}){{{{{{\rm{I}}}}}}}_{0}({{{{{\rm{t}}}}}})]-{{{{{{\rm{b}}}}}}}_{3}({{{{{\rm{t}}}}}}){{{{{{\rm{I}}}}}}}_{0}({{{{{\rm{t}}}}}})\}+\{\frac{{{{{{{\rm{I}}}}}}}_{4}(0)}{{{{{{{\rm{I}}}}}}}_{0}(0)}-{{{{{{\rm{c}}}}}}}_{1}\frac{{{{{{{\rm{I}}}}}}}_{3}(0)}{{{{{{{\rm{I}}}}}}}_{0}(0)}+{{{{{{\rm{c}}}}}}}_{2}\frac{{{{{{{\rm{I}}}}}}}_{2}(0)}{{{{{{{\rm{I}}}}}}}_{0}(0)}-{{{{{{\rm{c}}}}}}}_{3}\frac{{{{{{{\rm{I}}}}}}}_{1}(0)}{{{{{{{\rm{I}}}}}}}_{0}(0)}+{{{{{{\rm{c}}}}}}}_{4}\}{{{{{{\rm{I}}}}}}}_{0}({{{{{\rm{t}}}}}})$$

9 $${{{{{{\rm{I}}}}}}}_{5}({{{{{\rm{t}}}}}})= 	\,{{{{{{\rm{b}}}}}}}_{5}({{{{{\rm{t}}}}}}){{{{{{\rm{I}}}}}}}_{0}({{{{{\rm{t}}}}}})+({{{{{{\rm{b}}}}}}}_{4}({{{{{\rm{t}}}}}})-{{{{{{\rm{b}}}}}}}_{1}({{{{{\rm{t}}}}}}){{{{{{\rm{b}}}}}}}_{3}({{{{{\rm{t}}}}}}))\{{{{{{{\rm{I}}}}}}}_{1}({{{{{\rm{t}}}}}})-{{{{{{\rm{b}}}}}}}_{1}({{{{{\rm{t}}}}}}){{{{{{\rm{I}}}}}}}_{0}({{{{{\rm{t}}}}}})\}\\ 	 +({{{{{{\rm{b}}}}}}}_{3}({{{{{\rm{t}}}}}})-{{{{{{\rm{b}}}}}}}_{1}({{{{{\rm{t}}}}}}){{{{{{\rm{b}}}}}}}_{2}({{{{{\rm{t}}}}}}))\{{{{{{{\rm{I}}}}}}}_{2}({{{{{\rm{t}}}}}})-{{{{{{\rm{b}}}}}}}_{1}({{{{{\rm{t}}}}}})({{{{{{\rm{I}}}}}}}_{1}({{{{{\rm{t}}}}}})-{{{{{{\rm{b}}}}}}}_{1}({{{{{\rm{t}}}}}}){{{{{{\rm{I}}}}}}}_{0}({{{{{\rm{t}}}}}}))-{{{{{{\rm{b}}}}}}}_{2}({{{{{\rm{t}}}}}}){{{{{{\rm{I}}}}}}}_{0}({{{{{\rm{t}}}}}})\}\\ 	 +({{{{{{\rm{b}}}}}}}_{2}({{{{{\rm{t}}}}}})-{{{{{{\rm{b}}}}}}}_{1}^{2}({{{{{\rm{t}}}}}}))\{{{{{{{\rm{I}}}}}}}_{3}({{{{{\rm{t}}}}}})-{{{{{{\rm{b}}}}}}}_{1}({{{{{\rm{t}}}}}})[{{{{{{\rm{I}}}}}}}_{2}({{{{{\rm{t}}}}}})-{{{{{{\rm{b}}}}}}}_{1}({{{{{\rm{t}}}}}})({{{{{{\rm{I}}}}}}}_{1}({{{{{\rm{t}}}}}})-{{{{{{\rm{b}}}}}}}_{1}({{{{{\rm{t}}}}}}){{{{{{\rm{I}}}}}}}_{0}({{{{{\rm{t}}}}}}))-{{{{{{\rm{b}}}}}}}_{2}({{{{{\rm{t}}}}}}){{{{{{\rm{I}}}}}}}_{0}({{{{{\rm{t}}}}}})]\\ 	 -{{{{{{\rm{b}}}}}}}_{2}({{{{{\rm{t}}}}}})[{{{{{{\rm{I}}}}}}}_{1}({{{{{\rm{t}}}}}})-{{{{{{\rm{b}}}}}}}_{1}({{{{{\rm{t}}}}}}){{{{{{\rm{I}}}}}}}_{0}({{{{{\rm{t}}}}}})]-{{{{{{\rm{b}}}}}}}_{3}({{{{{\rm{t}}}}}}){{{{{{\rm{I}}}}}}}_{0}({{{{{\rm{t}}}}}})\}+{{{{{{\rm{b}}}}}}}_{1}({{{{{\rm{t}}}}}})({{{{{{\rm{I}}}}}}}_{4}({{{{{\rm{t}}}}}})-{{{{{{\rm{b}}}}}}}_{4}({{{{{\rm{t}}}}}}){{{{{{\rm{I}}}}}}}_{0}({{{{{\rm{t}}}}}}))\\ 	 +\{\frac{{{{{{{\rm{I}}}}}}}_{5}(0)}{{{{{{{\rm{I}}}}}}}_{0}(0)}-{{{{{{\rm{c}}}}}}}_{1}\frac{{{{{{{\rm{I}}}}}}}_{4}(0)}{{{{{{{\rm{I}}}}}}}_{0}(0)}+{{{{{{\rm{c}}}}}}}_{2}\frac{{{{{{{\rm{I}}}}}}}_{3}(0)}{{{{{{{\rm{I}}}}}}}_{0}(0)}-{{{{{{\rm{c}}}}}}}_{3}\frac{{{{{{{\rm{I}}}}}}}_{2}(0)}{{{{{{{\rm{I}}}}}}}_{0}(0)}+{{{{{{\rm{c}}}}}}}_{4}\frac{{{{{{{\rm{I}}}}}}}_{1}(0)}{{{{{{{\rm{I}}}}}}}_{0}(0)}-{{{{{{\rm{c}}}}}}}_{5}\}{{{{{{\rm{I}}}}}}}_{0}({{{{{\rm{t}}}}}})$$

It is emphasized that the formulas for the dynamics of RIA of the six mass isotopomers are applicable to any metabolic labeling enrichment resulting from atom-based stable isotope enrichments, such as ^15^N or ^13^C. The only change that is needed to make is to replace N_EH_ with the number of atoms in a peptide which are accessible to the labeling agent. For example, in ^15^N labeling, N_EH_ is replaced with the number of exchangeable Nitrogens in a peptide. In addition, the formulas are also applicable to accurately estimate deuterium enrichment in hydrogen/deuterium exchange mass spectrometry for studying protein structure^60^.

The above equations are used to obtain the ratios of raw abundances of the i^th^ and j^th^ mass isotopomers, A_i_(t)/A_j_(t), since the normalization coefficient cancels out:

$${{{{{{\rm{A}}}}}}}_{{{{{{\rm{i}}}}}}}\left({{{{{\rm{t}}}}}}\right)/{{{{{{\rm{A}}}}}}}_{{{{{{\rm{j}}}}}}}\left({{{{{\rm{t}}}}}}\right)={{{{{{\rm{I}}}}}}}_{i}{({{{{{\rm{t}}}}}})/{{{{{\rm{I}}}}}}}_{{{{{{\rm{j}}}}}}}(t)$$

The ratios are simplified and shown in the Supplementary Notes, where the outlines of the derivations for I_4_(t) and I_5_(t) are also presented.

### Reporting summary

Further information on research design is available in the Nature Portfolio Reporting Summary linked to this article.

## Supplementary information

Supplementary Information

Description of Additional Supplementary File

Supplementary Data 1

Supplementary Data 2

Supplementary Data 3

Supplementary Data 4

Supplementary Data 5

Supplementary Data 6

Reporting Summary

## Data Availability

The raw mass spectral data (Orbitrap Eclipse), Mascot search results, and d2ome+ data analysis results of the murine liver dataset generated in this work have been deposited in the MassIVE repository with the identifier MSV000090148 (http://massive.ucsd.edu). The results of the peak detection and quantification for every peptide of every protein (Protein_Name.csv) and their corresponding rate constants (Protein_Name.RateConst.csv) are available in the repository. Supplementary Data 1 shows examples of monoisotopic RIA time series that are improved by the two mass isotopomer approach. Peak detection and quantification results for all peptides from an unlabeled sample are presented in Supplementary Data 2. Turnover rates and confidence intervals of all proteins and their peptides are provided in Supplementary Data 3. Supplementary Data 4 shows the monoisotopic RIA time series and theoretical fits for slow turnover proteins. Supplementary Data 5 shows the monoisotopic RIA time series and theoretical fits for the “turnover” of contaminant proteins. Supplementary Data 6 contains Wilcoxon rank sum test results for protein complexes presented in Fig. 7.The raw mass spectral datasets from samples of murine liver, kidney, heart, and muscle tissues were downloaded from ProteomeXChanger using the identifier (PXD029639) provided in the original publication^36^. The mass spectral data in the mzML file format (from MSConvert), database search results (from Mascot), and protein turnover analysis results (from d2ome+) have been uploaded into MassIVE under the identifier shown above.

## Code availability

The code and instructions for d2ome+ software are available on GitHub, https://github.com/rgsadygov/d2ome/releases/tag/v1.05.
