# Supplementary material for: Quantifying label enrichment from two mass isotopomers increases proteome coverage for in vivo protein turnover using heavy water metabolic labeling
Source: Commun Chem. 2023 Apr 17;6:72. doi: 10.1038/s42004-023-00873-x (PMC10110577; doi:10.1038/s42004-023-00873-x)
Supplement: Supplementary file 1 — Supplementary Information [file 42004_2023_873_MOESM1_ESM.pdf]

Supplementary Information

for

Quantifying Label Enrichment from Two Mass Isotopomers Increases Proteome Coverage for *in vivo* Protein Turnover using Heavy Water Metabolic Labeling

TABLE OF CONTENTS

Supplementary Notes ……………………………………………………………..…… 4

Animal Experiments……………………………………………………………………...8

Determination of body water enrichment ………………………………………..…..9

Sample processing ……………………………………………………………..……..10

NanoLC MS/MS Analysis……………………………………..……………………..11

Protein identification………………………….……………………………………..12

d2ome+ workflow………………………………………………………………………13

Supplementary Figures

1. MS1 profile of peptide VLGTSVESIMATEDR^{+3} (CPSM_MOUSE) exhibits a co-elution (M_3-M_6) with a species of the same charge state………….. ……………..…..19
2. The peptide YLAEVAcGDDR^{+2} (1433T_MOUSE) co-elutes with a +4 charged species (M_2-M_5) …………………………………………………………………..….20

3. The peptide GLPDNISSVLNK^{+2} (UGPA_MOUSE) co-elutes with a +3 charged species (M_1 and M_3)……………………………………………………………..……….21
4. The interference in the isotope profile of peptide NAVTQEFGPVPDTAR^{+2} (ACON_MOUSE) is evident from the comparison with its interference-free profile from a replicate experiment…………………………………………………....……….22
5. The peptide HPDYSVSLLLR^{+3} (ALBU_MOUSE) co-elutes with a species of the same charge state (M_0)……………………………………………………………….23
6. Distribution of the differences of the monoisotopic RIAs computed from raw abundances of two mass isotopomers and complete isotope profiles for peptides with $R^2 \geq 0.95$ fit…………………………………………………………………...24
7. Distribution of difference in the turnover rates computed from complete isotope profiles and two mass isotopomers for peptides with $R^2 \geq 0.95$ fit…………………25
8. The joint distribution of the relative turnover rate and monoisotopic RIA errors ….26
9. The comparison of theoretical and experimental (computed from complete isotope profiles, $A_1(0)/A_0(0)$, $A_2(0)/A_0(0)$, $A_2(0)/A_1(0)$ ratios) monoisotopic RIA …………..27
10. Best fits to the theoretical monoisotopic RIAs from all data processing approaches of experimental data ………..……………….……………………………..………..29
11. The pie chart of contributions of RIA estimation methods …………………………30
12. The density plots of the relative errors of RIAs estimation methods ……………31
13. The bar charts of the number of peptides and RIA estimation accuracy…………..32
14. The significance of high mass isotopomers as a function of peptide mass………33
15. The box plot of the relative isotope error distributions along elution profile……….34
16. Density distributions of the errors of the RIAs for high and low quality fits ……..…35

17. The accuracy of the turnover rate estimations improve by using ratios …………..36
18. The density plot of the protein turnover rates for murine liver proteome…………..37
19. The protein subnetworks of the slow turnover proteins……………………………38
20. The performance of the approach using ratios on four different tissue types.........39
21. Comparison with the turnover rates of tissues with another study…………………40
22. Comparison of protein turnover rates obtained from two different labeling approaches ………………………………………………………………………….41

Supplementary Table 1 The numbers of peptides with improved monoisotopic RIAs from using a ratio of abundances of a pair of mass isotopomers ………..................................42

Supplementary Table 2 The effect of the mass accuracy on quantification of peptide features …………………………………………………..………...............................43

Supplementary Table 3 The effect of the chromatographic elution window on quantification of peptide features ………………………………..............................44

Supplementary Table 4 The effect of sample fractionation on quantification of peptide features ……………………………...45

Supplementary Table 5 The numbers of peptides and proteins that passed the various R^2 thresholds from the approaches using complete and partial isotope profiles………..46

References……………………………………………………………………………….47

Supplementary Notes

The relative isotope abundance (RIA) of the monoisotope, $I_0(t)$, is determined from the completed isotope profile of a peptide as a normalized abundance:

$$I_0(t) = \frac{A_0(t)}{\sum_{k=0}^{5} A_k(t)} \qquad \text{Eq. (1)}$$

In the above equation, $A_i(t)$ is the abundance of the ith mass isotopomer at the labeling time point t. Six mass isotopomers are normally used to compute the normalized monoisotopic RIA[1, 2].

The ratios of abundances of pairs of mass isotopomers are estimated from the following equations:

$$\frac{A_1(t)}{A_0(t)} = N_{EH} \frac{p_X(t)}{(1-p_H)(1-p_H-p_X(t))} + \frac{I_1(0)}{I_0(0)} \qquad \text{Eq. (2)}$$

The $A_2(t)/A_0(t)$ ratio is:

$$\frac{A_2(t)}{A_0(t)} = \frac{I_2(0)}{I_0(0)} - \frac{I_1(0)}{I_0(0)} b_1(0) + \frac{N_{EH}+1}{N_{EH}-1}\left(b_2(0) - b_2(t)\right) + b_1(t)\frac{I_1(t)}{I_0(t)} \qquad \text{Eq. (3)}$$

The $A_3(t)/A_0(t)$ ratio is:

$$\frac{A_3(t)}{A_0(t)} = b_3(t) + b_2(t)\left(\frac{I_1(t)}{I_0(t)} - b_1(t)\right) + b_1(t)\left\{\frac{I_2(t)}{I_0(t)} - b_1(t)\left(\frac{I_1(t)}{I_0(t)} - b_1(t)\right) - b_2(t)\right\} + \left\{\frac{I_3(0)}{I_0(0)} - c_1\frac{I_2(0)}{I_0(0)} + c_2\frac{I_1(0)}{I_0(0)} - c_3\right\} \qquad \text{Eq. (4)}$$

where

$$b_n(t) = \binom{N_{EH}}{n}\left(\frac{p_X(t)+p_H}{1-p_H-p_X(t)}\right)^n \text{ and } c_n = \binom{N_{EH}+n-1}{n}\left(\frac{p_H}{1-p_H}\right)^n$$

The $A_4(t)/A_0(t)$ ratio is obtained from the time course of the $I_4(t)$ which is derived in this work for the first time. The outlines of the derivation are following. $I_4(t)$ can be presented as a sum of five terms:

$I_4(t) = \sum_{n=0}^{4} p(A\backslash X_H = n)p(X_H = 4 - n)$

where, X_H denotes the hydrogen atoms in a peptide that are accessible to the deuterium in heavy water, $A\backslash X_H$ are all other atoms (Carbons, Oxygens, Nitrogens, Sulfurs, and Hydrogens that are non-accessible to deuterium). The probability of ^2H for the X_H atom type is ($p_X(t) + p_H$). The above representation separates the isotopologues originating from X_H from all other isotopologues. The first four terms of this equation are obtained from the previous derivations of $I_0(t)$ to $I_3(t)$. For example, for n = 0, the result is derived in the following:

$$p(A\backslash X_H = 0)p(X_H = 4) = p(A\backslash X_H = 0)p(X_H = 4) * p(X_H = 0)/p(X_H = 0) = I_0(0) * p(X_H = 4)/p(X_H = 0) = I_0(0) * \left(1 - \frac{p_X(t)}{1-p_H}\right)^{N_{EH}} * \binom{N_{EH}}{4}\left(\frac{p_H+p_X(t)}{1-p_H-p_X(t)}\right)^4 = I_0(t) * b_4(t)$$

For n = 1:

$$p(A\backslash X_H = 1)p(X_H = 3) = \frac{p(X_H=3)}{p(X_H=0)} p(A\backslash X_H = 1)p(X_H = 0) = b_3(t)\big(I_1(t) - p(A\backslash X_H = 0)\, p(X_H = 1)\big) = a_3(t)\left(I_1(t) - \frac{p(X_H=1)}{p(X_H=0)}\, p(A\backslash X_H = 0)p(X_H = 0)\right) = b_3(t)\{I_1(t) - b_1(t)\, I_0(t)\}$$

By following this approach, the expressions for n = 2 and 3 can be obtained. In the above expression for $I_4(t)$, the terms are arranged in the increasing sequence of n. The last term, in the curly brackets, corresponds to n=4.

The $A_4(t)/A_0(t)$ ratio is:

$$\frac{A_4(t)}{A_0(t)} = b_4(t) + b_3(t)\left(\frac{I_1(t)}{I_0(t)} - b_1(t)\right) + b_2(t)\left\{\frac{I_2(t)}{I_0(t)} - b_1(t)\left(\frac{I_1(t)}{I_0(t)} - b_1(t)\right) - b_2(t)\right\} + b_1(t)\left\{\frac{I_3(t)}{I_0(t)} - b_1(t)\left[\frac{I_2(t)}{I_0(t)} - b_1(t)\left(\frac{I_1(t)}{I_0(t)} - b_1(t)\right) - b_2(t)\right] - b_2(t)\left[\frac{I_1(t)}{I_0(t)} - b_1(t)\right] - b_3(t)\right\} + \left\{\frac{I_4(0)}{I_0(0)} - c_1\frac{I_3(0)}{I_0(0)} + c_2\frac{I_2(0)}{I_0(0)} - c_3\frac{I_1(0)}{I_0(0)} + c_4\right\} \quad \text{Eq. (5)}$$

The time course of the $I_5(t)$ is derived similarly, and the formula is shown in the main text.

The ratio $A_5(t)/A_0(t)$ is:

$$\frac{A_5(t)}{A_0(t)} = b_5(t) + \left(b_4(t) - b_1(t)b_3(t)\right)\left\{\frac{I_1(t)}{I_0(t)} - b_1(t)\right\} + \left(b_3(t) - b_1(t)b_2(t)\right)\left\{\frac{I_2(t)}{I_0(t)} - b_1(t)\left(\frac{I_1(t)}{I_0(t)} - b_1(t)\right) - b_2(t)\right\} + \left(b_2(t) - b_1^2(t)\right)\left\{\frac{I_3(t)}{I_0(t)} I_3 - b_1(t)\left[\frac{I_2(t)}{I_0(t)} - b_1(t)\left(\frac{I_1(t)}{I_0(t)} - b_1(t)\right) - b_2(t)\right] - b_2(t)\left[\frac{I_1(t)}{I_0(t)} - b_1(t)\right] - b_3(t)\right\} + b_1(t)\left\{\frac{I_4(t)}{I_0(t)} - b_4(t)\right\} + \left\{\frac{I_5(0)}{I_0(0)} - c_1\frac{I_4(0)}{I_0(0)} + c_2\frac{I_3(0)}{I_0(0)} - c_3\frac{I_2(0)}{I_0(0)} + c_4\frac{I_1(0)}{I_0(0)} - c_5\right\} \quad \text{Eq. (6)}$$

In the derivation for equations for $I_4(t)$ and $I_5(t)$, we have used the following identity from the combinatorics[3]:

$$\binom{a+b}{n} = \sum_{i=0}^{n}\binom{a}{i}\binom{b}{n-i}$$

In **Supplementary Eqs. (2-6)**, N_{EH} is the number of hydrogens accessible to deuterium in the peptide, $p_X(t)$ is the atomic percent enrichment (above the natural distribution of ^2H) of deuterium in the peptide, p_H is the relative abundance of ^2H in nature, $A_i(t)$ is the raw abundance of the ith mass isotopomer in the isotope profile of a peptide at labeling duration t, and $I_i(t)$ is the raw abundance of the ith mass isotopomer in the isotope profile of a peptide at labeling duration t.

In all cases, when $I_i(t)/I_j(t)$ is present in **Eqs. (1-5)**, the corresponding theoretical values were used. $A_i(t)/A_j(t)$ ratios were obtained from the experimental data.

As a goodness-of-fit of the theoretical exponential decay model to the experimental time series data, we used coefficient of determination (R^2), Pearson correlation, and the residual standard error between the experimental time course data and theoretical fit.

There are several definitions of the coefficient of determination[4]. In this work, the ratio of the regression sum of squares to the total sum of squares was used:

$$R^2 = 1 - \frac{\sum_{i=1}^{N}\left(I_0(t_i) - \hat{I}_0(t_i)\right)^2}{\sum_{i=1}^{N}(I_0(t_i) - \bar{I_0})^2} \qquad \text{Eq. (7)}$$

where, $I_0(t_i)$ is the monoisotopic RIA at the labeling time point t_i (i =1 is the RIA before the start of the labeling), $\hat{I}_0(t_i)$ is the theoretical fit (obtained using Eq. (1) in the main text) to the RIA at time point t_i, and $\bar{I_0}$ is the average monoisotopic RIA of all time points of labeling. N is the number of labeling time points.

The residual standard error, sde, was determined from the residual sum of squares of the model and the degrees of freedom:

$$sde = \sigma_0 = \sqrt{\sum_{i=1}^{N}\left(I_0(t_i) - \hat{I}_0(t_i)\right)^2 /(N-1)} \qquad \text{Eq. (8)}$$

The standard deviation of the rate constant is estimated using the Delta method[5]. A homoscedastic variance, $\mathrm{Var}(I_0(t)) = \sigma_0^2$, is assumed for $I_0(t)$ at all time points. The standard deviation of the rate constant is:

$$sd(k) = \sigma_k = \sigma_0 / \sqrt{\sum_{i=1}^{N} t_i^2\left(\hat{I}_0(t_i) - I_0^{asymp}\right)^2} \qquad \text{Eq. (9)}$$

I_0^{asymp} is the monoisotopic RIA achieved at the plateau of labeling. It is obtained from N_{EH}, the deuterium enrichment of body water (p_W), and p_H:

$$I_0^{asymp} = I_0(0)\left(1 - \frac{p_W}{1-p_H}\right)^{N_{EH}} \qquad \text{Eq. (10)}$$

The confidence intervals (CIs) for parameter estimation in non-linear regression models are determined using the t-test statistic. For a confidence level (1−α), the confidence region of the rate constant, k, is:

$$k \pm t_{\left(1-\frac{\alpha}{2}\right),(N-1)}\sigma_k \qquad \text{Eq. (11)}$$

$t_{\left(1-\frac{\alpha}{2}\right),(N-1)}$ is the (1− α/2) quantile of the t distribution with (N – 1) degrees of freedom. For 95% CI, α is equal to 0.05.

The CI of a protein was computed from the rate constants and standard deviations of its peptides. The protein turnover rate, k_{prot}, is assumed to be the median of the turnover rates of its peptides. The standard deviation of the protein rate is assumed to be the harmonic mean of the standard deviations of its peptide:

$$\sigma_{prot} = 1 \Big/ \sqrt{\sum_{i=1}^{K} 1 \Big/ \sigma_{i,pep}^2}$$

where K is the number of peptides that passed the goodness-of-fit threshold. The 95% CI interval for protein turnover is:

$$k_{prot} \pm 2\sigma_{prot} \qquad \text{Eq. (12)}$$

If only one peptide of a protein passes the goodness-of-fit threshold, then the CI of the peptide turnover rate is used for the protein turnover rate.

Animal experiments

Eighteen, four-month-old, C57/BL6J male mice were purchased from The Jackson Laboratory and housed in germ-free cages at UTMB. All animals were provided free access to the standard lab chow. Two mice were not labeled and their tissues were used to estimate natural isotope abundances. To initiate the deuterium labeling, each of the sixteen mice was IP injected 750-960 ul (5% body water enrichment based on 60% of body weight as water) of 99.9% D_2O (Cambridge Isotope Laboratories) made isotonic with 0.9 g NaCl w/v. Immediately thereafter mice were given free access to 8% enriched (v/v) deuterated water for the duration of the labeling. The labeling was carried for 0, 1, 2, 3, 4, 5, 6, 14 and 21 days. The labeling time points were determined using an approach

for optimization of labeling durations[6]. At each time point, two mice were euthanized with CO_2 and dissected livers were flash frozen in liquid nitrogen and stored at -80^0 C prior to the analysis. To determine body water enrichment in deuterium, blood was collected from mice via cardiac puncture using a syringe with 25G needle.

All mice experiments were carried out according to the Guide for Care and Use of Laboratory Animals by the National Research Council and approved by the Institutional Animal Care and Use Committee of UTMB. A UTMB veterinarian technician performed IP injection, euthanization, and tissue extractions.

Determination of body water enrichment

Body water deuterium enrichment was determined in plasma by isotope ratio mass spectrometry as described previously[7]. Briefly, 10 µl of plasma was diluted to a final volume of 200 µl in water and placed in an Exetainer® (Labco Ltd., UK), along with 5 mg of activated charcoal, 200 mg of copper powder, and a platinum catalytic rod. Afterwards, the Exetainer® was loaded on to a Gasbench II online gas preparation and introduction system (ThermoFisher Scientific, MA, USA), where the sample was flushed for 7 min with 2% H_2 in helium, before being allowed to equilibrate for 4 h. On equilibration, an aliquot of the gas headspace was injected into a Delta V Advantage isotope ratio mass spectrometer (ThermoFisher Scientific, MA, USA), and the ratio of deuterium to hydrogen determined in duplicate. Linearity of the machine over the expected range of deuterium body water enrichment was confirmed by the generation of a standard curve using 99.9% deuterium-enriched water (Sigma-Aldrich, St. Louis, MO, USA).

Sample processing

Peptide sample preparation. Approximately 100 mg of liver was diced and placed into 0.5 mL of 5% SDS, 50 mM TEAB, 1 mM TCEP solution in a clean 2mL Nunc screw cap tube. 3uL of benzonase nuclease (Sigma) was added to the solution followed by approximately 0.5 mL of 1.0 mm zirconia/silica beads (BioSpec) homogenized by bead beating was performed for 20 seconds using a Soni-Beast (BioSpec).. The sample was subjected to a second round of bead beating and repeated as necessary to fully homogenize the tissue. The sample was cooled to room temperature and 2 µL of 0.5 M iodoacetamide acid added and allowed to react for 20 minutes in the dark. The supernatant was transferred to a clean 15 mL Falcon tube and 2 µL of freshly prepared 0.5 M Dithiothreitol added and incubated at room temperature for 10 minutes. Proteins were precipitated using 3mL of ice-cold acetone and centrifuged after storing overnight at -20C. Acetone was decanted and the resulting pellet diluted in 5% SDS, 50 mM TEAB, pH 7.1, to a final volume of 200 µL. The samples were then centrifuged at 17,000*g* for 10 minutes to remove any debris. Next, 2.75 µL of 12% phosphoric acid was added to the protein solution, and 165 µL of binding buffer (90% methanol, 100 mM TEAB, final pH 7.1) was then added to the solution. The resulting solution was added to S-Trap spin column (protifi.com) and passed through the column using a benchtop centrifuge (30-second spin at 4,000*g*). The spin column was washed with 400 µL of binding buffer (90% methanol, 100mM TEAB, pH = 7.55) and centrifuged. This was repeated 2 more times. Trypsin was added to the protein mixture in a ratio of 1:25 in 50 mM TEAB, pH 8, and incubated at 37°C for 4 hours. Peptides were eluted with 80 µL of 50 mM TEAB, followed by 80 µL of 0.2% formic acid, and finally 80 µL of 50% acetonitrile, and 0.2% formic acid.

The combined peptide solution was then dried in a speed vac and resuspended in 2% acetonitrile, 0.1% formic acid, 97.9% water and placed in an autosampler vial.

NanoLC MS/MS Analysis

Peptide mixtures were analyzed by nanoflow liquid chromatography-tandem mass spectrometry (nanoLC-MS/MS) using a nano-LC chromatography system (UltiMate 3000 RSLCnano, Dionex), coupled on-line to a Thermo Orbitrap Eclipse mass spectrometer (Thermo Fisher Scientific, San Jose, CA) through a nanospray ion source. The sample was loading directly onto the analytical column, an Aurora (75um X 25 cm, 1.6 µm) from (Ionopticks). After equilibrating the column in 99% solvent A (0.1% formic acid in water) and 1% solvent B (0.1% formic acid in acetonitrile (ACN)), the samples (2 µL in solvent A) and subsequently eluted (400 nL/min) by gradient elution onto the C18 column as follows: isocratic at 2% B, 0-5 min; 2% to 5% 5-6 min, 4% to 25% B, 6-170 min; 25% to 38% B, 170-196 min; 38% to 90% B, 196-199 min; isocratic at 90% B, 199-200 min; 90% to 5%, 200-201 min; 5% 201-202.5 min; 5%-90% 202.5-203 min, isocratic at 90% B, for 1min and 90% to 1%, 204-205 min and held there for 15 minutes.

The separated peptides were ionized at a spray voltage of 1500V, and the ions were collected into an ion transfer capillary set at 300°C. The RF lens was set to 30%. The data-dependent acquisition was used by first obtaining MS1 data in the Orbitrap at a resolution of 60,000 (at an m/z = 200) and a maximum injection time of 50 ms for target values of 400,000 ions (normal AGC target = 100%) in the 380–1400 m/z mass range. Precursor ions with charges of +2 to +8 and intensities greater than 10,000 were selected

for MS/MS sequencing with a maximum fill time of 35 ms using the top speed option. Precursor ions were isolated with an isolation window of 1.4 Da and fragmented by high energy dissociation (HCD) set at normalized collision energy (NCE) of 30%. Dynamic exclusion time was set to 60 s.

Protein identification

Raw mass spectral data were converted to mzML format using the MSConvert tool of proteowizard[8] version 3.0.22048. Peptides were identified from tandem mass spectra using the database search engine Mascot[9] (Matrix Science, Boston, MA) version 2.5. SwissProt protein sequence database (12 February 2022 release) was used. The database search parameters were set to Mus Musculus for taxonomy, 15 ppm (part per million) mass tolerance for peptide precursors, 0.6 Da mass tolerance for fragment ions, fixed modification of Cys residues with the carbamidomethylation, variable oxidation of Met (+15.9949), and variable protein N-terminal acetylation. # ^{13}C was set to 2, which allows for 0/1/2 isotope errors. This setting permits the consideration of precursor selections from higher mass isotopomers. Trypsin was specified as the protease with up to 3 missed cleavage sites. The false discovery rate (FDR) was controlled by reversed database approach. Peptide FDR was set to 1%. Only peptides with the highest score (Mascot bold red peptides) of matching to tandem mass spectra were retained.

Peptide fractionation. For one (unlabeled) sample we performed peptide fractionation to reduce the proteome complexity and compare the performance of the methods to estimate the monoisotopic RIA. Proteolytic digests from the mouse liver proteins onto an

equilibrated, high-pH, reversed-phase fractionation spin column (Thermo Cat#84868). Peptides are bound to the resin under aqueous conditions and desalted by washing the column with water by low-speed centrifugation. A step gradient of increasing acetonitrile concentrations (5-50% Acetonitrile each with 0.1% Trietylamine) in a volatile is applied to the columns to elute bound peptides into eight different fractions collected by centrifugation. Each fraction is then dried in a vacuum centrifuge, reconstituted in 2% acetonitrile, 1% acetic acid, and 0.08% formic acid, and put in an autosampler for LCMS analysis using a 90-minute gradient and the same settings as the long gradient study.

d2ome+ workflow

d2ome+ takes a spectral data set, protein identification results, body water enrichment, and labeling time course as input. Its output is the protein turnover rate. For spectral data set and protein identification results, it accepts files in the Human Proteome Organizations' Proteomics Standard Initiative's formats - mzML[10] and mzIdentML[11], respectively. The tool collects the list of peptides that pass user-specified thresholds for protein/peptide identification. For each peptide in every experiment, it performs peak detection and integration in the chromatographic time domain. The formulas (shown in the Methods section) for RIAs of mass isotopomers are used to estimate if the maximum RIA of a mass isotopomer at specified body water enrichment (p_W) is higher than 1%. If the RIA does not pass the threshold, it is not included in the isotope profile. The exclusion reduces the chances of the contributions from the contaminants. Abundances of up to six mass isotopomers for each peptide at every time point of labeling are reported in a Protein_Name.Quant.csv file. Each csv file contains constituent peptides of a single

protein. For every peptide, the tool computes $I_0(t)$ and estimates the $p_X(t)$ values from $A_2(t)/A_0(t)$, $A_2(t)/A_1(t)$, and $A_1(t)/A_0(t)$ ratios. From these estimated ratios, $I_0(t)$ is reconstructed using **Eq. (3)** in the main text. The newly generated data is fit to **Eq. (1)** in the main text, and a new R^2 is obtained. Often, only one of the ratios provides an improved estimate of the label incorporation. The turnover rates and their confidence intervals for each protein and its constituent peptides are reported in the Protein_Name.RateConstant.csv file. Analyzed_Proteins.csv file contains combined results from all proteins. From the graphical user interface, the choice of complete or partial isotope profiles for computing monoisotopic RIA can be made. The adjustable parameters include peptide mass accuracy, protein/peptide consistencies (the minimum number of experiments where a protein/peptide has been identified), and chromatographic elution window.

Comparison of Complete and Partial Isotope Profile methods. The estimations of RIAs of unlabeled peptides from each of the approaches for all peptides (without filtering) are shown in **Supplementary Figure 12** as density plots of the relative differences with respect to the theoretically calculated (from natural isotope distributions) monoisotopic RIAs. The trends observed for the filtered data (**Supplementary Figure 10**) are conserved for the full data set. The density functions of errors from RIA estimations using the A_2/A_0 and A_1/A_0 ratios were close to that of the complete isotope profiles in the small-error region (less than 10% relative error). Next, we analyzed relative errors in RIA estimations from each of the methods for possible overlaps (the numbers of peptides that were accurately estimated by several methods), **Supplementary Figure 13 A-D**. As seen from the histograms, the methods were complementary. For example, for 6693 peptides,

monoisotopic RIAs computed using the A_2/A_0 ratio were the most (compared to other ratios and complete isotope profiles) accurate and within one and five percent of the corresponding theoretical RIAs, **Supplementary Figure 13 C**. For 1379 of these peptides, the complete isotope profile method also produces the monoisotopic RIA to within one to five percent relative error. For 1960 peptides, only the A_2/A_0 ratio could estimate the RIA to this accuracy. Except that for A_2/A_1, all histograms had a mode in the relative error range of one to five percent.

To evaluate the isotopic properties of the peptides whose monoisotopic RIAs are improved by the new method, we analyzed the results based on the relative monoisotopic RIA errors. It was assumed that in the cases when the complete isotope profile produced monoisotopic RIA within 8% of the theoretical value, the improvements by the new method could possibly be due to the fluctuations in the isotope profiles. However, if the relative error of the monoisotopic RIA from the complete isotope profile was more than 20% (see below the reasons for selecting the thresholds), and the error was reduced to less than 8% by the new method, then it was assumed that the isotope profile had interferences from co-eluting species which corrected by the new method. For one experiment (from the unlabeled sample), we present all peptides and the estimations of the monoisotopic RIAs from all methods, **Supplementary Data 2**. From the data, **Supplementary Table 1** was generated. The table shows the numbers of peptides classified by their abundances and relative errors with original and new methods. It is divided into two parts, which correspond to the overestimations and underestimations of the monoisotopic RIA by the method using complete isotope profiles. As is seen from the table, in more than 50% of cases, the new method corrects monoisotopic RIAs, which

were underestimated by the original method. These correspond to the co-elutions of contaminants, which affect the high mass isotopomers of target peptides. The 20% threshold for the relative error was chosen based on the median monoisotopic RIA (0.34), median N_{EH} (32), the median turnover rate of murine liver proteins (0.28 day^{-1}), body water enrichment (0.05), and the shortest duration of labeling in this experiment (one day). The relative change of the monoisotopic RIA for median values of the inputs resulted in 0.18. Relative errors larger than this will make the RIA computed from the unlabeled sample indistinguishable from the monoisotopic RIA of the labeled samples. The tolerable error in the monoisotopic RIA was assumed be less than the half of the 0.18 and was chosen to be 0.8.

In the above analyses, when computing the monoisotopic RIA from complete isotope profiles, six mass isotopomers were used. It may seem counterintuitive that the high mass isotopomers contributed significantly to the isotope profiles. However, the high mass isotopomers become important (RIA > 0.03) for labeled peptides, even with small masses. This is shown in **Supplementary Figure 14**. The figure shows the proportions of peptides (from mouse proteome in SwissProt) for which $I_3(t)$, $I_4(t)$, and $I_5(t)$ are higher than 0.03 in nature (continuous lines) and after being labeled with 5% enriched heavy water (dashed lines). As seen from the figure, for more than half of all peptides with masses higher than 1.3 kDa, their mass isotopomers have RIAs higher than 0.03 when labeled with 5% heavy water. Therefore, for accurate estimation of the monoisotopic RIA from the complete isotope profiles, for most of the peptides, up to six mass isotopomers are required. The more mass isotopomers are required for computing the monoisotopic RIA, the more the chances of overlapping with the isotope profiles of other peptides.

The effects of mass accuracy and elution window on the partial and complete isotope profile estimations of the monoisotopic RIA. For an unlabeled sample, we have analyzed the effects of the mass accuracy and chromatographic elution time window on the accuracy of the monoisotopic RIA estimations. We fixed the chromatographic elution window (at 1 minute), varied the mass accuracy, and computed the percentage of peptide features that were accurately quantified by complete and partial isotopes methods, **Supplementary Table 2**. As the mass accuracy was reduced, the percentage of peptide features accurately computed using complete isotope profiles monotonically decreased, and the percentage of peptide features with high relative isotope error (> 0.2) monotonically increased. The percentage of peptide features whose monoisotopic RIA estimation improved by the partial isotope approach has slightly increased with the decrease in the mass accuracy. It is explained by how the peak detection algorithm works[12, 13]. It identifies the m/z peak, which is the nearest to the theoretical m/z. Therefore, the increase in the mass accuracy will mostly affect the low abundance high mass isotopomers, which may be absent when high mass accuracy is used. The first three mass isotopomers, which are used in partial isotope profiles, corrected the estimation of the monoisotopic RIA.

To analyze the effect of the elution window on the approaches to estimate the monoisotopic RIA, we fixed the mass accuracy at 20 ppm and varied the chromatographic elution window. The results are shown in **Supplementary Table 3**. As the elution window increased, the accuracy of the monoisotopic RIA estimation by both methods decreased. When the elution window increases, the peak detection (based on the nearest m/z with respect to the theoretical monoisotopic m/z) tends to pick a co-eluting contaminant. It

resulted in wrong isotope profiles for the complete and first three mass isotopomers. The accuracy of the monoisotopic RIA estimation by both methods suffered.

The analysis of the estimation of monoisotopic RIA in a fractionated data set. For the unlabeled sample, we performed peptide fraction (described in the experimental data collection section of the Supplementary Note). We determined the numbers of the accurately (within 8% of the theoretical value) estimated RIA from peptide features in eight fractions. In all fractions, the percentage peptide features for which the RIA was accurately estimated from the complete isotope profile approach was higher than that in the unfractionated sample, **Supplementary Table 4**. The percentage was the highest for the least complex samples – the sample with the smallest number of peptide features. Combining data from all fractions doubled the number of quantified peptide features and increased their percentage of accurately estimated RIAs from the complete isotope profile approach. Even for the combined data set, the partial isotope profiles improved the RIA estimation for 45% of peptide features whose RIA estimation from the complete isotope profile approach had higher than 20% relative error.

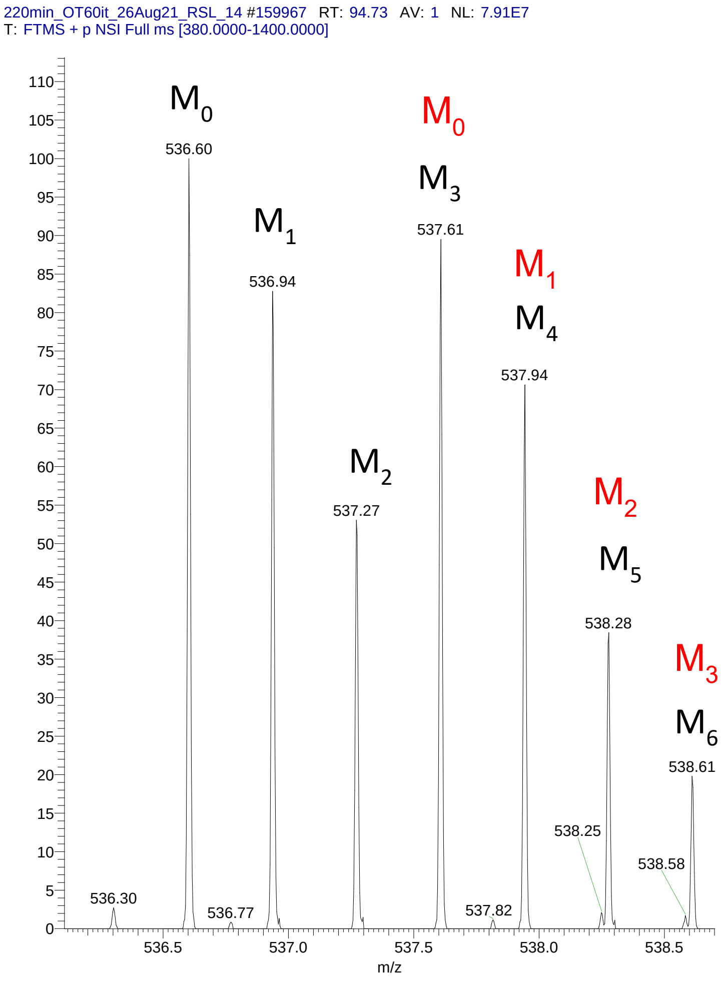

Supplementary Figure 1. The raw abundances of M_0, M_1, or M_2 mass isotopomers correctly estimate $I_0(t)$. The figure shows the MS1 profile of the VLGTSVESIMATEDR^{+3} (CPSM_MOUSE) at the apex of its elution. As seen from the figure, the M_3-M_5 mass isotopomers were affected by the interferences from mass isotopomers (shown in red) of a co-eluting peptide. Therefore, the complete isotope profile underestimates $I_0(t)$. However, estimation of the label enrichment from M_0, M_1, and/or M_2 correctly reconstructs $I_0(t)$. The R^2 from the complete isotope profile was 0.74. It changed to 0.98 after estimation of $I_0(t)$ using the $A_1(t)/A_0(t)$ ratio. The time course of the peptide is shown on page 1 of **Supplementary Data 1**. The above spectrum corresponds to one day of labeling.

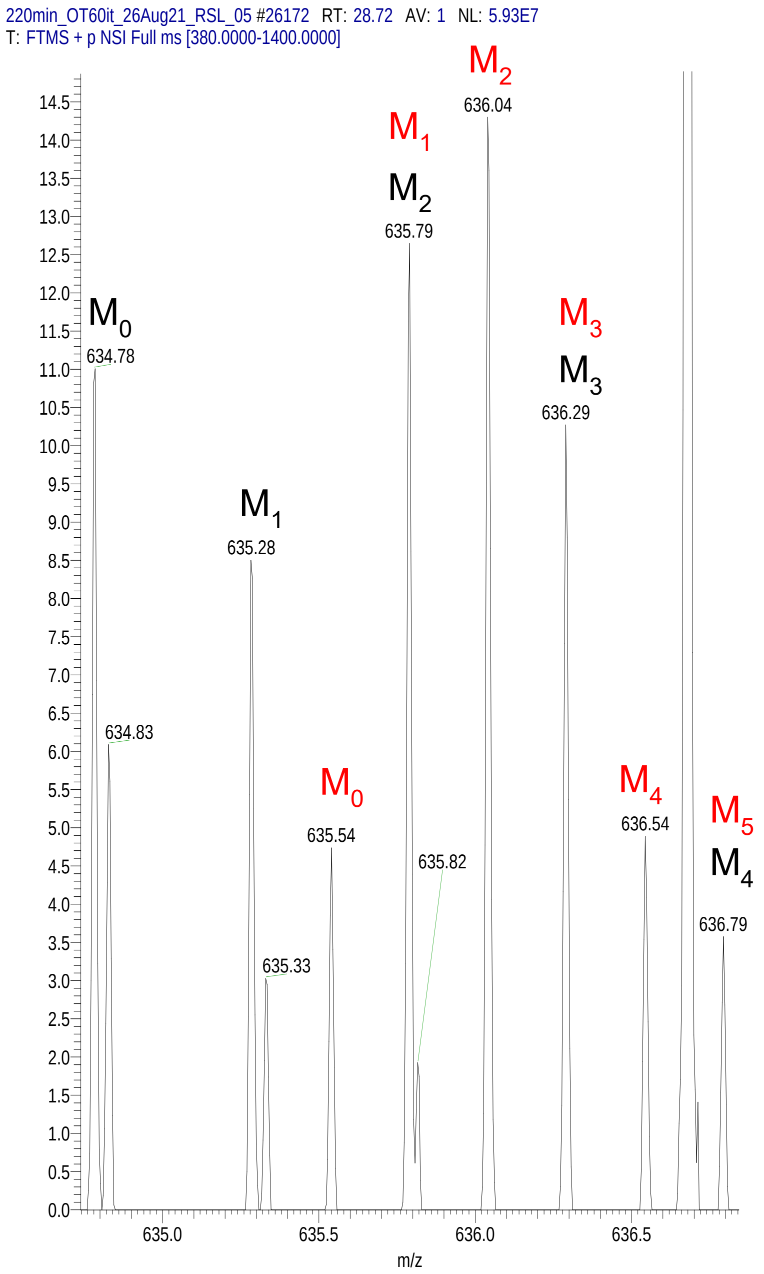

Supplementary Figure 2. M_2 and higher mass isotopomers of the peptide, YLAEVAcGDDR^{+2} (1433T_MOUSE) co-elute with the contaminant. The mass isotopomers of the contaminant, monoisotopic m/z = 635.54 and z = +4, are denoted in red. The complete isotope profile underestimates $I_0(t)$. The $A_1(t)/A_0(t)$ ratio reconstructs $I_0(t)$ correctly. Shown is a segment of mass spectral data for the peptide at the apex of its elution. The time course of the peptide is shown on page 1 of **Supplementary Data 1**.

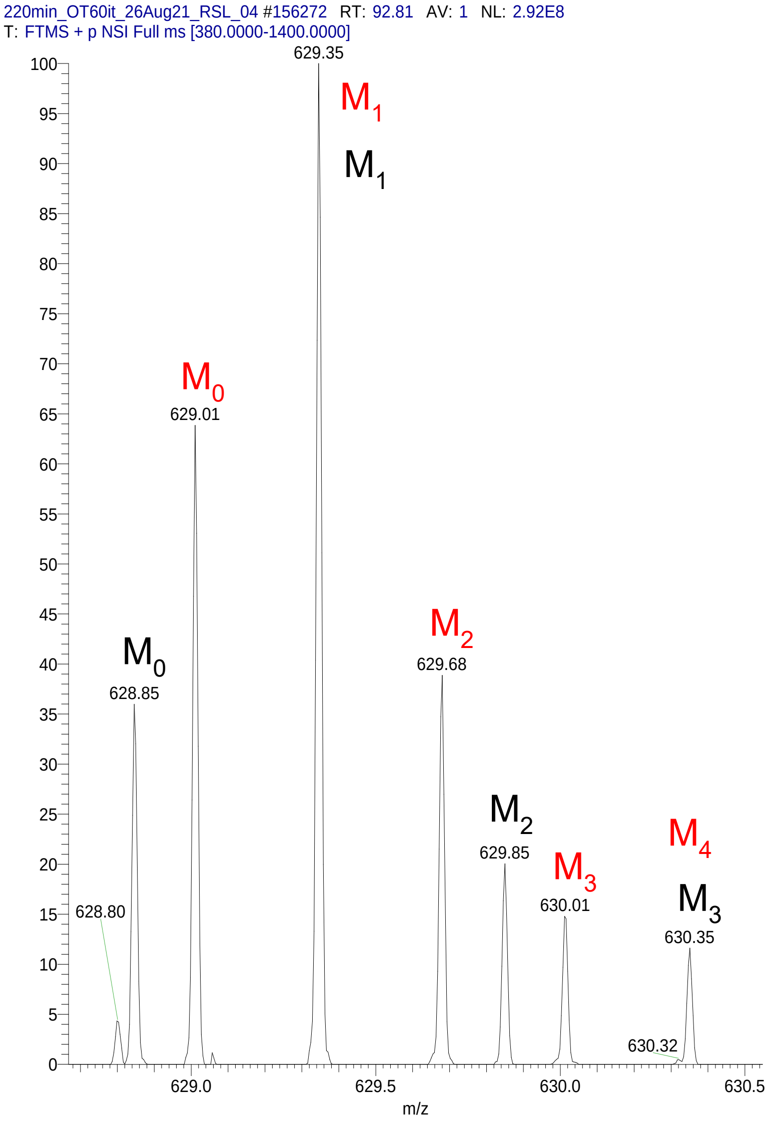

Supplementary Figure 3. Relative abundances of M_0 and M_2 mass isotopomers of the peptide GLPDNISSVLNK^{+2} (UGPA_MOUSE) correctly estimate $I_0(t)$. The M_1 and M_3 mass isotopomers of the peptide overlaps (up to the mass accuracy of the MS) with the M_1 and M_4 (in red) of the contaminant (+3 charged), respectively. Therefore, the monoisotopic RIA is underestimated from the complete isotope profile. However, M_0 and M_2 are free of any interference. The use of raw abundances of these mass isotopomers better estimates the label enrichment and, subsequently, the RIA of the monoisotope. The time course of the peptide is shown on page 1 of **Supplementary Data 1**.

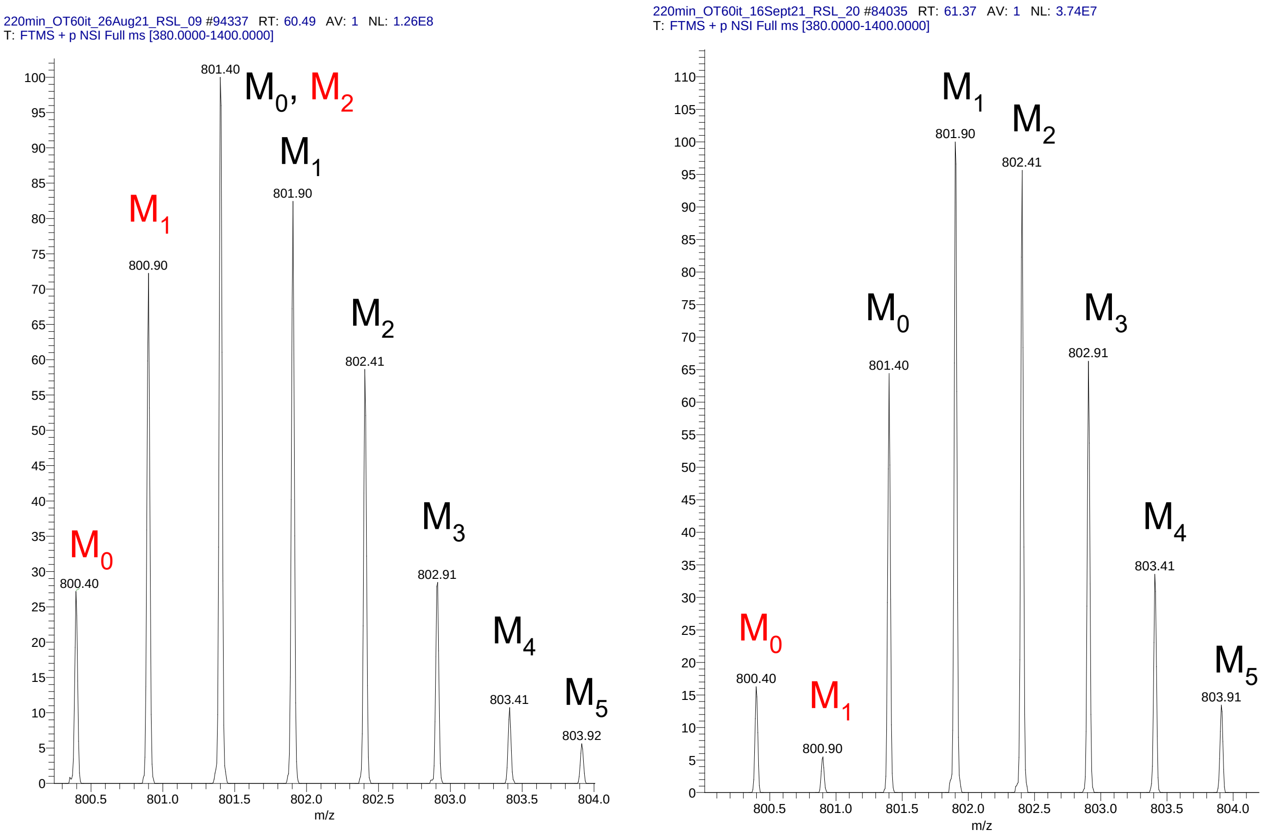

A **B**

Supplementary Figure 4. Relative abundances of the $M_{0\text{-}5}$ mass isotopomers of peptide NAVTQEFGPVPDTAR^{+2} (ACON_MOUSE), and a co-eluting peptide (annotated in red) after 14 days of labeling. **A** and **B** show the MS1 profiles in two replicate experiments. As seen from the figures, in experiment **A**, the relative contribution from the contaminant was high and strongly affected the M_0 of the peptide. The monoisotopic RIA obtained from averaging those (computed from complete isotope profiles) of replicates was overestimated and was equal to 0.26. The A_1/A_2 ratios are less affected by these co-elutions and provide the RIA equal to 0.17, which improved R^2 of the theoretical fit. The time course of the peptide is shown on page 1 of **Supplementary Data 1**.

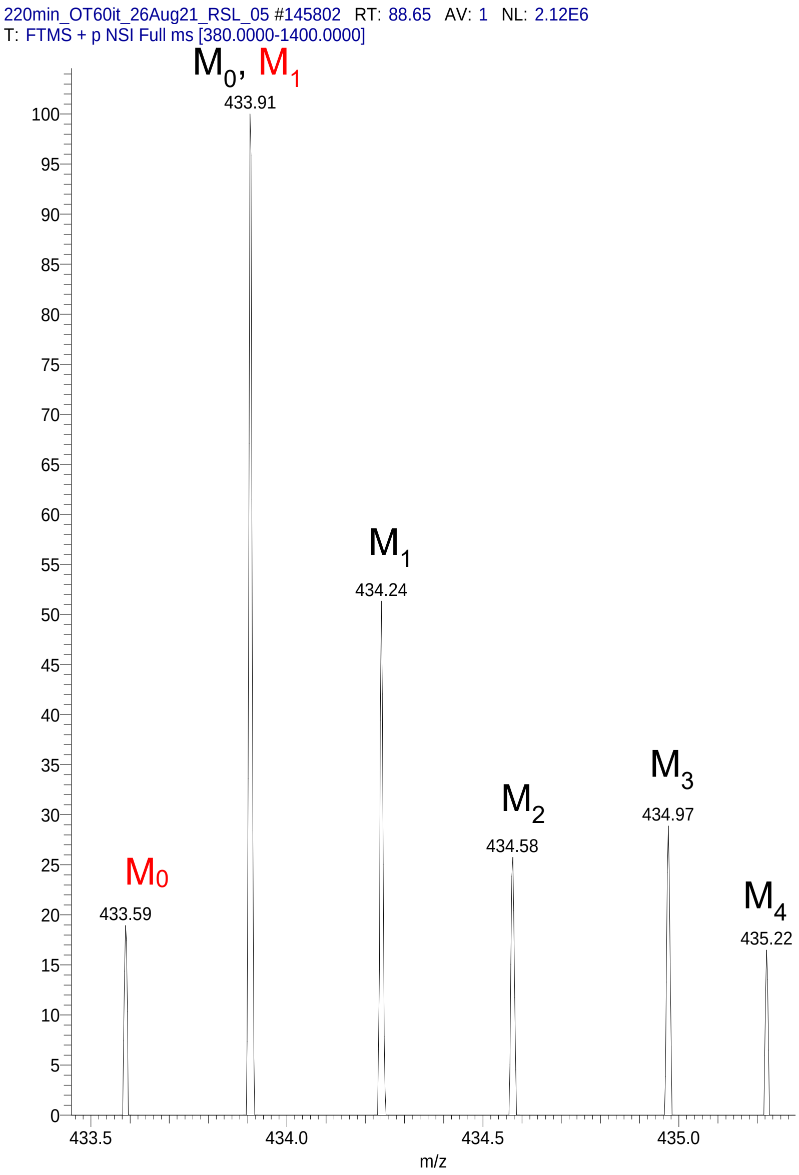

Figure 5. The abundances of M_1 and M_2 mass isotopomers of peptide, HPDYSVSLLLR^{+3} (ALBU_MOUSE) correctly estimate $I_0(t)$. Shown is the MS1 profile of the peptide at the apex of its elution. As seen from the figure, the monoisotope (M_0) is interfered with by a contaminant (red colored C). The complete isotope profile overestimates the RIA. The estimation of the label enrichment from M_1 and M_2 combined with $I_1(0)$ and $I_2(0)$ correctly reconstructs $I_0(t)$. The relative abundance of M_3 was less than 5%. It was not recorded by the MS. The time course of the peptide is shown on page 1 of **Supplementary Data 1**.

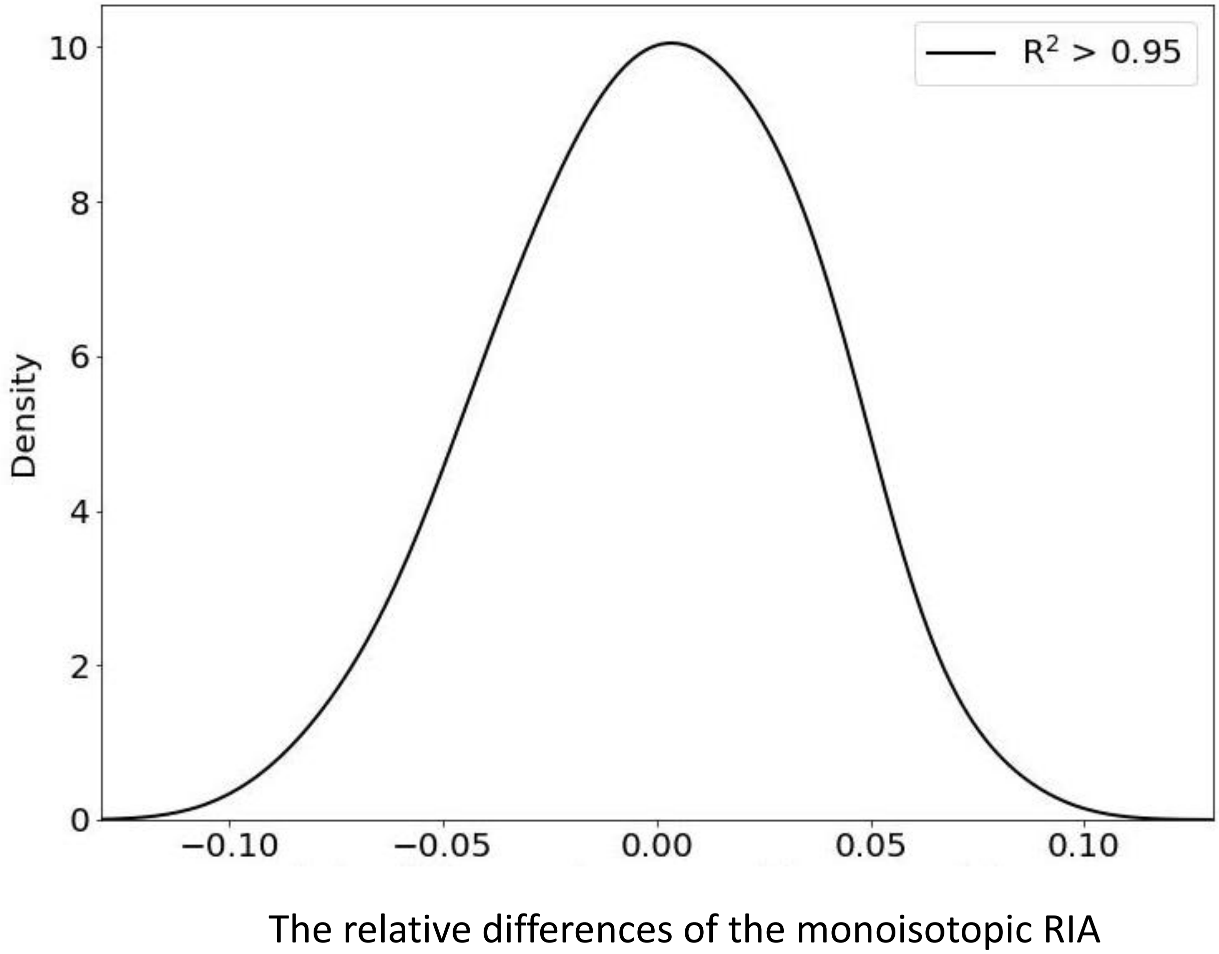

Supplementary Figure 6. For high quality theoretical fits ($R^2 \geq 0.95$) to the experimental time course of $I_0(t)$, the monoisotopic RIA computed from raw abundances of two mass isotopomers and complete isotope profiles are equivalent. The figure shows the density plot of the relative difference between the two RIAs, $\left(I_0(t) - \widetilde{I_0(t)}\right)/I_0(t)$.

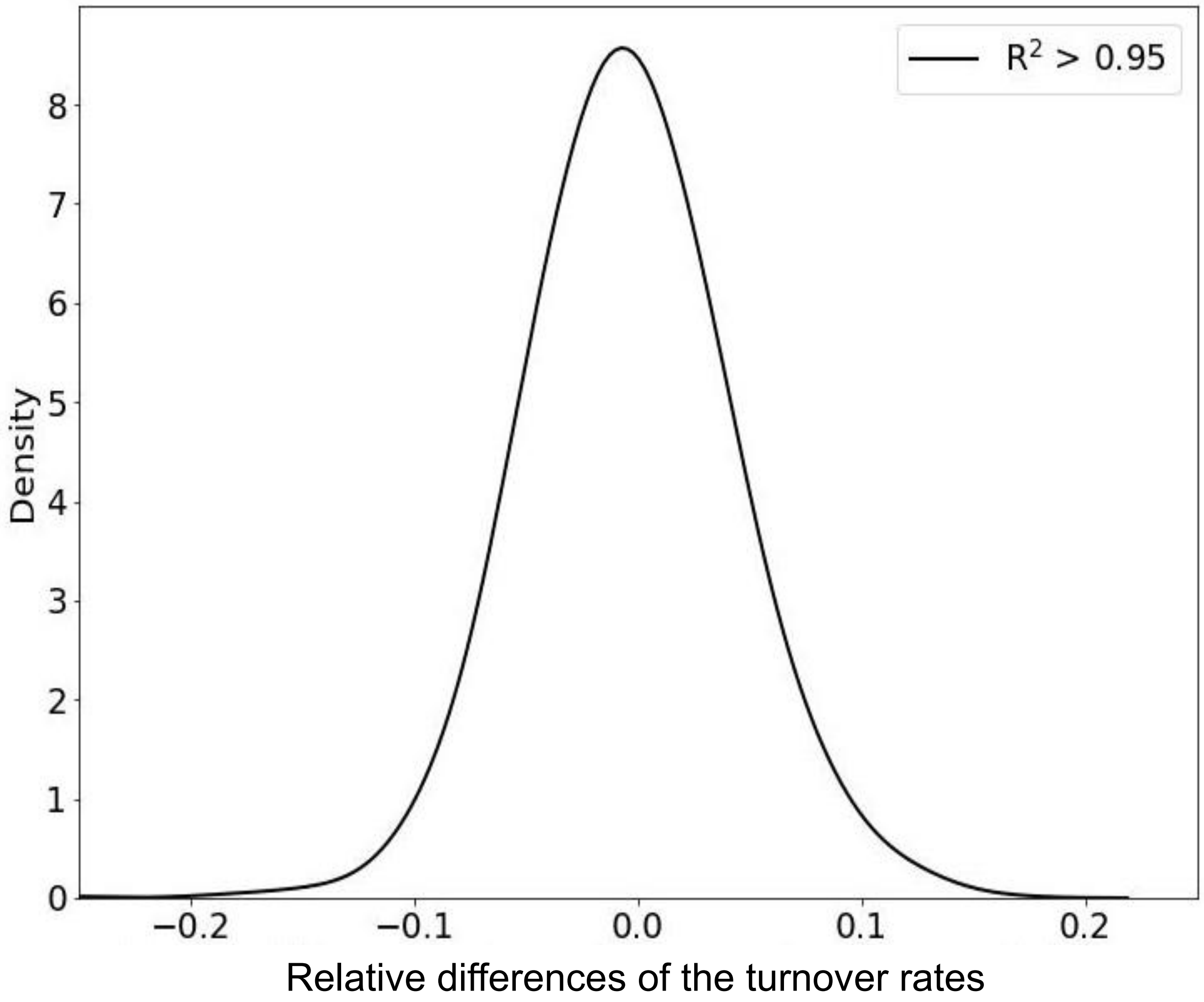

Supplementary Figure 7. For the high-quality theoretical fits, $R^2 \geq 0.95$, the turnover rates computed from complete isotope profiles and two mass isotopomers are equivalent. The figure shows the density (y-axis) plot of the relative differences of turnover rates (x-axis) from the complete isotope profiles and a ratio of abundances of two mass isotopomers.

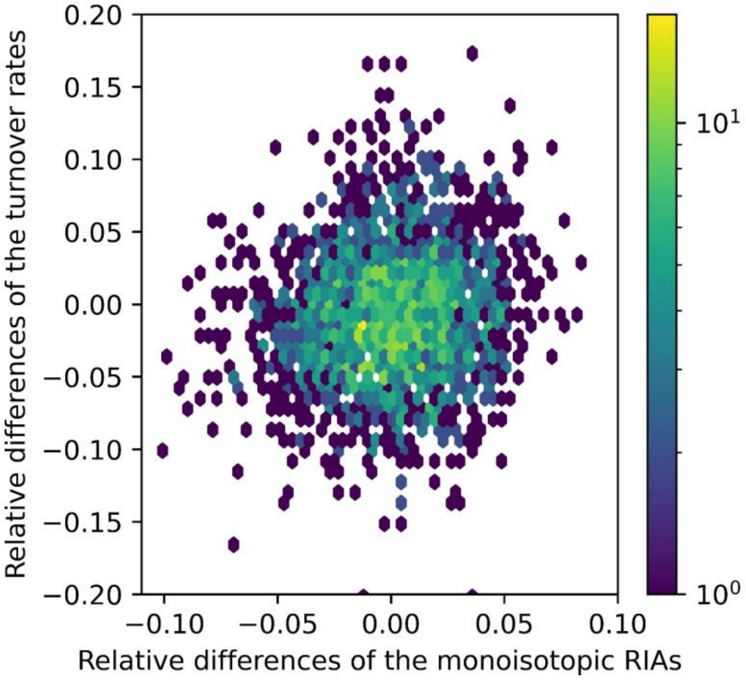

Supplementary Figure 8. The new approach, which uses two mass isotopomers for label enrichment estimation, reproduces turnover rates obtained from using the traditional approach based on the complete isotope profile. The scatter plot shows the relative differences between the turnover rates (y-axis) and the monoisotopic RIAs (x-axis) computed by the two methods. As in **Supplementary Figures 6 and 7**, the data is shown for peptides whose coefficient of determination to the experimental data was no less than 0.95, $R^2 \geq 0.95$.

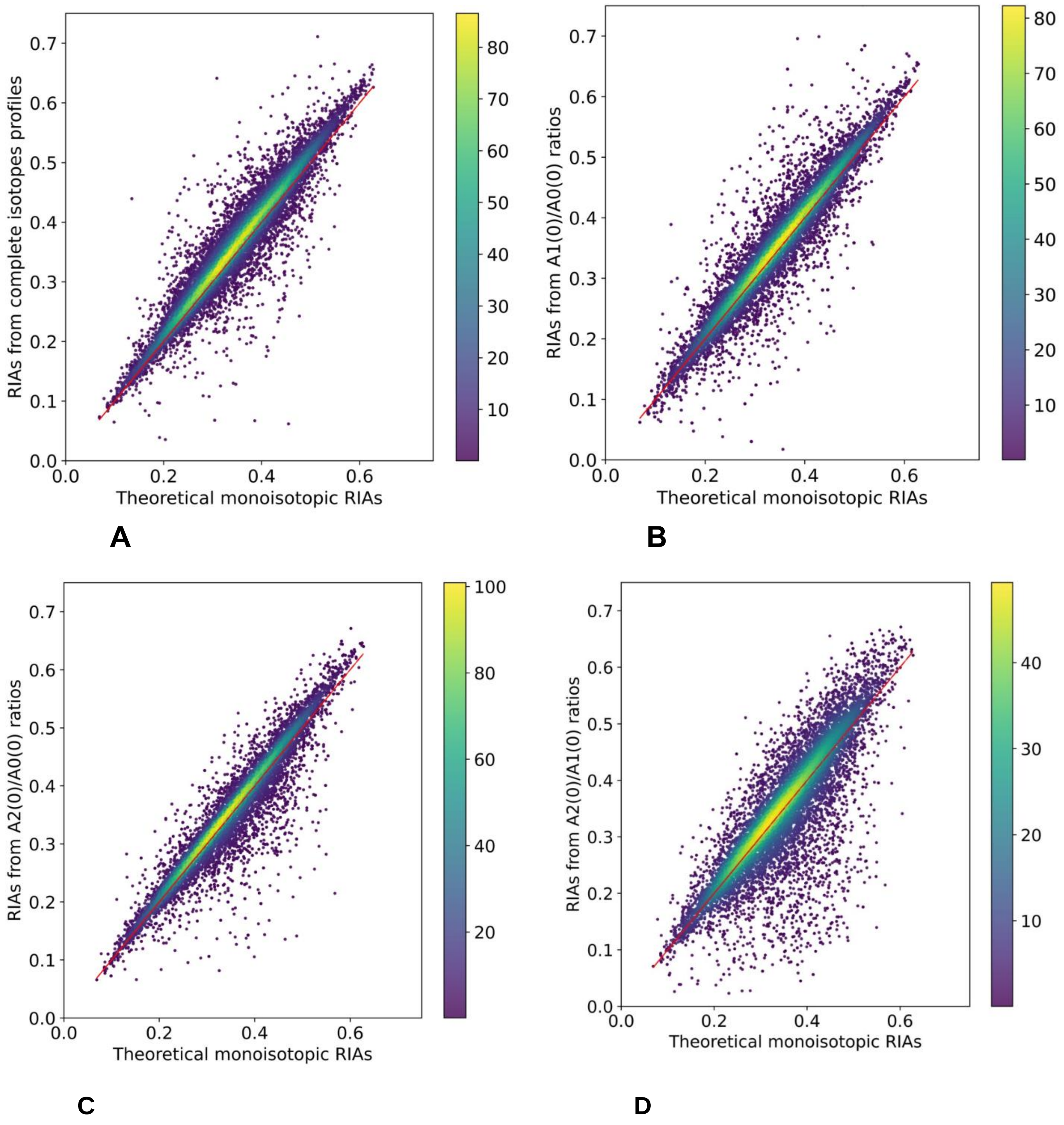

Supplementary Figure 9. The comparison of the theoretical and experimental estimations of the monoisotopic RIA. Shown are the experimentally measured RIAs (y-

axis) and the corresponding theoretical RIAs (x-axis). The red line is the identity line. The data is shown for the labeling time point zero (prior to the commencement of labeling). The peptides were selected such that the coefficient of determination between theoretical and experimental time course data was not less than 0.75. **A**. Experimental RIAs are computed from complete isotope profiles. The estimations of the monoisotopic RIAs from complete isotope profiles of peptides are not ideal. The mean and standard deviation of the relative RIAs differences were 0.0427 and 0.093, respectively. Pearson correlation between the RIAs was 0.95. **B**. The experimental RIAs are computed from the $A_1(0)/A_0(0)$ ratios. The mean and standard deviation of the relative differences in RIAs were 0.028 and 0.104, respectively. Pearson correlation between the RIAs was 0.95. **C.** The experimental RIAs were computed from the $A_2(0)/A_0(0)$ ratios. The mean and standard deviation of the relative differences of the RIAs were 0.011 and 0.085, respectively. Pearson correlation between the RIAs was 0.95. **D**. The experimental RIAs were computed from the $A_2(0)/A_1(0)$ ratios. The mean and standard deviation of the relative differences of the RIAs were -0.0053 and 0.162, respectively. Pearson correlation between the RIAs was 0.84.

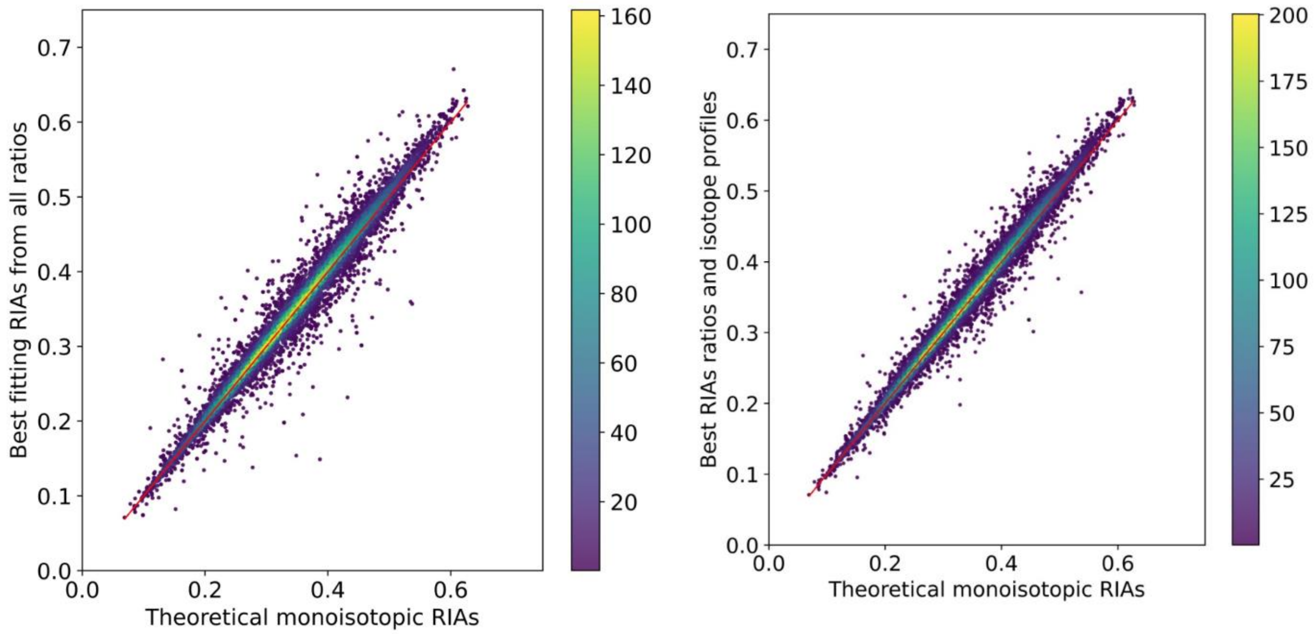

A **B**

Supplementary Figure 10. The best match to the theoretical monoisotopic RIAs. Shown are the scatter plots of RIAs obtained from the experiments (y-axis) and theoretical isotope distributions (x-axis). The peptides were selected as in **Supplementary Figures 9**, $R^2 \geq 0.75$. **A.** When three ratios are combined to select the best fit to the theoretical distribution, the deviations of produced RIAs from the theoretical RIAs reduced significantly. The mean and standard deviation of the relative differences of the RIAs were - 0.0096 and 0.049, respectively. Pearson correlation between the RIAs was 0.98. **B**. RIA estimates from the complete isotope profiles and any of the three ratios are combined to select the best fit to the theoretical distribution. The deviations of produced RIAs from the theoretical RIAs were the smallest. The mean and standard deviation of the relative differences of the RIAs were 0.0097 and 0.037, respectively. Pearson correlation between the RIAs was 0.99.

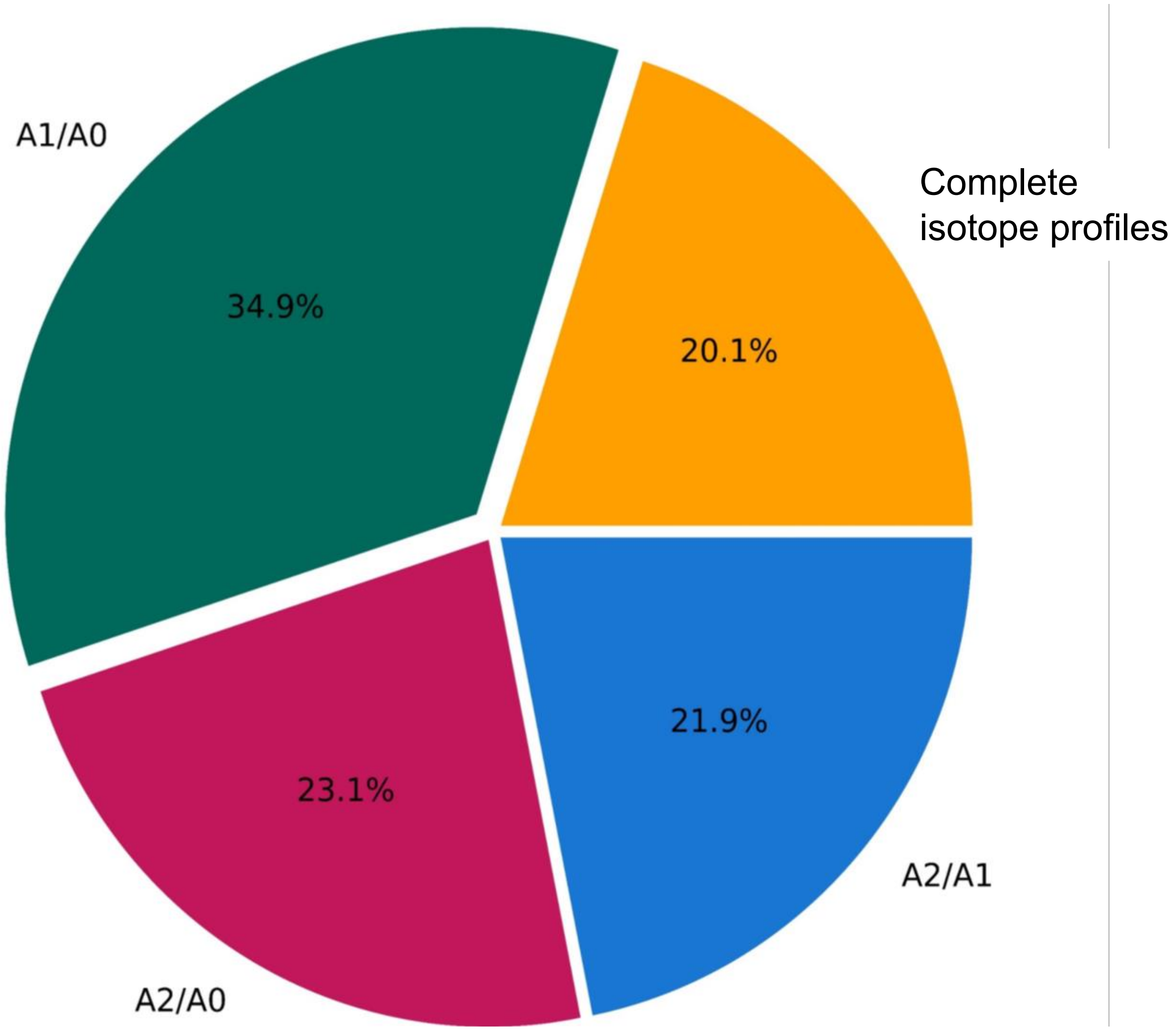

Supplementary Figure 11. The highest contribution to the combined best fit to the theoretical monoisotopic RIA was provided by A_1/A_0 ratio. The pie chart shows the percentages of peptides for which one of the four tested determinations of the monoisotopic RIA was closest to the theoretical RIA. Thus, in **Supplementary Figure 10 B**, 34.9% of RIA estimations come from the A_1/A_0 ratios. More than 15700 peptides were used in these calculations.

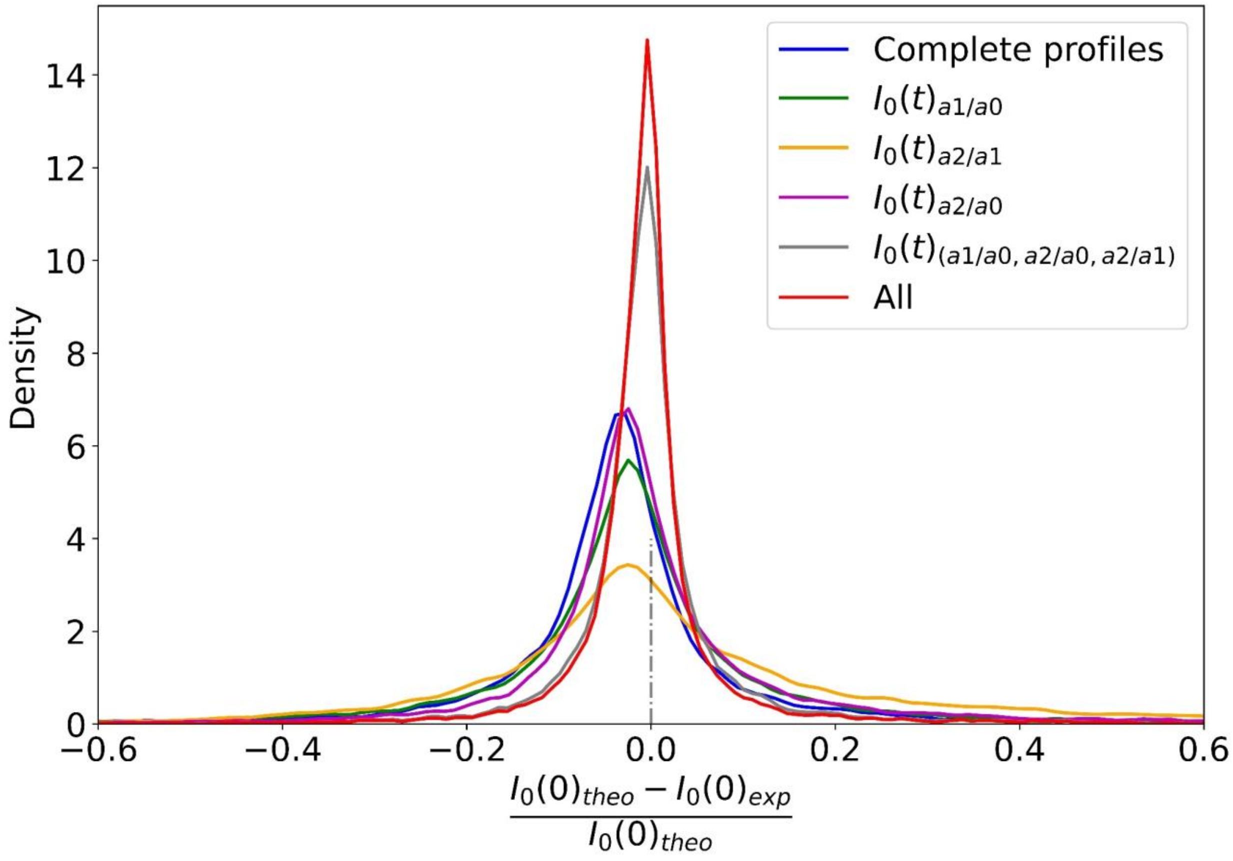

Supplementary Figure 12. The density plots of the estimations of the RIAs from complete isotope profiles and from ratios of pairs of two mass isotopomers. The shown data are from one experiment (before the start of the labeling). No filters were applied, and all identified (from MS/MS) peptides were used to generate the plots. For each peptide, its estimated RIA was transformed relative to the (expected) natural RIA value, $\left(\mathrm{I}_0(0)_{\mathrm{theo}} - \widetilde{\mathrm{I}_0(0)}\right)/\mathrm{I}_0(0)_{\mathrm{theo}}$ for the reconstructed (from the ratios) and $\left(\mathrm{I}_0(0)_{\mathrm{theo}} - \mathrm{I}_0(0)_{\mathrm{complete}}\right)/\mathrm{I}_0(0)_{\mathrm{theo}}$ for the complete isotope profiles. Overall, A_2/A_0 ratios produced slightly better results than the complete isotope profiles. Combining the improvements of the monoisotopic RIA estimations from all pairs of ratios reduced the width of the density function by more than twice. Shown are the results from all peptides of the unlabeled sample.

The ratios containing the second heavy mass isotopomer explained the monoisotopic RIAs of heavy peptides, with median masses of 1875 Da (for A_2/A_0 ratio) and 1821 Da (for A_2/A_1 ratio). The median absolute deviations (MAD) were 330 Da and 344 Da, respectively. The peptides in the A_1/A_0 ratio group had the smallest median mass, 1714 Da (MAD=314 Da). The median mass of the peptides, whose monoisotopic RIA was explained the best with the complete isotope profiles, was 1807 Da (MAD=360 Da).

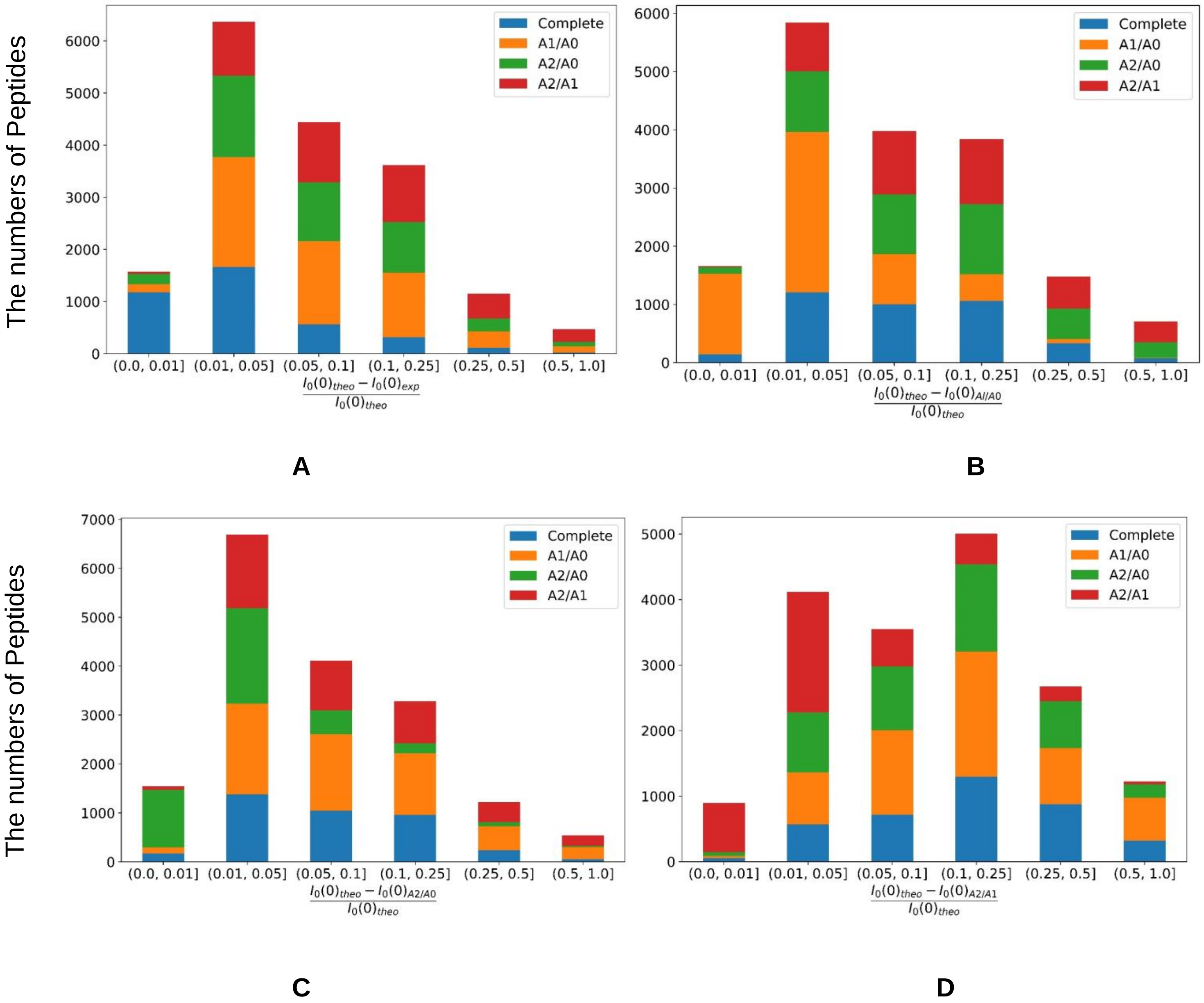

Supplementary Figure 13. The bar charts of the number of peptides (y-axis) and accuracy (x-axis) of the monoisotopic RIA estimation for each method: **A**) complete isotope profiles, **B**) A_1/A_0 ratio, **C**) A_2/A_0 ratio, and **D**) A_2/A_1 ratio. For the RIAs computed using complete isotope profiles (**A**), 1575 peptides were quantified to within 1% of the theoretical RIA, and 6365 peptides were quantified in between one and five percent of the corresponding theoretical RIAs. Among the latter peptides, 2110, 1565, and 1030 can also be explained to within the same (between one and five percent) accuracy by A_1/A_0, A_2/A_0, and A_2/A_1 ratios, respectively. Shown are the results from the unlabeled sample. No filtering was applied.

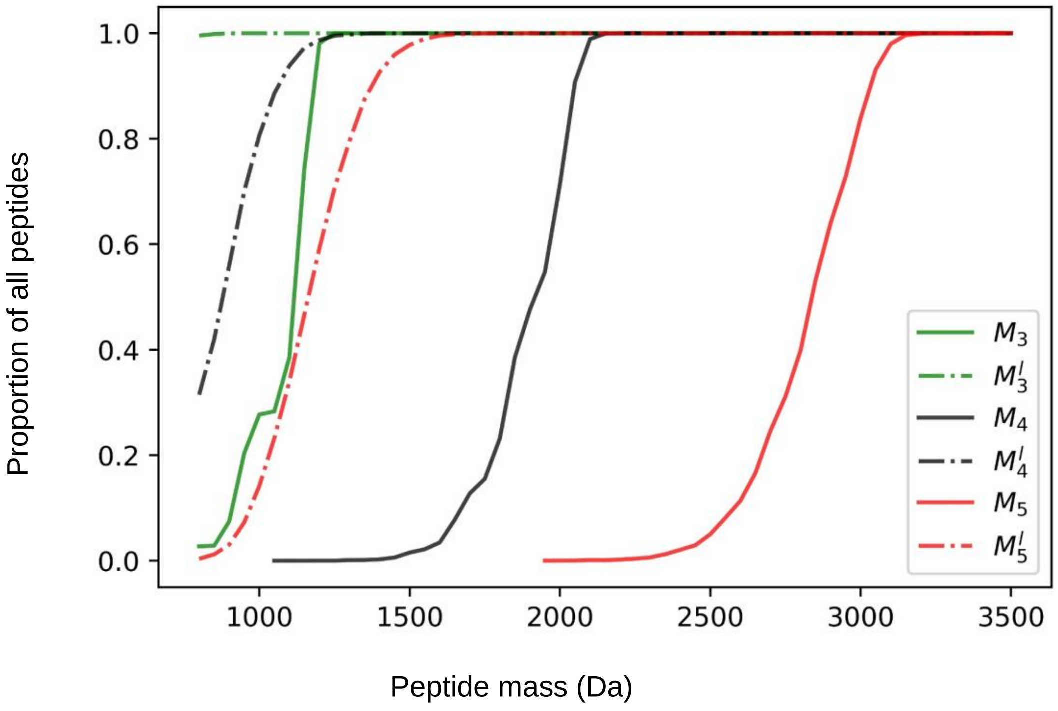

Supplementary Figure 14. For peptides labeled with 5% enriched heavy water, up to six mass isotopomers are important for computing the monoisotopic relative isotope abundance (RIA). The figure shows the relative proportions (y-axis) of peptides for which the RIA of the M_3 (green), M_4 (black), and M_5 (red) mass isotopomers are higher than 0.03 as a function of the peptide mass. The continuous lines show the proportions of natural peptides, and the dashed lines show the corresponding proportions of the labeled peptides. As seen from the figure, for more than half of peptides with masses of 1.3 kDa and higher, M_5 mass isotopomer is significant and can affect the accuracy of the estimation of the monoisotopic RIA.

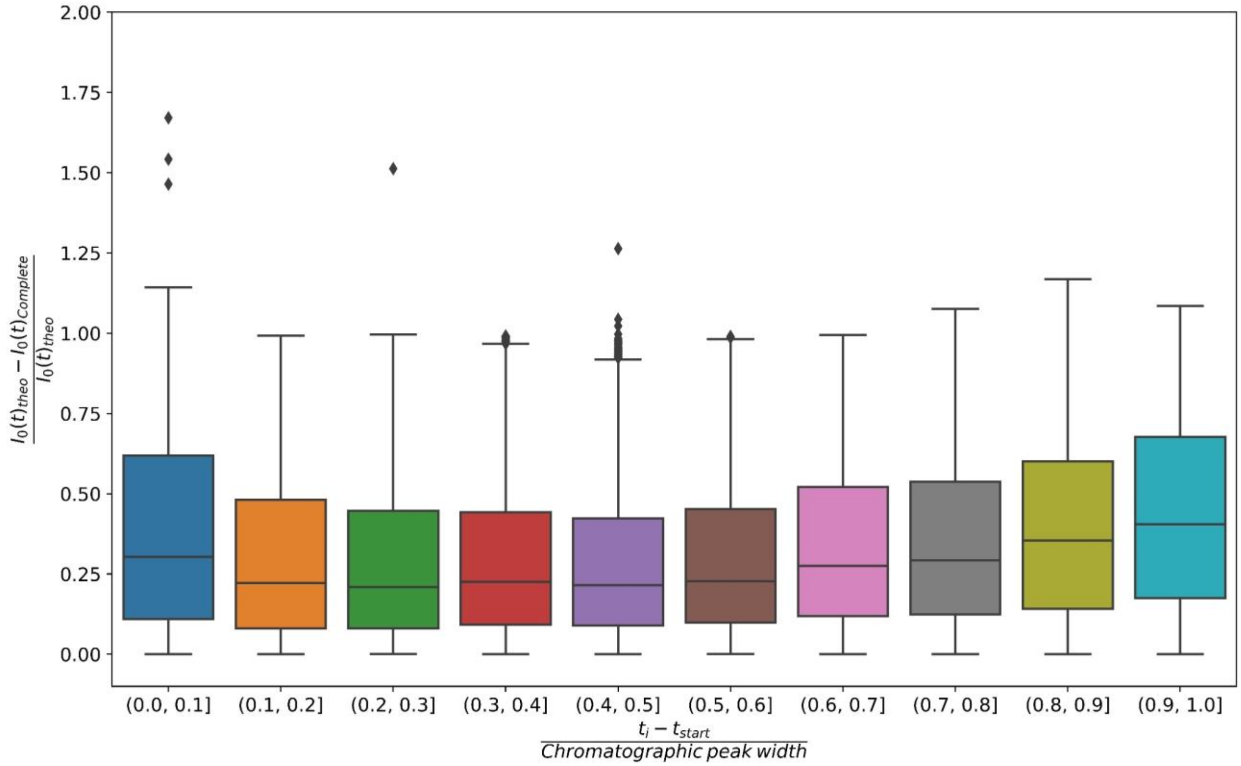

Supplementary Figure 15. The box plot of the relative isotope error distributions as a function of a position in chromatographic peak elution. The x-axis is the shifted elution (the difference between the elution time and the start of the elution) and normalized (by dividing by the relative chromatographic elution peak width) time binned into ten ranges. The y-axis is the absolute relative error of the monoisotopic RIA at the specific position in the elution profile. The intervals (0, 0.1] and (0.9, 1.0] correspond to the start and end of the elution, respectively. The intervals (0.4, 0.5] and (0.5, 0.6] are the apex of the elution. The figure shows the data for the peptides whose RIA estimation by the complete isotope profiles had more than 0.2 relative error. As seen from the box plot, the median of the errors was the smallest in the apex.

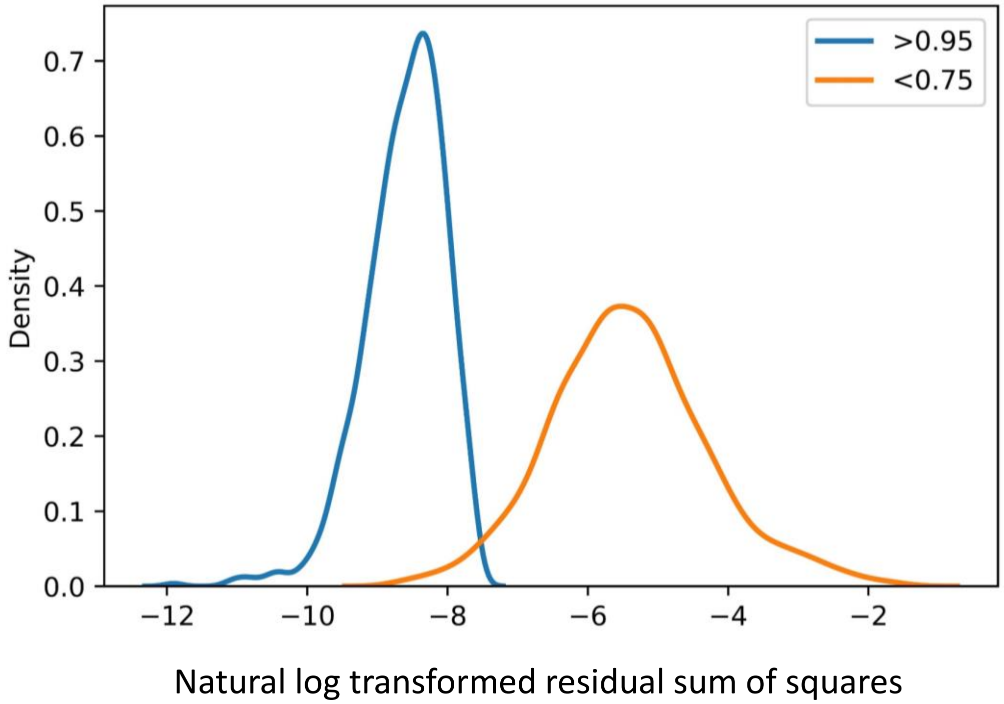

Supplementary Figure 16. The high goodness-of-fit measure (R^2) results from isotope profiles that have good spectral accuracy (as measured by the difference between predicted and observed monoisotopic RIAs). The figure shows the density (y-axis) plot of the natural log transformed residual sum of squares between predicted and experimental RIAs for high quality (blue line) and low quality (orange line) data points.

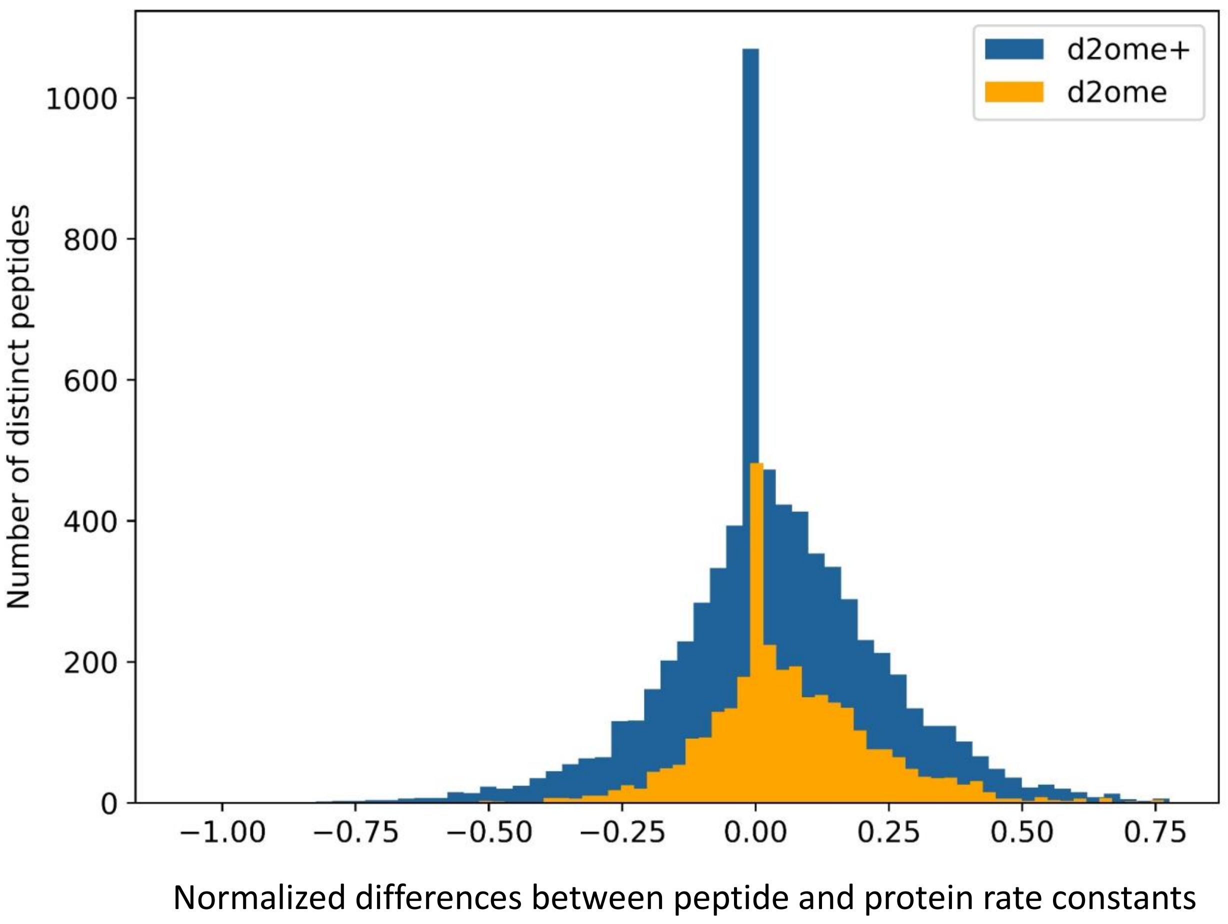

Supplementary Figure 17. The histograms of the differences between protein and peptide turnover rates before (yellow) and after (blue) the use of ratios for estimating label enrichments. As a test metric, the normalized difference between the protein, k_{prot}, and peptide, k_{pep}, turnover rates were used. The metric was $\left(k_{pep} - k_{prot}\right)/\sqrt{{k_{pep}}^2 + {k_{prot}}^2}$. 3333 (46%) more peptides passed the R^2 threshold after the use of the ratios.

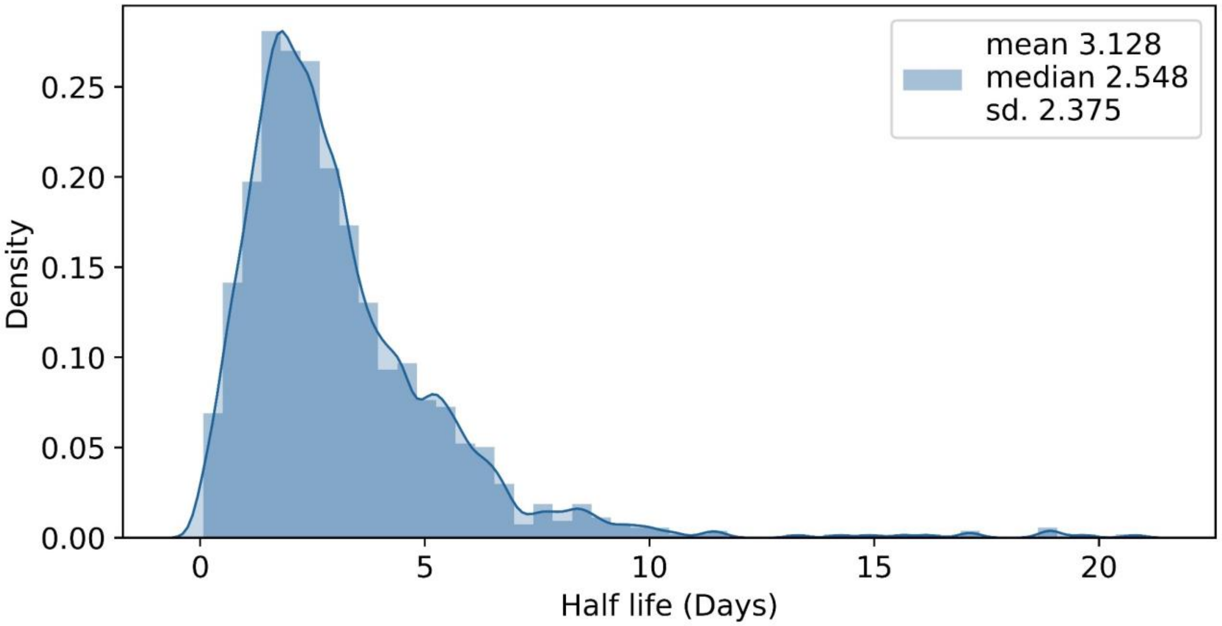

Supplementary Figure 18. The density distribution of murine liver protein half-life distribution. The mean and median of the distribution were 2.55 and 3.13 days, respectively.

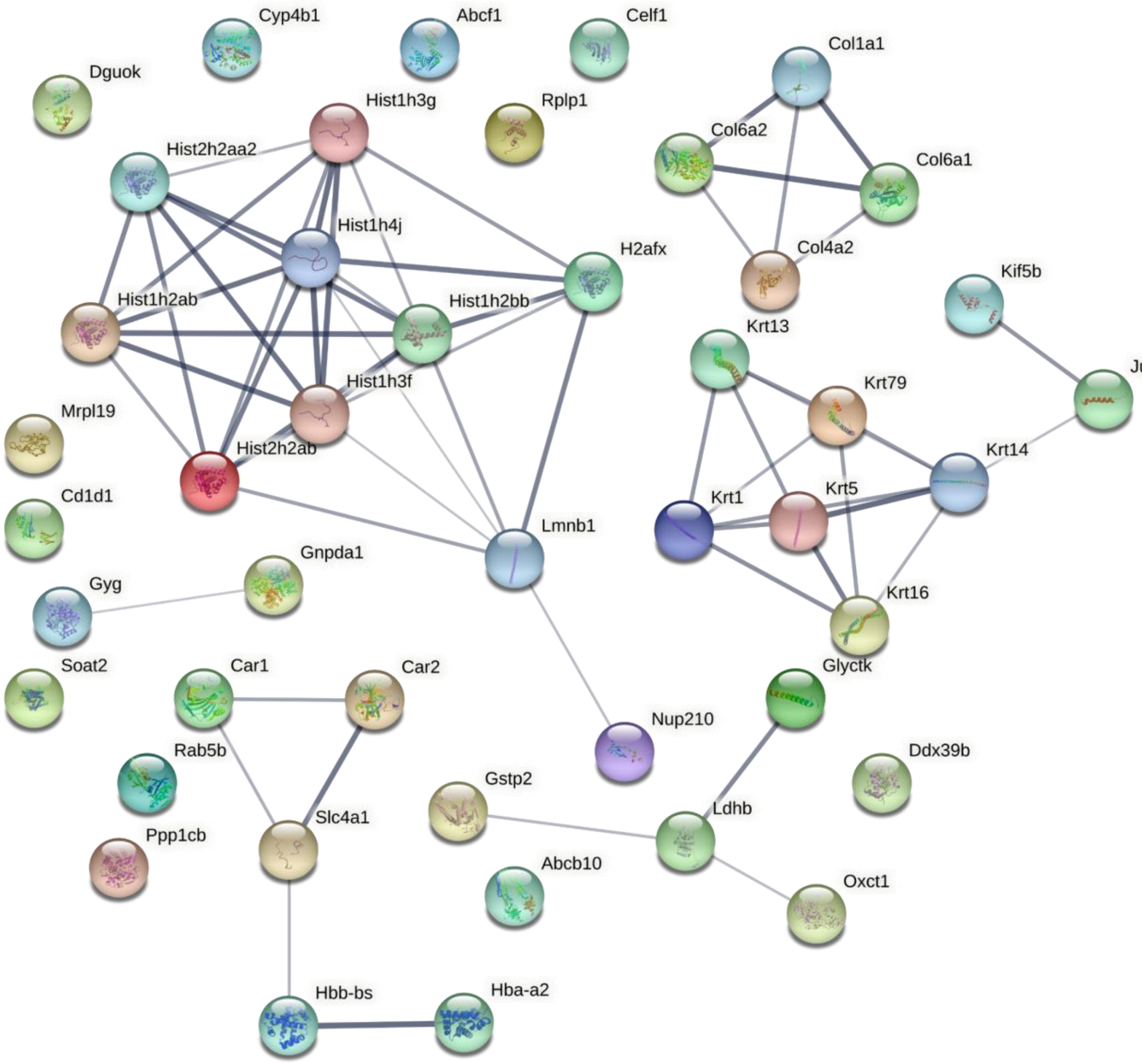

Supplementary Figure 19. The STRING[14] database generated protein network of slow turnover proteins in murine liver data set. The histones, collagens, cytoskeletal keratins, carbonic anhydrase and hemoglobins formed local subclusters.

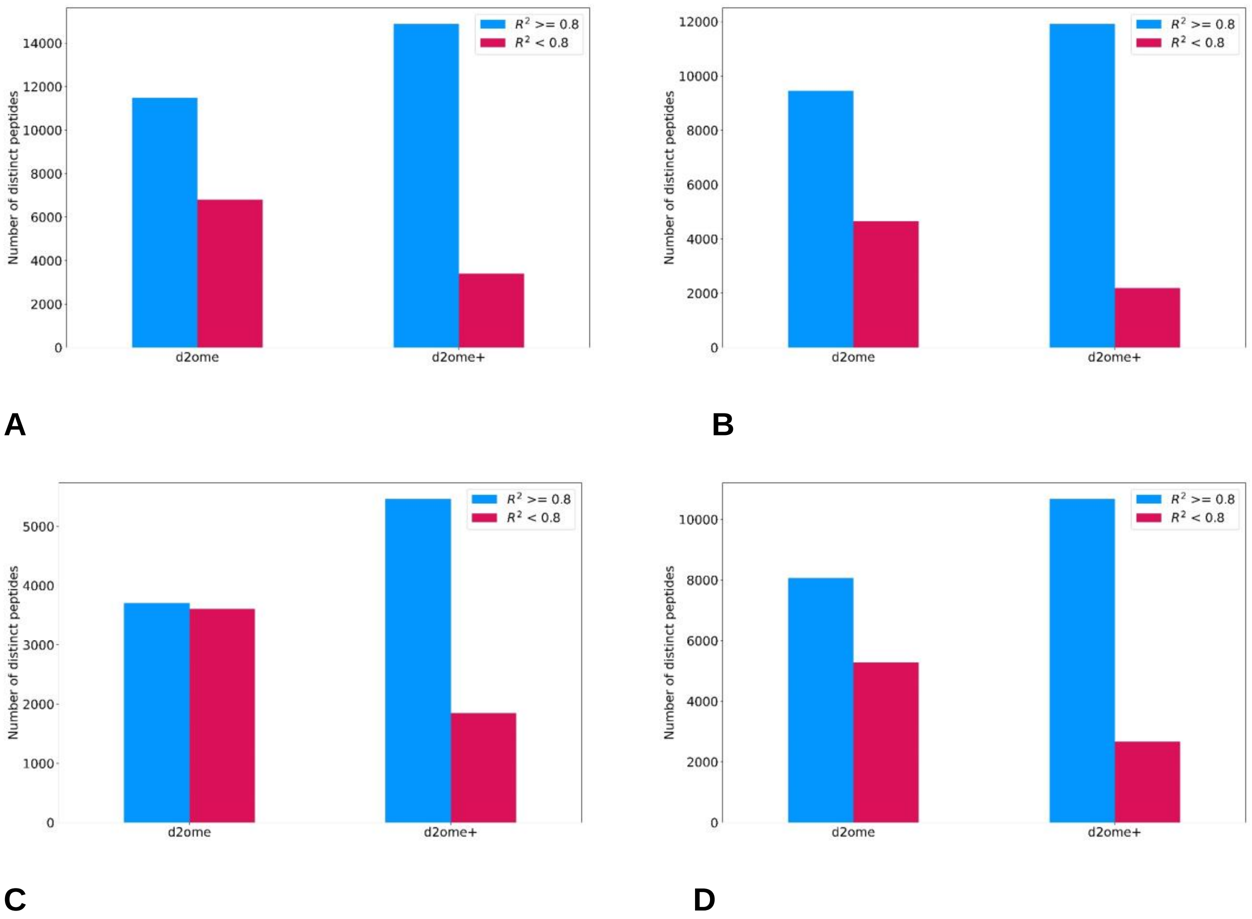

Supplementary Figure 20. d2ome+ improves the goodness-of-fit measure (R^2) for protein turnover rate estimations from four murine tissues data acquired using Orbitrap Q Exactive HF mass spectrometer. Results for **A**. Liver proteome. **B**. Murine kidney proteome. **C**. Murine muscle proteome. **D**. Murine heart proteome. For all tissues there was increase in the number of peptides with improved $R^2 \geq 0.8$. The number of low-quality fits ($R^2 < 0.8$) was reduced by more two times.

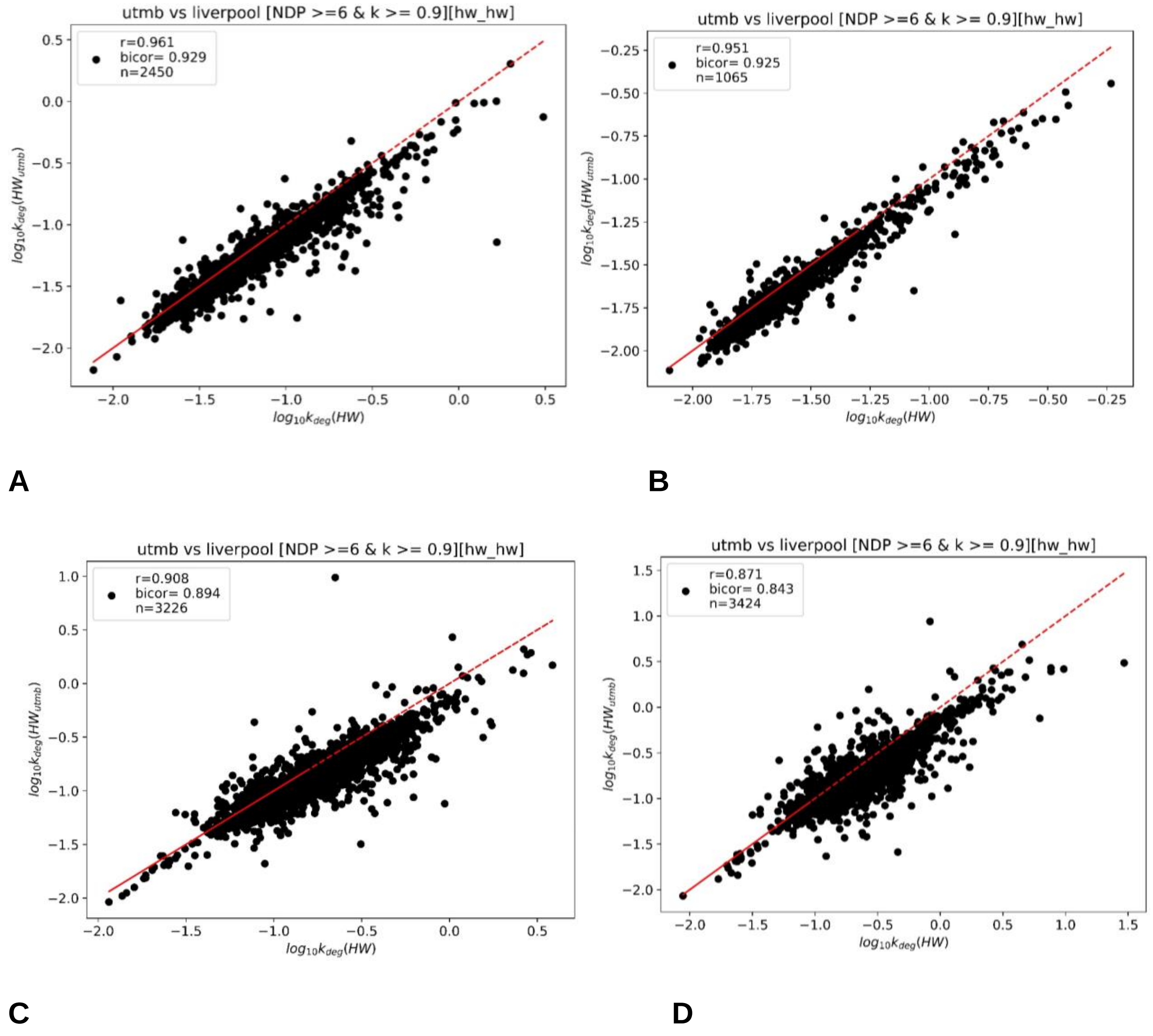

Supplementary Figure 21. Peptide turnover rates reported in a recent study[1] agreed well with the calculations using d2ome+. **A)** Muscle proteome, Spearman correlation was 0.96; **B)** Heart proteome, Spearman correlation was 0.95; **C)** Kidney proteome, Spearman correlation was 0.91; **D)** Liver proteome, Spearman correlation was 0.87. The red line is the unity line. Shown is the scatter plot of the base-10 log transformed peptide turnover rates (day^{-1}): x-axis are the results from d2ome+, y-axis are the results from Hammond et. al[1].

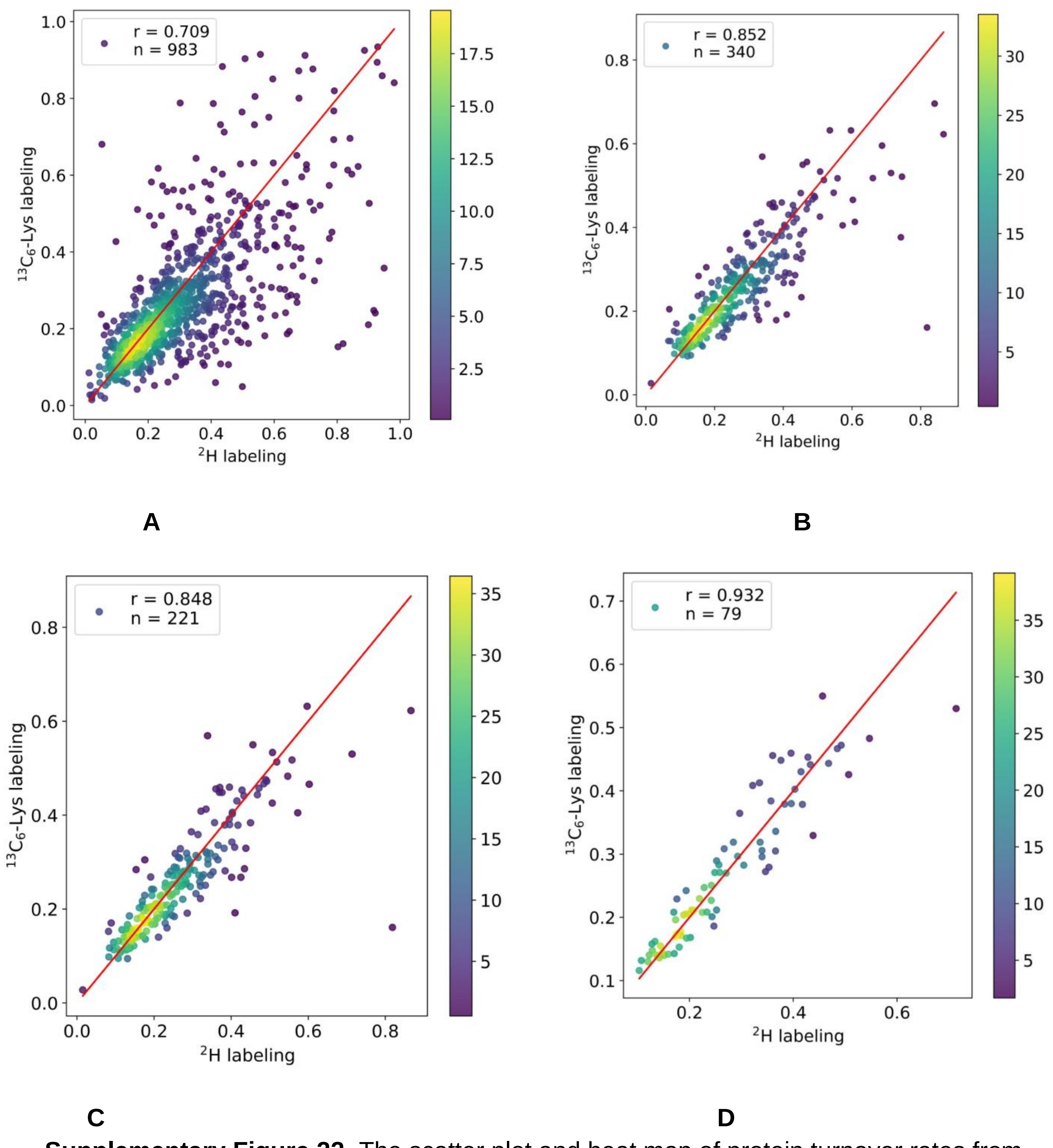

Supplementary Figure 22. The scatter plot and heat map of protein turnover rates from heavy water (x-axis) and $^{13}C_6$-Lys labelings. A) proteins in the [0.01, 1.0] day-1 turnover rate range, all (983) common proteins, B), C), D) proteins quantified by at least three (340), five (221), and ten (79) unique peptides. As the number of quantified peptides increased, the correlations between the turnover rates increased.

Supplementary Table 1. The numbers of peptides (murine liver proteome) that improved the monoisotopic relative isotope abundance (RIA) estimations from the ratio of abundances of two mass isotopomers[a].

	[1]N_0	[2]N_1	[3]N_2	[4]N_3	[5]N_4	[6]N_5
$10^{10} \leq A_0$	150	143	3	0	2	0
$10^{9} \leq A_0 < 10^{10}$	1815	1538	98	25	77	3
$10^{8} \leq A_0 < 10^{9}$	7015	4526	805	282	557	73
$10^{7} \leq A_0 < 10^{8}$	9858	4005	1725	973	919	384
$10^{6} \leq A_0 < 10^{7}$	3871	972	767	982	256	419
$A_0 < 10^{6}$	200	35	68	46	12	18

[a] A_0 – the raw abundance of the monoisotope.

[1]N_0 - the number of all unique peptides (amino acid sequence, charge state, and modification)

[2]N_1 - the number of all unique peptides, whose relative monoisotopic error estimation using complete isotope profile was less than 0.08, $\frac{\mathrm{abs}(I_0(0)_{\mathrm{theo}} - I_0(0)_{\mathrm{complete\_isotopes}})}{I_0(0)_{\mathrm{theo}}} \leq 0.08$.

[3]N_2 - the number of all unique peptides, whose relative monoisotopic error estimation using complete isotope profiles was larger than 0.2, $\frac{(I_0(0)_{\mathrm{theo}} - I_0(0)_{\mathrm{complete\_isotopes}})}{I_0(0)_{\mathrm{theo}}} \geq 0.2$.

[4]N_3 - the number of all unique peptides, whose relative monoisotopic error estimation using complete isotope profiles was less than -0.2, $\frac{(I_0(0)_{\mathrm{theo}} - I_0(0)_{\mathrm{complete\_isotopes}})}{I_0(0)_{\mathrm{theo}}} \leq -0.2$.

[5]N_4 - the number of all unique peptides, whose relative monoisotopic error estimation using complete isotope profiles was larger than 0.2 and became less than 0.08 (absolute value), after estimation by the ratio of two mass isotopomers. The percentage of peptides with improved estimations was 53 %.

[6]N_5 - the number of all unique peptides, whose relative monoisotopic error estimation using complete isotope profiles was less than -0.2 and became less than 0.08 (absolute value), after estimation by the ratio of two mass isotopomers. For 39% of all peptides in category the estimations of the monoisotopic RIA by the new method improved.

Supplementary Table 2. The analysis of the performance of the partial isotope profile approach with the mass accuracy of peptide features (murine liver proteome). As the mass accuracy decreases, the number of quantified peptide features increases while the percentage of the features accurately reproducing the monoisotopic RIA decreases. The number of peptides for which the partial isotope profiles improved the RIA estimations at lower mass accuracy increased, which indicates that the decrease in the mass accuracy mostly affected the higher mass isotopomers. The chromatographic elution time window was fixed at 1 minute. The presented data is obtained from the analysis of an unlabeled sample, 220min_OT60it_26Aug21_RSL_01. An unlabeled sample was used, as it allowed the comparison of the computed RIA with the natural RIA.

Mass Accuracy (ppm)	Number of peptides	Percent peptides with relative error of RIA less than 0.08	Percent peptides with relative error of RIA larger than 0.2	Percent peptides with relative error of RIA larger than 0.2 and partial isotope RIA error better than 0.08
10	20484	55.0	18.1	43.6
20	22909	48.9	25.2	47.1
30	21850	47.6	28.2	48.3
40	22099	45.6	31.4	48.7
50	22270	43.6	34.0	48.6

Supplementary Table 3. The analysis of the performance of the RIA estimations by traditional and alternative approaches with the chromatographic elution window (murine liver proteome). As the chromatographic elution window increases, the number of accurately quantified peptide features (by both methods) decreases. The mass accuracy was fixed at 20 ppm. The presented data is obtained from the analysis of an unlabeled sample, 220min_OT60it_26Aug21_RSL_01. An unlabeled sample was used, as it allowed the comparison of the computed RIA with the natural RIA.

Chromatographic elution window (minutes)	Number of peptides	Percent peptides with the relative error of RIA less than 0.08	Percent peptides with relative error of RIA larger than 0.2	Percent peptides with relative error of RIA larger than 0.2 and partial isotope RIA error better than 0.08
1	22909	48.9	25.2	47.1
2	23091	48.1	26.7	44.6
3	23197	47.5	27.9	42.0
4	23291	47.0	28.8	40.5
5	23354	46.5	29.3	39.3

Supplementary Table 4. The accuracy of the monoisotopic RIA estimations in an unlabeled fractionated sample (murine liver proteome). The mass accuracy and elution time window were fixed at 20 ppm and 1 minute, respectively. For each of the fractionated samples, the percentage of unique peptide features accurately quantified by complete isotope profiles was higher than that from the unfractionated data. The percentage was the highest for the fraction with the least number of peptides, the least complex Fraction 1. The number of unique peptide features from all fractionated samples was more than twice higher than from the unfractionated sample.

Sample	Number of peptides	Percent peptides with relative error of RIA less than 0.08	Percent peptides with relative error of RIA larger than 0.2	Percent peptides with relative error of RIA larger than 0.2 and partial isotope RIA error better than 0.08
Unfractionated	20484	48.9	25.2	47.1
Fraction 1	5388	53.2	23.3	50.9
Fraction 2	11271	51.5	25.1	48.7
Fraction 3	12510	52.0	21.9	47.1
Fraction 4	13601	50.8	24.2	48.0
Fraction 5	13686	50.4	23.9	45.0
Fraction 6	12623	49.4	24.0	46.6
Fraction 7	12684	49.5	24.3	47.5
Fraction 8	11685	49.7	23.2	44.0
Eight fractions combined	51142	56.4	20.8	45.3

Supplementary Table 5. The numbers of peptides and proteins (Orbitrap Eclipse data set) for several values of the coefficient of determination, R^2. All distinct peptides were required to be quantified at least four times.

	Complete Isotope Profiles			Partial Isotope Profiles		
	Peptides[a]	Proteins[b]	Peptides from slow turnover proteins[c]	Peptides[a]	Proteins[b]	Peptides from slow turnover proteins[c]
$R^2 \geq 0.95$	3106	943	160	8402	1693	225
$R^2 \geq 0.90$	6043	1418	160	11543	1935	225
$R^2 \geq 0.80$	9016	1769	160	14367	2108	
$R^2 \geq 0.75$	10010			15200		
$R^2 < 0.75$	12638			7448		

[a]Shown are the numbers of the peptides that passed the specified R^2 thresholds in the approaches using complete and partial isotope profiles. For example, the numbers of peptides that had $R^2 \geq 0.9$ in the complete and partial isotopes profiles were 6043 and 11543, respectively.

[b]Shown are the numbers of the proteins that had at least one peptide with the specified R^2 thresholds. For example, the numbers of proteins that had at least one peptide with $R^2 \geq 0.9$ in the complete and partial isotopes profiles were 1418 and 1935, respectively.

[c]Shown are the numbers of the peptide that passed the specified R^2 thresholds or had the standard error of theoretical fit (≤ 0.05), if the rate is smaller than 0.01 day^{-1}.

Supplementary References

(1) Hammond, D. E.; Simpson, D. M.; Franco, C.; Wright Muelas, M.; Waters, J.; Ludwig, R. W.; Prescott, M. C.; Hurst, J. L.; Beynon, R. J.; Lau, E. Harmonizing Labeling and Analytical Strategies to Obtain Protein Turnover Rates in Intact Adult Animals. *Mol Cell Proteomics* **2022**, *21* (7), 100252. DOI: 10.1016/j.mcpro.2022.100252.

(2) Sadygov, R. G. Protein turnover models for LC-MS data of heavy water metabolic labeling. *Brief Bioinform* **2022**, *23* (2). DOI: 10.1093/bib/bbab598.

(3) Feller, W. *An Introduction to Probability Theory and Its Applications*; John Wiley & Sons, 1971.

(4) Kvalseth, T. O. Cautionary Note about R². *The American Statistician* **1985**, *39* (4), 279-285. DOI: 10.2307/2683704 (acccessed 2022/07/27/).JSTOR.

(5) Casella, G.; Berger, R. L. *Statistical inference*; Thomson Learning, 2002.

(6) Sadygov, V. R.; Zhang, W.; Sadygov, R. G. Timepoint Selection Strategy for In Vivo Proteome Dynamics from Heavy Water Metabolic Labeling and LC-MS. *J Proteome Res* **2020**, *19* (5), 2105-2112. DOI: 10.1021/acs.jproteome.0c00023.

(7) Jameson, T. S. O.; Kilroe, S. P.; Fulford, J.; Abdelrahman, D. R.; Murton, A. J.; Dirks, M. L.; Stephens, F. B.; Wall, B. T. Muscle damaging eccentric exercise attenuates disuse-induced declines in daily myofibrillar protein synthesis and transiently prevents muscle atrophy in healthy men. *Am J Physiol Endocrinol Metab* **2021**, *321* (5), E674-E688. DOI: 10.1152/ajpendo.00294.2021.

(8) Kessner, D.; Chambers, M.; Burke, R.; Agus, D.; Mallick, P. ProteoWizard: open source software for rapid proteomics tools development. *Bioinformatics* **2008**, *24* (21), 2534-2536.

(9) Perkins, D. N.; Pappin, D. J.; Creasy, D. M.; Cottrell, J. S. Probability-based protein identification by searching sequence databases using mass spectrometry data. *Electrophoresis* **1999**, *20* (18), 3551-3567.

(10) Martens, L.; Chambers, M.; Sturm, M.; Kessner, D.; Levander, F.; Shofstahl, J.; Tang, W. H.; Rompp, A.; Neumann, S.; Pizarro, A. D.; et al. mzML--a community standard for mass spectrometry data. *Mol Cell Proteomics* **2011**, *10* (1), R110 000133. DOI: 10.1074/mcp.R110.000133.

(11) Jones, A. R.; Eisenacher, M.; Mayer, G.; Kohlbacher, O.; Siepen, J.; Hubbard, S. J.; Selley, J. N.; Searle, B. C.; Shofstahl, J.; Seymour, S. L.; et al. The mzIdentML data standard for mass spectrometry-based proteomics results. *Mol Cell Proteomics* **2012**, *11* (7), M111 014381. DOI: 10.1074/mcp.M111.014381.

(12) Sadygov, R. G.; Avva, J.; Rahman, M.; Lee, K.; Ilchenko, S.; Kasumov, T.; Borzou, A. Correction to "d2ome, Software for in Vivo Protein Turnover Analysis Using Heavy Water Labeling and LC-MS, Reveals Alterations of Hepatic Proteome Dynamics in a Mouse Model of NAFLD". *J Proteome Res* **2021**, *20* (10), 4912. DOI: 10.1021/acs.jproteome.1c00698.

(13) Sadygov, R. G.; Avva, J.; Rahman, M.; Lee, K.; Ilchenko, S.; Kasumov, T.; Borzou, A. d2ome, Software for in Vivo Protein Turnover Analysis Using Heavy Water Labeling and LC-MS, Reveals Alterations of Hepatic Proteome Dynamics in a Mouse Model of NAFLD. *J Proteome Res* **2018**, *17* (11), 3740-3748. DOI: 10.1021/acs.jproteome.8b00417.

(14) Szklarczyk, D.; Morris, J. H.; Cook, H.; Kuhn, M.; Wyder, S.; Simonovic, M.; Santos, A.; Doncheva, N. T.; Roth, A.; Bork, P.; et al. The STRING database in 2017: quality-controlled protein-protein association networks, made broadly accessible. *Nucleic Acids Res* **2017**, *45* (D1), D362-D368. DOI: 10.1093/nar/gkw937.
