# Supplementary material for: Quantifying label enrichment from two mass isotopomers increases proteome coverage for in vivo protein turnover using heavy water metabolic labeling
Source: Commun Chem. 2023 Apr 17;6:72. doi: 10.1038/s42004-023-00873-x (PMC10110577; doi:10.1038/s42004-023-00873-x)
Supplement: Supplementary file 2 — Description of Additional Supplementary File [file 42004_2023_873_MOESM2_ESM.pdf]

Description of Additional Supplementary Files

File Name: Supplementary Data 1

Description: Examples of time series data of peptides which improved the coefficient of determination after the use of two mass isotopomers to compute label enrichment. The black circles are the original monoisotopic relative isotope abundances (RIAs) computed using complete isotope profiles. The blue crosses are the estimates of the RIAs from abundances of two mass isotopomers.

File Name: Supplementary Data 2

Description: The peak detection and quantification results for all peptides from an unlabelled sample, 220min_OT60it_26Aug21_RSL_01.raw.

File Name: Supplementary Data 3

Description: Turnover rates, confidence intervals of all proteins and their peptides from both Orbitrap Eclipse and Q Exactive datasets. Presented are the turnover rates from murine liver, kidney, heart, and muscle tissues.

File Name: Supplementary Data 4

Description: The file shows the labelling time course data and theoretical fits for peptides of slow turnover proteins. The black circles are the original monoisotopic relative isotope abundances (RIAs).

File Name: Supplementary Data 5

Description: The labelling time courses of peptides of the contaminant proteins. The black circles are the original monoisotopic relative isotope abundances (RIAs).

File Name: Supplementary Data 6

Description: Wilcoxon rank sum test results for protein complexes shown on **Figure 7** of the main text.
