# Supplementary material for: Quantifying label enrichment from two mass isotopomers increases proteome coverage for in vivo protein turnover using heavy water metabolic labeling
Source: Commun Chem. 2023 Apr 17;6:72. doi: 10.1038/s42004-023-00873-x (PMC10110577; doi:10.1038/s42004-023-00873-x)
Supplement: Supplementary file 3 — Supplementary Data 1 [file 42004_2023_873_MOESM3_ESM.pdf]

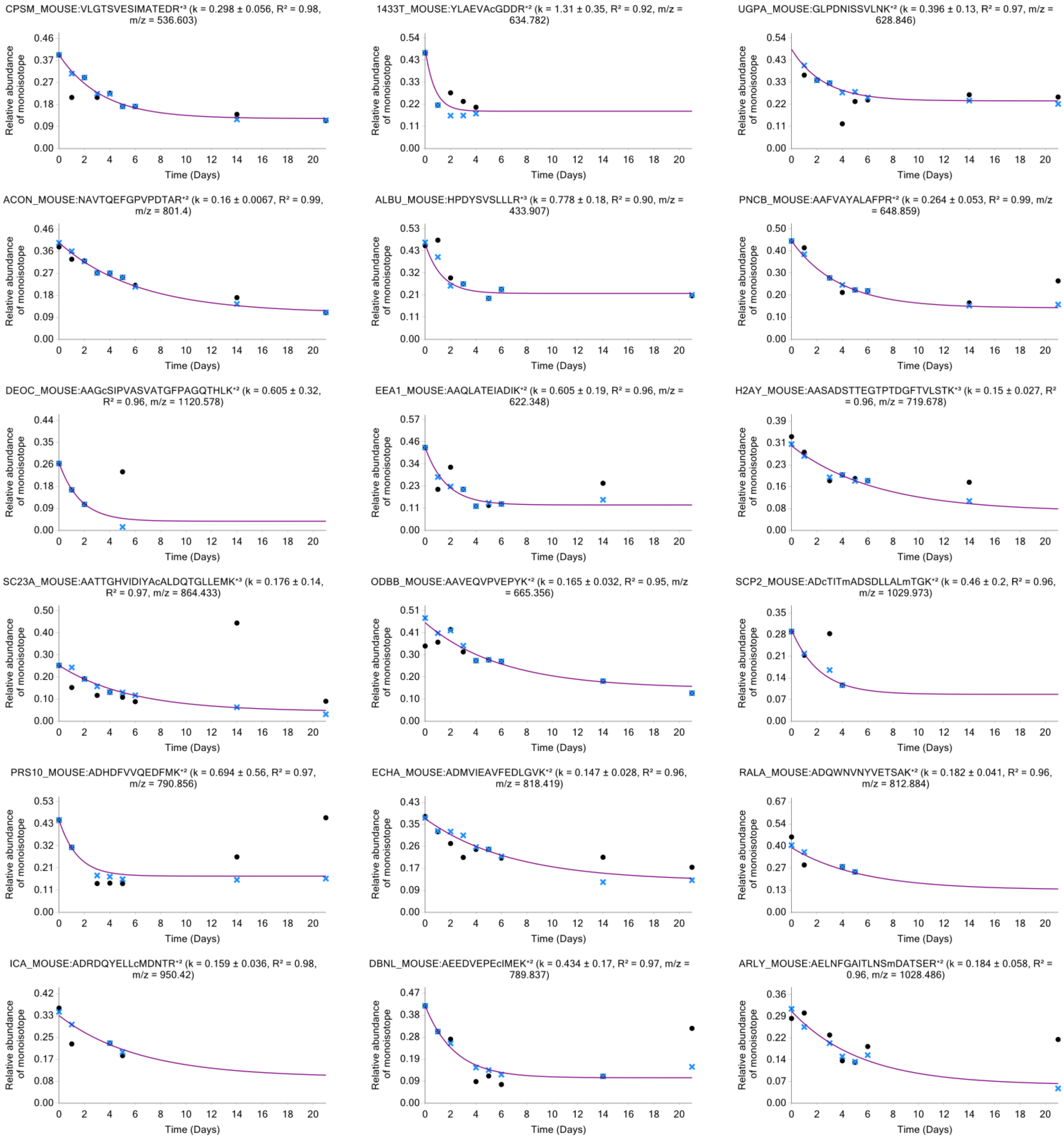

The Figures show the monoisotopic RIAs estimated from the complete isotope profile (black circles), RIA estimates from the complete isotope profiles, and any of the three partial isotope profiles combined ($A_1(t)/A_0(t)$, $A_2(t)/A_0(t)$, $A_2(t)/A_1(t)$) (blue crosses). The solid line shows the fit from the label enrichment determination technique.

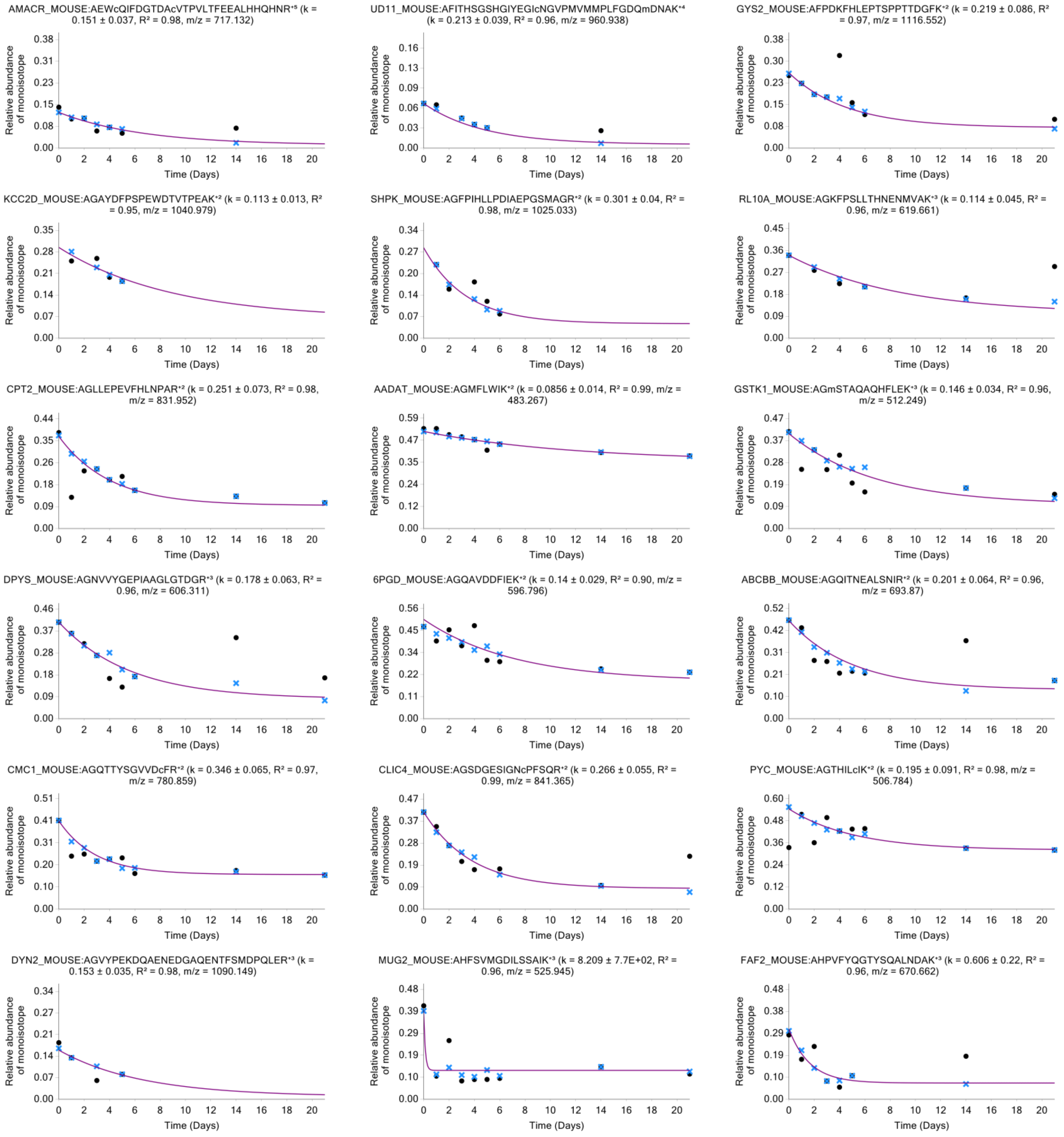

The Figures show the monoisotopic RIAs estimated from the complete isotope profile (black circles), RIA estimates from the complete isotope profiles, and any of the three partial isotope profiles combined ($A_1(t)/A_0(t)$, $A_2(t)/A_0(t)$, $A_2(t)/A_1(t)$) (blue crosses). The solid line shows the fit from the label enrichment determination technique.

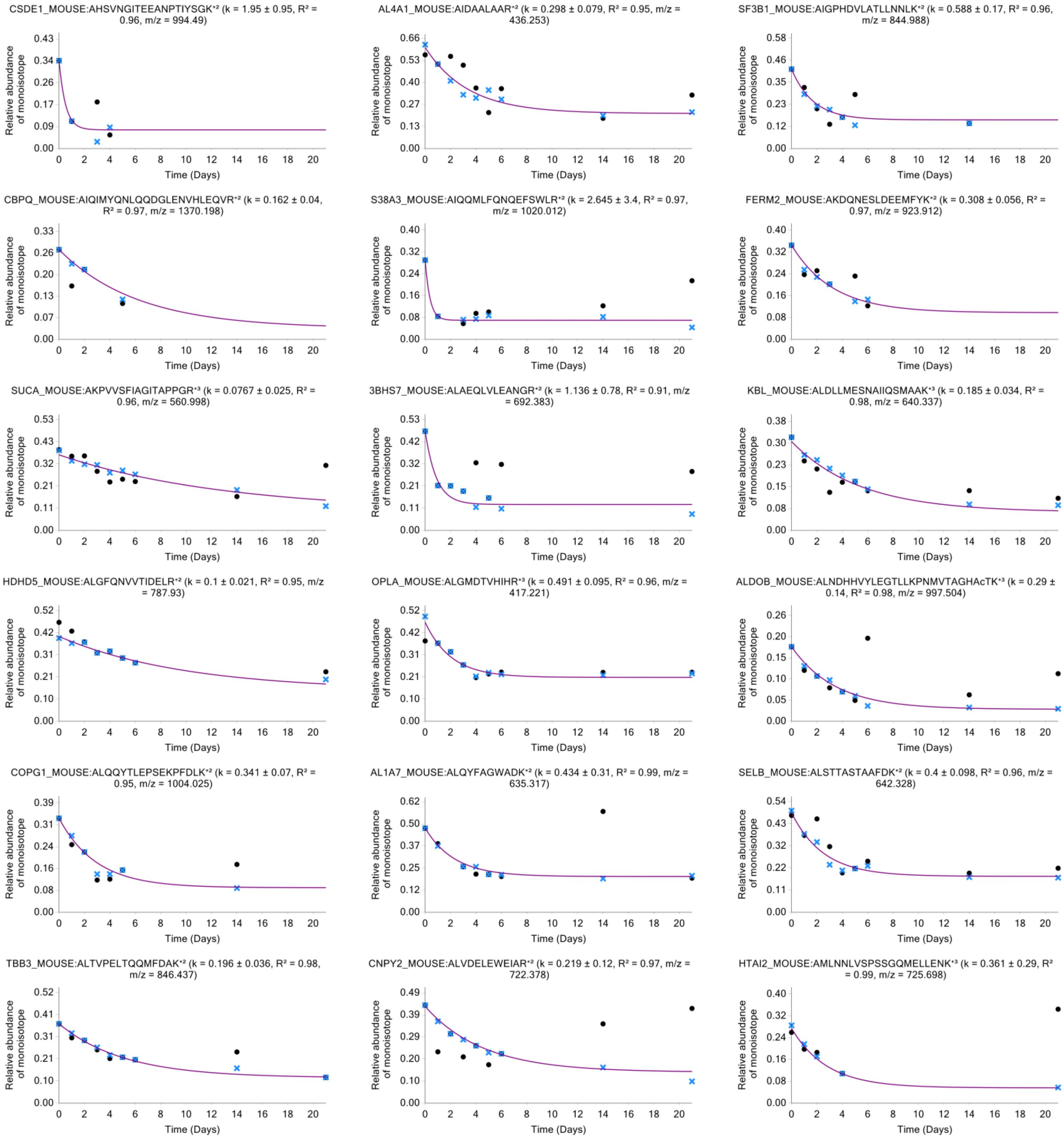

The Figures show the monoisotopic RIAs estimated from the complete isotope profile (black circles), RIA estimates from the complete isotope profiles, and any of the three partial isotope profiles combined ($A_1(t)/A_0(t)$, $A_2(t)/A_0(t)$, $A_2(t)/A_1(t)$) (blue crosses). The solid line shows the fit from the label enrichment determination technique.

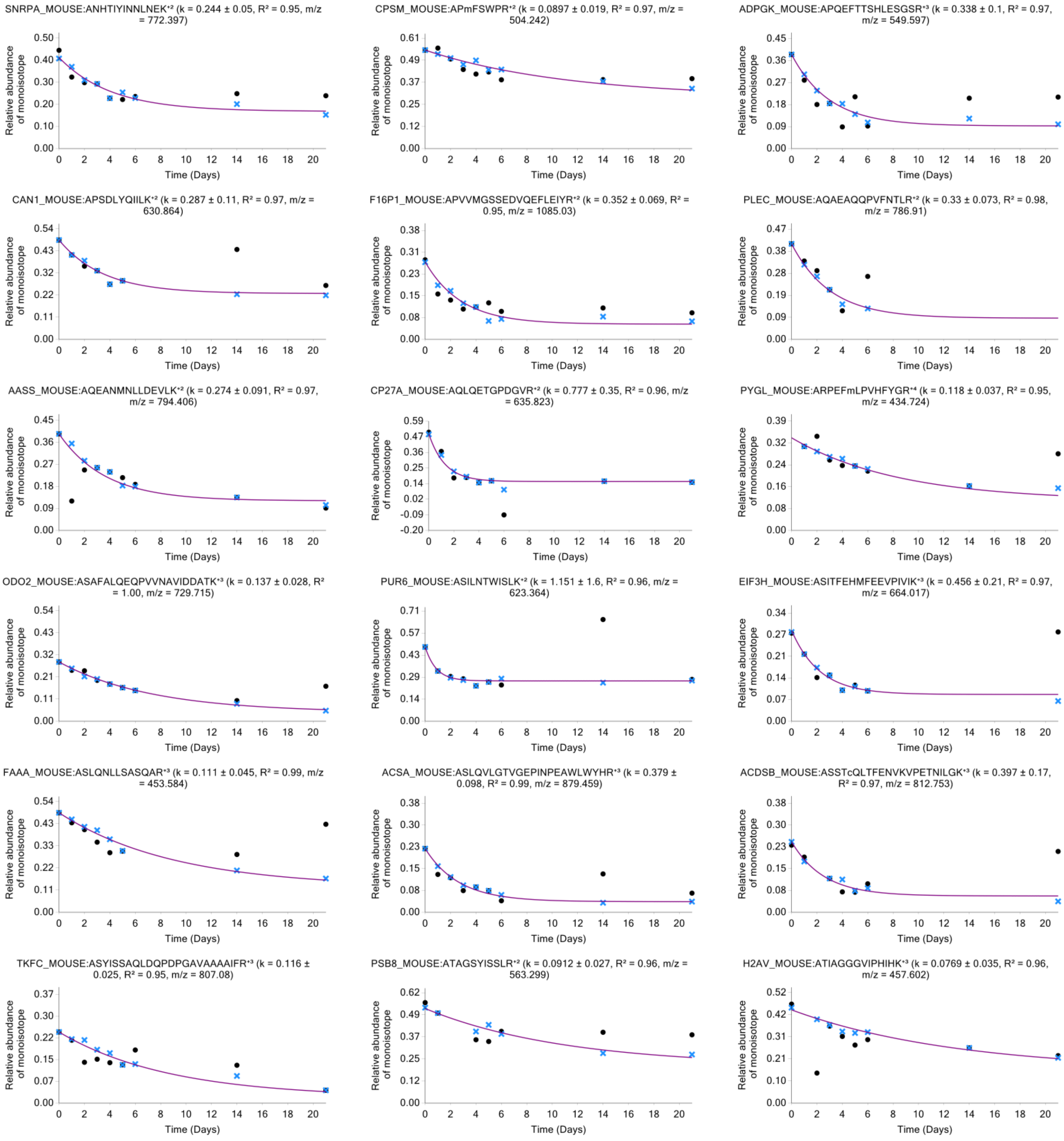

The Figures show the monoisotopic RIAs estimated from the complete isotope profile (black circles), RIA estimates from the complete isotope profiles, and any of the three partial isotope profiles combined ($A_1(t)/A_0(t)$, $A_2(t)/A_0(t)$, $A_2(t)/A_1(t)$) (blue crosses). The solid line shows the fit from the label enrichment determination technique.

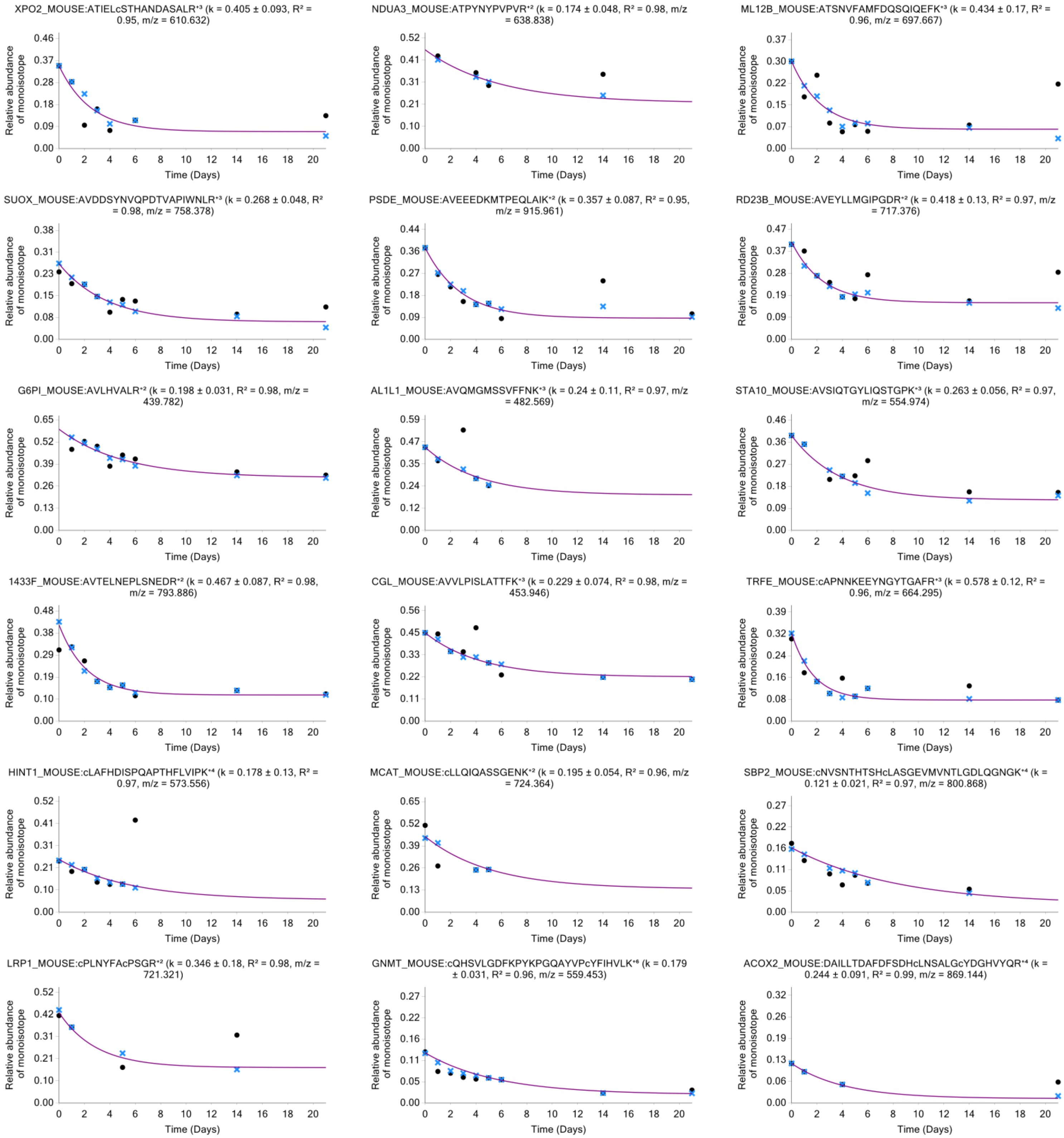

The Figures show the monoisotopic RIAs estimated from the complete isotope profile (black circles), RIA estimates from the complete isotope profiles, and any of the three partial isotope profiles combined ($A_1(t)/A_0(t)$, $A_2(t)/A_0(t)$, $A_2(t)/A_1(t)$) (blue crosses). The solid line shows the fit from the label enrichment determination technique.

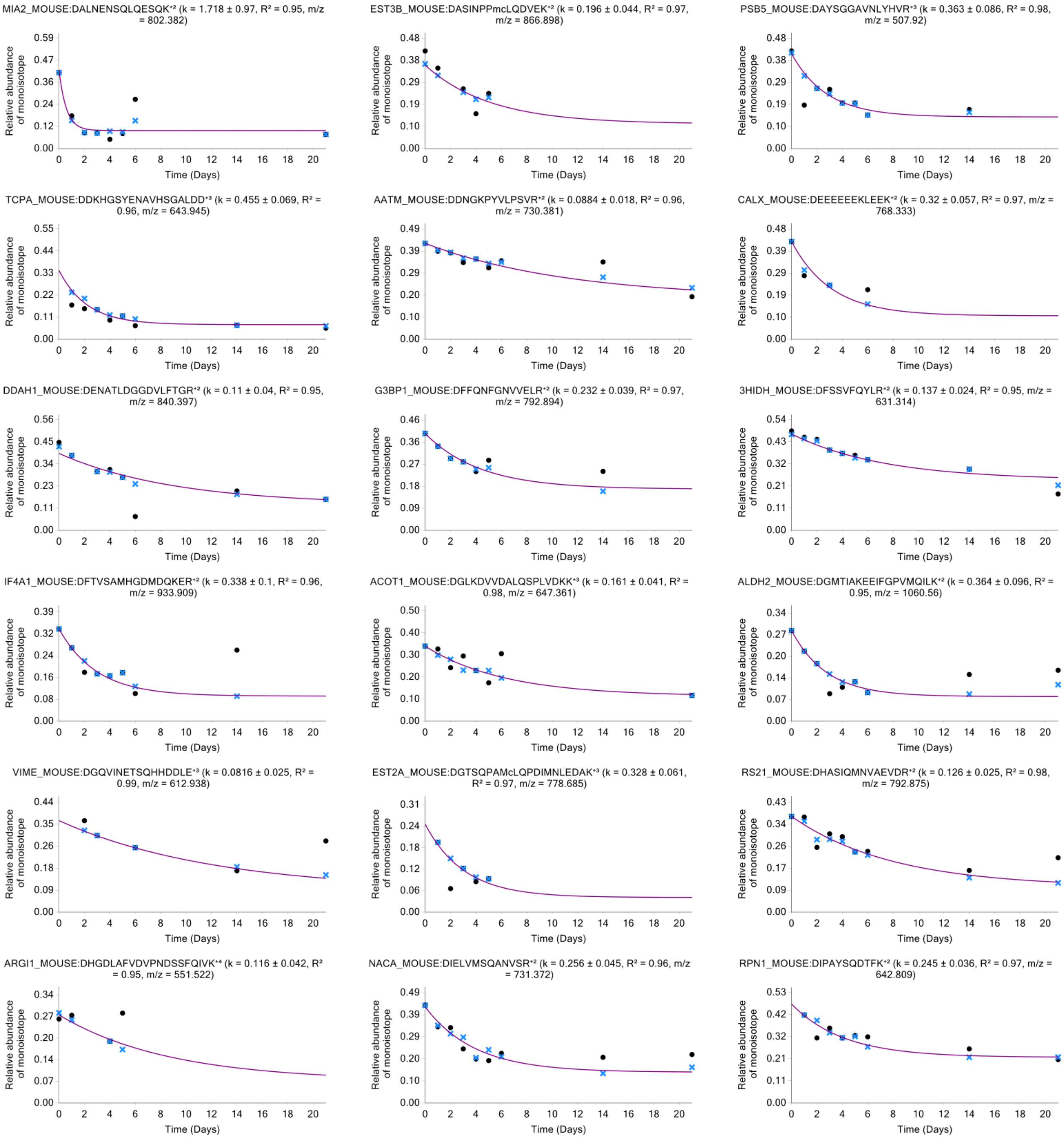

The Figures show the monoisotopic RIAs estimated from the complete isotope profile (black circles), RIA estimates from the complete isotope profiles, and any of the three partial isotope profiles combined ($A_1(t)/A_0(t)$, $A_2(t)/A_0(t)$, $A_2(t)/A_1(t)$) (blue crosses). The solid line shows the fit from the label enrichment determination technique.

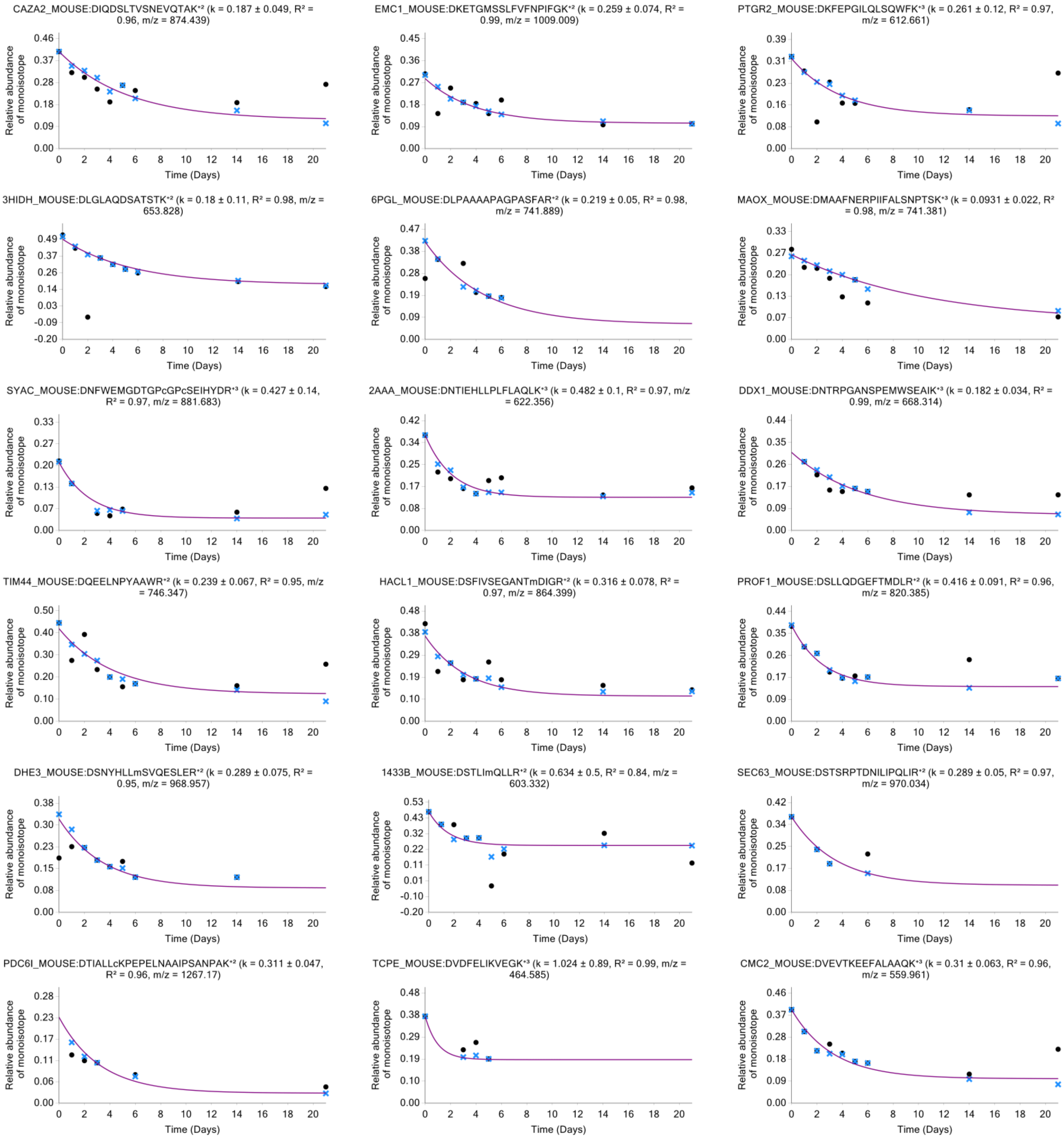

The Figures show the monoisotopic RIAs estimated from the complete isotope profile (black circles), RIA estimates from the complete isotope profiles, and any of the three partial isotope profiles combined ($A_1(t)/A_0(t)$, $A_2(t)/A_0(t)$, $A_2(t)/A_1(t)$) (blue crosses). The solid line shows the fit from the label enrichment determination technique.

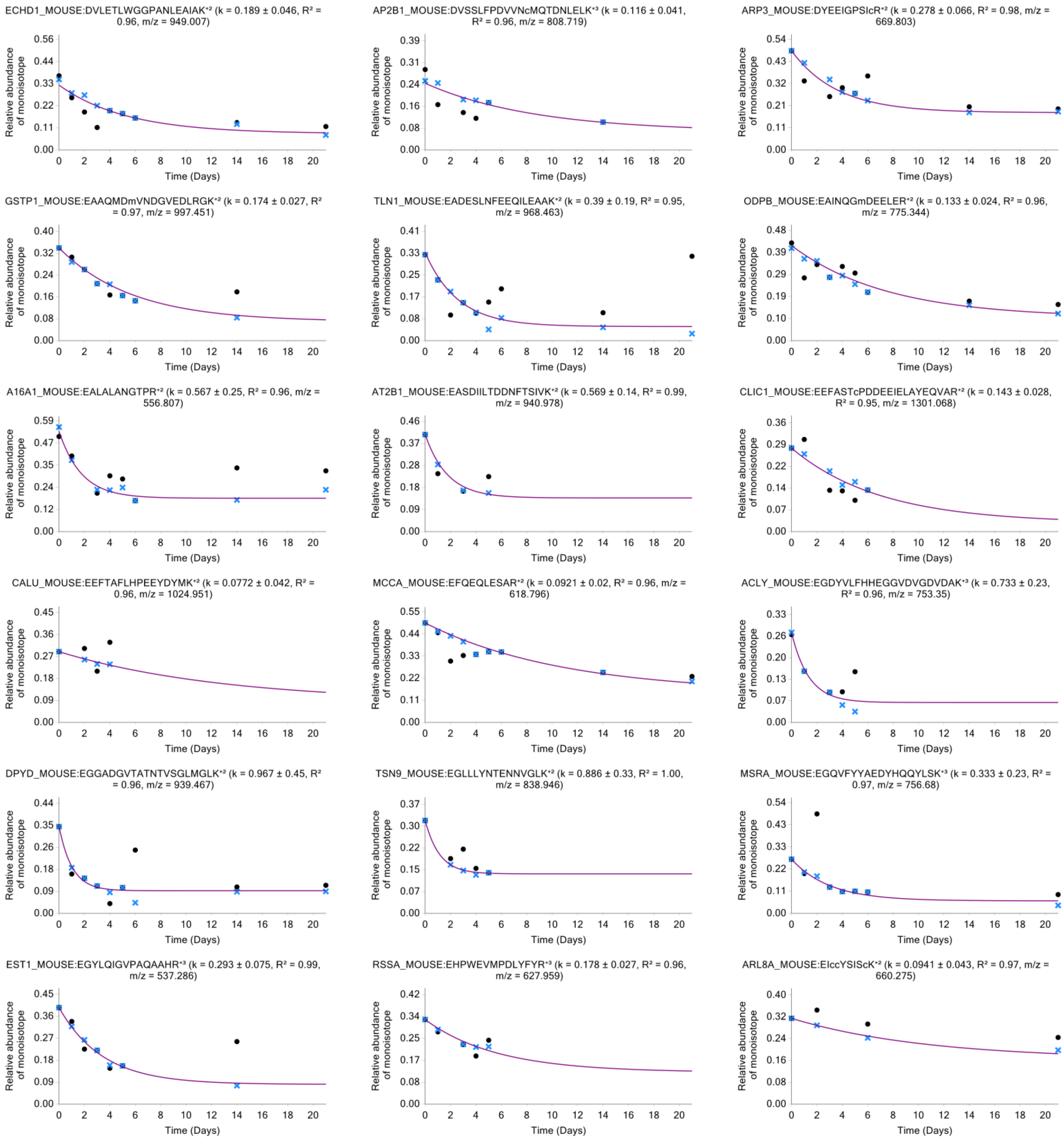

The Figures show the monoisotopic RIAs estimated from the complete isotope profile (black circles), RIA estimates from the complete isotope profiles, and any of the three partial isotope profiles combined ($A_1(t)/A_0(t)$, $A_2(t)/A_0(t)$, $A_2(t)/A_1(t)$) (blue crosses). The solid line shows the fit from the label enrichment determination technique.

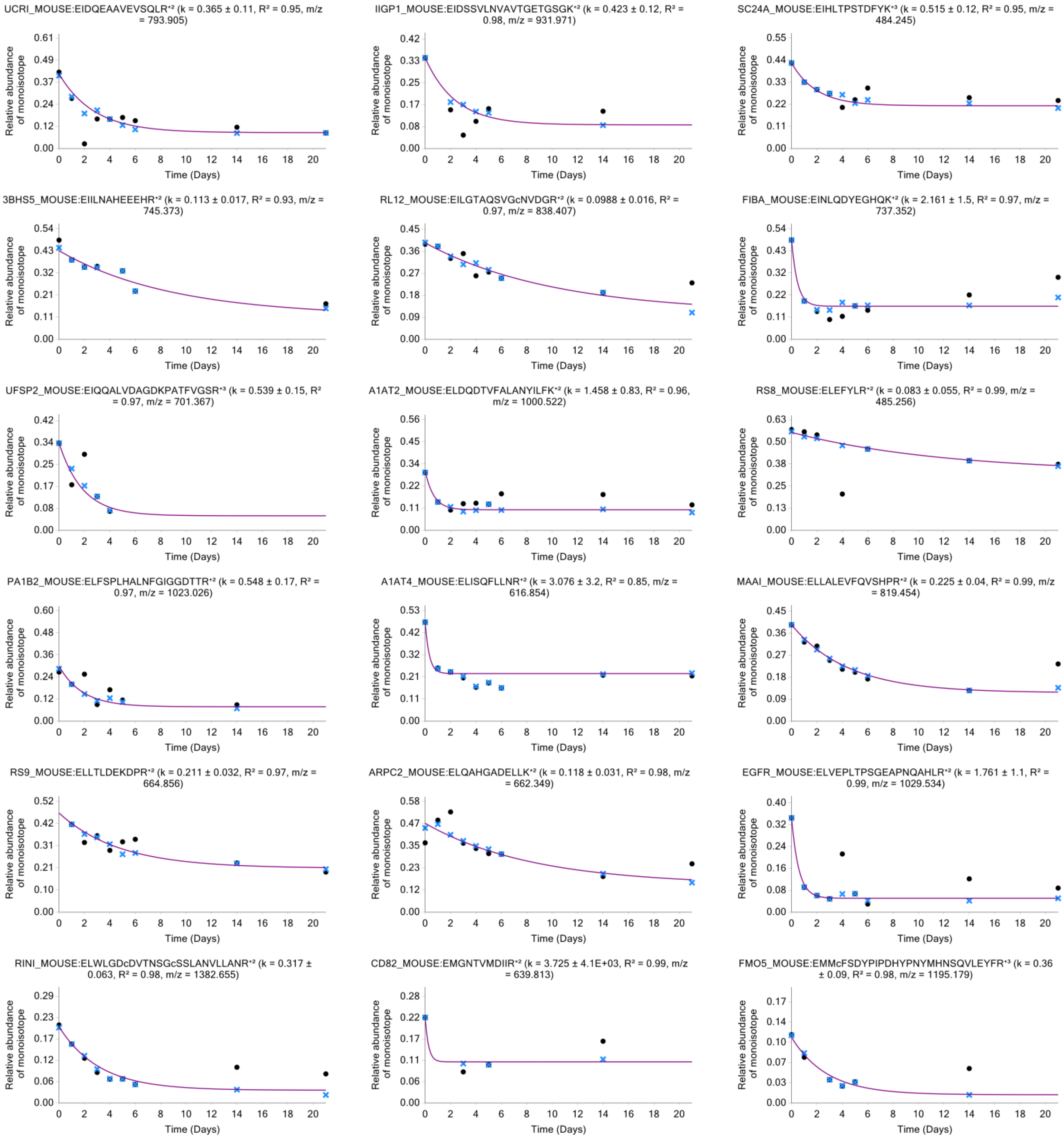

The Figures show the monoisotopic RIAs estimated from the complete isotope profile (black circles), RIA estimates from the complete isotope profiles, and any of the three partial isotope profiles combined ($A_1(t)/A_0(t)$, $A_2(t)/A_0(t)$, $A_2(t)/A_1(t)$) (blue crosses). The solid line shows the fit from the label enrichment determination technique.

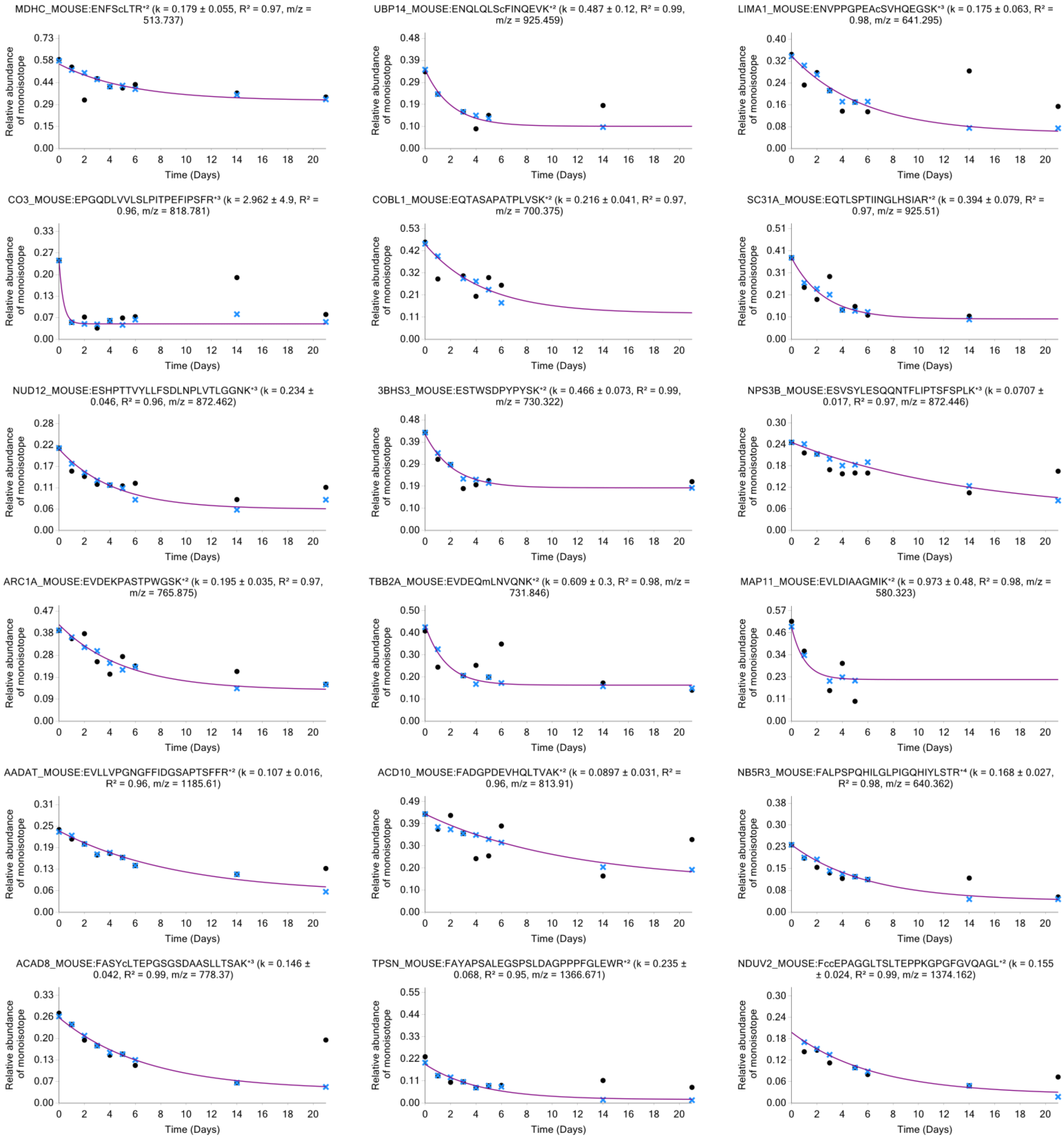

The Figures show the monoisotopic RIAs estimated from the complete isotope profile (black circles), RIA estimates from the complete isotope profiles, and any of the three partial isotope profiles combined ($A_1(t)/A_0(t)$, $A_2(t)/A_0(t)$, $A_2(t)/A_1(t)$) (blue crosses). The solid line shows the fit from the label enrichment determination technique.

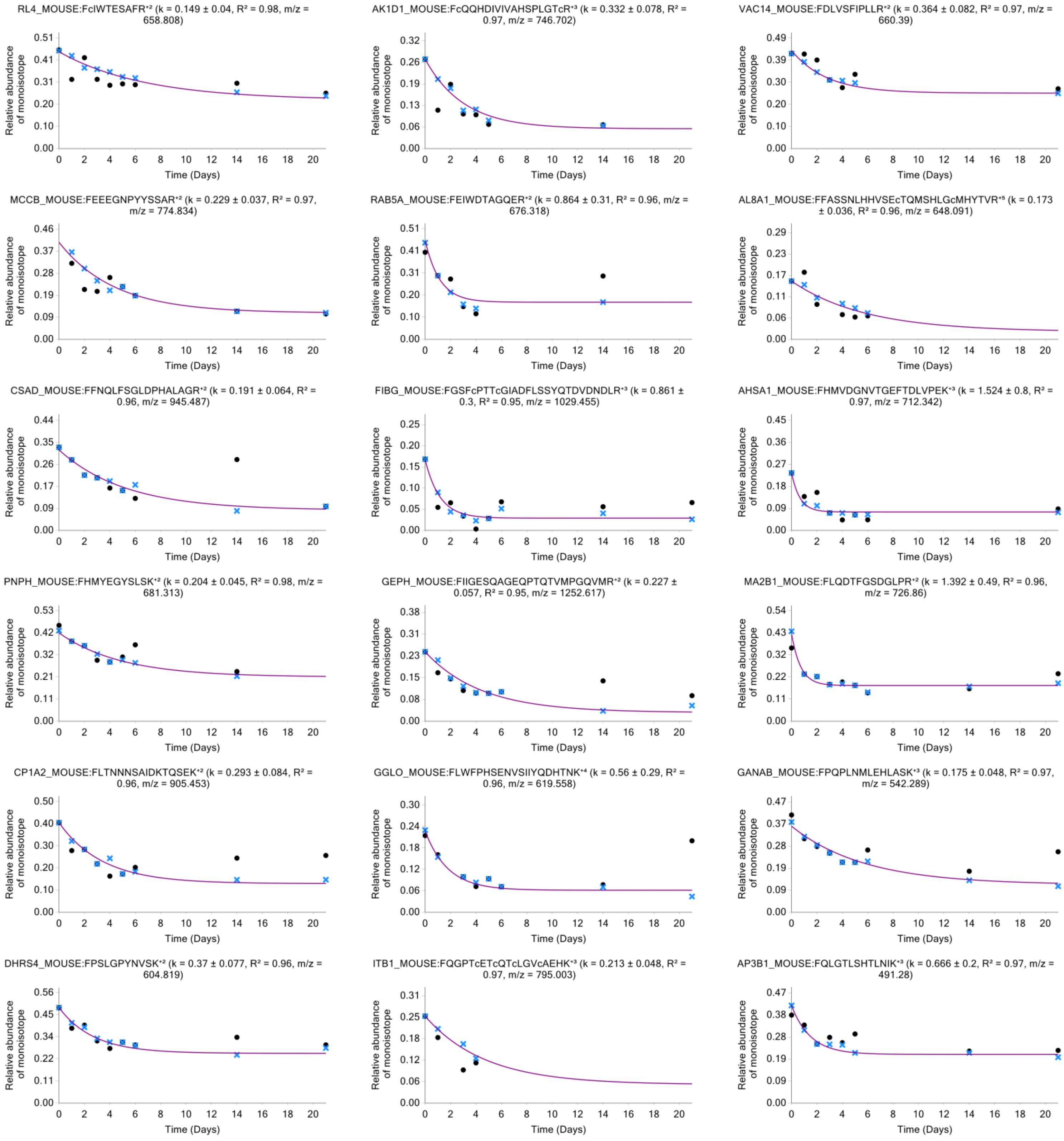

The Figures show the monoisotopic RIAs estimated from the complete isotope profile (black circles), RIA estimates from the complete isotope profiles, and any of the three partial isotope profiles combined ($A_1(t)/A_0(t)$, $A_2(t)/A_0(t)$, $A_2(t)/A_1(t)$) (blue crosses). The solid line shows the fit from the label enrichment determination technique.

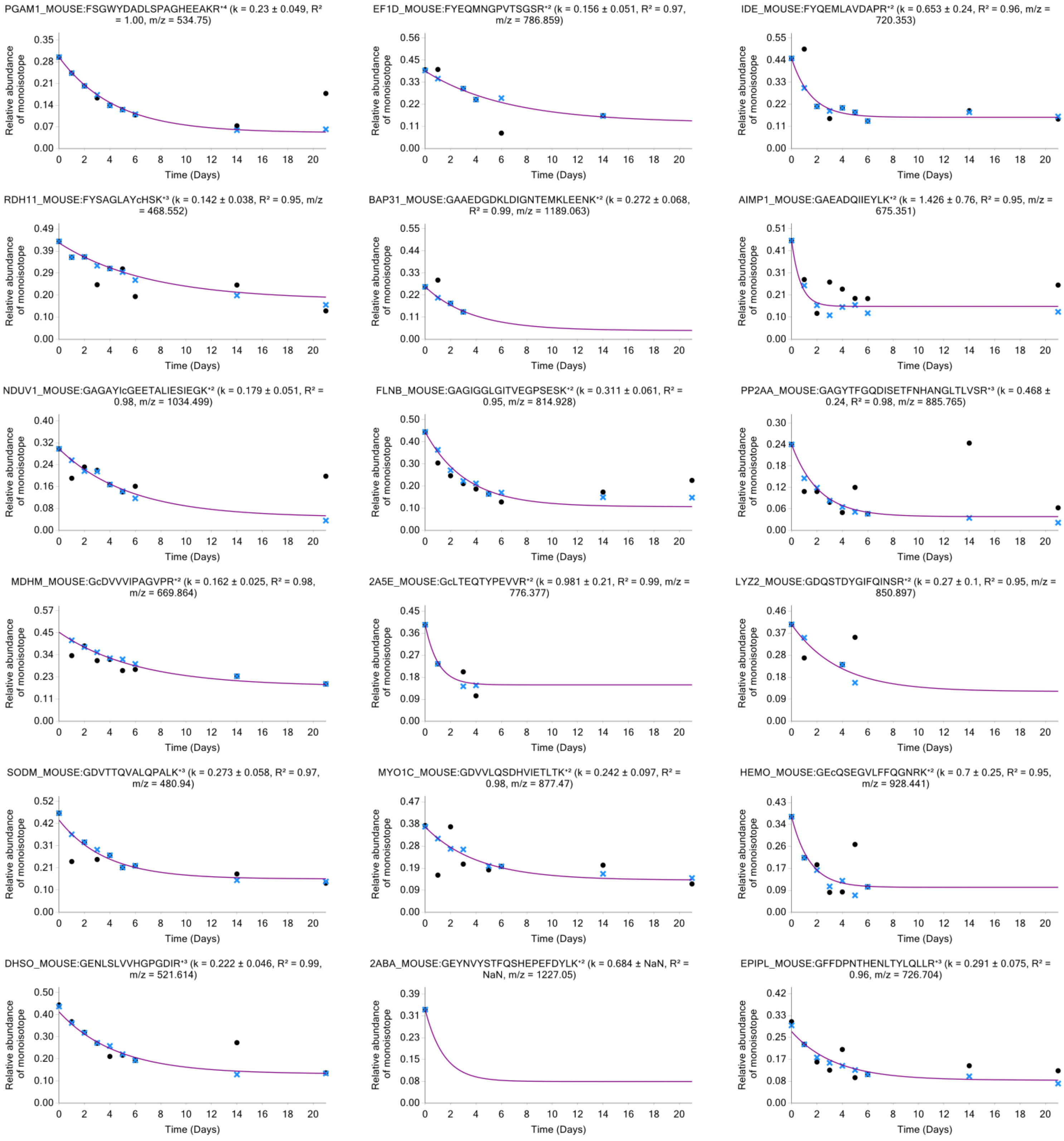

The Figures show the monoisotopic RIAs estimated from the complete isotope profile (black circles), RIA estimates from the complete isotope profiles, and any of the three partial isotope profiles combined ($A_1(t)/A_0(t)$, $A_2(t)/A_0(t)$, $A_2(t)/A_1(t)$) (blue crosses). The solid line shows the fit from the label enrichment determination technique.

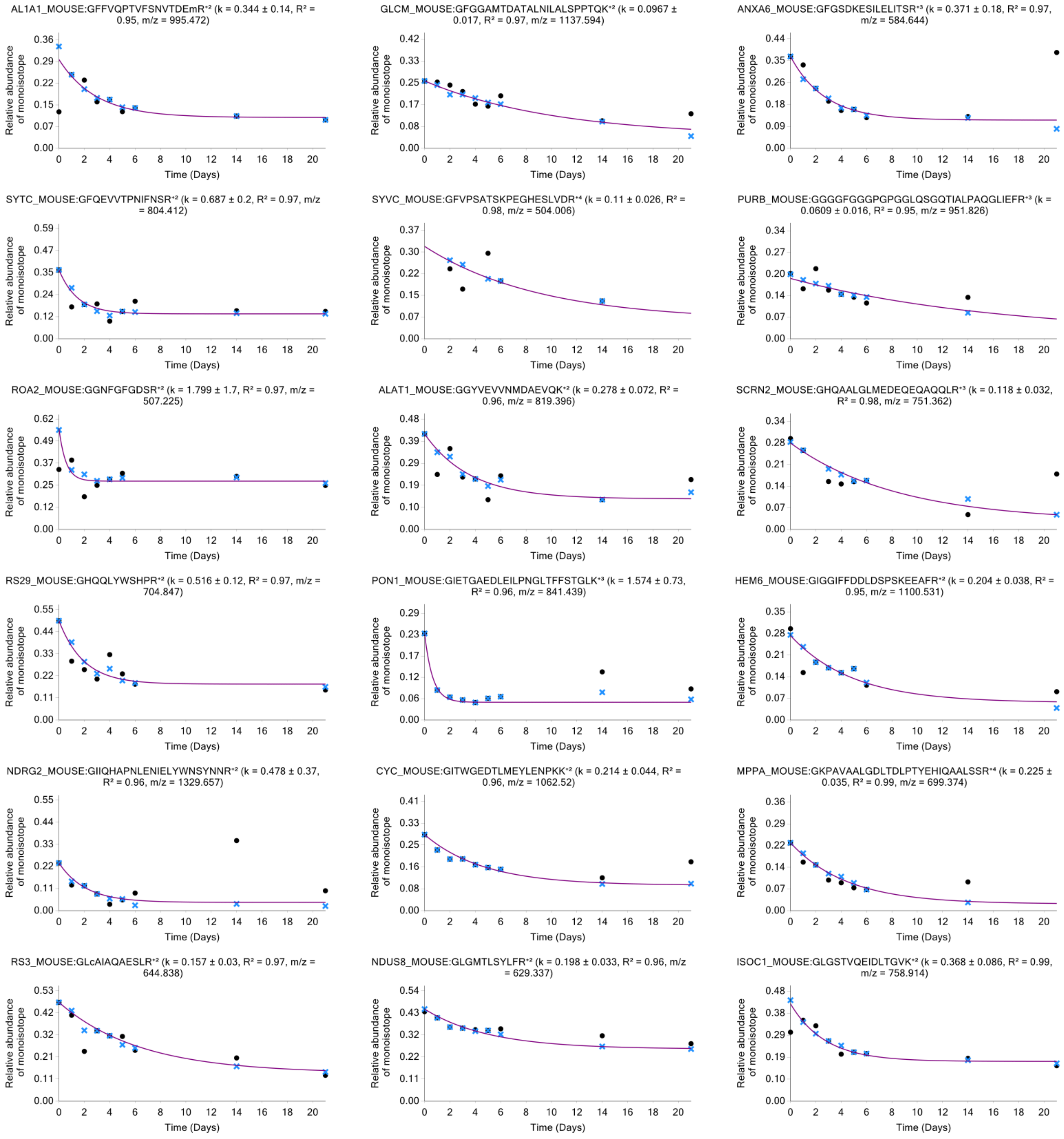

The Figures show the monoisotopic RIAs estimated from the complete isotope profile (black circles), RIA estimates from the complete isotope profiles, and any of the three partial isotope profiles combined ($A_1(t)/A_0(t)$, $A_2(t)/A_0(t)$, $A_2(t)/A_1(t)$) (blue crosses). The solid line shows the fit from the label enrichment determination technique.

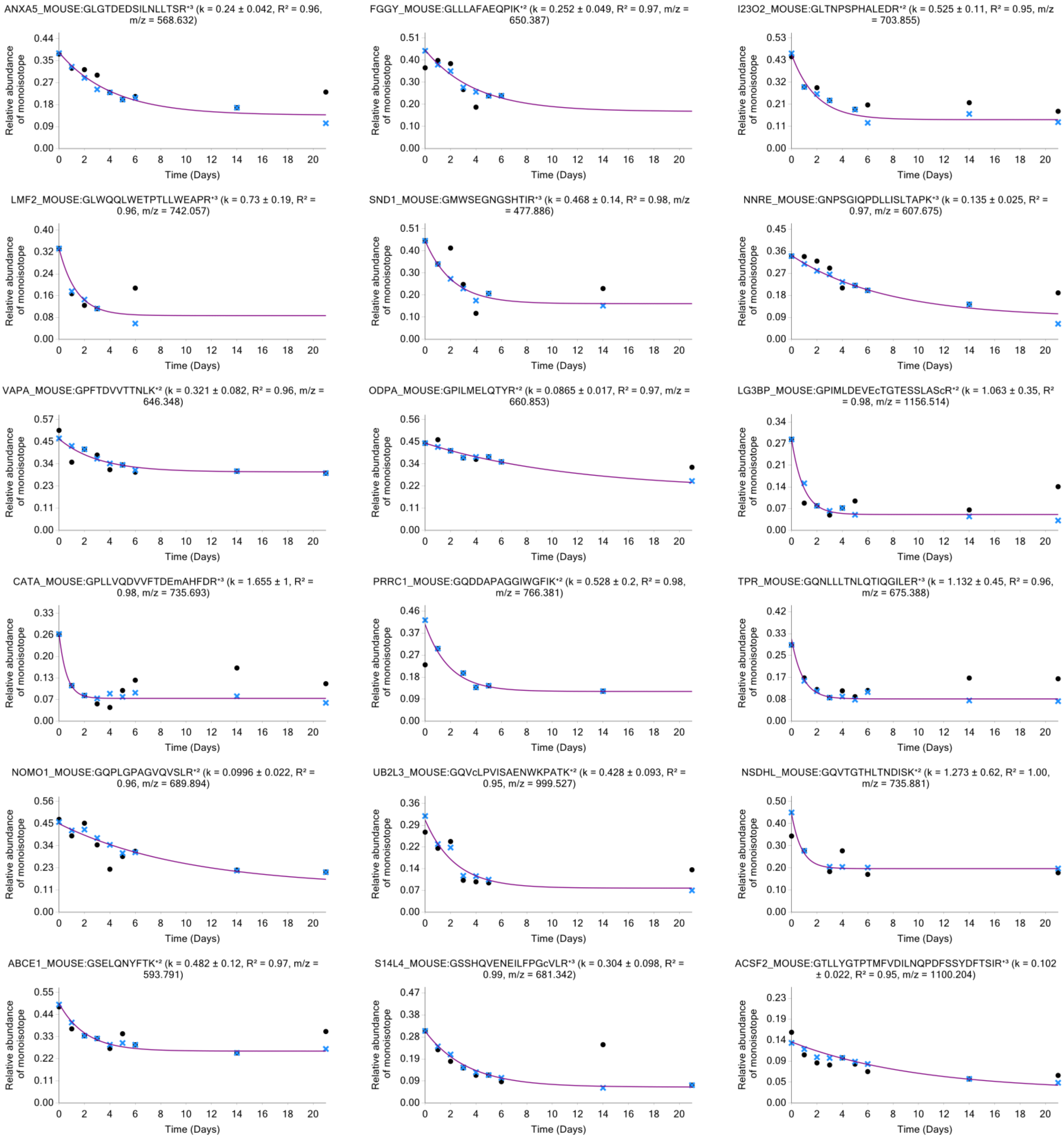

The Figures show the monoisotopic RIAs estimated from the complete isotope profile (black circles), RIA estimates from the complete isotope profiles, and any of the three partial isotope profiles combined ($A_1(t)/A_0(t)$, $A_2(t)/A_0(t)$, $A_2(t)/A_1(t)$) (blue crosses). The solid line shows the fit from the label enrichment determination technique.

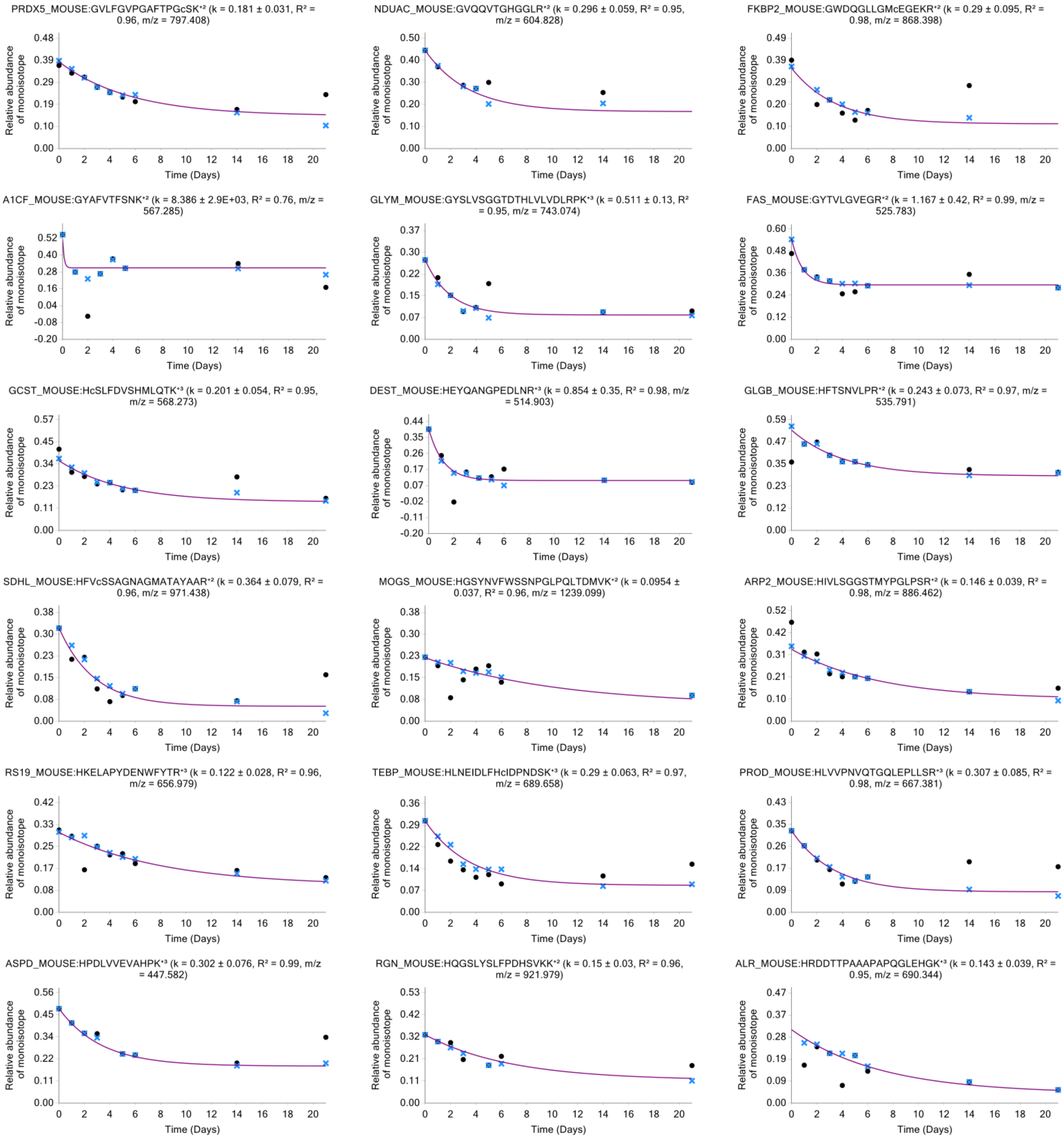

The Figures show the monoisotopic RIAs estimated from the complete isotope profile (black circles), RIA estimates from the complete isotope profiles, and any of the three partial isotope profiles combined ($A_1(t)/A_0(t)$, $A_2(t)/A_0(t)$, $A_2(t)/A_1(t)$) (blue crosses). The solid line shows the fit from the label enrichment determination technique.

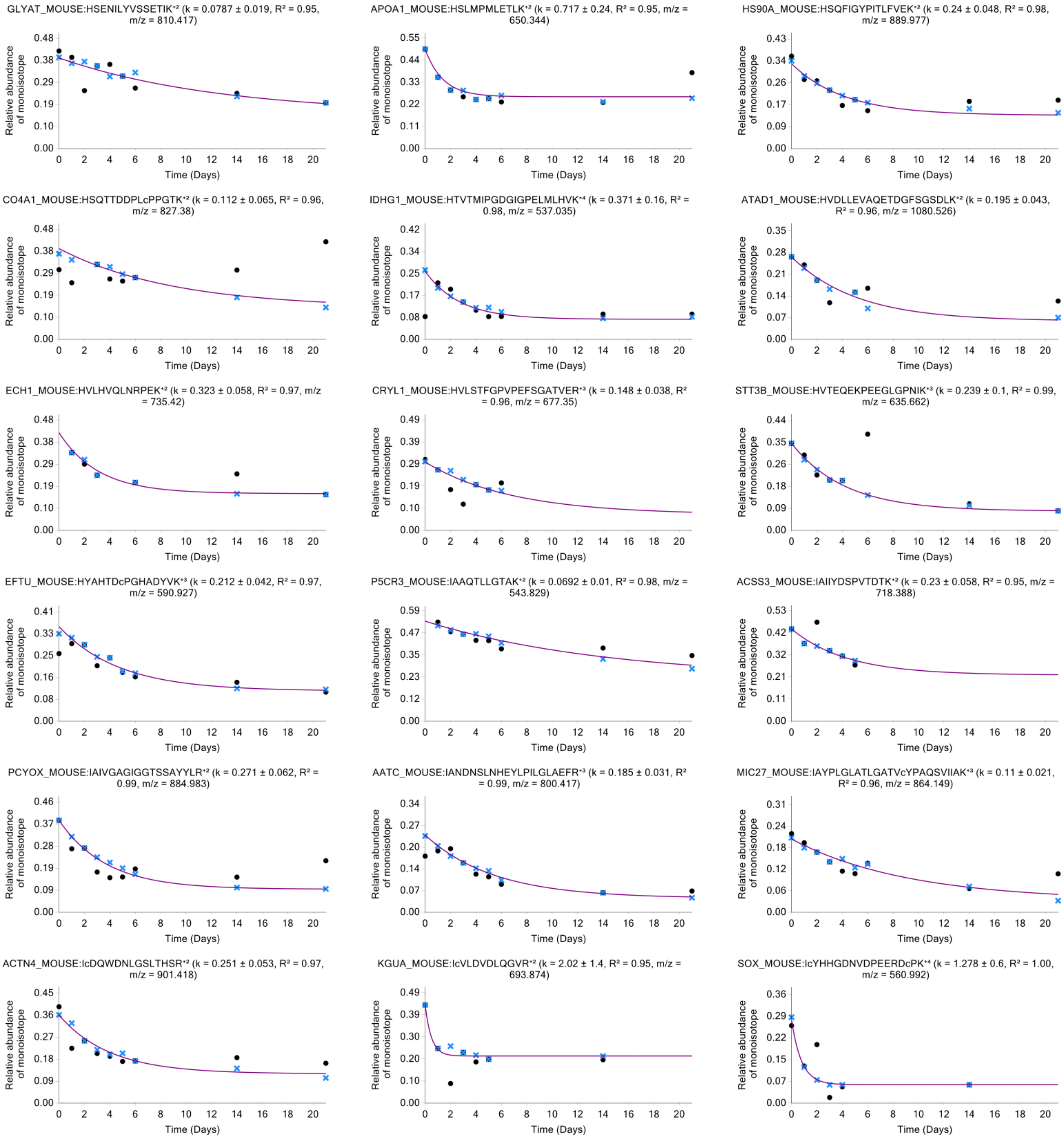

The Figures show the monoisotopic RIAs estimated from the complete isotope profile (black circles), RIA estimates from the complete isotope profiles, and any of the three partial isotope profiles combined ($A_1(t)/A_0(t)$, $A_2(t)/A_0(t)$, $A_2(t)/A_1(t)$) (blue crosses). The solid line shows the fit from the label enrichment determination technique.

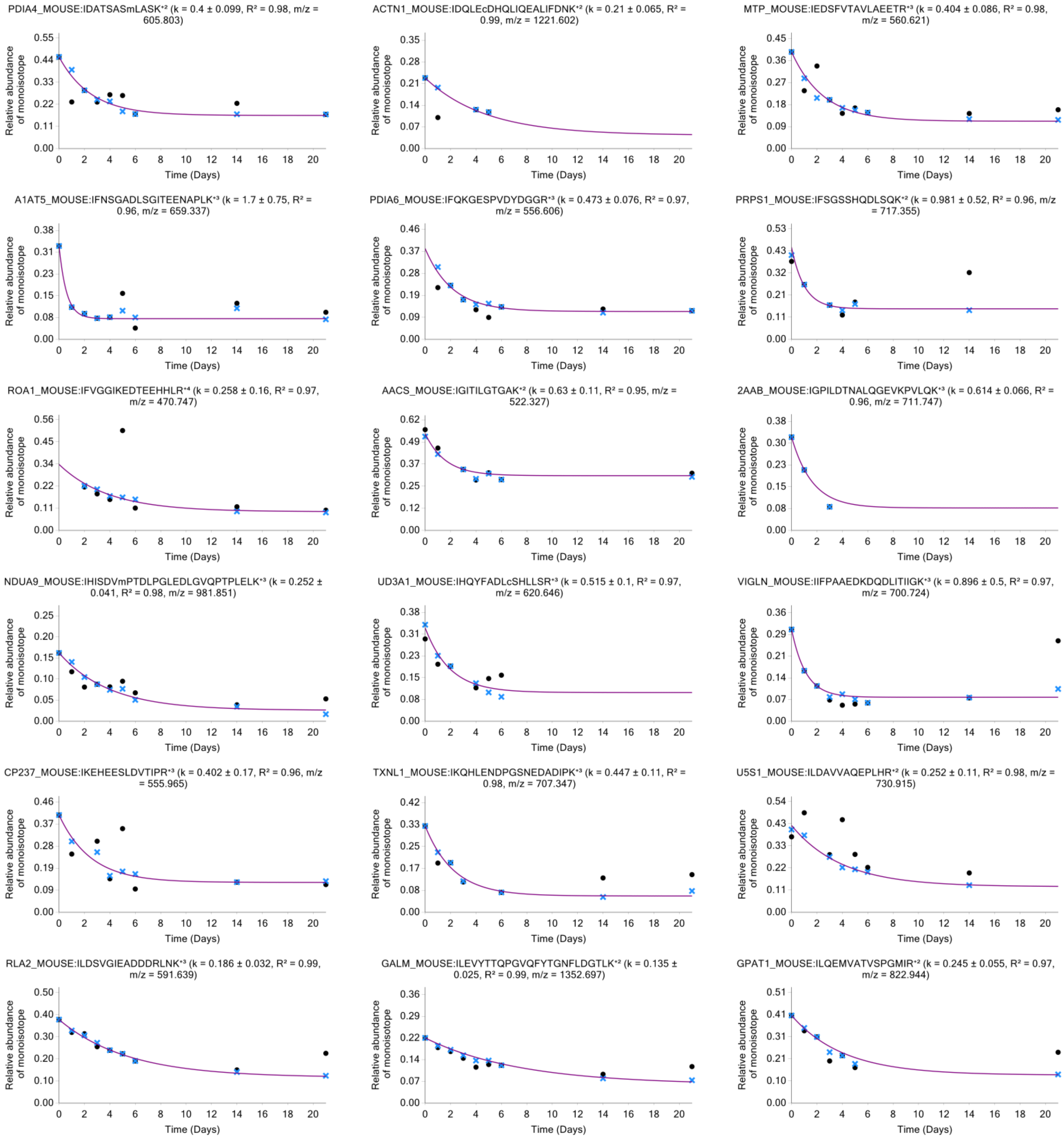

The Figures show the monoisotopic RIAs estimated from the complete isotope profile (black circles), RIA estimates from the complete isotope profiles, and any of the three partial isotope profiles combined ($A_1(t)/A_0(t)$, $A_2(t)/A_0(t)$, $A_2(t)/A_1(t)$) (blue crosses). The solid line shows the fit from the label enrichment determination technique.

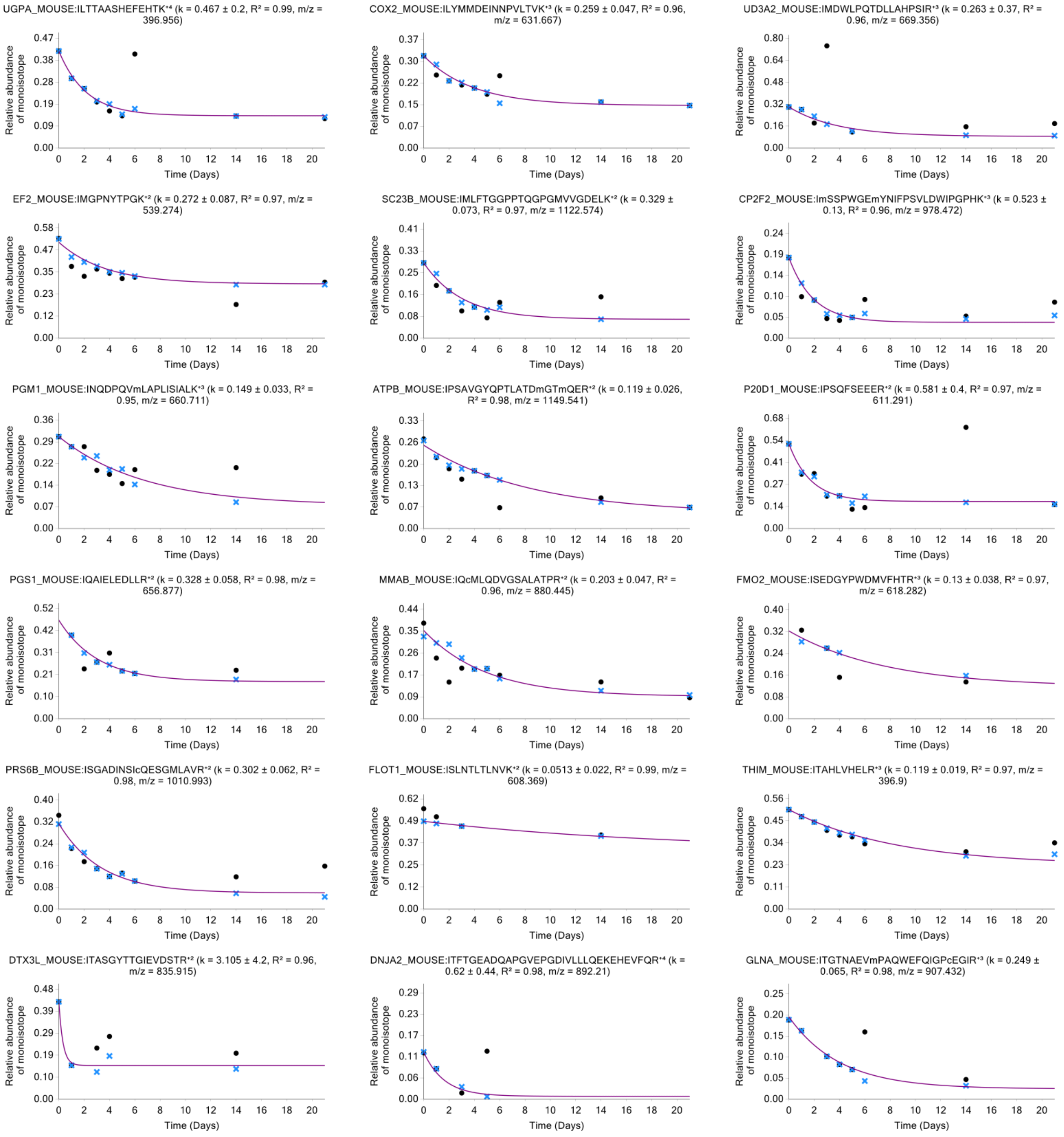

The Figures show the monoisotopic RIAs estimated from the complete isotope profile (black circles), RIA estimates from the complete isotope profiles, and any of the three partial isotope profiles combined ($A_1(t)/A_0(t)$, $A_2(t)/A_0(t)$, $A_2(t)/A_1(t)$) (blue crosses). The solid line shows the fit from the label enrichment determination technique.

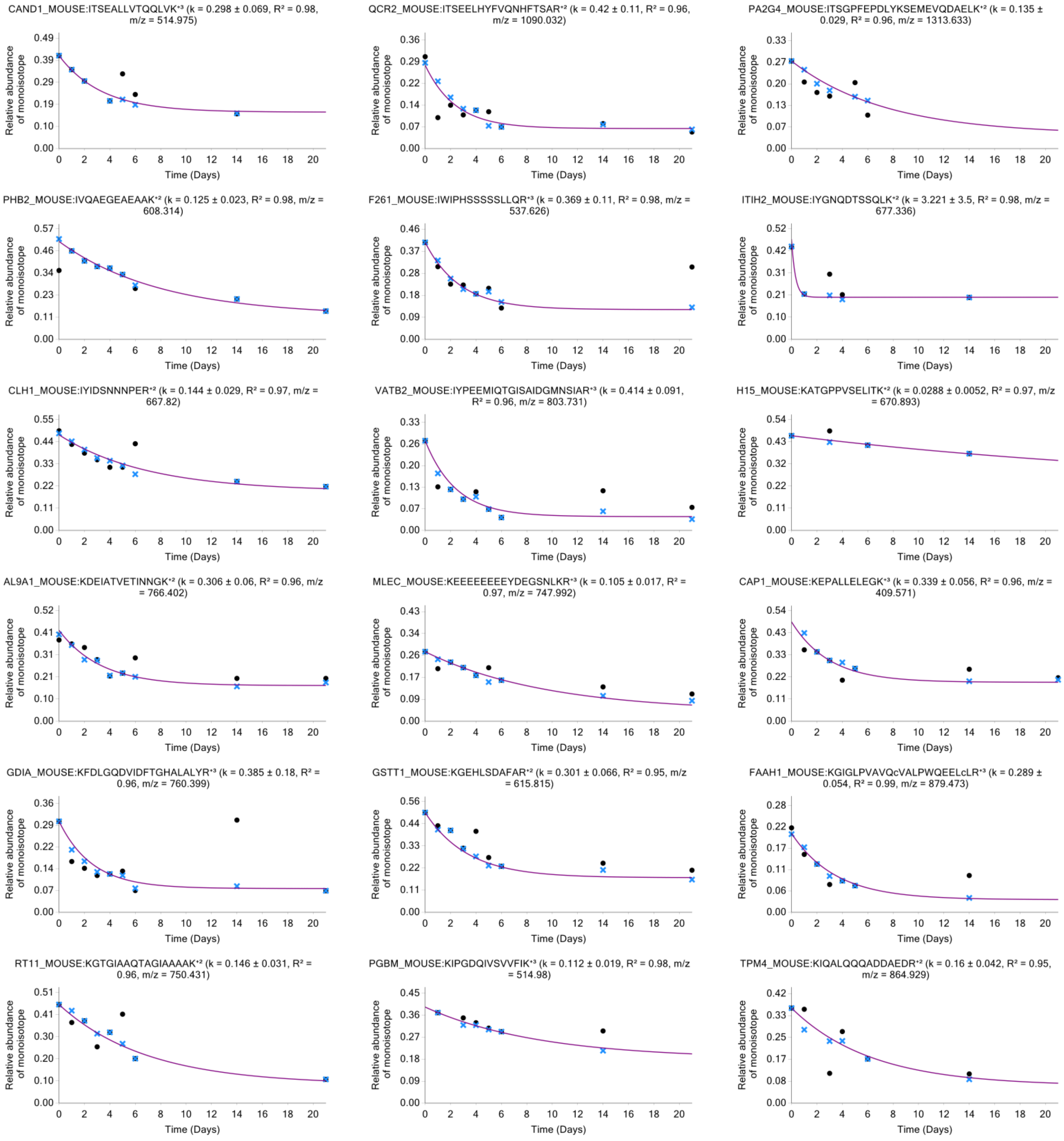

The Figures show the monoisotopic RIAs estimated from the complete isotope profile (black circles), RIA estimates from the complete isotope profiles, and any of the three partial isotope profiles combined ($A_1(t)/A_0(t)$, $A_2(t)/A_0(t)$, $A_2(t)/A_1(t)$) (blue crosses). The solid line shows the fit from the label enrichment determination technique.

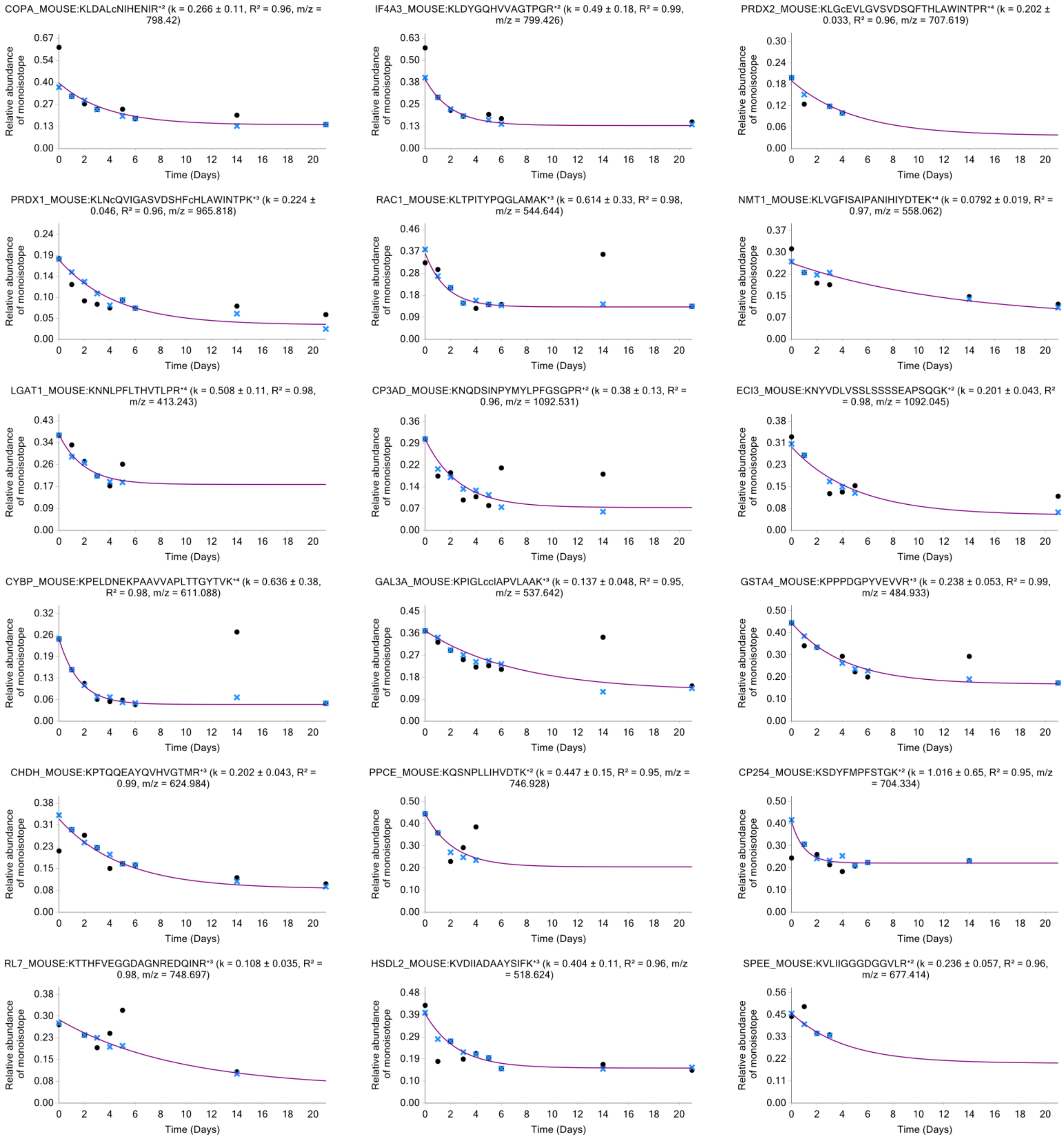

The Figures show the monoisotopic RIAs estimated from the complete isotope profile (black circles), RIA estimates from the complete isotope profiles, and any of the three partial isotope profiles combined ($A_1(t)/A_0(t)$, $A_2(t)/A_0(t)$, $A_2(t)/A_1(t)$) (blue crosses). The solid line shows the fit from the label enrichment determination technique.

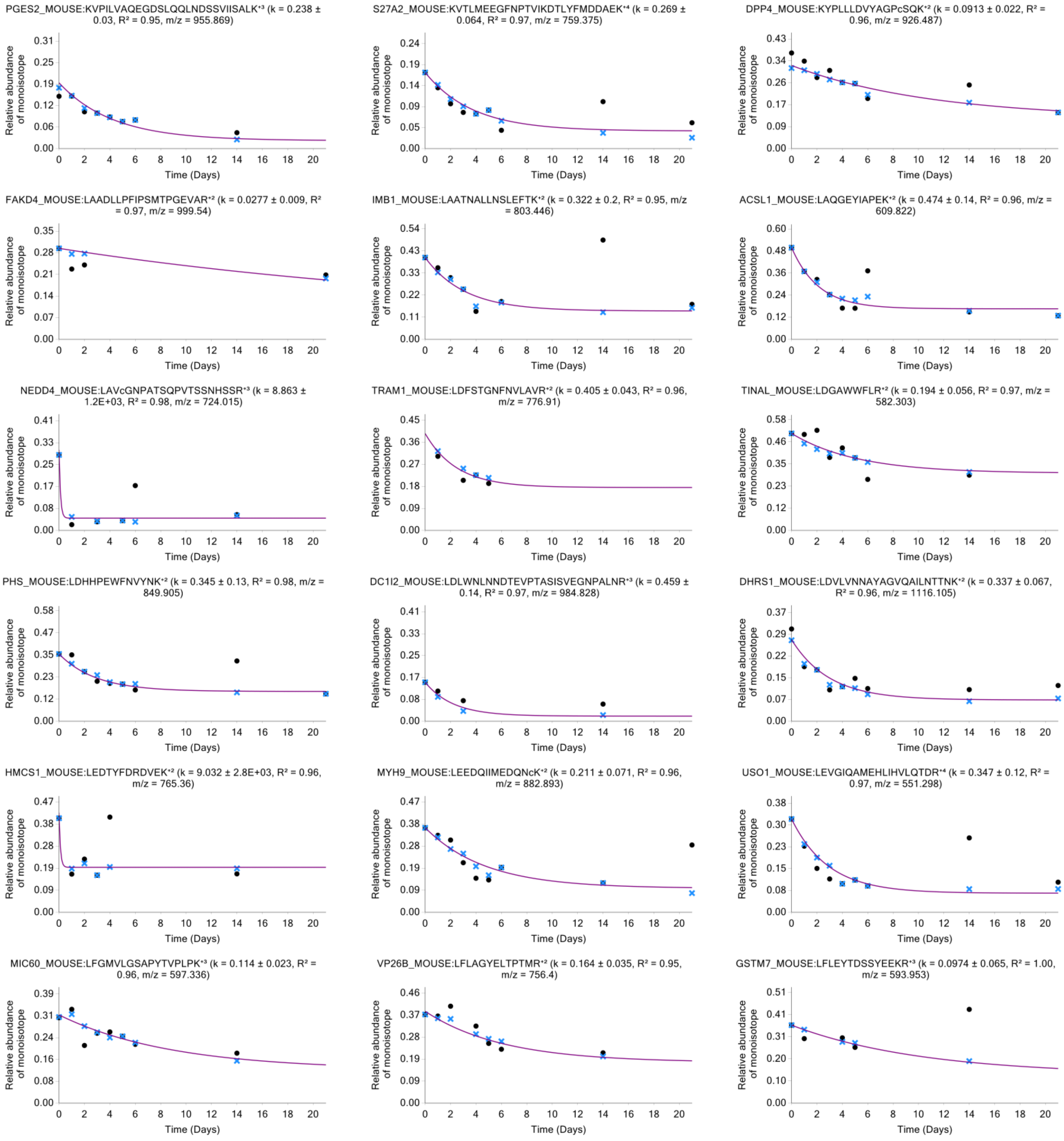

The Figures show the monoisotopic RIAs estimated from the complete isotope profile (black circles), RIA estimates from the complete isotope profiles, and any of the three partial isotope profiles combined ($A_1(t)/A_0(t)$, $A_2(t)/A_0(t)$, $A_2(t)/A_1(t)$) (blue crosses). The solid line shows the fit from the label enrichment determination technique.

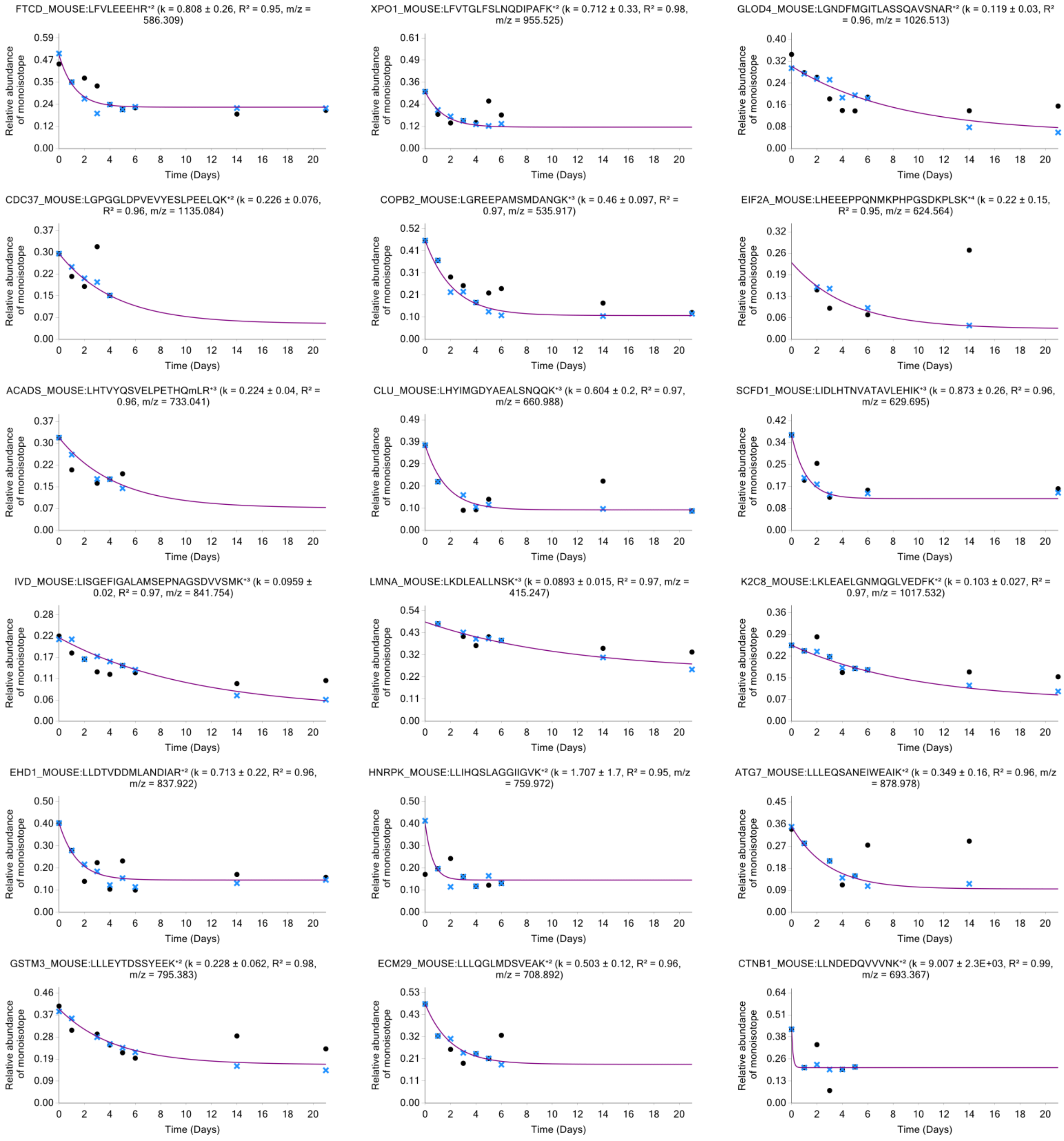

The Figures show the monoisotopic RIAs estimated from the complete isotope profile (black circles), RIA estimates from the complete isotope profiles, and any of the three partial isotope profiles combined ($A_1(t)/A_0(t)$, $A_2(t)/A_0(t)$, $A_2(t)/A_1(t)$) (blue crosses). The solid line shows the fit from the label enrichment determination technique.

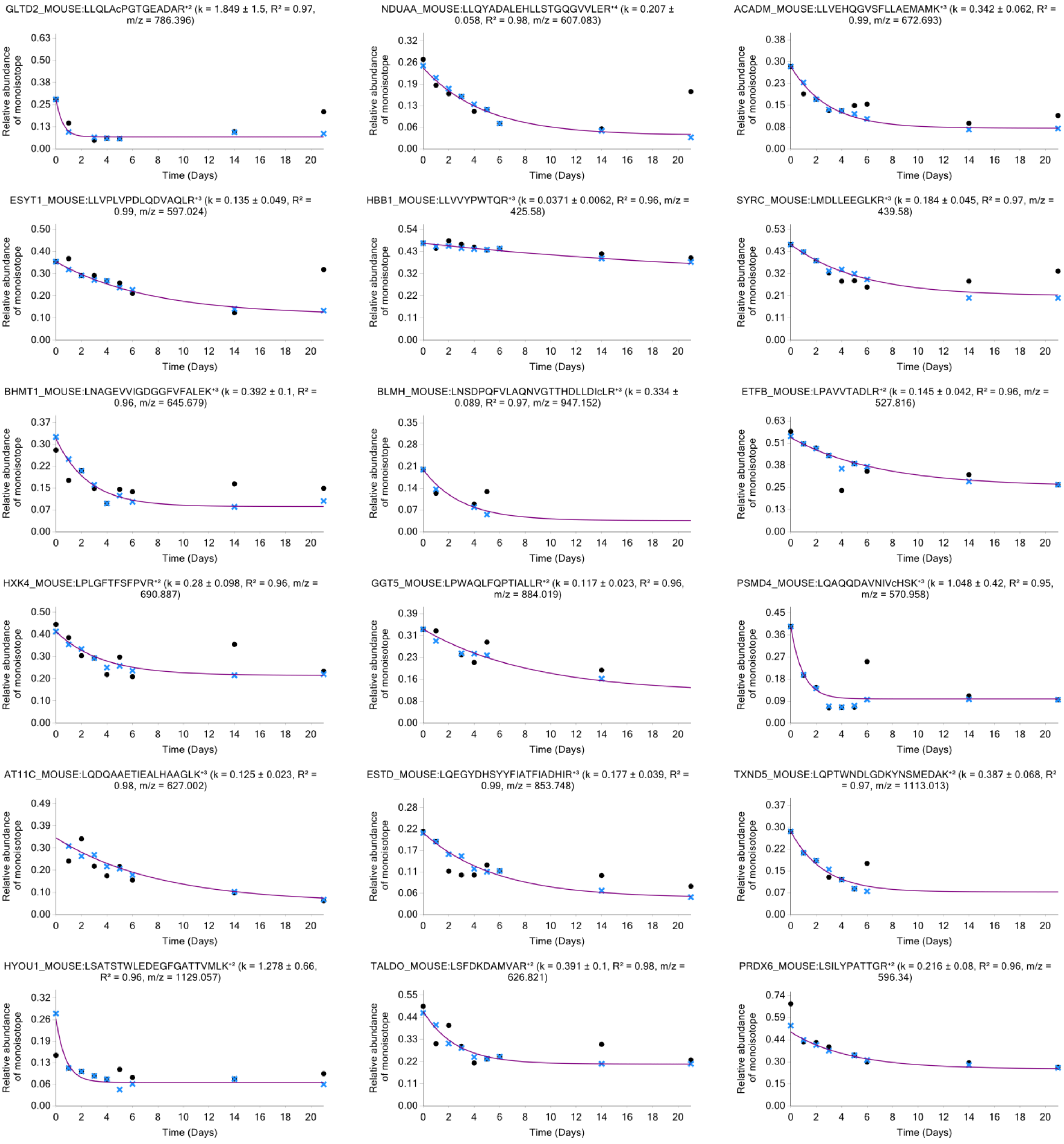

The Figures show the monoisotopic RIAs estimated from the complete isotope profile (black circles), RIA estimates from the complete isotope profiles, and any of the three partial isotope profiles combined ($A_1(t)/A_0(t)$, $A_2(t)/A_0(t)$, $A_2(t)/A_1(t)$) (blue crosses). The solid line shows the fit from the label enrichment determination technique.

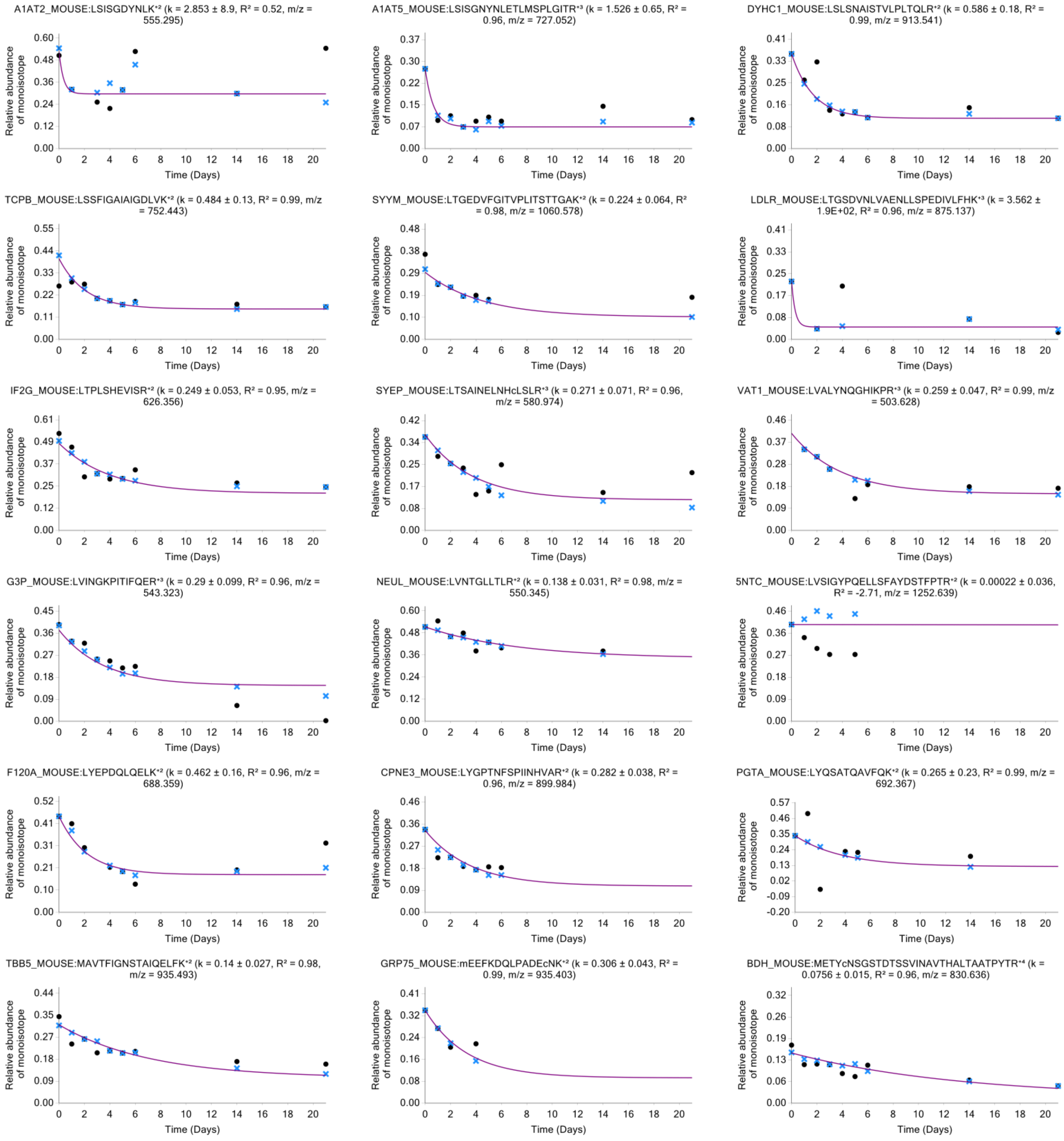

The Figures show the monoisotopic RIAs estimated from the complete isotope profile (black circles), RIA estimates from the complete isotope profiles, and any of the three partial isotope profiles combined ($A_1(t)/A_0(t)$, $A_2(t)/A_0(t)$, $A_2(t)/A_1(t)$) (blue crosses). The solid line shows the fit from the label enrichment determination technique.

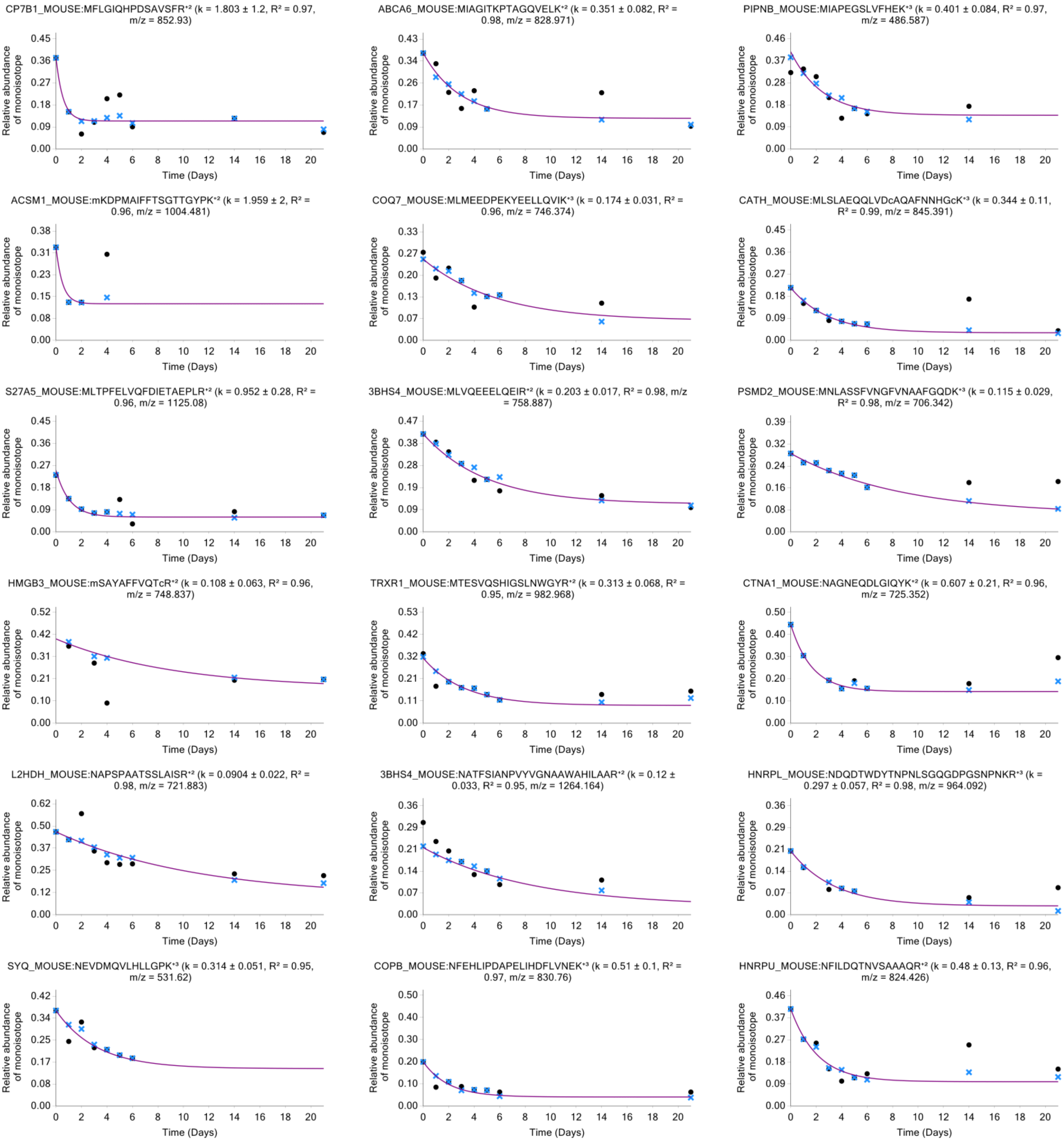

The Figures show the monoisotopic RIAs estimated from the complete isotope profile (black circles), RIA estimates from the complete isotope profiles, and any of the three partial isotope profiles combined ($A_1(t)/A_0(t)$, $A_2(t)/A_0(t)$, $A_2(t)/A_1(t)$) (blue crosses). The solid line shows the fit from the label enrichment determination technique.

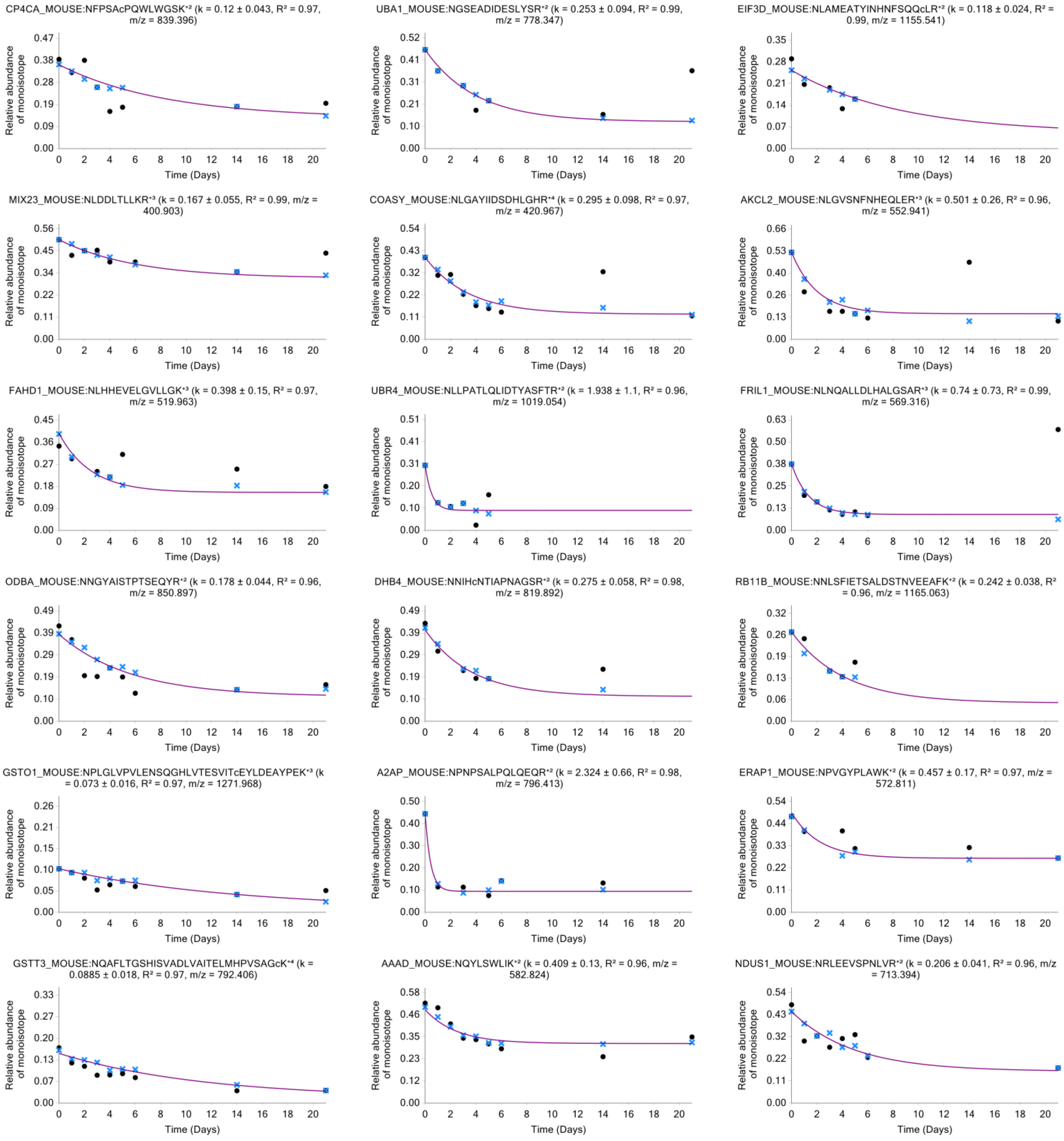

The Figures show the monoisotopic RIAs estimated from the complete isotope profile (black circles), RIA estimates from the complete isotope profiles, and any of the three partial isotope profiles combined ($A_1(t)/A_0(t)$, $A_2(t)/A_0(t)$, $A_2(t)/A_1(t)$) (blue crosses). The solid line shows the fit from the label enrichment determination technique.

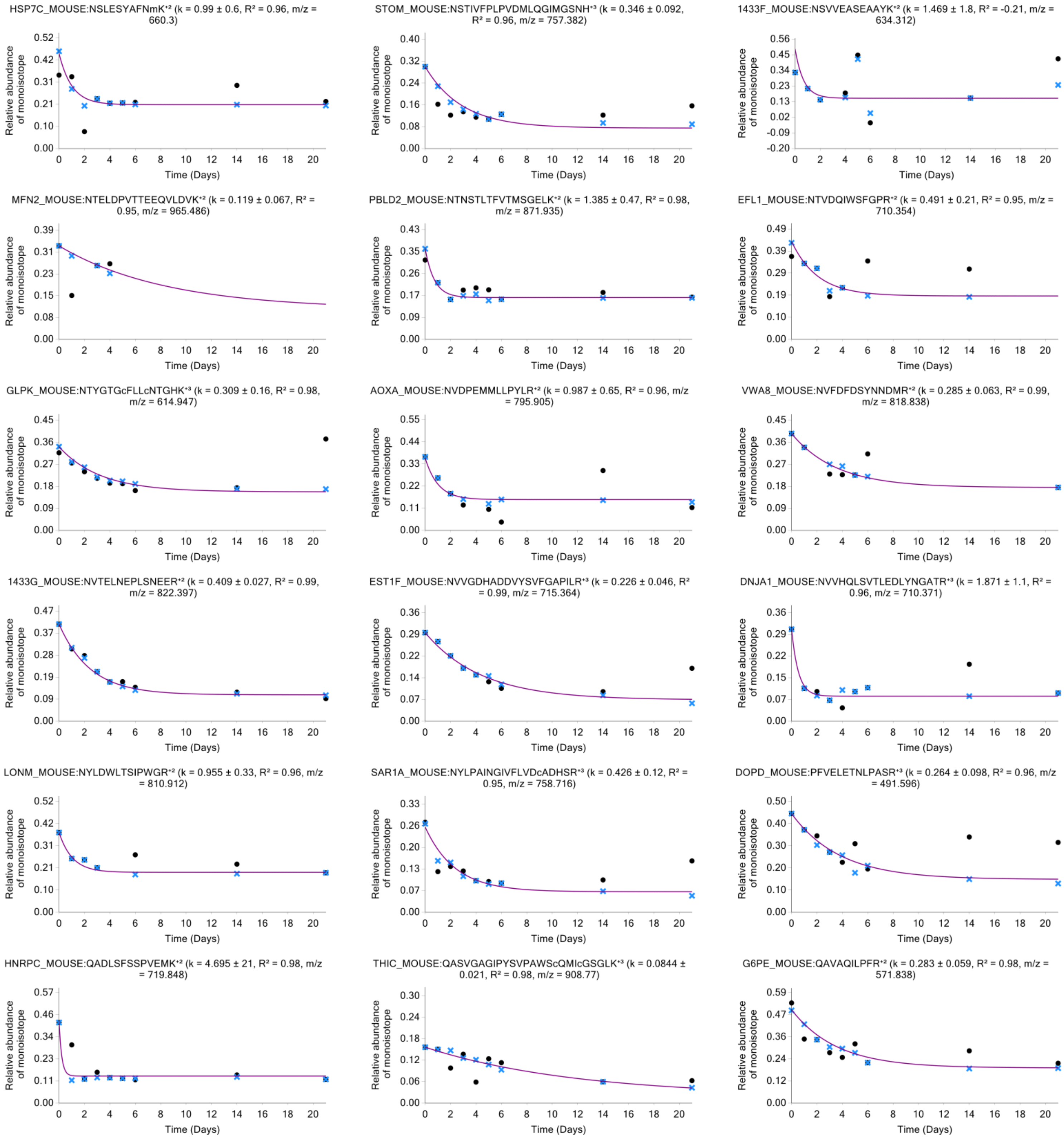

The Figures show the monoisotopic RIAs estimated from the complete isotope profile (black circles), RIA estimates from the complete isotope profiles, and any of the three partial isotope profiles combined ($A_1(t)/A_0(t)$, $A_2(t)/A_0(t)$, $A_2(t)/A_1(t)$) (blue crosses). The solid line shows the fit from the label enrichment determination technique.

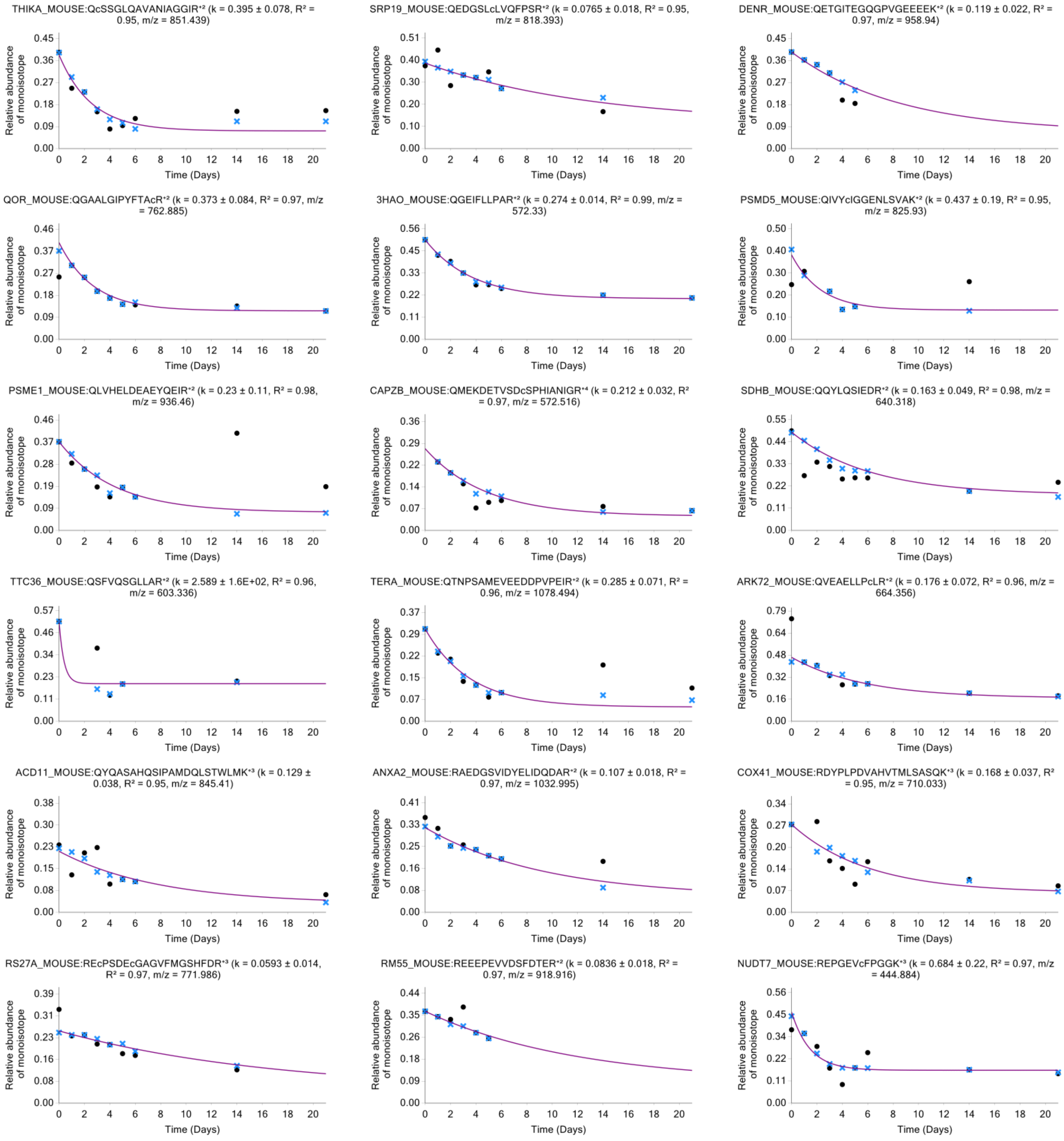

The Figures show the monoisotopic RIAs estimated from the complete isotope profile (black circles), RIA estimates from the complete isotope profiles, and any of the three partial isotope profiles combined ($A_1(t)/A_0(t)$, $A_2(t)/A_0(t)$, $A_2(t)/A_1(t)$) (blue crosses). The solid line shows the fit from the label enrichment determination technique.

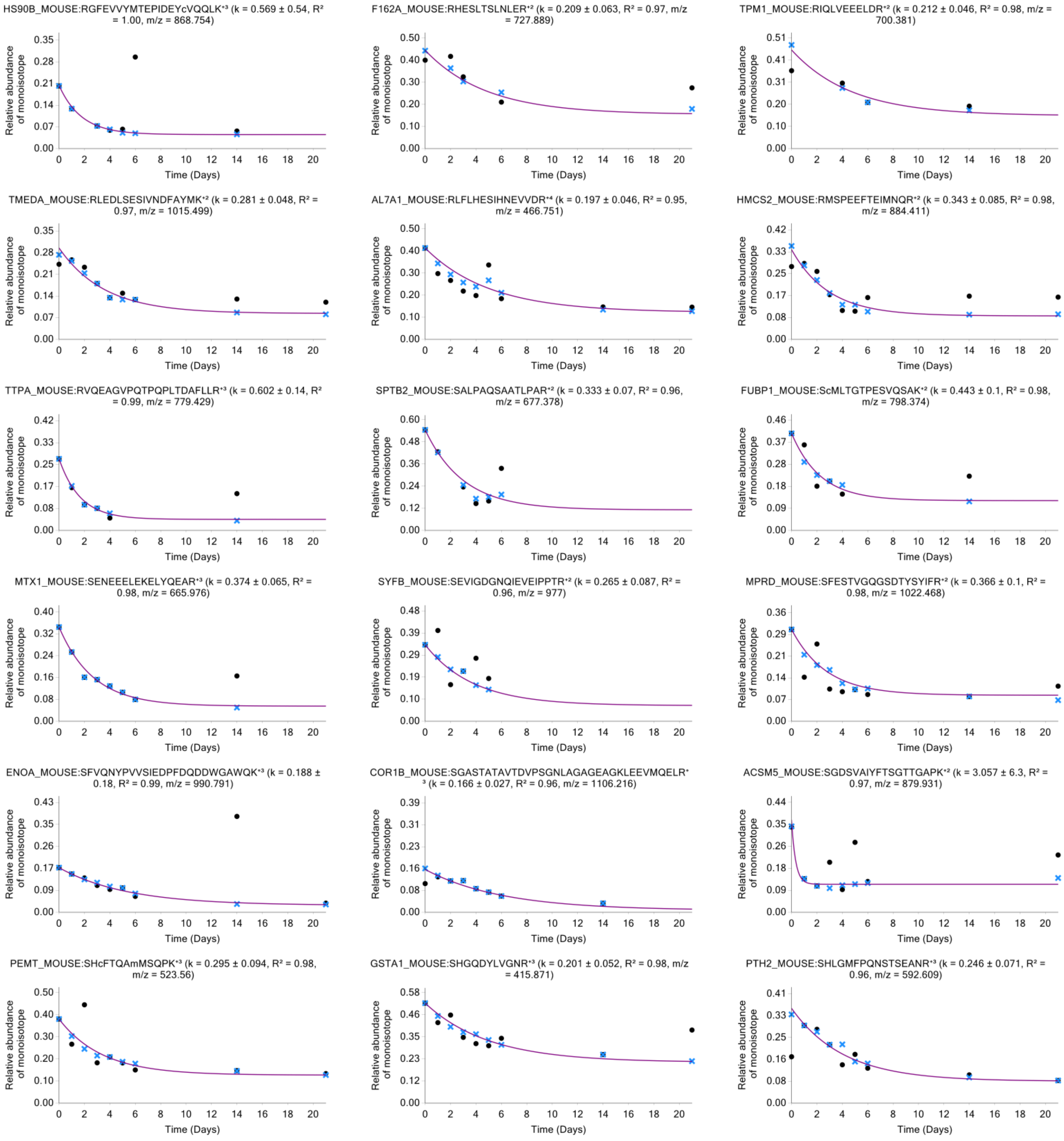

The Figures show the monoisotopic RIAs estimated from the complete isotope profile (black circles), RIA estimates from the complete isotope profiles, and any of the three partial isotope profiles combined ($A_1(t)/A_0(t)$, $A_2(t)/A_0(t)$, $A_2(t)/A_1(t)$) (blue crosses). The solid line shows the fit from the label enrichment determination technique.

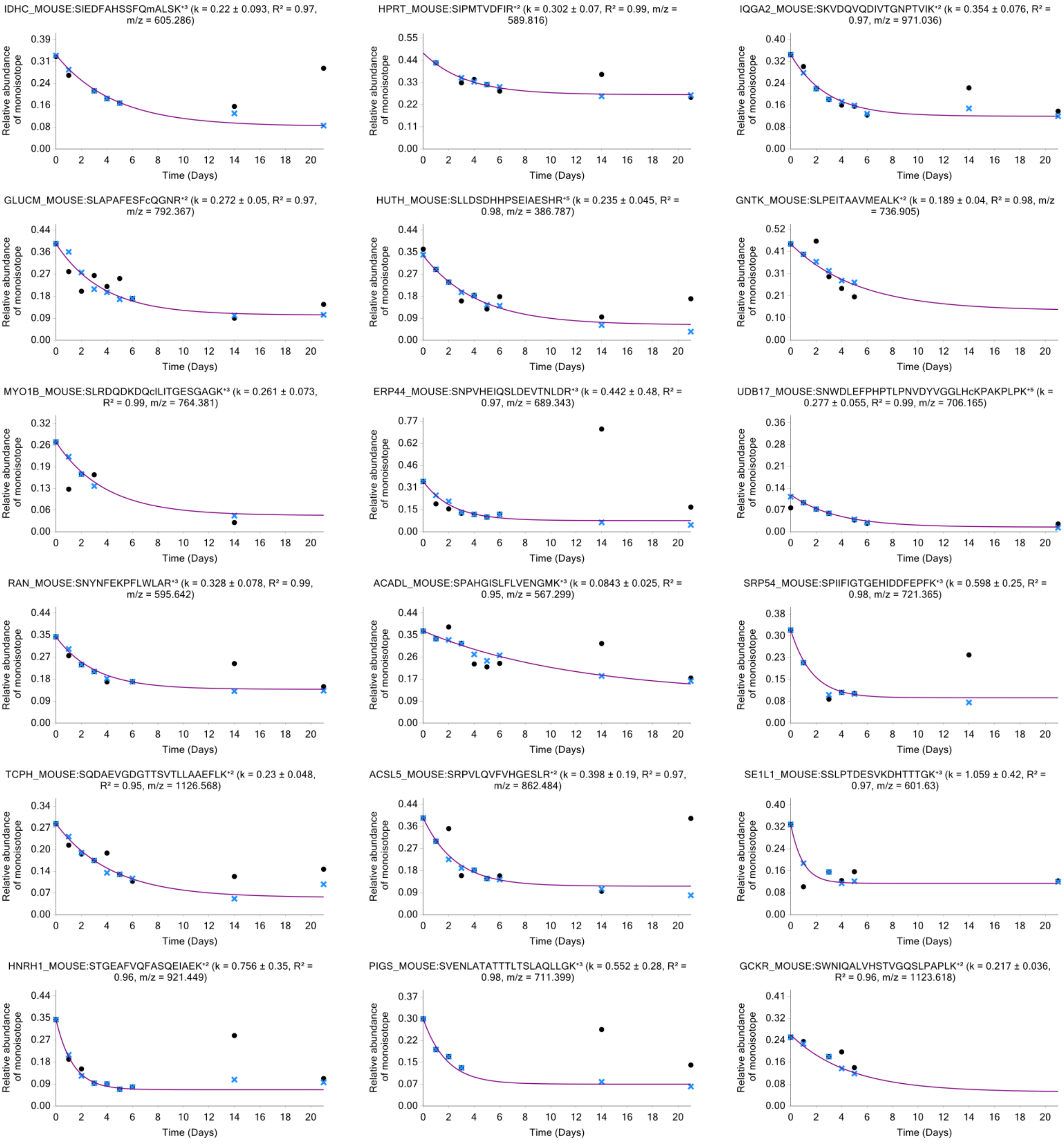

The Figures show the monoisotopic RIAs estimated from the complete isotope profile (black circles), RIA estimates from the complete isotope profiles, and any of the three partial isotope profiles combined ($A_1(t)/A_0(t)$, $A_2(t)/A_0(t)$, $A_2(t)/A_1(t)$) (blue crosses). The solid line shows the fit from the label enrichment determination technique.

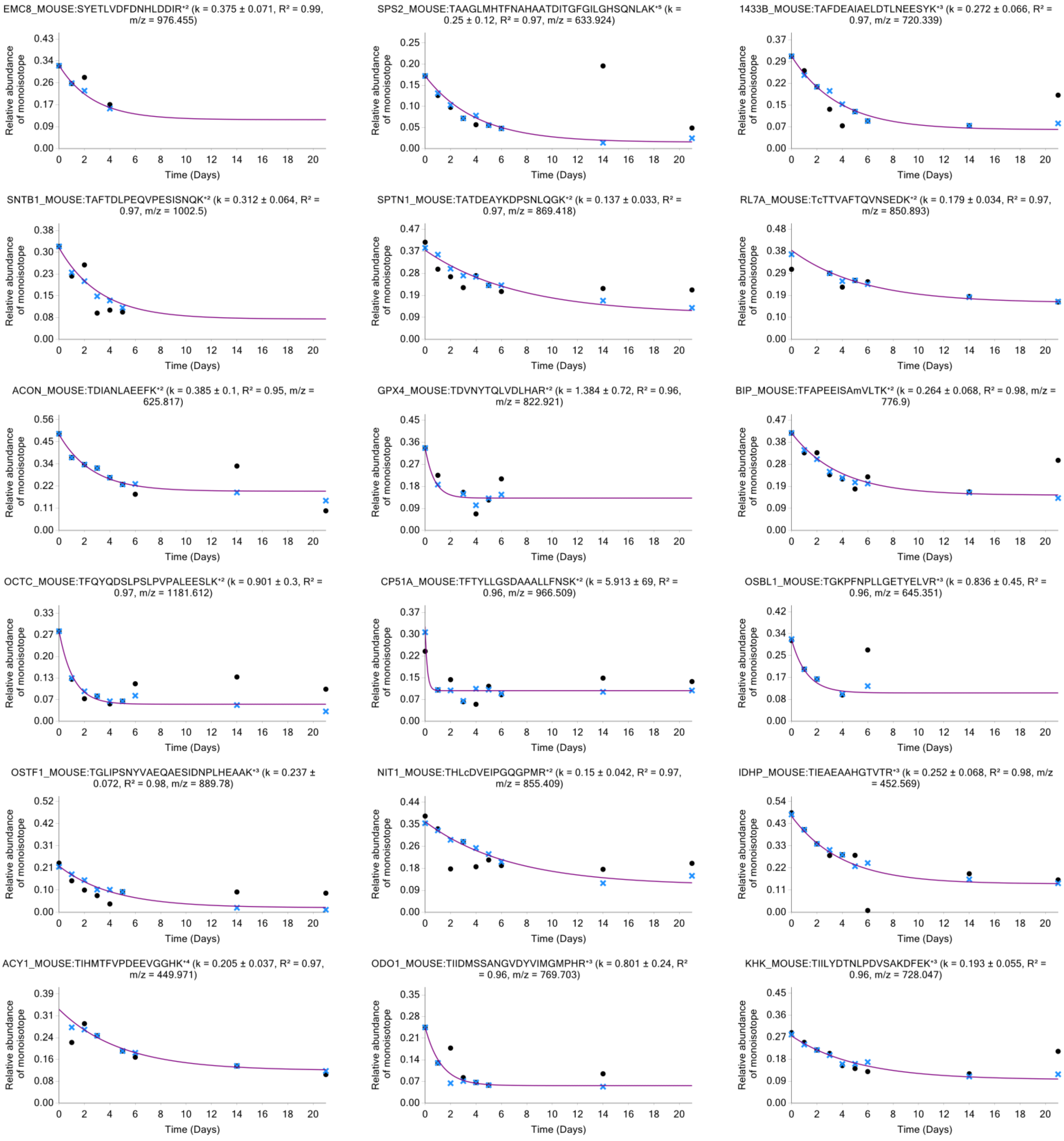

The Figures show the monoisotopic RIAs estimated from the complete isotope profile (black circles), RIA estimates from the complete isotope profiles, and any of the three partial isotope profiles combined ($A_1(t)/A_0(t)$, $A_2(t)/A_0(t)$, $A_2(t)/A_1(t)$) (blue crosses). The solid line shows the fit from the label enrichment determination technique.

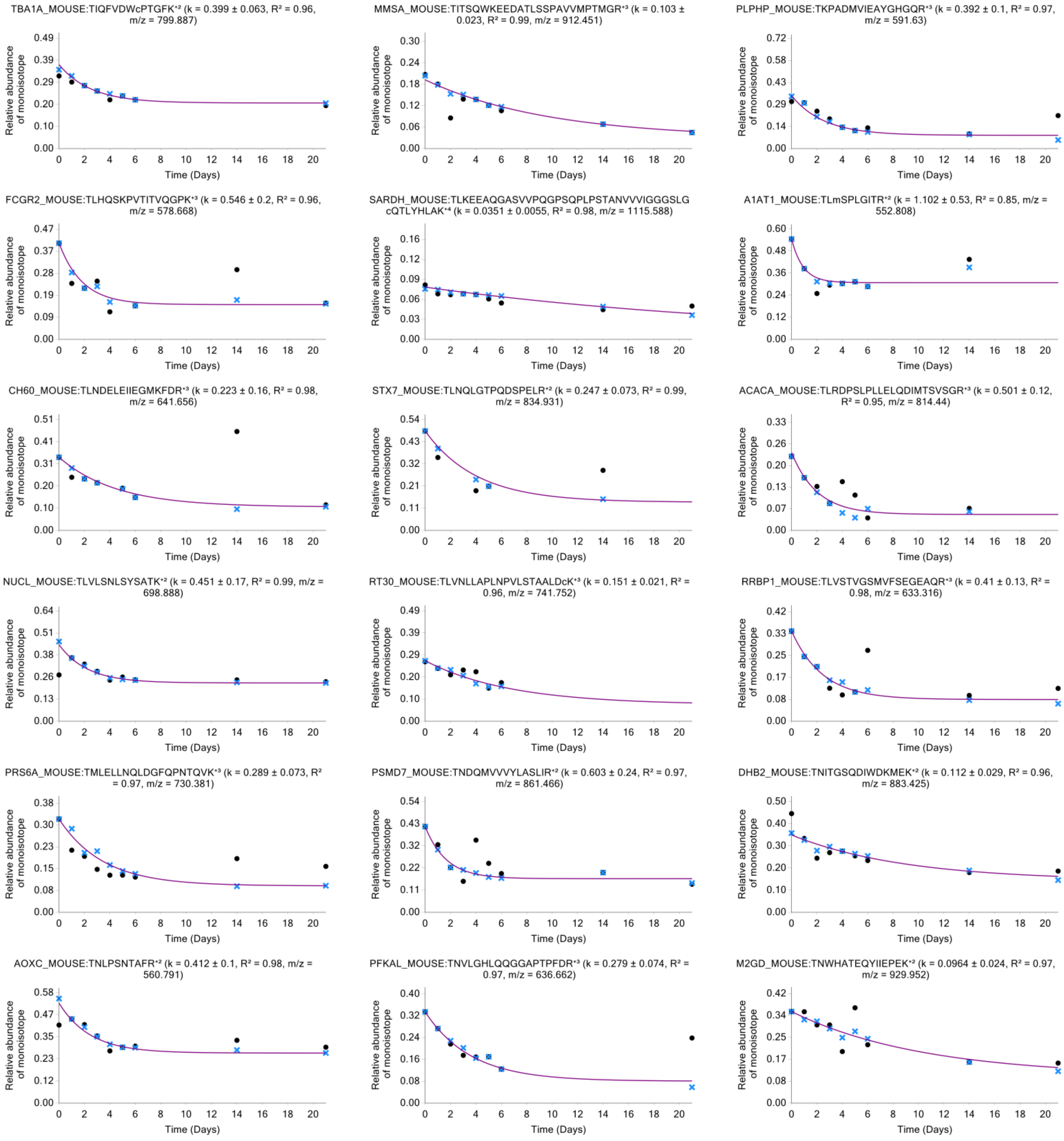

The Figures show the monoisotopic RIAs estimated from the complete isotope profile (black circles), RIA estimates from the complete isotope profiles, and any of the three partial isotope profiles combined ($A_1(t)/A_0(t)$, $A_2(t)/A_0(t)$, $A_2(t)/A_1(t)$) (blue crosses). The solid line shows the fit from the label enrichment determination technique.

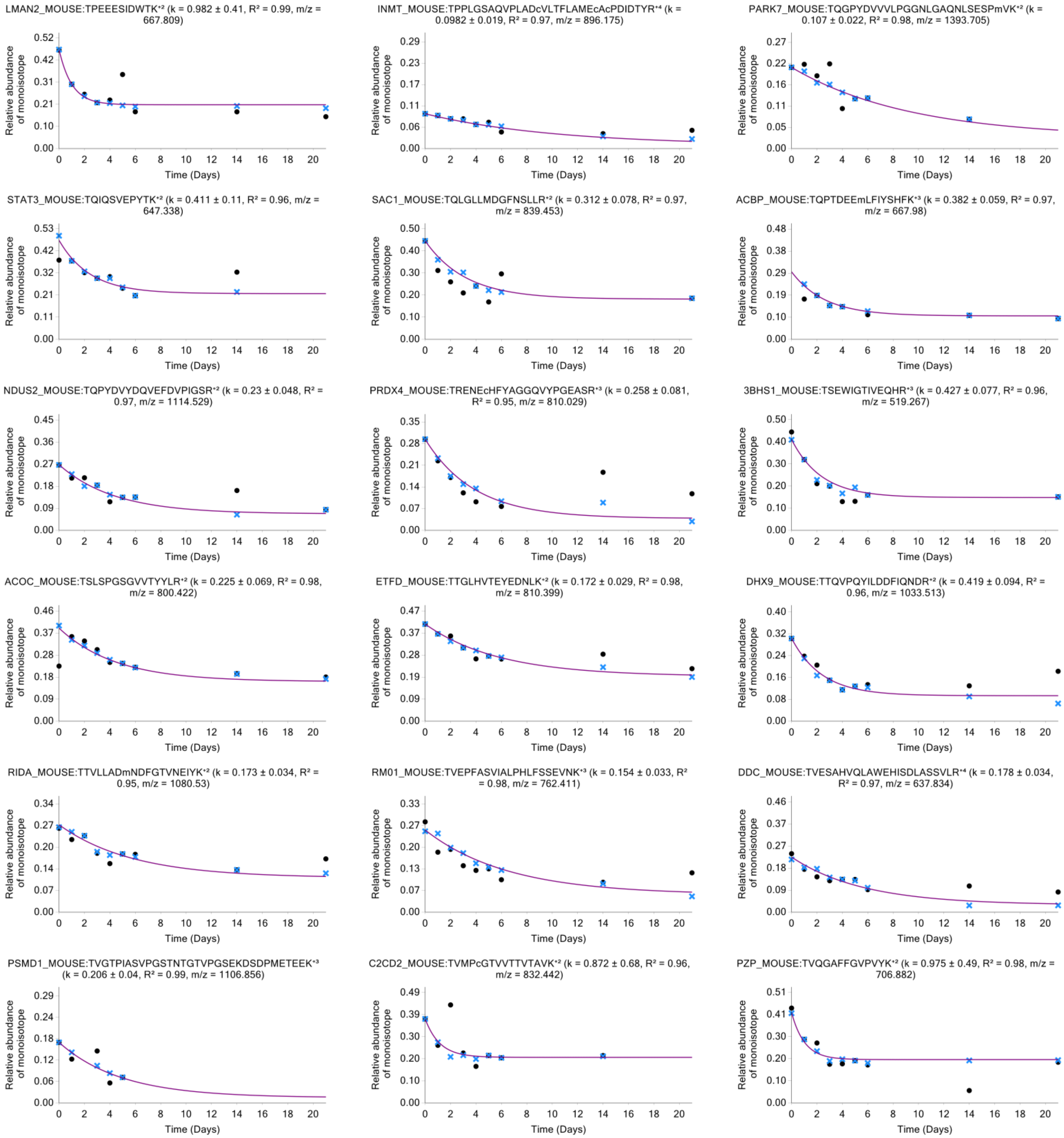

The Figures show the monoisotopic RIAs estimated from the complete isotope profile (black circles), RIA estimates from the complete isotope profiles, and any of the three partial isotope profiles combined ($A_1(t)/A_0(t)$, $A_2(t)/A_0(t)$, $A_2(t)/A_1(t)$) (blue crosses). The solid line shows the fit from the label enrichment determination technique.

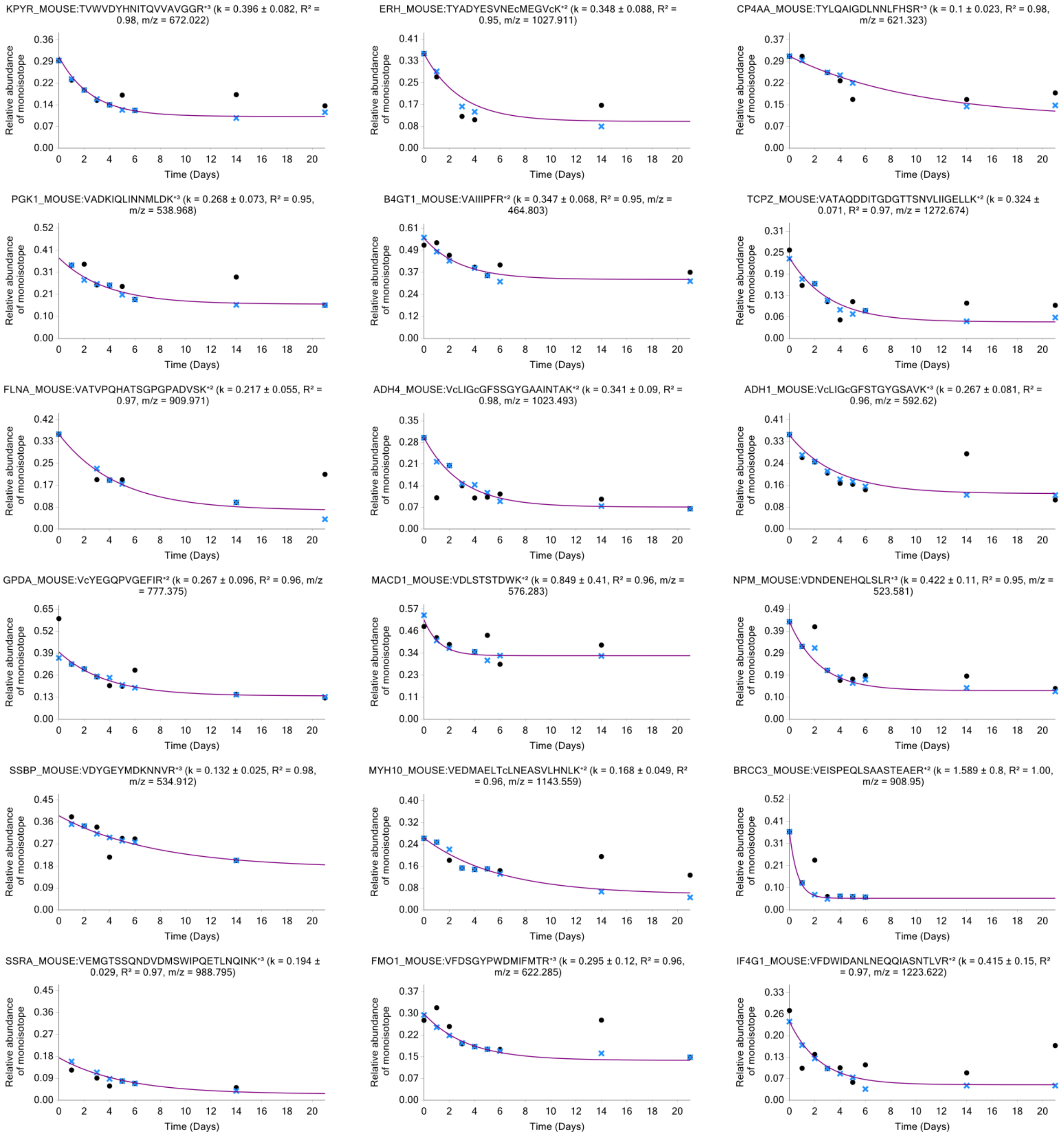

The Figures show the monoisotopic RIAs estimated from the complete isotope profile (black circles), RIA estimates from the complete isotope profiles, and any of the three partial isotope profiles combined ($A_1(t)/A_0(t)$, $A_2(t)/A_0(t)$, $A_2(t)/A_1(t)$) (blue crosses). The solid line shows the fit from the label enrichment determination technique.

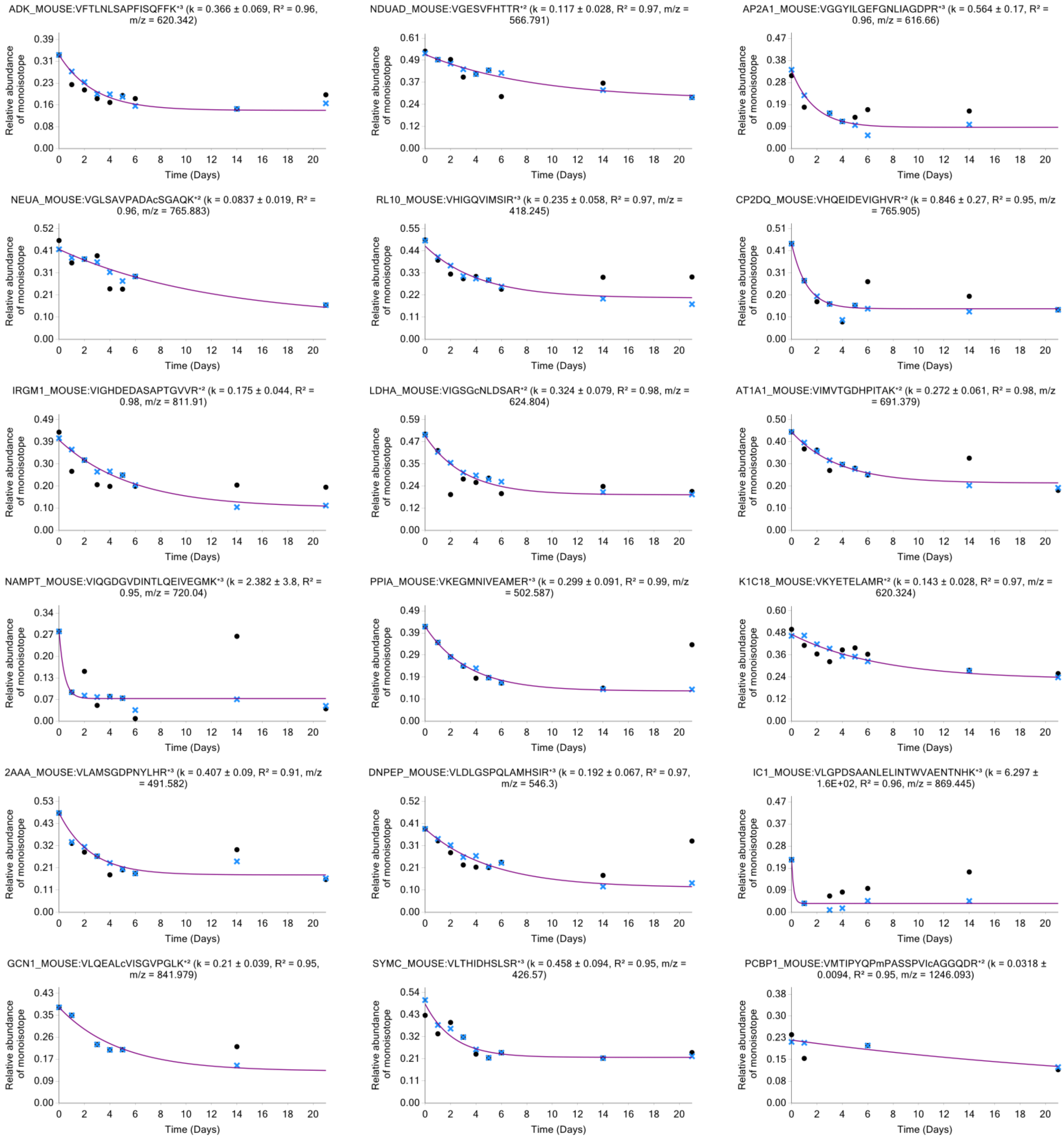

The Figures show the monoisotopic RIAs estimated from the complete isotope profile (black circles), RIA estimates from the complete isotope profiles, and any of the three partial isotope profiles combined ($A_1(t)/A_0(t)$, $A_2(t)/A_0(t)$, $A_2(t)/A_1(t)$) (blue crosses). The solid line shows the fit from the label enrichment determination technique.

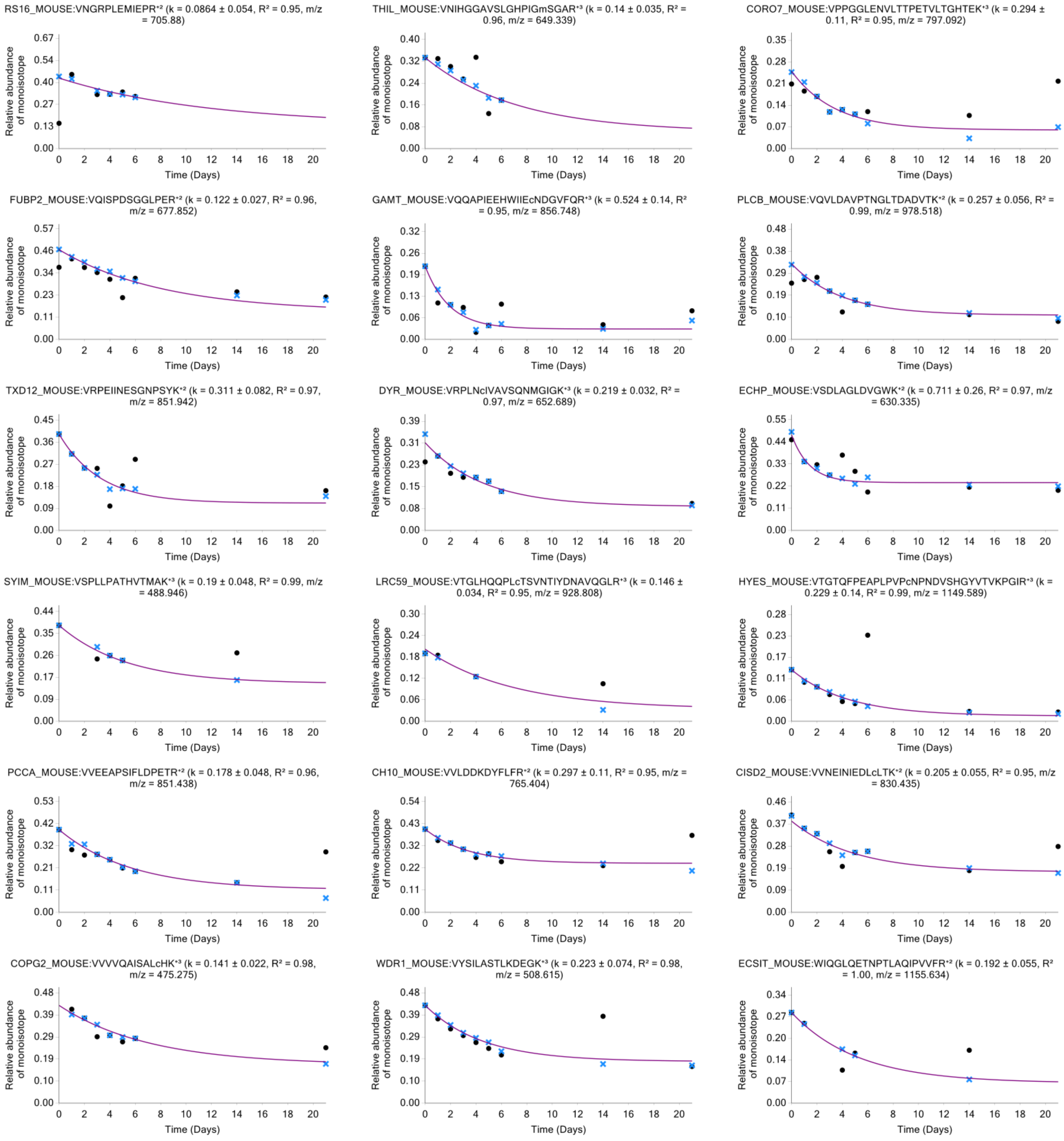

The Figures show the monoisotopic RIAs estimated from the complete isotope profile (black circles), RIA estimates from the complete isotope profiles, and any of the three partial isotope profiles combined ($A_1(t)/A_0(t)$, $A_2(t)/A_0(t)$, $A_2(t)/A_1(t)$) (blue crosses). The solid line shows the fit from the label enrichment determination technique.

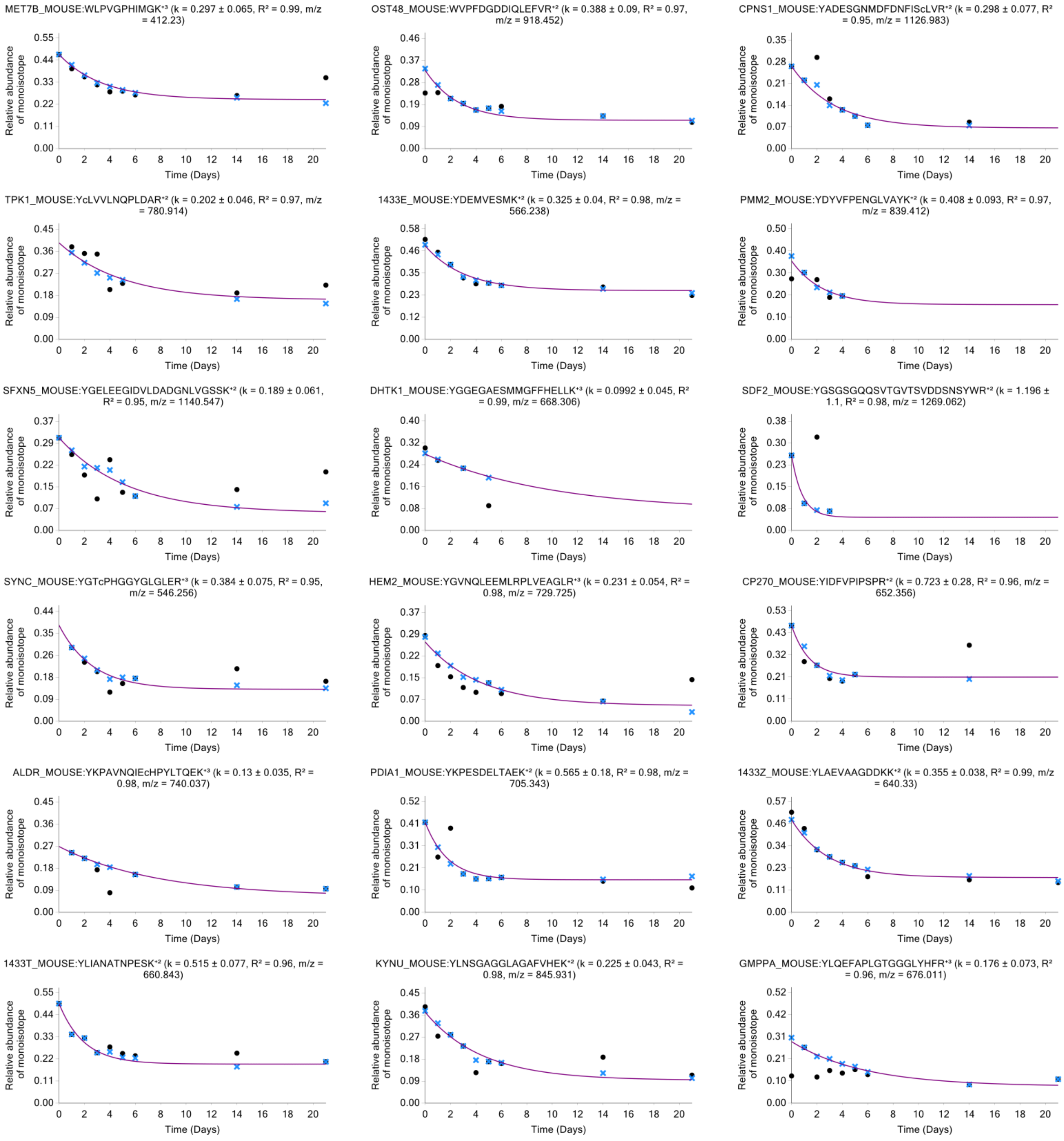

The Figures show the monoisotopic RIAs estimated from the complete isotope profile (black circles), RIA estimates from the complete isotope profiles, and any of the three partial isotope profiles combined ($A_1(t)/A_0(t)$, $A_2(t)/A_0(t)$, $A_2(t)/A_1(t)$) (blue crosses). The solid line shows the fit from the label enrichment determination technique.

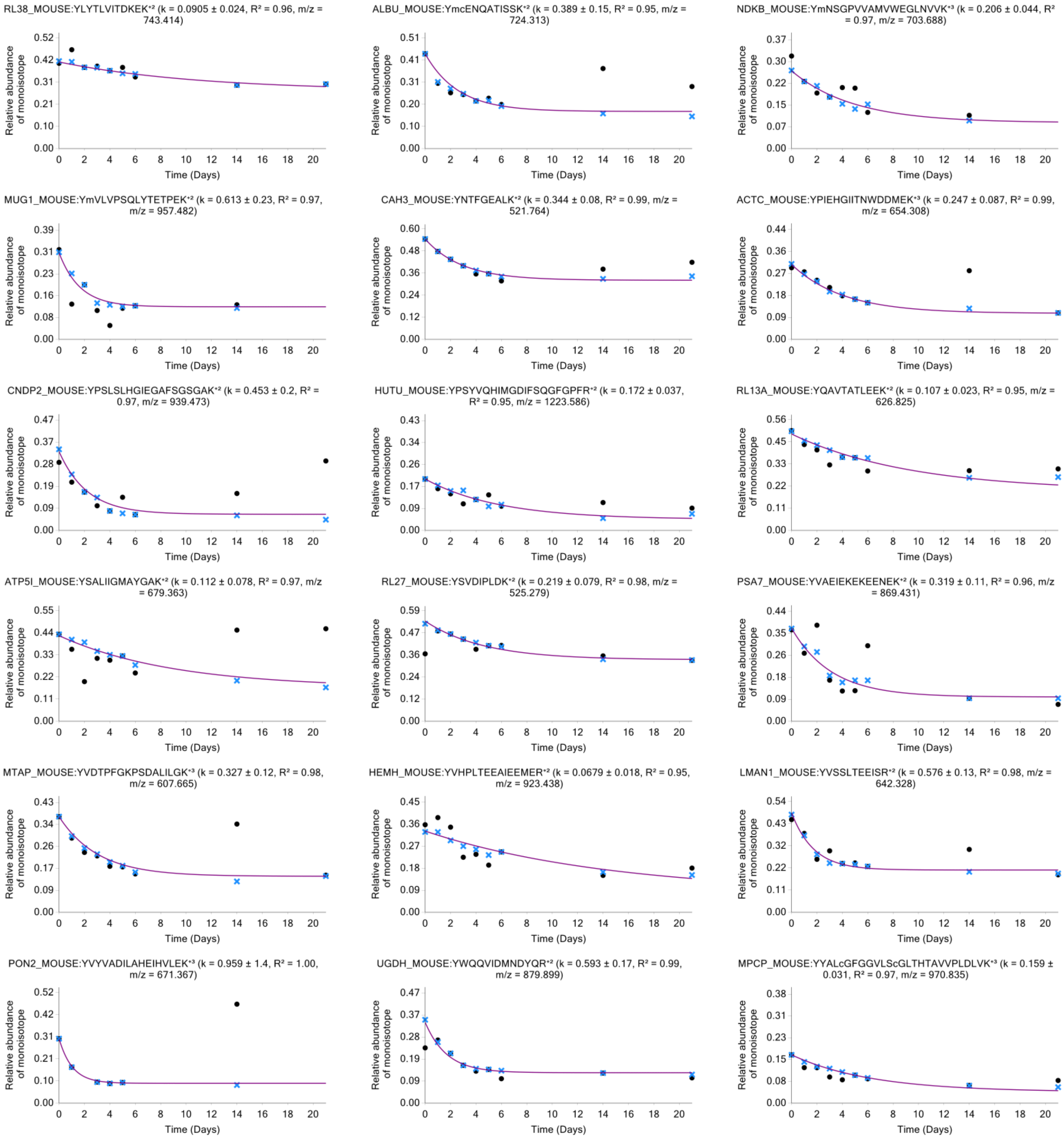

The Figures show the monoisotopic RIAs estimated from the complete isotope profile (black circles), RIA estimates from the complete isotope profiles, and any of the three partial isotope profiles combined ($A_1(t)/A_0(t)$, $A_2(t)/A_0(t)$, $A_2(t)/A_1(t)$) (blue crosses). The solid line shows the fit from the label enrichment determination technique.

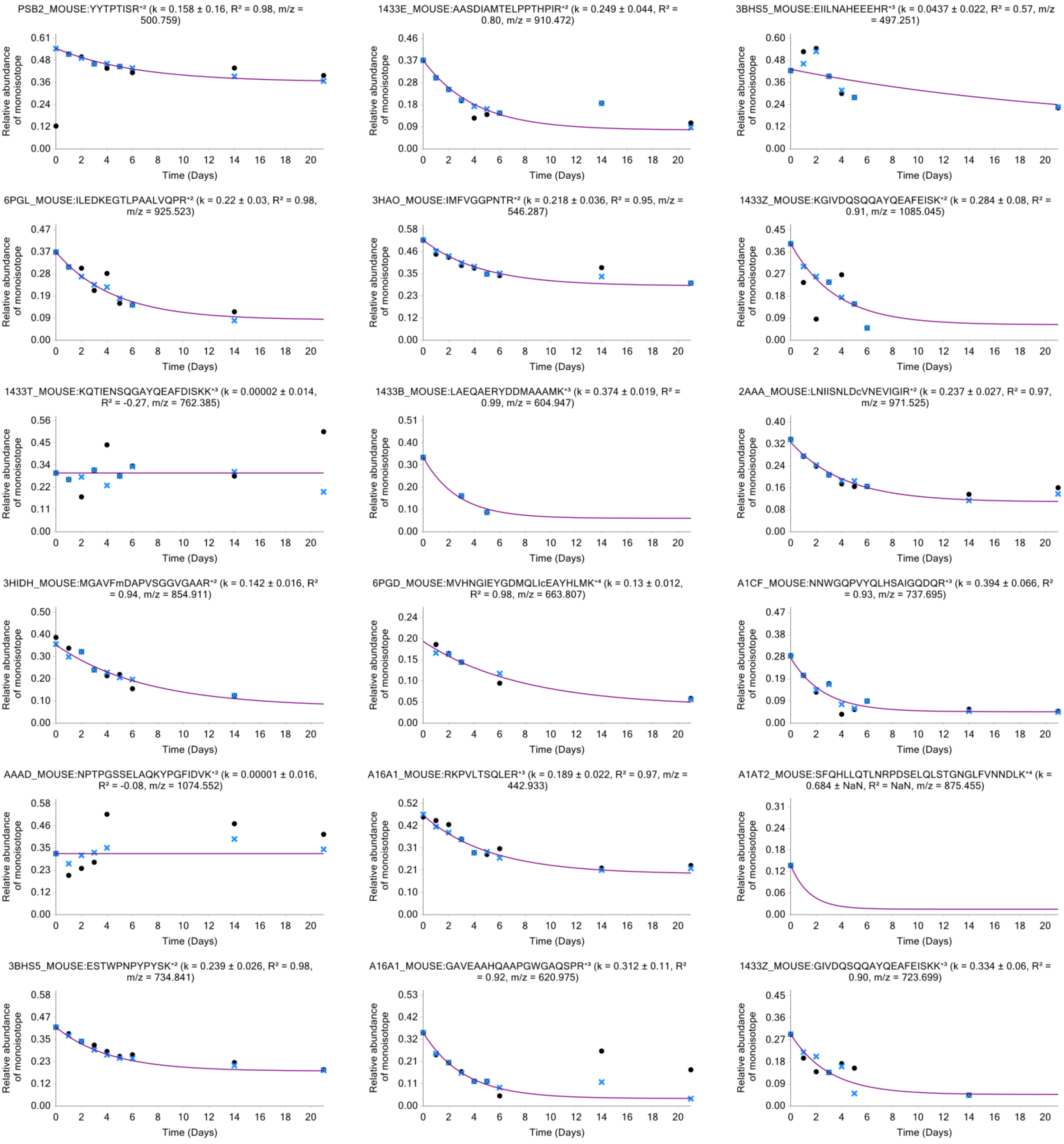

The Figures show the monoisotopic RIAs estimated from the complete isotope profile (black circles), RIA estimates from the complete isotope profiles, and any of the three partial isotope profiles combined ($A_1(t)/A_0(t)$, $A_2(t)/A_0(t)$, $A_2(t)/A_1(t)$) (blue crosses). The solid line shows the fit from the label enrichment determination technique.

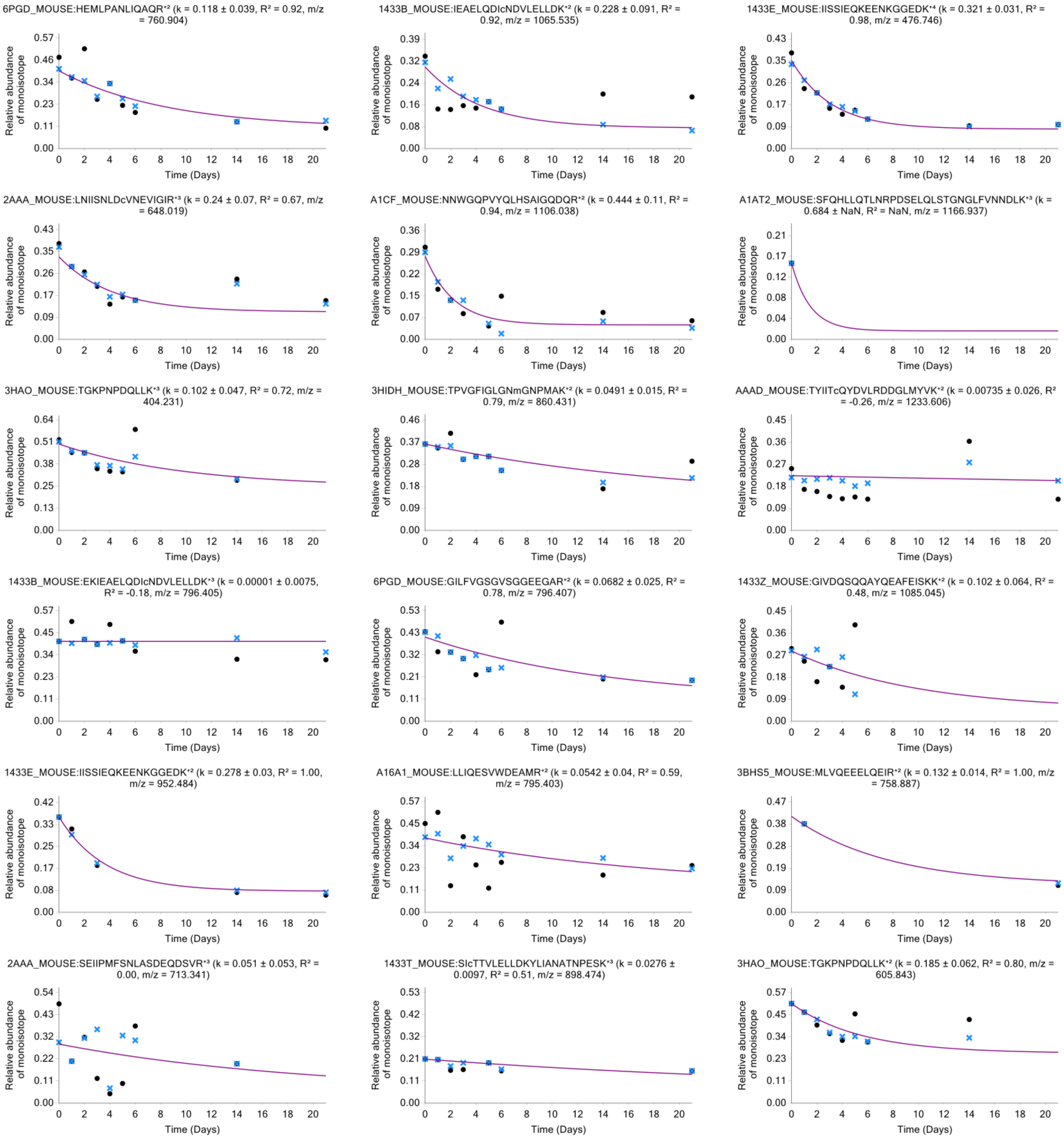

The Figures show the monoisotopic RIAs estimated from the complete isotope profile (black circles), RIA estimates from the complete isotope profiles, and any of the three partial isotope profiles combined ($A_1(t)/A_0(t)$, $A_2(t)/A_0(t)$, $A_2(t)/A_1(t)$) (blue crosses). The solid line shows the fit from the label enrichment determination technique.

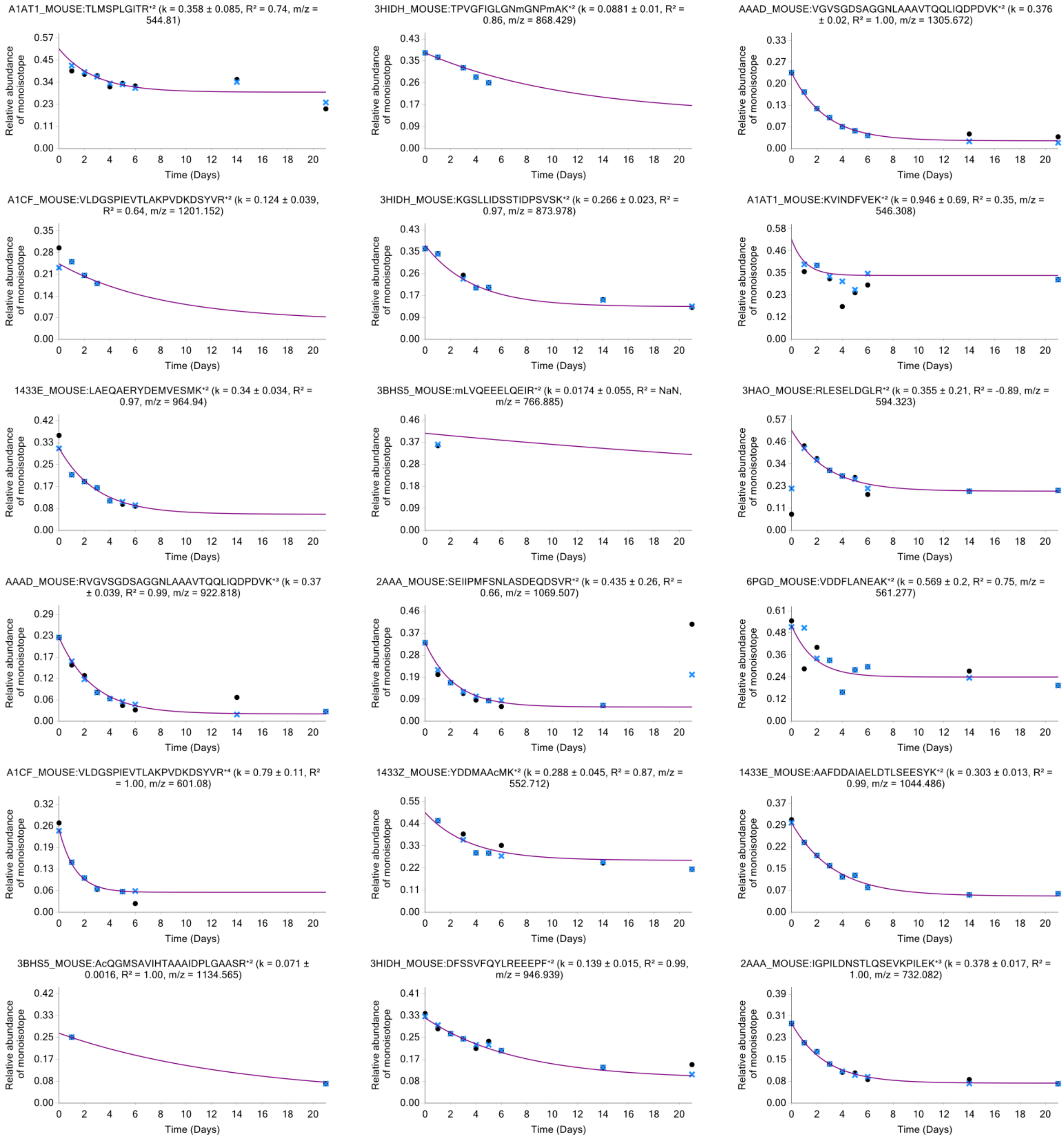

The Figures show the monoisotopic RIAs estimated from the complete isotope profile (black circles), RIA estimates from the complete isotope profiles, and any of the three partial isotope profiles combined ($A_1(t)/A_0(t)$, $A_2(t)/A_0(t)$, $A_2(t)/A_1(t)$) (blue crosses). The solid line shows the fit from the label enrichment determination technique.

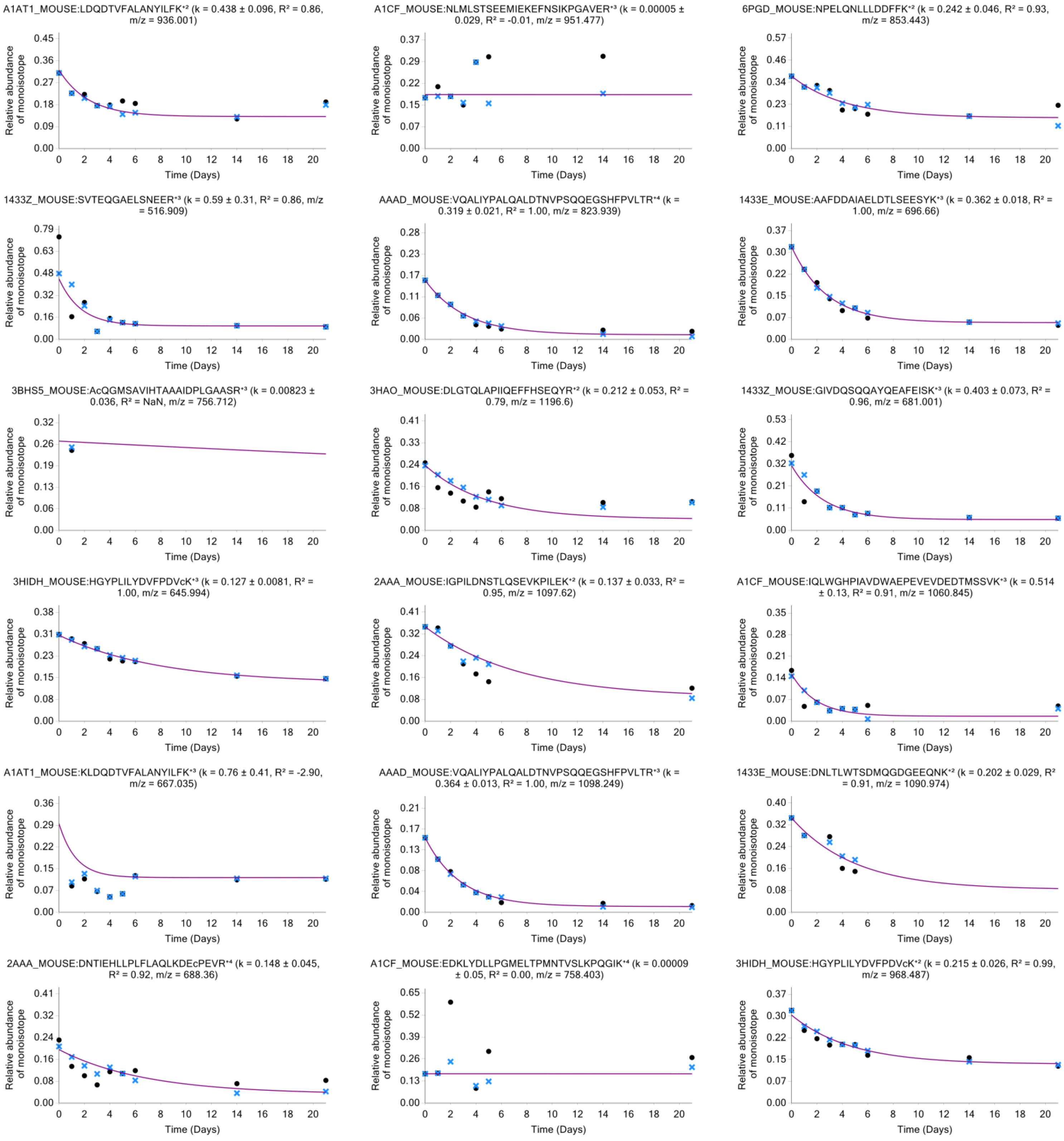

The Figures show the monoisotopic RIAs estimated from the complete isotope profile (black circles), RIA estimates from the complete isotope profiles, and any of the three partial isotope profiles combined ($A_1(t)/A_0(t)$, $A_2(t)/A_0(t)$, $A_2(t)/A_1(t)$) (blue crosses). The solid line shows the fit from the label enrichment determination technique.

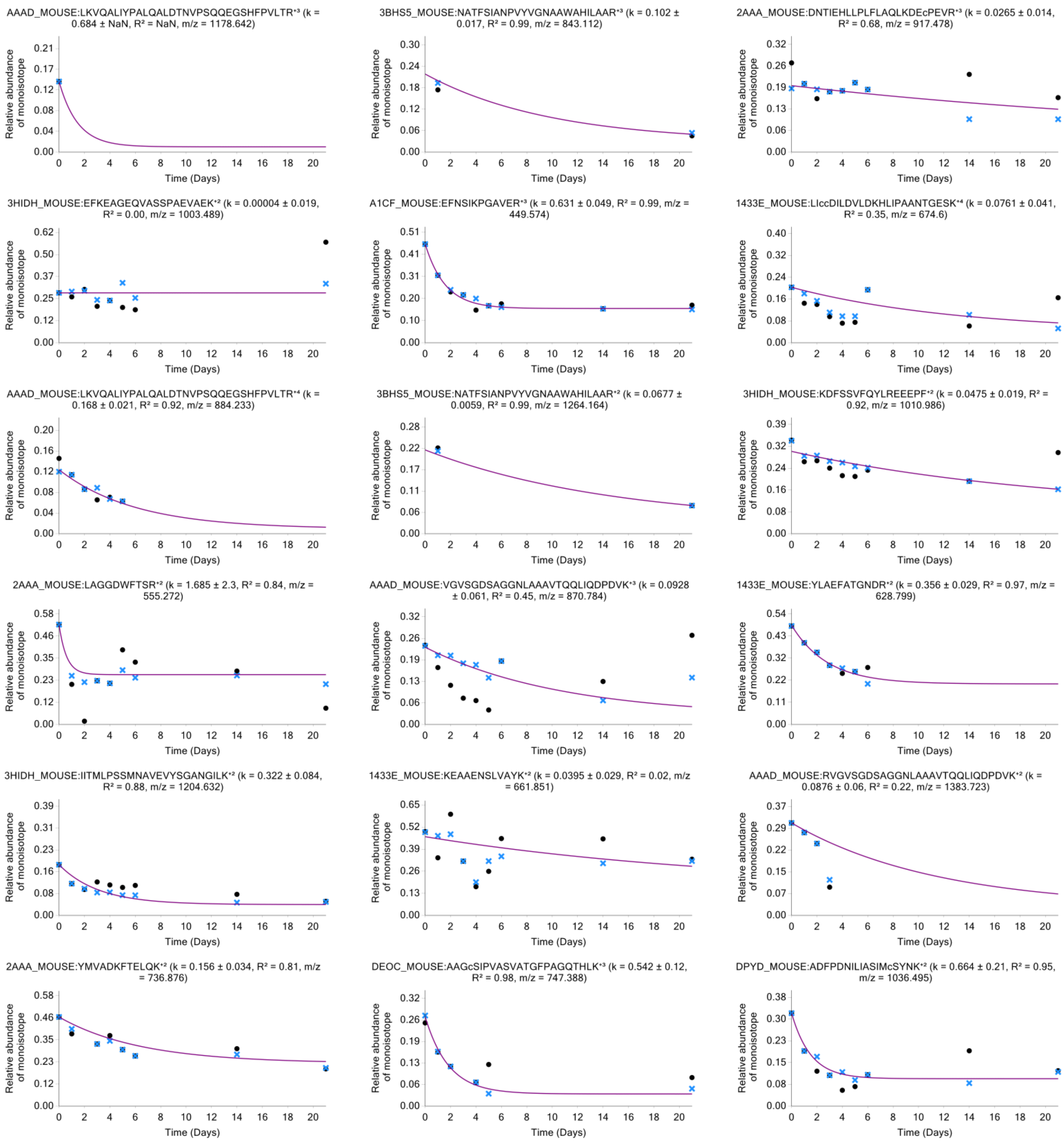

The Figures show the monoisotopic RIAs estimated from the complete isotope profile (black circles), RIA estimates from the complete isotope profiles, and any of the three partial isotope profiles combined ($A_1(t)/A_0(t)$, $A_2(t)/A_0(t)$, $A_2(t)/A_1(t)$) (blue crosses). The solid line shows the fit from the label enrichment determination technique.

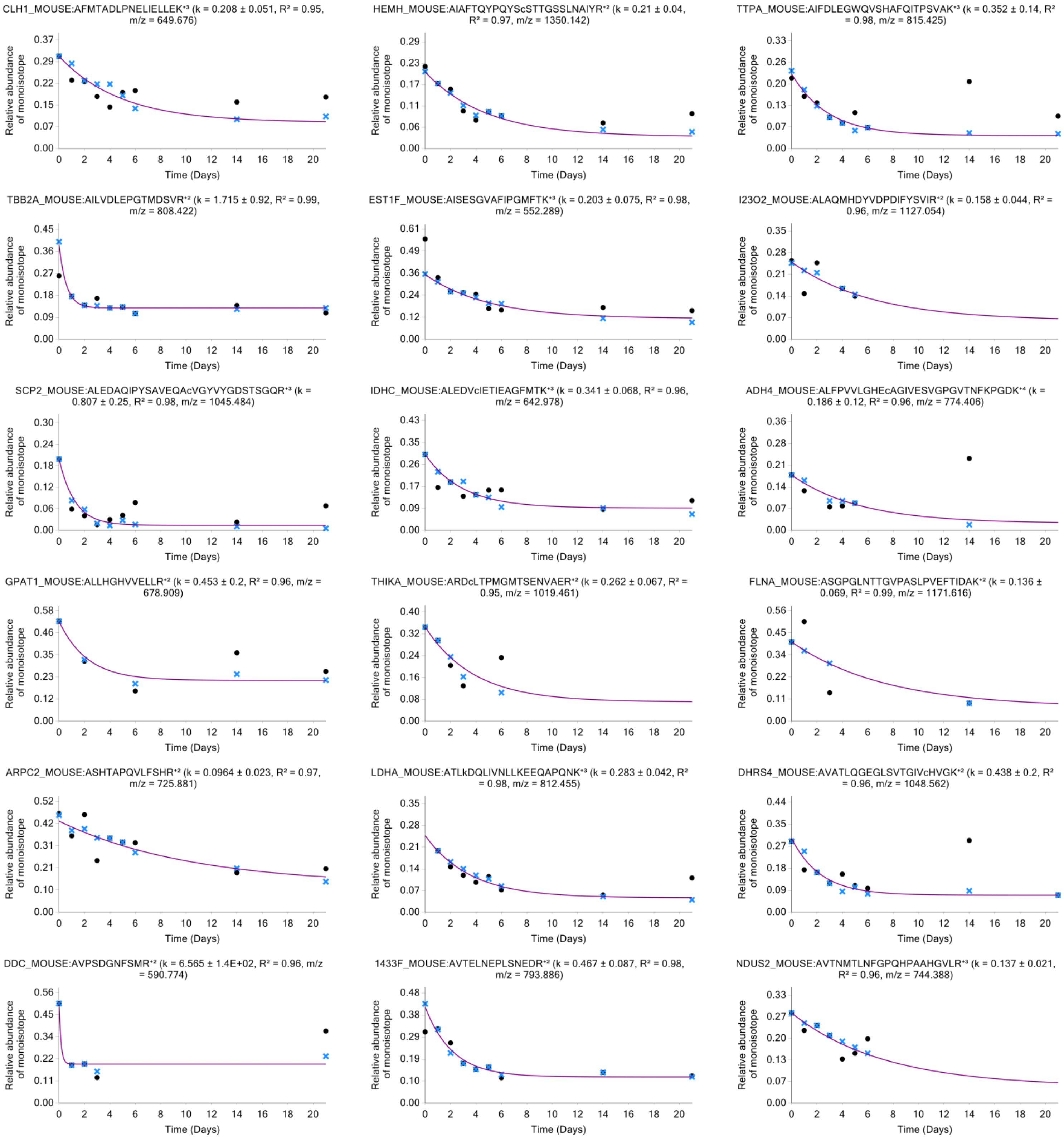

The Figures show the monoisotopic RIAs estimated from the complete isotope profile (black circles), RIA estimates from the complete isotope profiles, and any of the three partial isotope profiles combined ($A_1(t)/A_0(t)$, $A_2(t)/A_0(t)$, $A_2(t)/A_1(t)$) (blue crosses). The solid line shows the fit from the label enrichment determination technique.

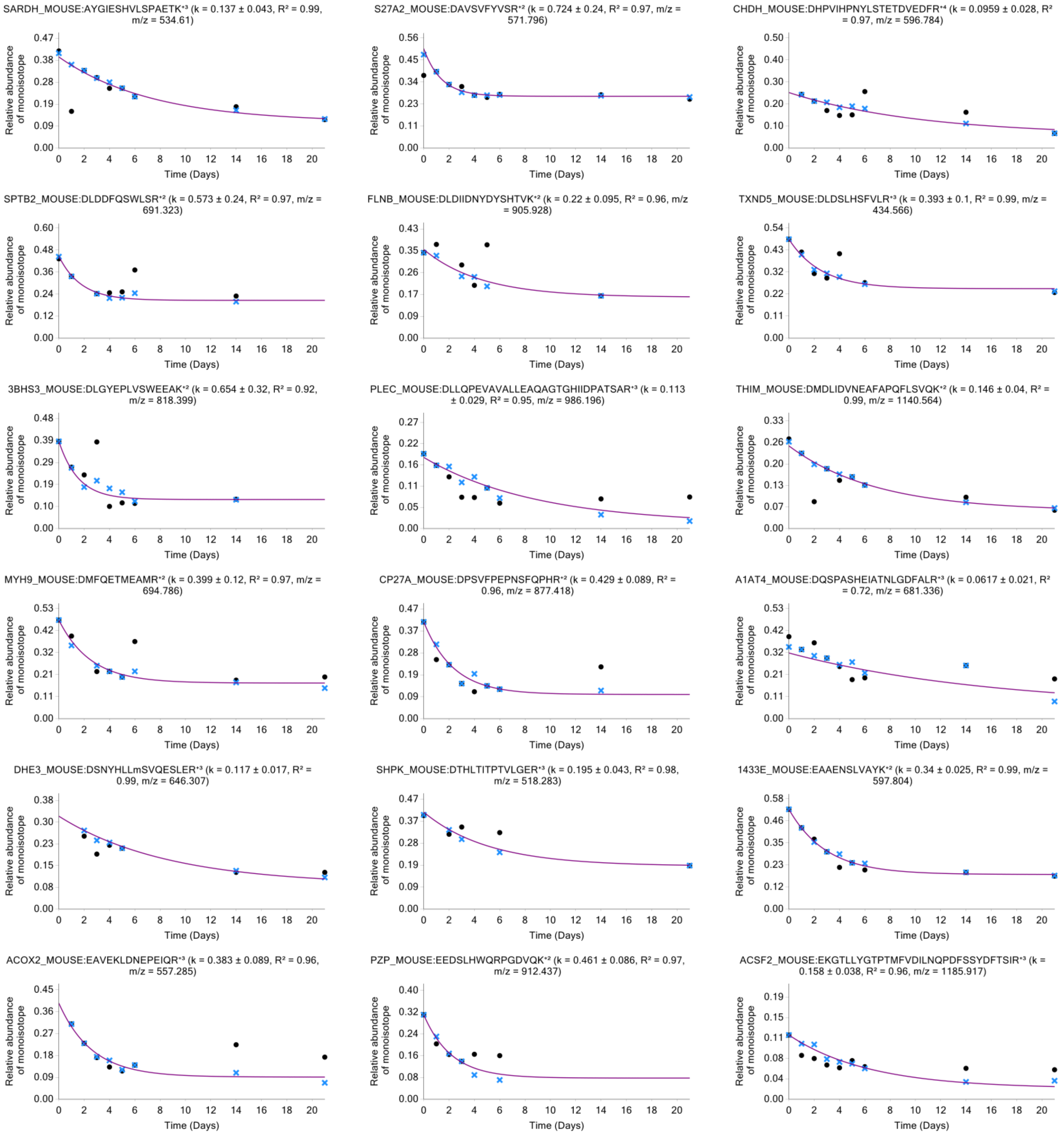

The Figures show the monoisotopic RIAs estimated from the complete isotope profile (black circles), RIA estimates from the complete isotope profiles, and any of the three partial isotope profiles combined ($A_1(t)/A_0(t)$, $A_2(t)/A_0(t)$, $A_2(t)/A_1(t)$) (blue crosses). The solid line shows the fit from the label enrichment determination technique.

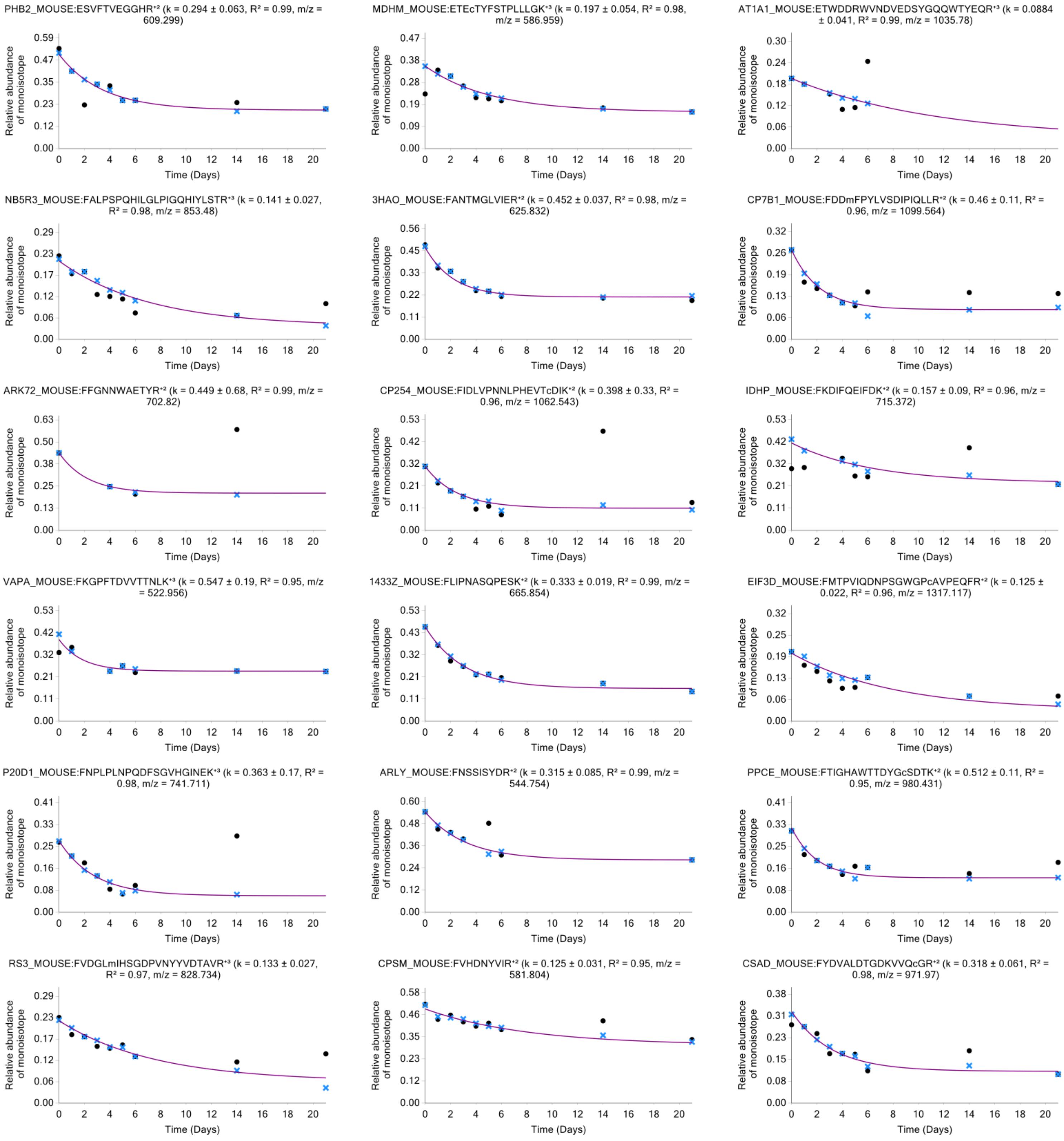

The Figures show the monoisotopic RIAs estimated from the complete isotope profile (black circles), RIA estimates from the complete isotope profiles, and any of the three partial isotope profiles combined ($A_1(t)/A_0(t)$, $A_2(t)/A_0(t)$, $A_2(t)/A_1(t)$) (blue crosses). The solid line shows the fit from the label enrichment determination technique.

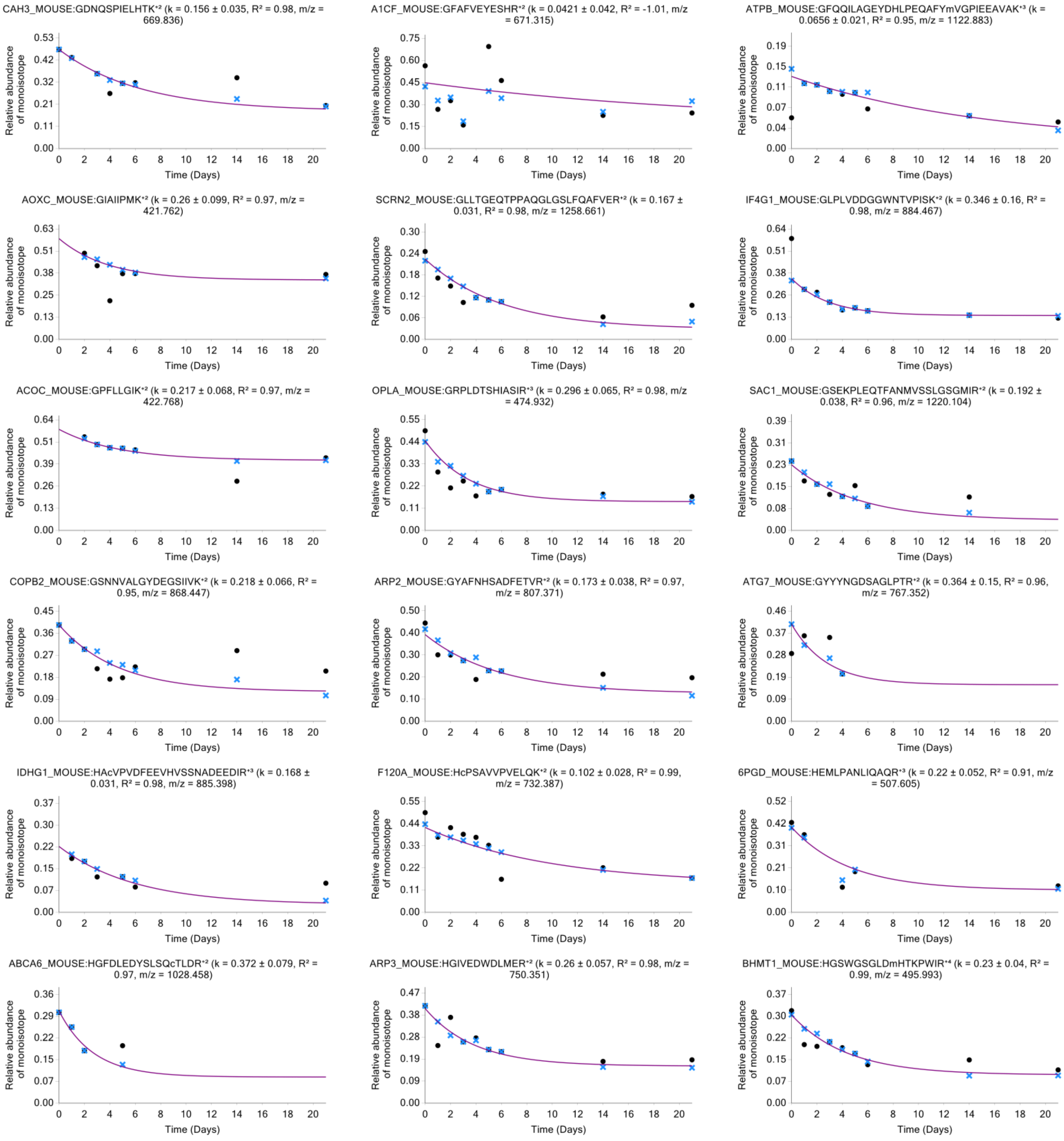

The Figures show the monoisotopic RIAs estimated from the complete isotope profile (black circles), RIA estimates from the complete isotope profiles, and any of the three partial isotope profiles combined ($A_1(t)/A_0(t)$, $A_2(t)/A_0(t)$, $A_2(t)/A_1(t)$) (blue crosses). The solid line shows the fit from the label enrichment determination technique.

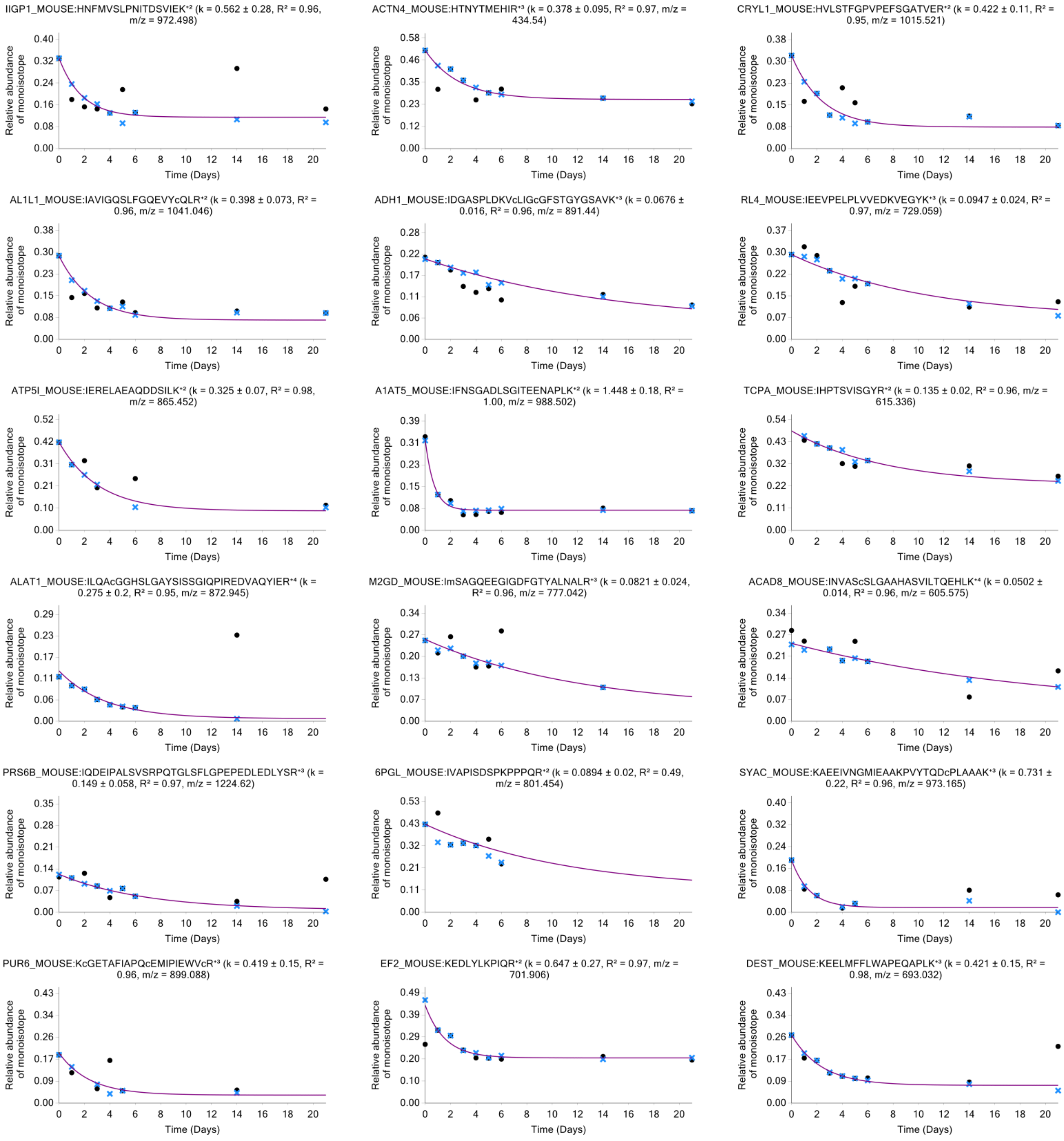

The Figures show the monoisotopic RIAs estimated from the complete isotope profile (black circles), RIA estimates from the complete isotope profiles, and any of the three partial isotope profiles combined ($A_1(t)/A_0(t)$, $A_2(t)/A_0(t)$, $A_2(t)/A_1(t)$) (blue crosses). The solid line shows the fit from the label enrichment determination technique.

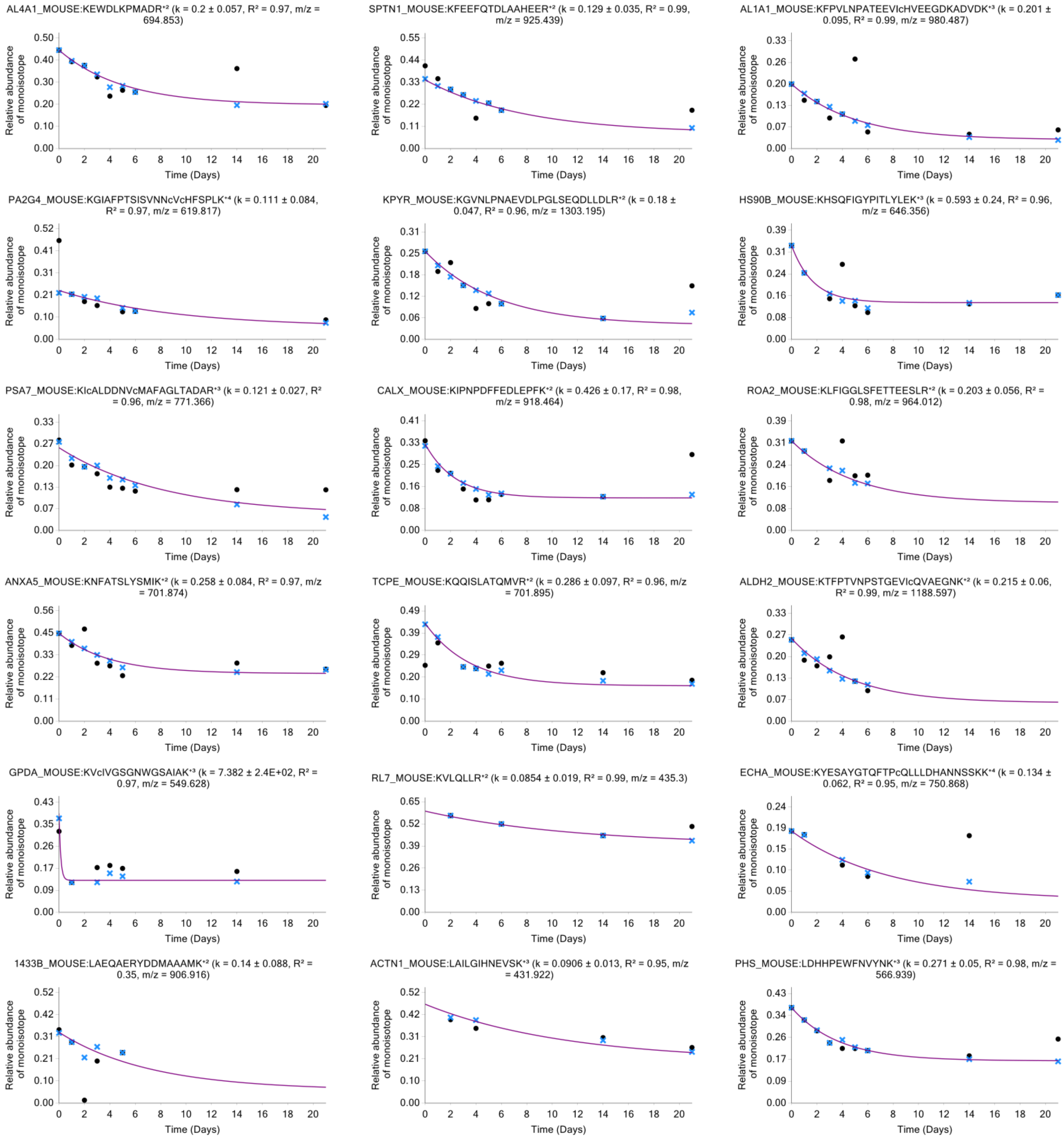

The Figures show the monoisotopic RIAs estimated from the complete isotope profile (black circles), RIA estimates from the complete isotope profiles, and any of the three partial isotope profiles combined ($A_1(t)/A_0(t)$, $A_2(t)/A_0(t)$, $A_2(t)/A_1(t)$) (blue crosses). The solid line shows the fit from the label enrichment determination technique.

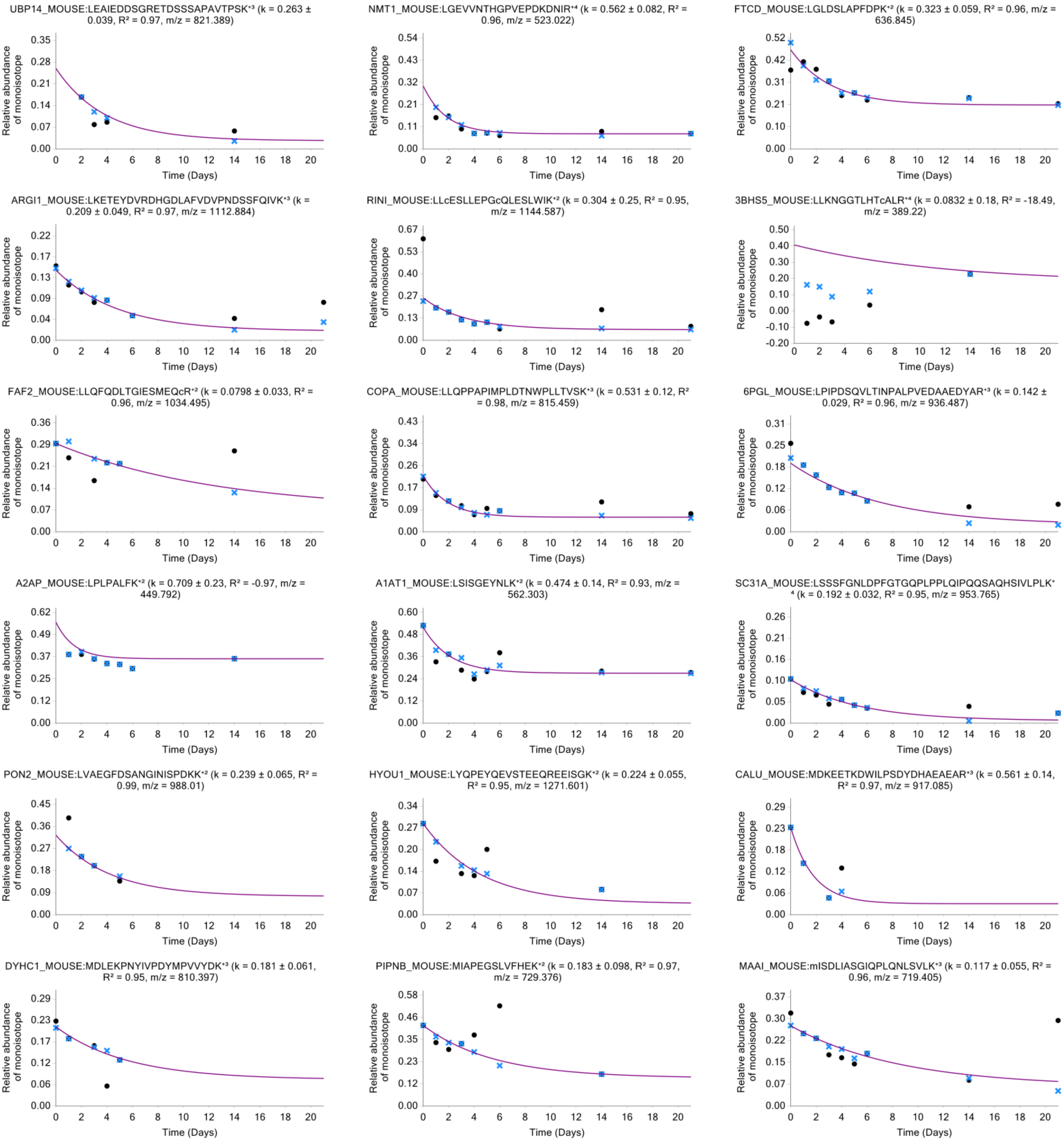

The Figures show the monoisotopic RIAs estimated from the complete isotope profile (black circles), RIA estimates from the complete isotope profiles, and any of the three partial isotope profiles combined ($A_1(t)/A_0(t)$, $A_2(t)/A_0(t)$, $A_2(t)/A_1(t)$) (blue crosses). The solid line shows the fit from the label enrichment determination technique.

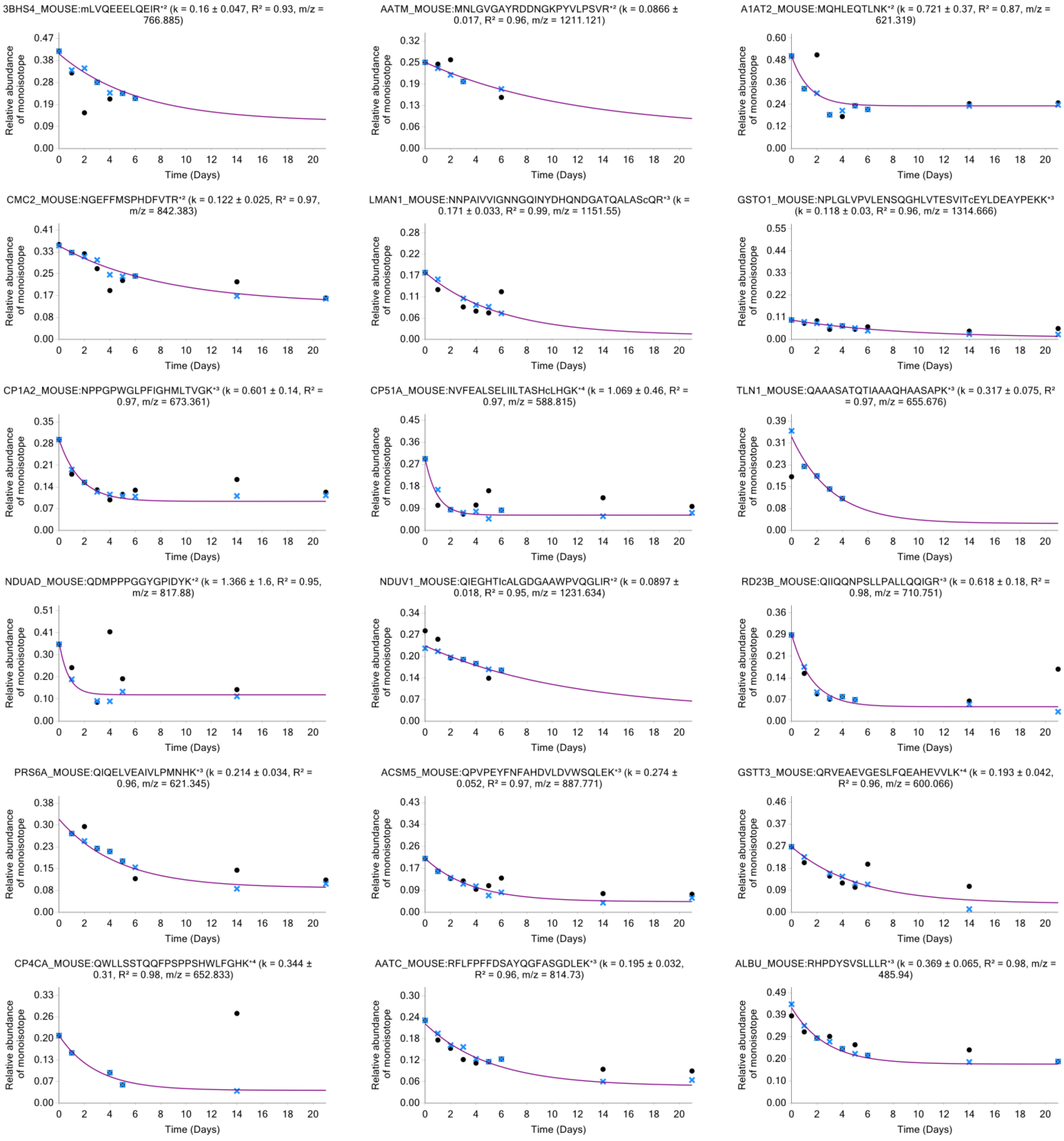

The Figures show the monoisotopic RIAs estimated from the complete isotope profile (black circles), RIA estimates from the complete isotope profiles, and any of the three partial isotope profiles combined ($A_1(t)/A_0(t)$, $A_2(t)/A_0(t)$, $A_2(t)/A_1(t)$) (blue crosses). The solid line shows the fit from the label enrichment determination technique.

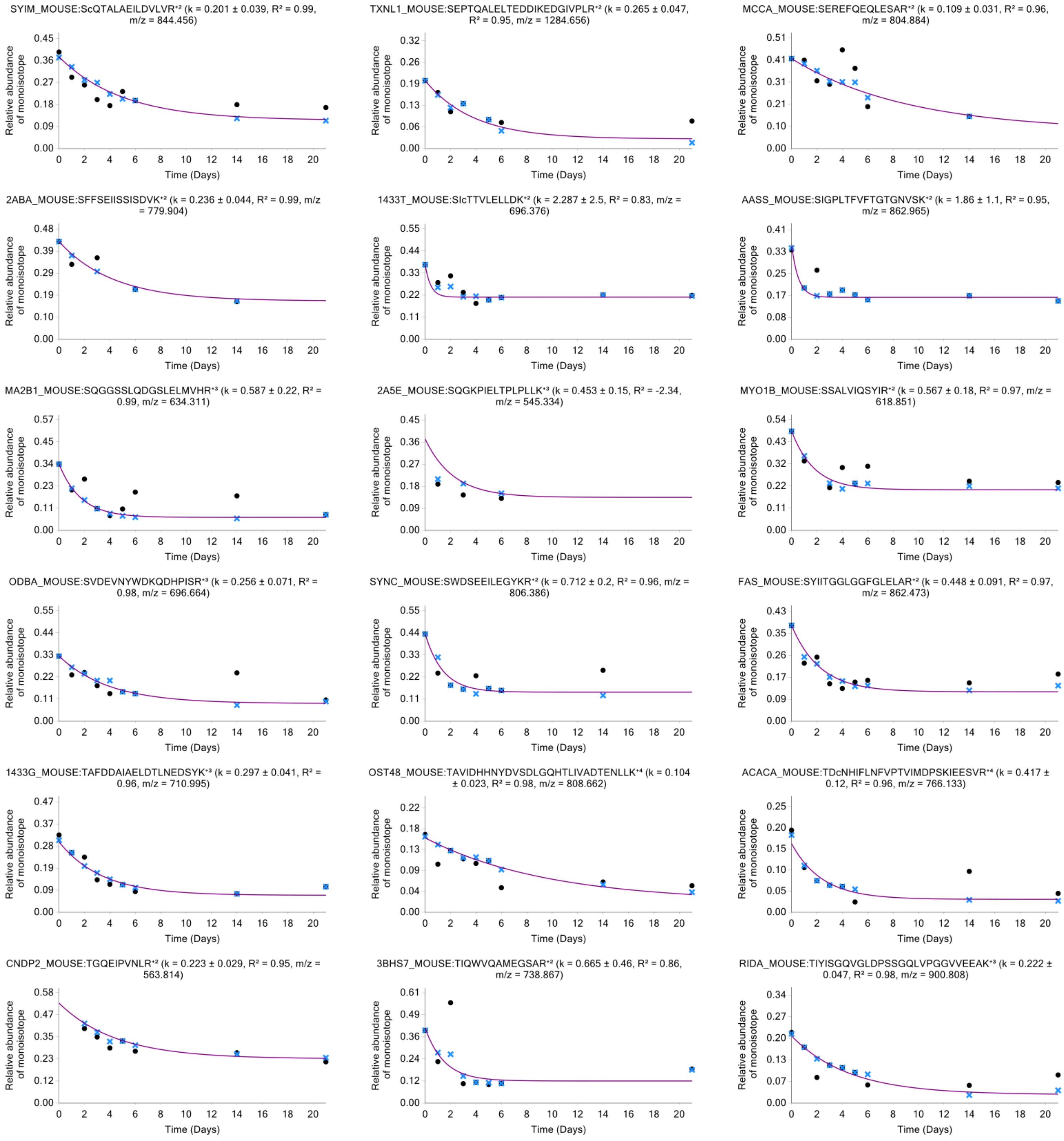

The Figures show the monoisotopic RIAs estimated from the complete isotope profile (black circles), RIA estimates from the complete isotope profiles, and any of the three partial isotope profiles combined ($A_1(t)/A_0(t)$, $A_2(t)/A_0(t)$, $A_2(t)/A_1(t)$) (blue crosses). The solid line shows the fit from the label enrichment determination technique.

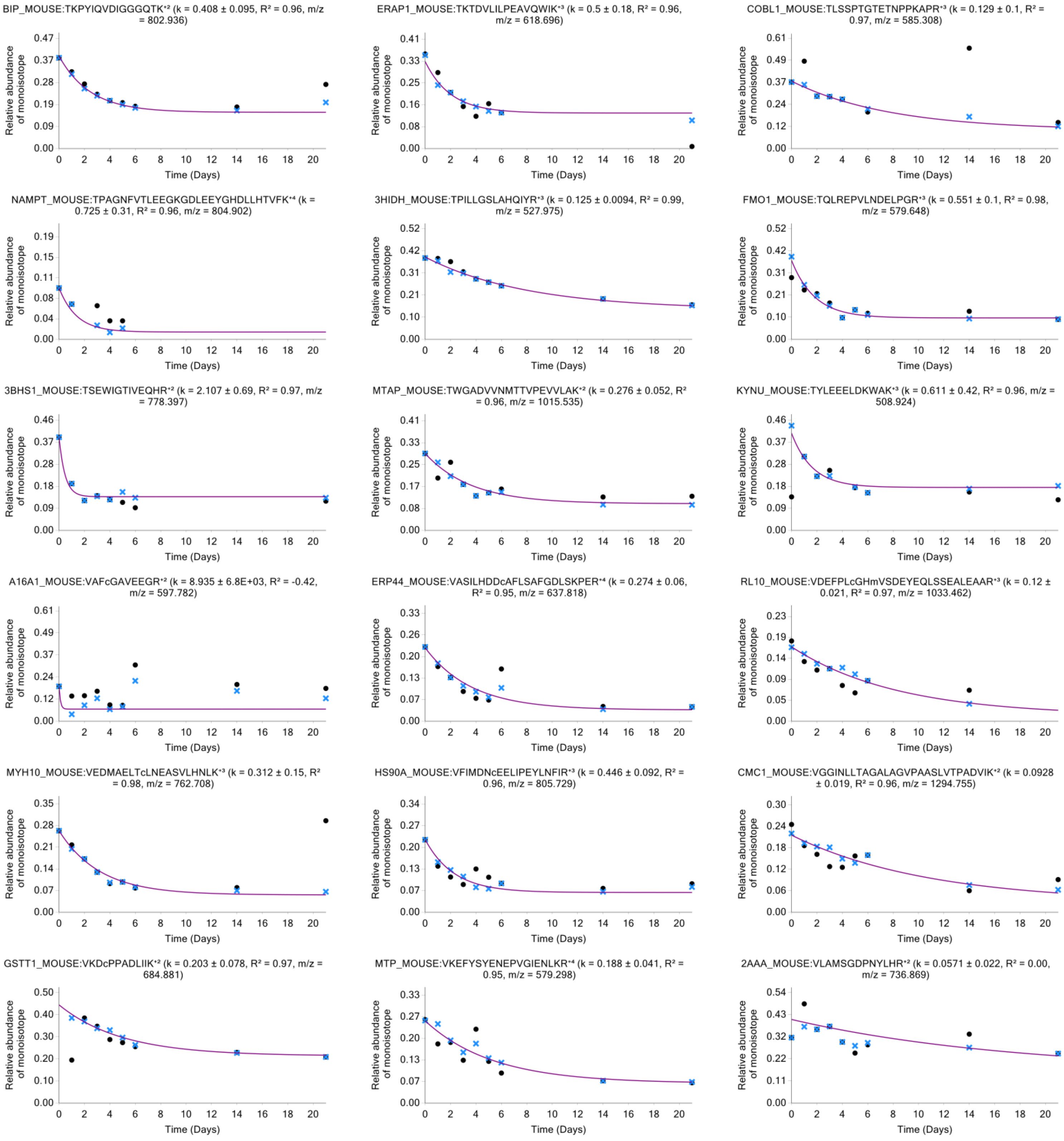

The Figures show the monoisotopic RIAs estimated from the complete isotope profile (black circles), RIA estimates from the complete isotope profiles, and any of the three partial isotope profiles combined ($A_1(t)/A_0(t)$, $A_2(t)/A_0(t)$, $A_2(t)/A_1(t)$) (blue crosses). The solid line shows the fit from the label enrichment determination technique.

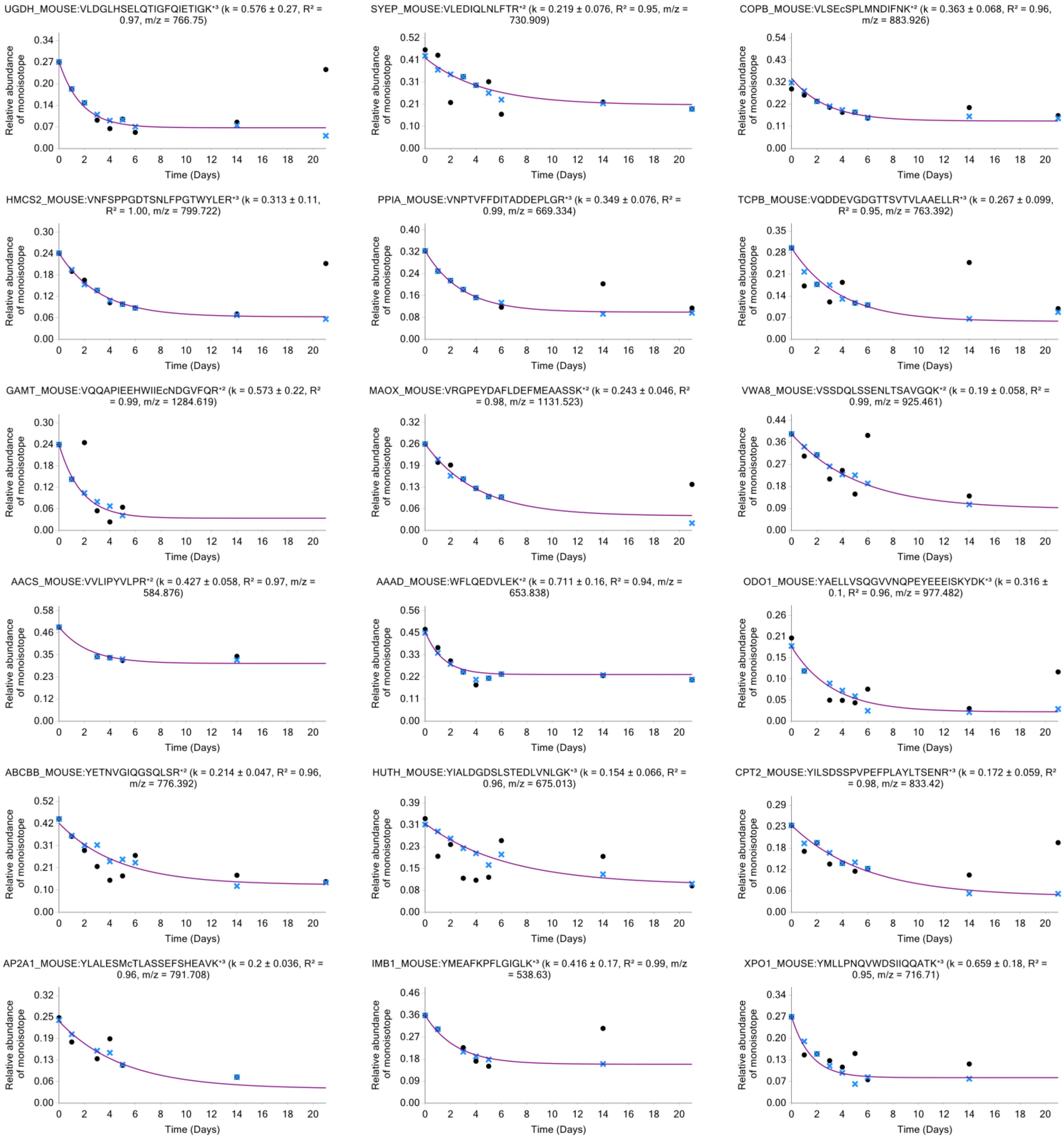

The Figures show the monoisotopic RIAs estimated from the complete isotope profile (black circles), RIA estimates from the complete isotope profiles, and any of the three partial isotope profiles combined ($A_1(t)/A_0(t)$, $A_2(t)/A_0(t)$, $A_2(t)/A_1(t)$) (blue crosses). The solid line shows the fit from the label enrichment determination technique.

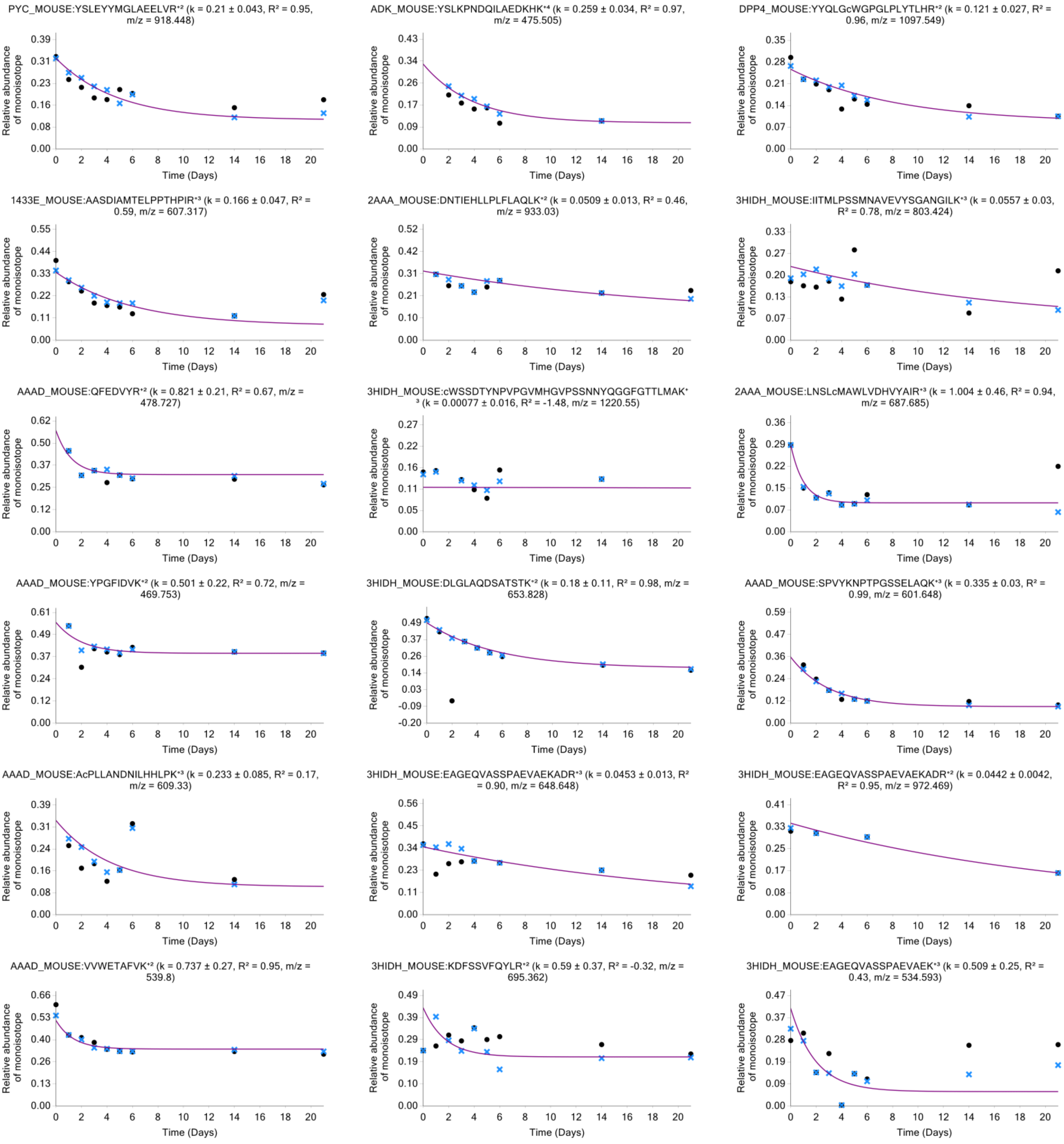

The Figures show the monoisotopic RIAs estimated from the complete isotope profile (black circles), RIA estimates from the complete isotope profiles, and any of the three partial isotope profiles combined ($A_1(t)/A_0(t)$, $A_2(t)/A_0(t)$, $A_2(t)/A_1(t)$) (blue crosses). The solid line shows the fit from the label enrichment determination technique.

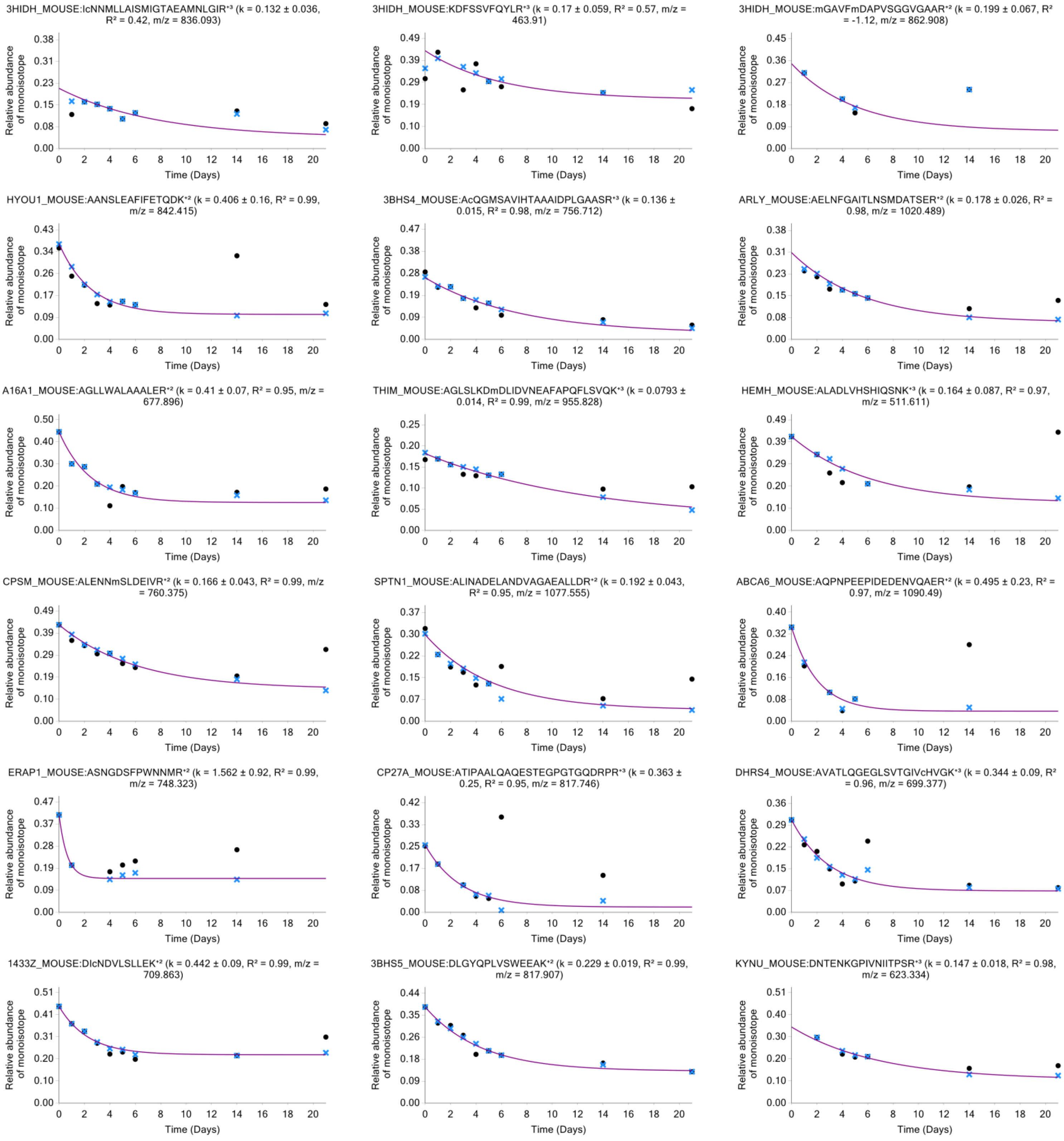

The Figures show the monoisotopic RIAs estimated from the complete isotope profile (black circles), RIA estimates from the complete isotope profiles, and any of the three partial isotope profiles combined ($A_1(t)/A_0(t)$, $A_2(t)/A_0(t)$, $A_2(t)/A_1(t)$) (blue crosses). The solid line shows the fit from the label enrichment determination technique.

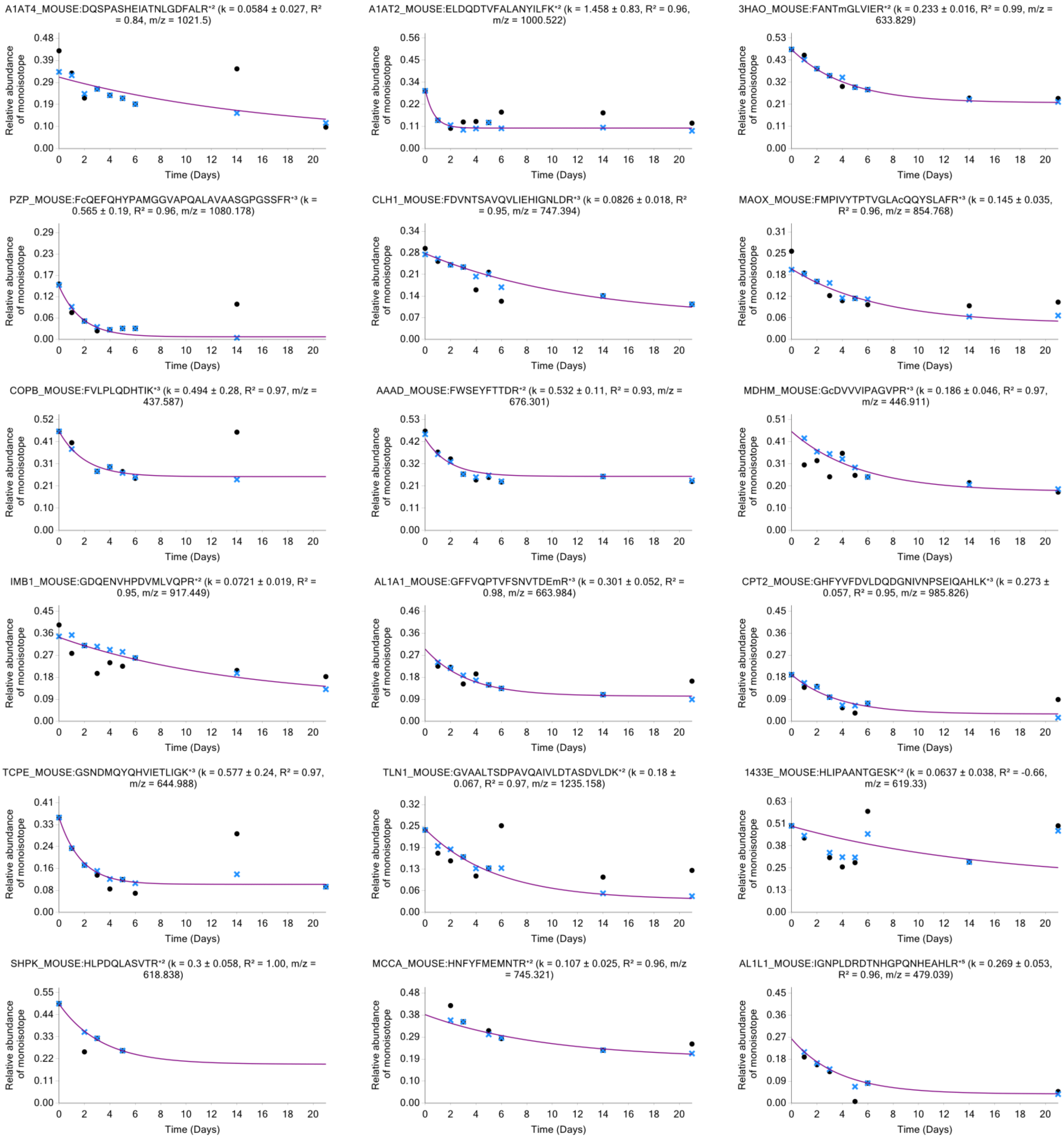

The Figures show the monoisotopic RIAs estimated from the complete isotope profile (black circles), RIA estimates from the complete isotope profiles, and any of the three partial isotope profiles combined ($A_1(t)/A_0(t)$, $A_2(t)/A_0(t)$, $A_2(t)/A_1(t)$) (blue crosses). The solid line shows the fit from the label enrichment determination technique.

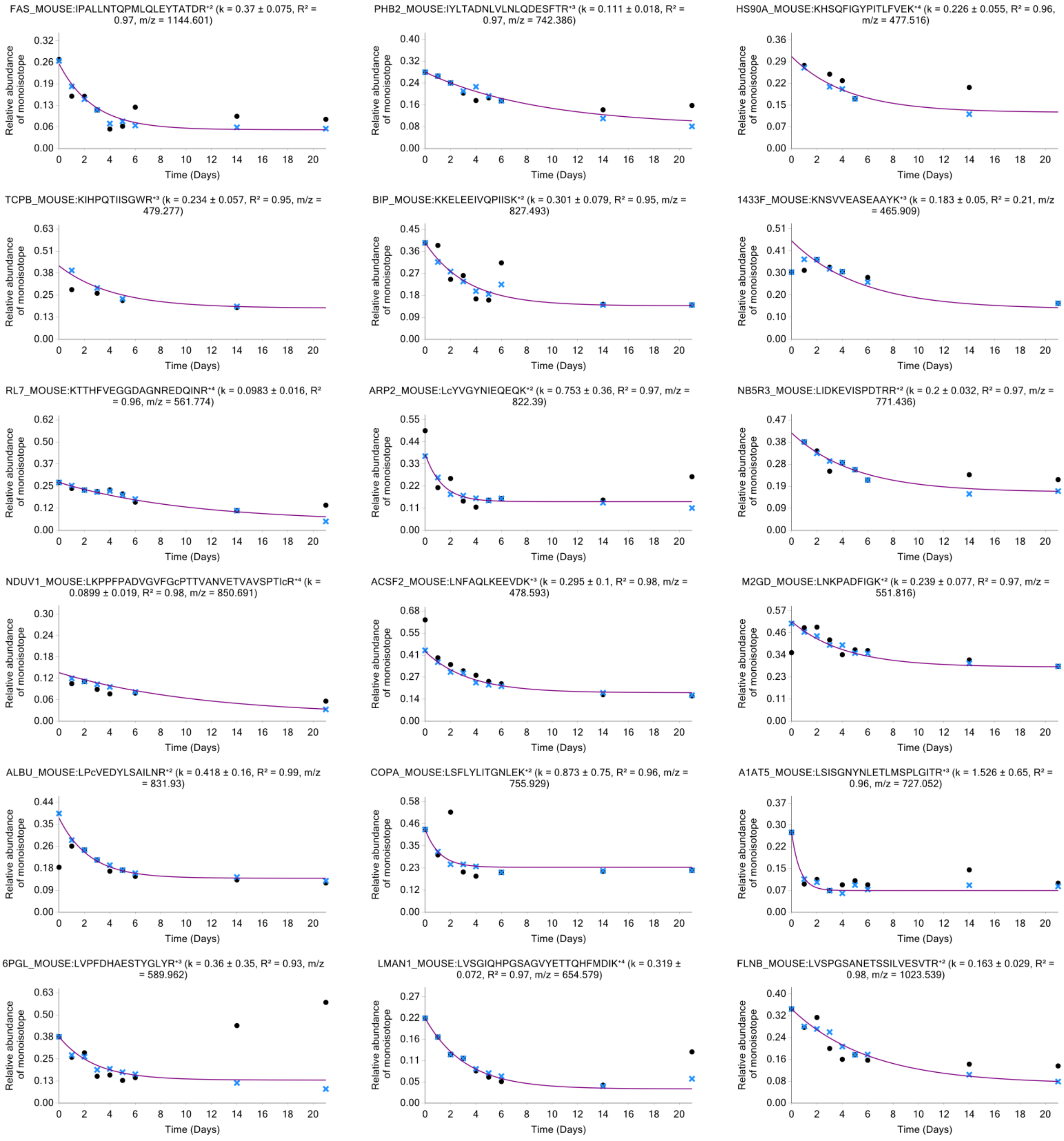

The Figures show the monoisotopic RIAs estimated from the complete isotope profile (black circles), RIA estimates from the complete isotope profiles, and any of the three partial isotope profiles combined ($A_1(t)/A_0(t)$, $A_2(t)/A_0(t)$, $A_2(t)/A_1(t)$) (blue crosses). The solid line shows the fit from the label enrichment determination technique.

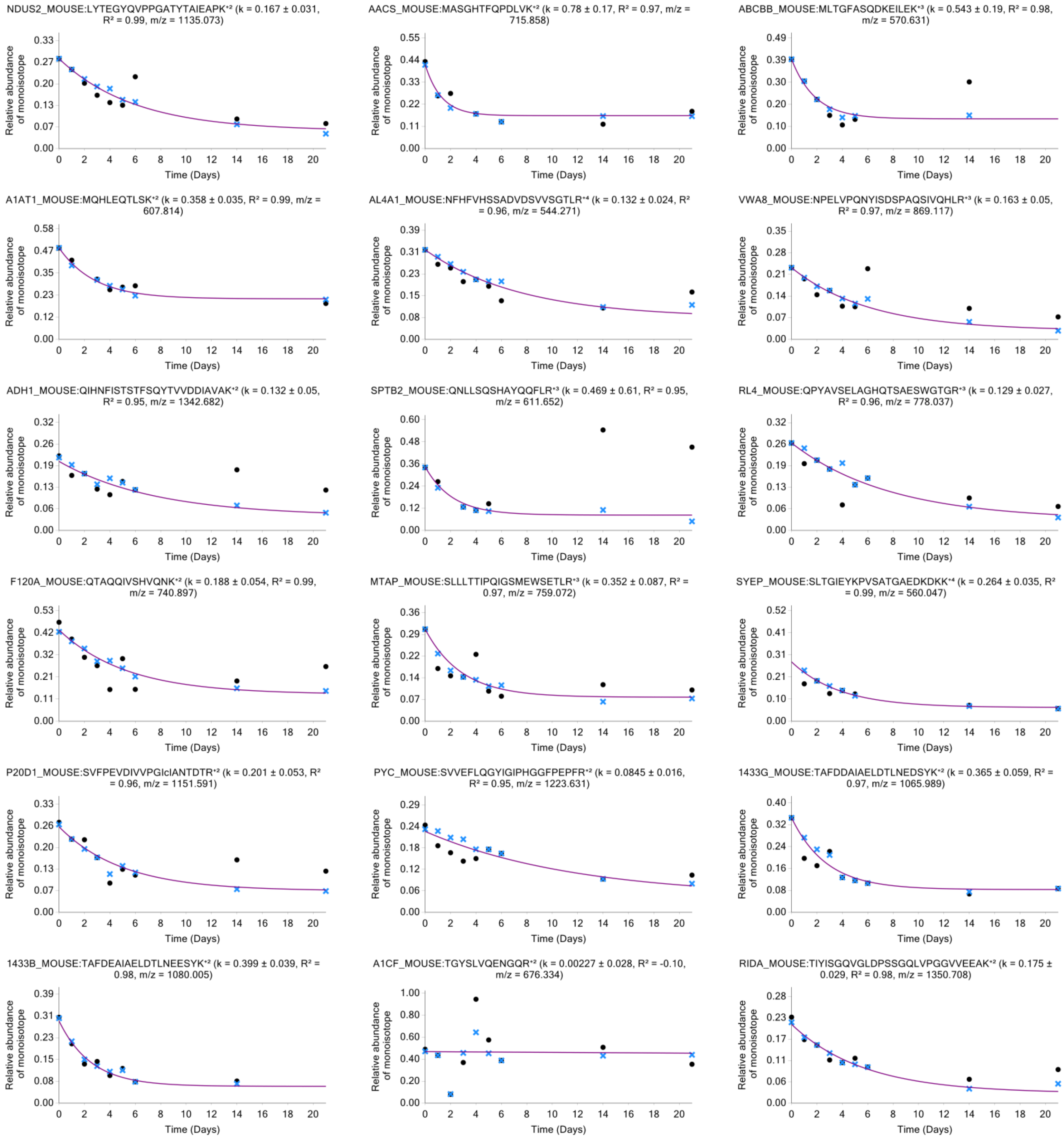

The Figures show the monoisotopic RIAs estimated from the complete isotope profile (black circles), RIA estimates from the complete isotope profiles, and any of the three partial isotope profiles combined ($A_1(t)/A_0(t)$, $A_2(t)/A_0(t)$, $A_2(t)/A_1(t)$) (blue crosses). The solid line shows the fit from the label enrichment determination technique.

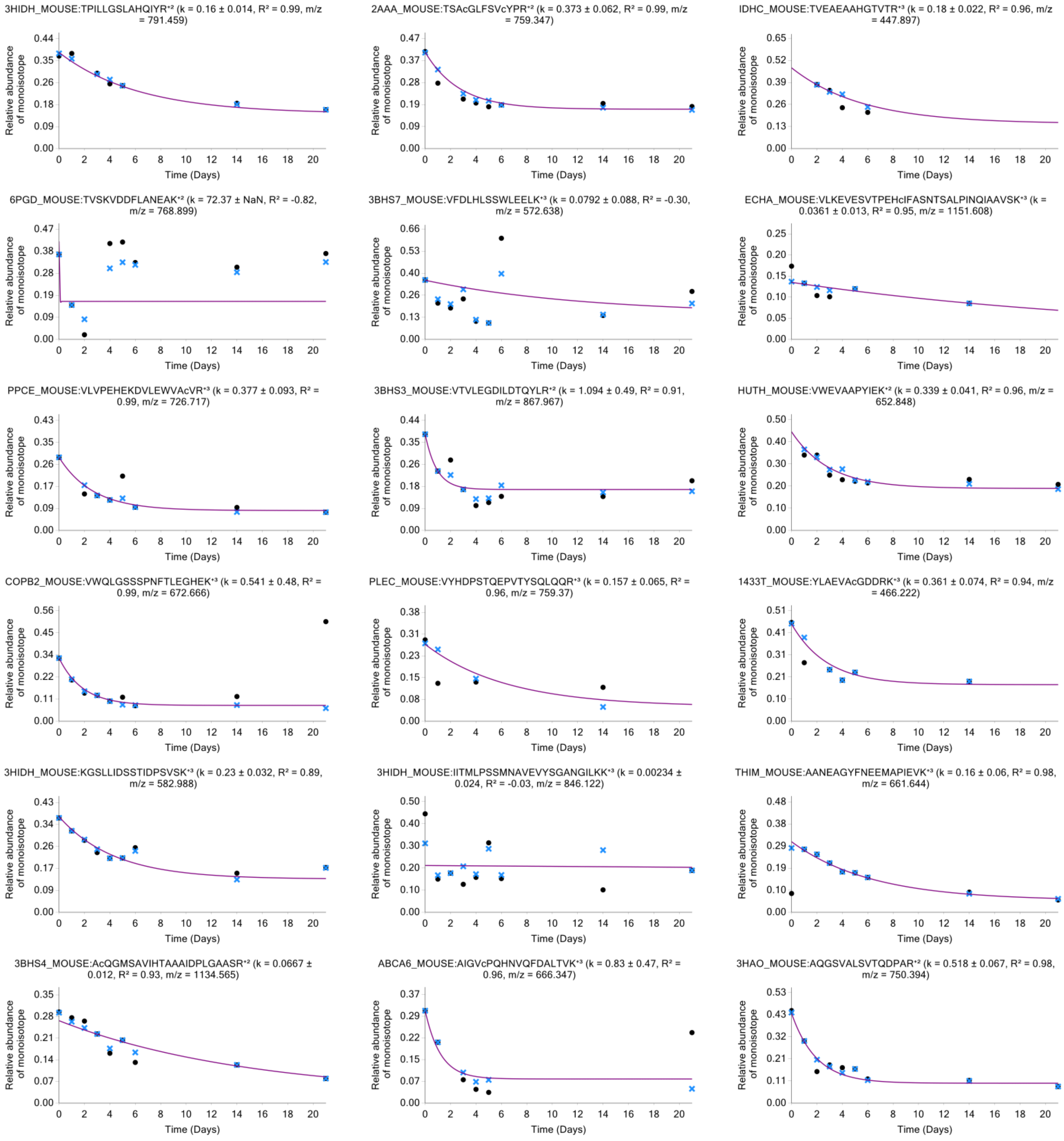

The Figures show the monoisotopic RIAs estimated from the complete isotope profile (black circles), RIA estimates from the complete isotope profiles, and any of the three partial isotope profiles combined ($A_1(t)/A_0(t)$, $A_2(t)/A_0(t)$, $A_2(t)/A_1(t)$) (blue crosses). The solid line shows the fit from the label enrichment determination technique.

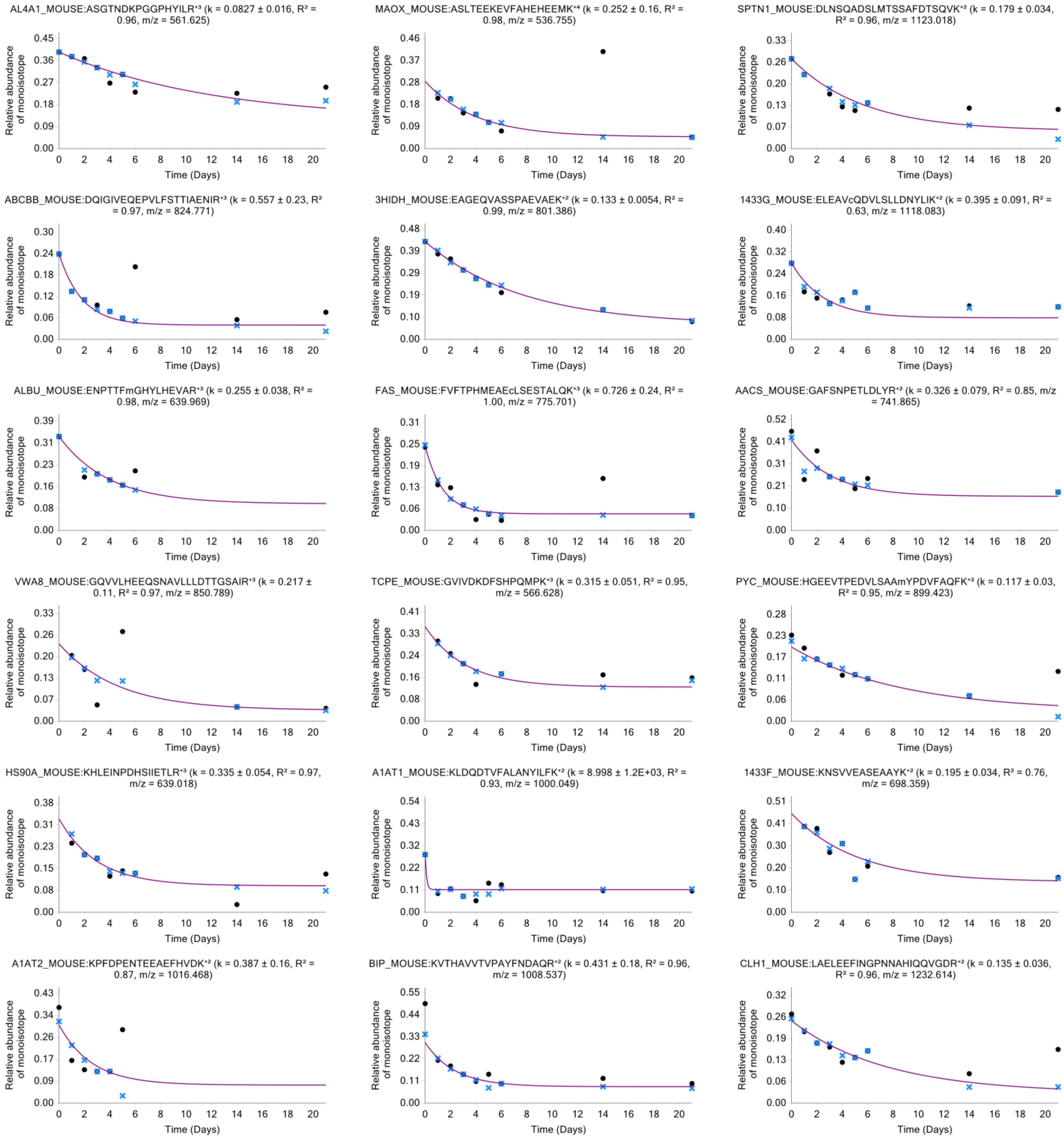

The Figures show the monoisotopic RIAs estimated from the complete isotope profile (black circles), RIA estimates from the complete isotope profiles, and any of the three partial isotope profiles combined ($A_1(t)/A_0(t)$, $A_2(t)/A_0(t)$, $A_2(t)/A_1(t)$) (blue crosses). The solid line shows the fit from the label enrichment determination technique.

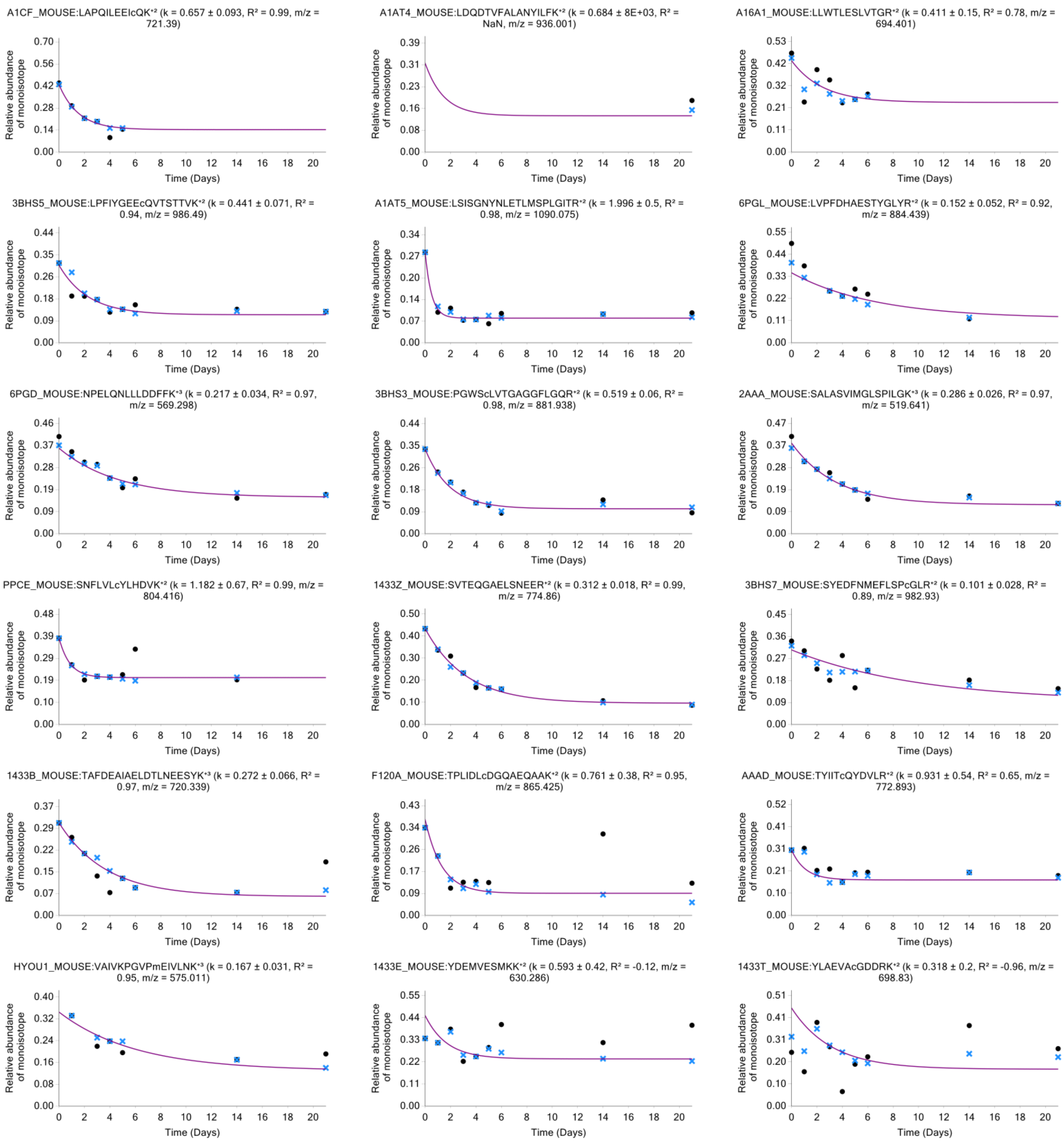

The Figures show the monoisotopic RIAs estimated from the complete isotope profile (black circles), RIA estimates from the complete isotope profiles, and any of the three partial isotope profiles combined ($A_1(t)/A_0(t)$, $A_2(t)/A_0(t)$, $A_2(t)/A_1(t)$) (blue crosses). The solid line shows the fit from the label enrichment determination technique.

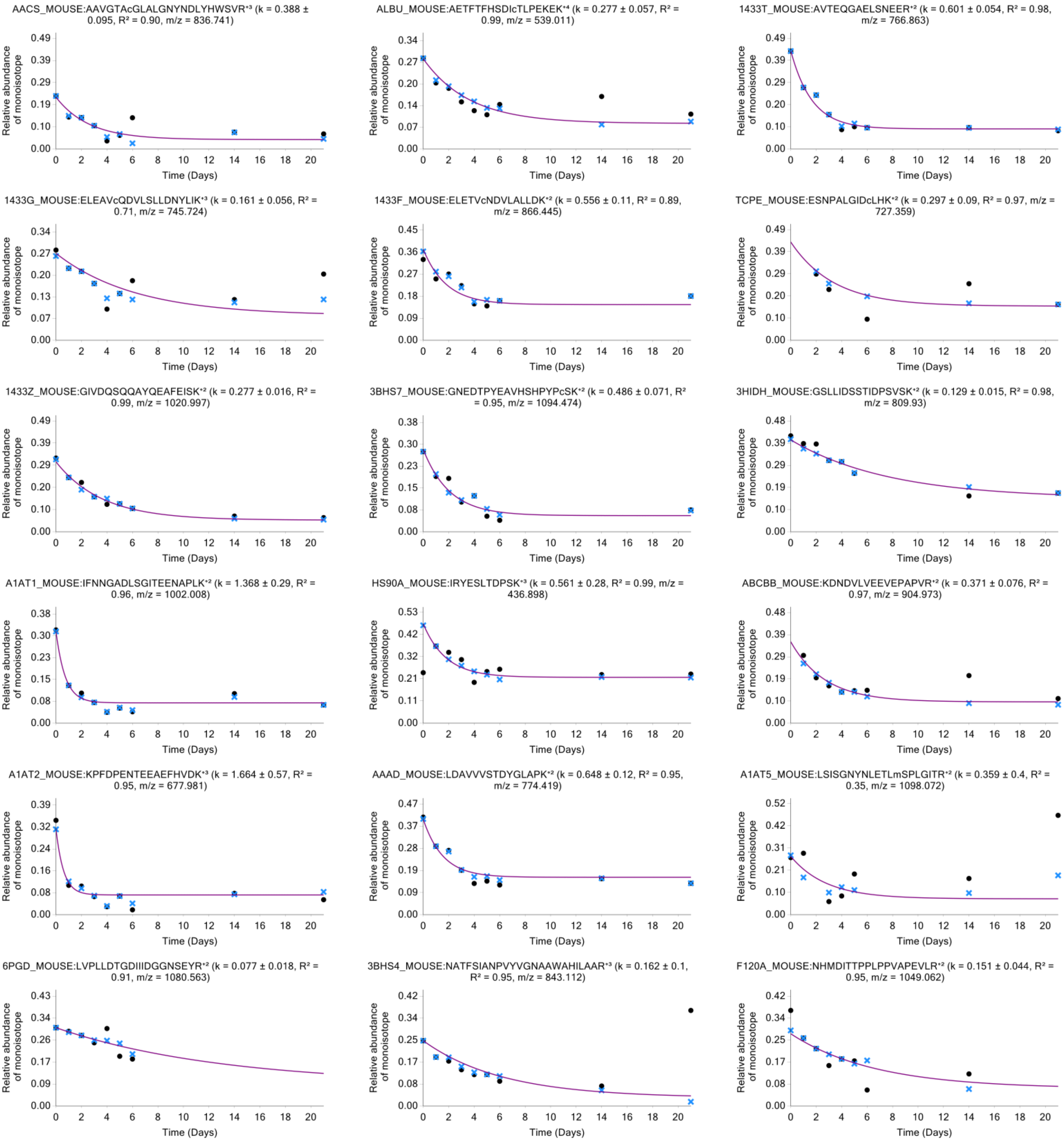

The Figures show the monoisotopic RIAs estimated from the complete isotope profile (black circles), RIA estimates from the complete isotope profiles, and any of the three partial isotope profiles combined ($A_1(t)/A_0(t)$, $A_2(t)/A_0(t)$, $A_2(t)/A_1(t)$) (blue crosses). The solid line shows the fit from the label enrichment determination technique.

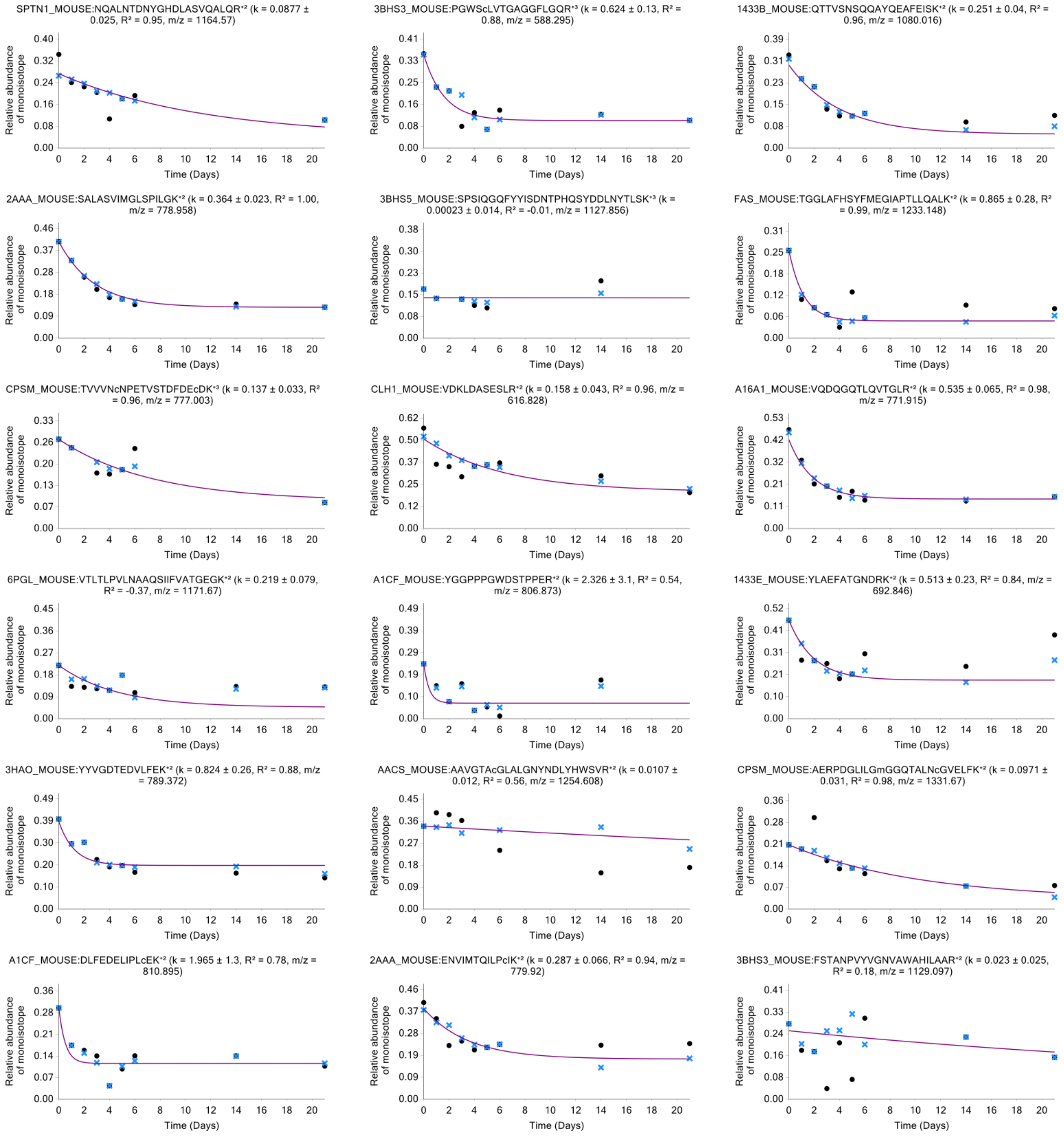

The Figures show the monoisotopic RIAs estimated from the complete isotope profile (black circles), RIA estimates from the complete isotope profiles, and any of the three partial isotope profiles combined ($A_1(t)/A_0(t)$, $A_2(t)/A_0(t)$, $A_2(t)/A_1(t)$) (blue crosses). The solid line shows the fit from the label enrichment determination technique.

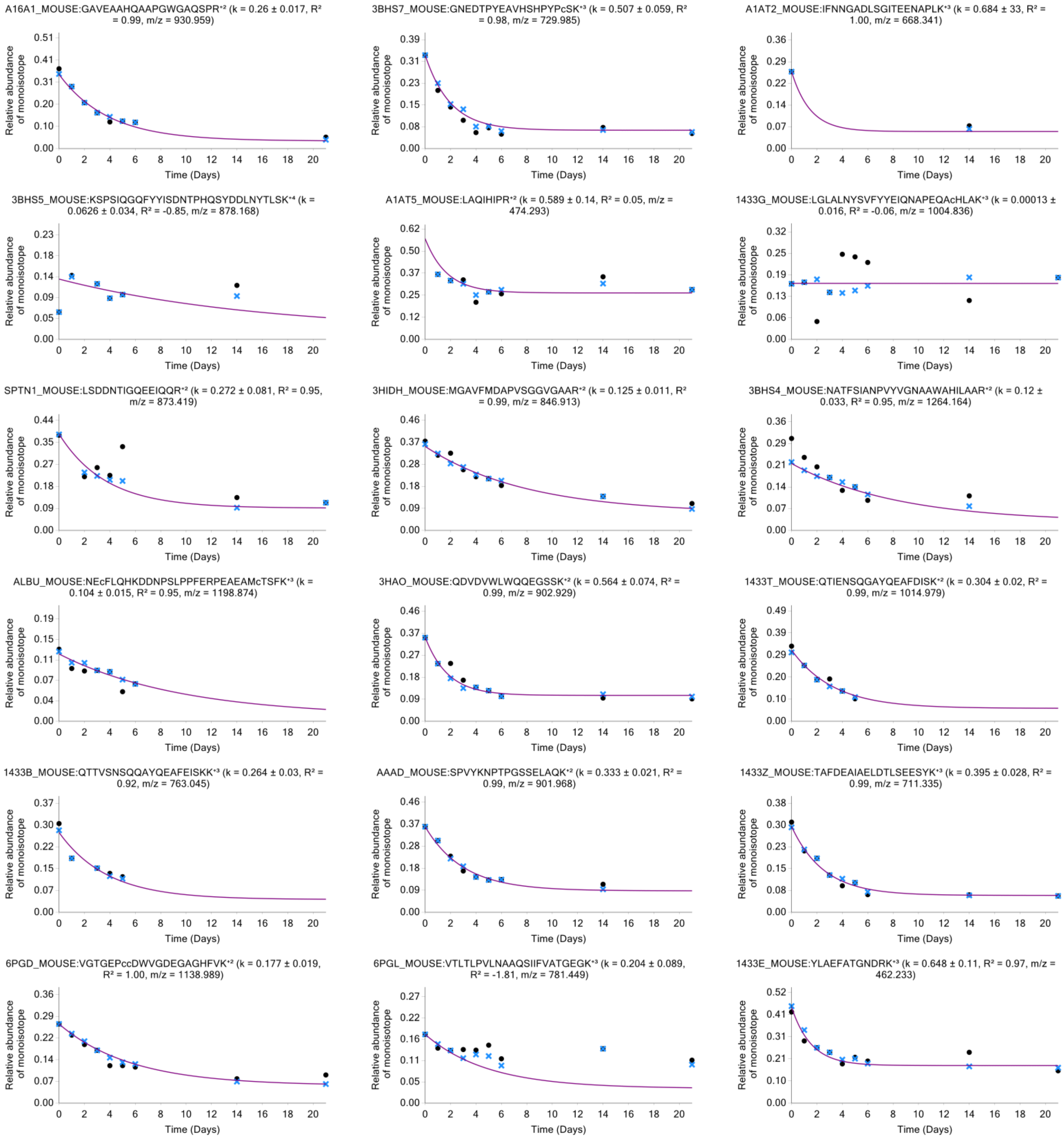

The Figures show the monoisotopic RIAs estimated from the complete isotope profile (black circles), RIA estimates from the complete isotope profiles, and any of the three partial isotope profiles combined ($A_1(t)/A_0(t)$, $A_2(t)/A_0(t)$, $A_2(t)/A_1(t)$) (blue crosses). The solid line shows the fit from the label enrichment determination technique.

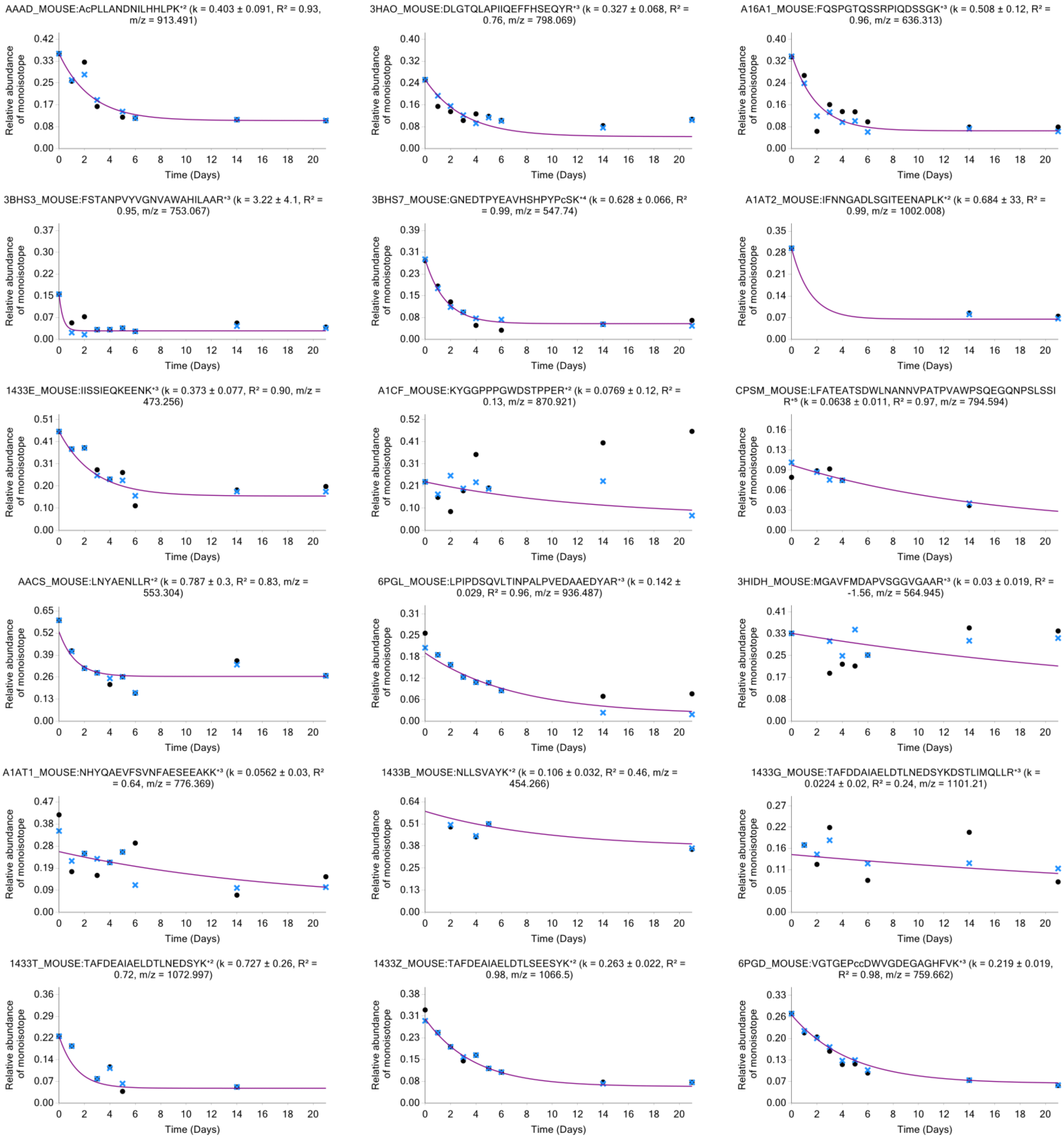

The Figures show the monoisotopic RIAs estimated from the complete isotope profile (black circles), RIA estimates from the complete isotope profiles, and any of the three partial isotope profiles combined ($A_1(t)/A_0(t)$, $A_2(t)/A_0(t)$, $A_2(t)/A_1(t)$) (blue crosses). The solid line shows the fit from the label enrichment determination technique.

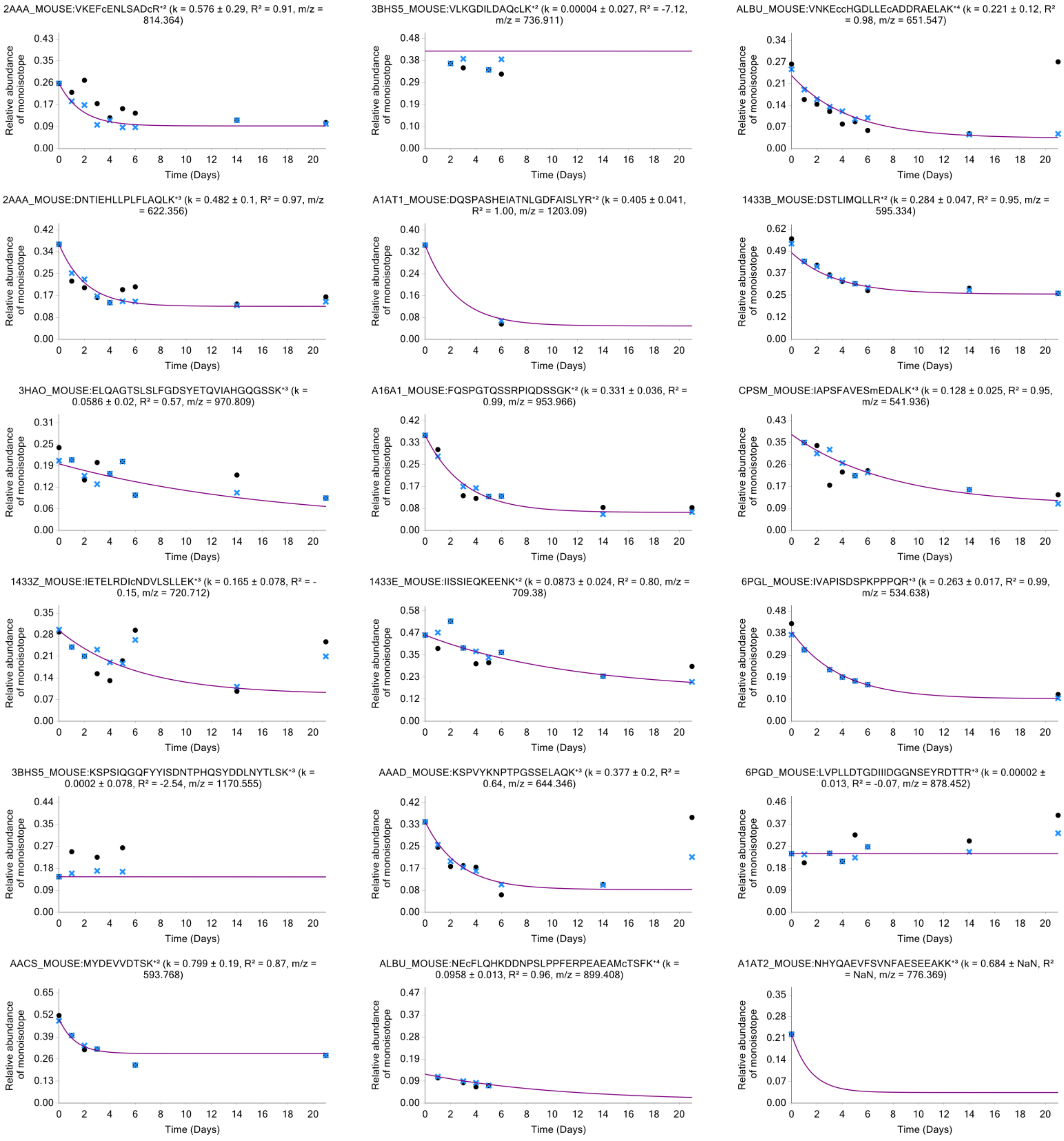

The Figures show the monoisotopic RIAs estimated from the complete isotope profile (black circles), RIA estimates from the complete isotope profiles, and any of the three partial isotope profiles combined ($A_1(t)/A_0(t)$, $A_2(t)/A_0(t)$, $A_2(t)/A_1(t)$) (blue crosses). The solid line shows the fit from the label enrichment determination technique.

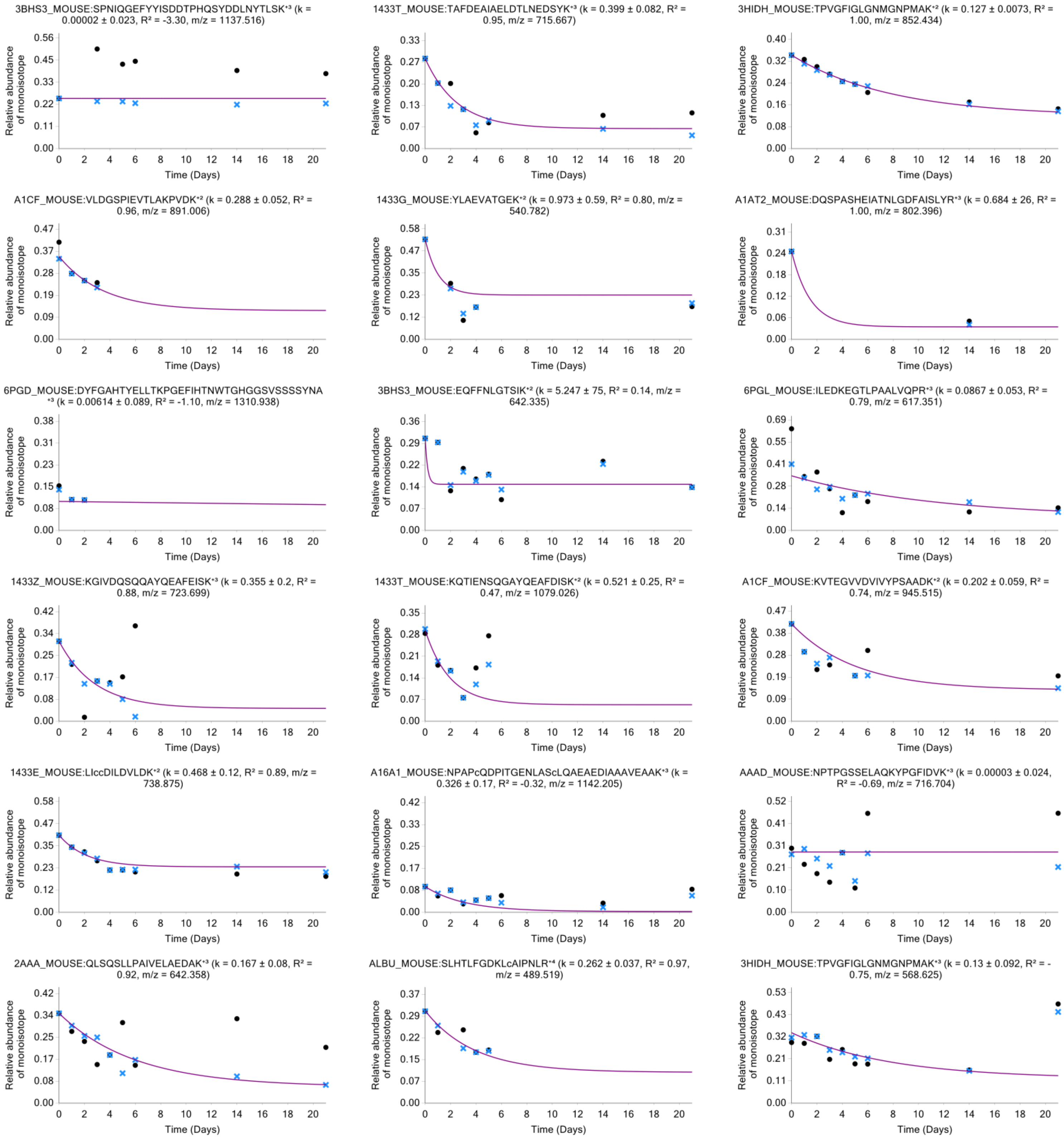

The Figures show the monoisotopic RIAs estimated from the complete isotope profile (black circles), RIA estimates from the complete isotope profiles, and any of the three partial isotope profiles combined ($A_1(t)/A_0(t)$, $A_2(t)/A_0(t)$, $A_2(t)/A_1(t)$) (blue crosses). The solid line shows the fit from the label enrichment determination technique.

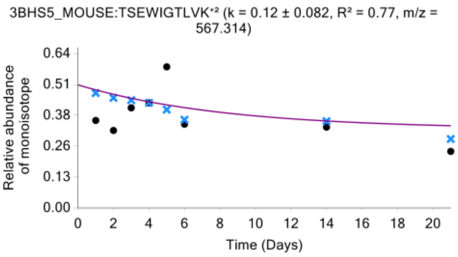

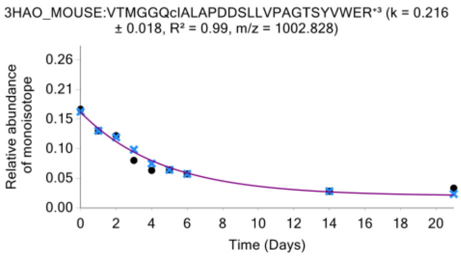

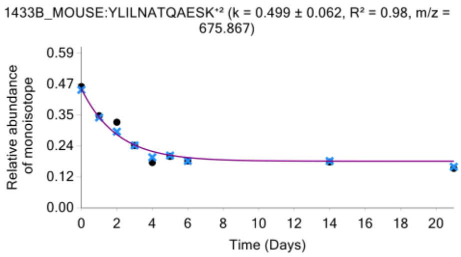

The Figures show the monoisotopic RIAs estimated from the complete isotope profile (black circles), RIA estimates from the complete isotope profiles, and any of the three partial isotope profiles combined ($A_1(t)/A_0(t)$, $A_2(t)/A_0(t)$, $A_2(t)/A_1(t)$) (blue crosses). The solid line shows the fit from the label enrichment determination technique.
