# Supplementary material for: Quantifying label enrichment from two mass isotopomers increases proteome coverage for in vivo protein turnover using heavy water metabolic labeling
Source: Commun Chem. 2023 Apr 17;6:72. doi: 10.1038/s42004-023-00873-x (PMC10110577; doi:10.1038/s42004-023-00873-x)
Supplement: Supplementary file 9 — Reporting Summary [file 42004_2023_873_MOESM9_ESM.pdf]

nature portfolio

Corresponding author(s): Rovshan G. Sadygov

Last updated by author(s): 09/16/2022

Reporting Summary

Nature Portfolio wishes to improve the reproducibility of the work that we publish. This form provides structure for consistency and transparency in reporting. For further information on Nature Portfolio policies, see our Editorial Policies and the Editorial Policy Checklist.

Statistics

For all statistical analyses, confirm that the following items are present in the figure legend, table legend, main text, or Methods section.

n/a	Confirmed	
☐	☒	The exact sample size (n) for each experimental group/condition, given as a discrete number and unit of measurement
☐	☒	A statement on whether measurements were taken from distinct samples or whether the same sample was measured repeatedly
☐	☒	The statistical test(s) used AND whether they are one- or two-sided<br>*Only common tests should be described solely by name; describe more complex techniques in the Methods section.*
☒	☐	A description of all covariates tested
☐	☒	A description of any assumptions or corrections, such as tests of normality and adjustment for multiple comparisons
☐	☒	A full description of the statistical parameters including central tendency (e.g. means) or other basic estimates (e.g. regression coefficient) AND variation (e.g. standard deviation) or associated estimates of uncertainty (e.g. confidence intervals)
☐	☒	For null hypothesis testing, the test statistic (e.g. F, t, r) with confidence intervals, effect sizes, degrees of freedom and P value noted<br>*Give P values as exact values whenever suitable.*
☒	☐	For Bayesian analysis, information on the choice of priors and Markov chain Monte Carlo settings
☒	☐	For hierarchical and complex designs, identification of the appropriate level for tests and full reporting of outcomes
☐	☒	Estimates of effect sizes (e.g. Cohen's d, Pearson's r), indicating how they were calculated

Our web collection on statistics for biologists contains articles on many of the points above.

Software and code

Policy information about availability of computer code

Data collection	The approach to estimate label enrichment from two mass isotopomers followed by protein turnover rate calculations was developed, benchmarked, and validated using publicly available and newly generated data. The latter was acquired in the data-dependent acquisition mode using ThermoFishers' Orbitrap Eclipse. The version of the XCalibur used for mass spectral data acquisition was 4.4.16.14.
Data analysis	MSCovertGUI (proteowizard version 3.0.10577) was used to convert the mass spectral data from the vendor's .raw format to the .mzML format. Centroiding of peaks in MS1 was done using the vendor's software in the proteowizard. Protein/peptide identification was performed using Mascot version 2.7.0. SwissProt database was downloaded on May 12th of 2021. Protein turnover rates were generated using d2ome+ version 1.05. Protein turnover results were analyzed using statistical and visualization tools in Python version 3.9.7.

For manuscripts utilizing custom algorithms or software that are central to the research but not yet described in published literature, software must be made available to editors and reviewers. We strongly encourage code deposition in a community repository (e.g. GitHub). See the Nature Portfolio guidelines for submitting code & software for further information.

Data

Policy information about availability of data

All manuscripts must include a data availability statement. This statement should provide the following information, where applicable:

- Accession codes, unique identifiers, or web links for publicly available datasets
- A description of any restrictions on data availability
- For clinical datasets or third party data, please ensure that the statement adheres to our policy

The raw mass spectral files in vendors' .raw and mzML formats, Mascot database search results in the .mzid format, and outputs from d2ome+ in .csv format are provided in MassIVE. The other dataset was generated in a different project. It is publicly available via the ProteomeXchange repository with the dataset identifier PXD029639.

Human research participants

Policy information about studies involving human research participants and Sex and Gender in Research.

Reporting on sex and gender	*Use the terms sex (biological attribute) and gender (shaped by social and cultural circumstances) carefully in order to avoid confusing both terms. Indicate if findings apply to only one sex or gender; describe whether sex and gender were considered in study design whether sex and/or gender was determined based on self-reporting or assigned and methods used. Provide in the source data disaggregated sex and gender data where this information has been collected, and consent has been obtained for sharing of individual-level data; provide overall numbers in this Reporting Summary. Please state if this information has not been collected. Report sex- and gender-based analyses where performed, justify reasons for lack of sex- and gender-based analysis.*
Population characteristics	*Describe the covariate-relevant population characteristics of the human research participants (e.g. age, genotypic information, past and current diagnosis and treatment categories). If you filled out the behavioural & social sciences study design questions and have nothing to add here, write "See above."*
Recruitment	*Describe how participants were recruited. Outline any potential self-selection bias or other biases that may be present and how these are likely to impact results.*
Ethics oversight	*Identify the organization(s) that approved the study protocol.*

Note that full information on the approval of the study protocol must also be provided in the manuscript.

Field-specific reporting

Please select the one below that is the best fit for your research. If you are not sure, read the appropriate sections before making your selection.

☒ Life sciences ☐ Behavioural & social sciences ☐ Ecological, evolutionary & environmental sciences

For a reference copy of the document with all sections, see nature.com/documents/nr-reporting-summary-flat.pdf

Life sciences study design

All studies must disclose on these points even when the disclosure is negative.

Sample size	The sample size for comparison of improvements by the data analysis approach was based on two considerations: the number of identified peptides in each experiment and the number of labeling time points. The number of peptides was important to the performance evaluation of the approach. The number of labeling time points improved the goodness-of-fit and confidence intervals of the calculated rates. The software benchmarks assessed the number of quantified proteins and peptides. The number of peptides quantified with the highest (> .95) coefficient has more than doubled. The corresponding number of proteins increased by 80%. The numbers identified/quantified peptides and the number of labeling time points (n >= 4) followed the standard practice in the field. The newly acquired data sets had nine, and the publicly available data set had eleven labeling time points.
Data exclusions	No data were excluded from the analysis.
Replication	All replications were successful.
Randomization	Randomization was not relevant for this study. The study presents computational benchmarks of two types of approaches for protein turnover rate estimation mass spectral data of stable isotope-labeled samples.
Blinding	In this study, the only intervention was the labeling duration. The mice were chosen randomly for time points of labeling duration. Blinding was not considered relevant to this study. The experiments were performed in an unbiased automatic fashion.

Reporting for specific materials, systems and methods

We require information from authors about some types of materials, experimental systems and methods used in many studies. Here, indicate whether each material, system or method listed is relevant to your study. If you are not sure if a list item applies to your research, read the appropriate section before selecting a response.

Materials & experimental systems

n/a	Involved in the study
☒	☐ Antibodies
☒	☐ Eukaryotic cell lines
☒	☐ Palaeontology and archaeology
☐	☒ Animals and other organisms
☒	☐ Clinical data
☒	☐ Dual use research of concern

Methods

n/a	Involved in the study
☒	☐ ChIP-seq
☒	☐ Flow cytometry
☒	☐ MRI-based neuroimaging

Animals and other research organisms

Policy information about studies involving animals; ARRIVE guidelines recommended for reporting animal research, and Sex and Gender in Research

Laboratory animals	Eighteen, four-month-old, C57/BL6J male mice were used in the study.
Wild animals	The study did not involve wild animals.
Reporting on sex	Male mice was used in this study.
Field-collected samples	The study did not involve samples collected from the field.
Ethics oversight	All mice experiments were carried out according to the Guide for Care and Use of Laboratory Animals by the National Research Council and approved by the Institutional Animal Care and Use Committee of UTMB.

Note that full information on the approval of the study protocol must also be provided in the manuscript.
